# Contact pattern, current immune barrier, and pathogen virulence determines the optimal strategy of further vaccination

**DOI:** 10.1016/j.idm.2023.01.003

**Published:** 2023-01-13

**Authors:** Xiaohao Guo, Ziyan Liu, Shiting Yang, Zeyu Zhao, Yichao Guo, Guzainuer Abudurusuli, Shanlu Zhao, Ge Zeng, Shixiong Hu, Kaiwei Luo, Tianmu Chen

**Affiliations:** a State Key Laboratory of Molecular Vaccinology and Molecular Diagnostics, School of Public Health, Xiamen University, Xiamen City, 361102, Fujian Province, People's Republic of China; b Hunan Provincial Center for Disease Control and Prevention, 405 Furong Middle Road Section One, Kaifu District, Changsha City, 410001, Hunan Province, People's Republic of China

**Keywords:** Vaccine, Allocation strategy, SARS-CoV-2, Optimal control, Immune barrier, Contact pattern, Greedy algorithm

## Abstract

**Background:**

The current outbreak of novel coronavirus disease 2019 has caused a serious disease burden worldwide. Vaccines are an important factor to sustain the epidemic. Although with a relatively high-vaccination worldwide, the decay of vaccine efficacy and the arising of new variants lead us to the challenge of maintaining a sufficient immune barrier to protect the population.

**Method:**

A case-contact tracking data in Hunan, China, is used to estimate the contact pattern of cases for scenarios including school, workspace, etc, rather than ordinary susceptible population. Based on the estimated vaccine coverage and efficacy, a multi-group vaccinated-exposed-presymptomatic-symptomatic-asymptomatic-removed model (*VEFIAR*) with 8 age groups, with each partitioned into 4 vaccination status groups is developed. The optimal dose-wise vaccinating strategy is optimized based on the currently estimated immunity barrier of coverage and efficacy, using the greedy algorithm that minimizes the cumulative cases, population size of hospitalization and fatality respectively in a certain future interval. Parameters of Delta and Omicron variants are used respectively in the optimization.

**Results:**

The estimated contact matrices of cases showed a concentration on middle ages, and has compatible magnitudes compared to estimations from contact surveys in other studies. The *VEFIAR* model is numerically stable. The optimal controled vaccination strategy requires immediate vaccination on the un-vaccinated high-contact population of age 30–39 to reduce the cumulative cases, and is stable with different basic reproduction numbers ($R_{0}$). As for minimizing hospitalization and fatality, the optimized strategy requires vaccination on the un-vaccinated of both aged 30–39 of high contact frequency and the vulnerable older.

**Conclusion:**

The objective of reducing transmission requires vaccination in age groups of the highest contact frequency, with more priority for un-vaccinated than un-fully or fully vaccinated. The objective of reducing total hospitalization and fatality requires not only to reduce transmission but also to protect the vulnerable older. The priority changes by vaccination progress. For any region, if the local contact pattern is available, then with the vaccination coverage, efficacy, and disease characteristics of relative risks in heterogeneous populations, the optimal dose-wise vaccinating process will be obtained and gives hints for decision-making.

## Graphical abstract

**Figure:**
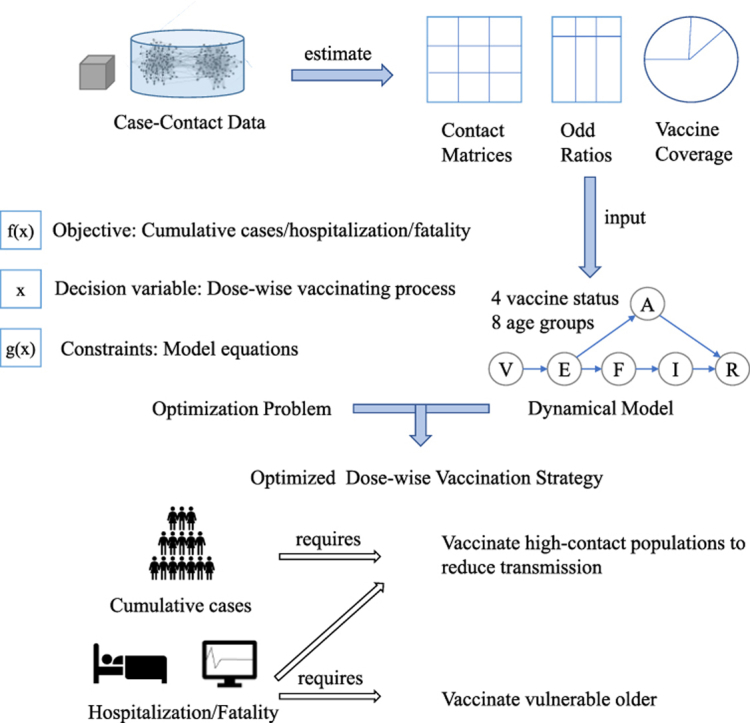

## 1 Introduction

At the end of 2019, the novel coronavirus disease 2019 caused by severe acute respiratory syndrome coronavirus 2 (SARS-CoV-2) first broke out in Wuhan, China, and then swept across the world at an extremely rapid pace. As of September 27, 2022, a total of 612,236,677 confirmed cases globally with 6,514,397 deaths had been reported by WHO (Organization). Although several targeted interventions have been implemented globally to contain the spread of the new crown epidemic, the situation remains challenging as dominant variant strain updates one by another. A safe and effective new-coronavirus vaccine is essential to reduce transmission, ICUs, mobility and even end the outbreak of novel coronavirus. Although there was a high vaccine coverage with fully vaccinations, the vaccine efficacy (VE) in preventing infection is not satisfying. The emergence of new variants poses a challenge to the effectiveness of the new-coronavirus vaccine. The US Centers for Disease Control and Prevention began recommending that all people who receive the Johnson & Johnson vaccine receive a booster at least 2 months in advance (Cohn et al., 2022). Regular vaccinations help to maintain antibody levels in the body and a booster shot of the new-coronavirus vaccine may be the general trend.

Vaccines are a powerful tool in the current fight against the epidemic, but with limited supplies, it is important to consider who should be prioritized for vaccination and what vaccine distribution scheme should be implemented to maximize health and economic benefits. WHO believes that those who could benefit from safe and effective SARS-CoV-2 vaccines should have access as quickly as possible, starting with those at the highest risk of serious disease or death (Organization, 2021). As age is one of the risk factors for serious SARS-CoV-2 infections and death (Gao et al., 2020), the elderly population is the most frequently cited priority target group (Noh et al., 2021) (Matrajt et al., 2021a) (Moore et al., 2021) (Zhao et al., 2021), but studies have also suggested that there is a strong moral case for giving children priority for vaccination to protect the elderly (Matrajt et al., 2021b) (Giubilini et al., 2020). Depending on the objectives, several studies have suggested that priority should be given to vaccinating the elderly population against the new coronavirus to reduce the number of seriously ill patients and reduce mortality, and to the young population to reduce the spread of the new coronavirus (Buckner et al., 2021) (Bubar et al., 2021). According to the effectiveness and availability of the vaccine, it has been suggested that in cases of low vaccine effectiveness and low vaccine availability, it is preferable to allocate the vaccine to the higher age groups first; in cases of high vaccine effectiveness and high vaccine coverage, it is preferable to allocate the vaccine to the lower age (Matrajt et al., 2021b). As the dynamics of the novel coronavirus pneumonia epidemic and the status of vaccine supply continue to change, we should adjust our immunization strategy promptly and identify priority groups for vaccination.

Most of those studies considers static allocation of vaccination instead of a dose-wise or time-varying vaccination process. Besides, the dynamical model of SIR (susceptible-infectious-removed), SEIR (susceptible-exposed-infectious-removed), and SEIAR (susceptible-exposed-symptomatic/asymptomatic infectious-removed) does not precisely represent the natural history of SARS-CoV-2, where a considerable pre-symptomatic infectious stage acts in tranmission.

To have a better understanding of the impact on age-specific contact patterns and immune barrier in the optimal vaccination strategy, this study analyzed local exposure patterns and vaccine effectiveness using case-contact data from Hunan Province, simulated the transmission of age groups and computed the optimal dose-wise vaccination strategy. A vaccinated-exposed-presymptomatic-symptomatic/asymptomatic-removed (VEFIAR) ordinary differential equation model is developed with a pre-symptomatic stage for better approximation of natural history of SARS-CoV-2. The dose-wise vaccination process is considered for optimization instead of a static vaccine distribution optimization for capturing more details. Unlike the most previous studies that simulates the age-grouped models on contact data of health people, this study considers the different behaviors between health people and cases by using data of close contact to construct the special contact pattern of cases and simulates.

## 2 Materials and methods

### 2.1 Data collection

A real world scenario of Delta variant outbreak in Hunan Province, China during July 22, 2021 to August 15, 2021 is choosed to illustrate the optimal vaccination strategy. Among all of 129 cases in this outbreak, 124 cases and their 13915 close contacts or sub-close contacts have finished a survey of close contact tracing. The information collected including age, sex, date of last contact, locations where the last contact taken placed, relation between case and close contacts, exposure ranking, and vaccination status. The definition and standard for judgement of close contact and sub-close contact is given by the Diagnosis and Treatment Protocol for Novel Coronavirus Pneumonia, Version 8 (Diagnosis and Treatment Protocol for, 2021). Data of all cases and their close contacts are provided by Hunan Provincial Centers for Disease Control and Prevention.

### 2.2 Statistical analysis

We first evaluate the vaccine coverage in population by inspecting the four vaccination status: none, un-fully vaccinated, fully vaccinated, and booster vaccinated of close contacts in contact data.

The total attack rates (TARs) are computed for people in different age groups and people with different vaccination status. To handle the uncertainty of the collected data, we use bootstrap (Givens and Hoeting, 2012) to construct the empirical distribution of all these TARs. We performed the bootstrap 10000 times on the contact data, with each bootstrapped data set containing equally 10000 contacts. Each of those bootstrapped data sets produces an 8×4 matrix of TAR in age groups (in rows) and vaccine groups (in columns). Based on those resampled TARs, we depict the distribution of TAR in age groups and vaccination status groups in box-plot respectively.

The following logistic regression model is used for analyzing odds ratios (OR) of age groups and vaccination status groups:

$$\begin{array}{rr} logit\left(E\left\{Y|X=x\right\}\right)= & \left[1,X^{T}\right]\beta \\ \left\{Y|X=x\right\}∼ & binomial\;distribution \end{array}$$

where $logit\left(x\right)=\ln\left(\frac{x}{1-x}\right)$ is the canonical link function of binomial distribution; E denote the expectation operation of random variables; Y is the 0–1 random variable of whether a close contact is infected (1 represent infected, 0 represent un-infected); X is a 4×1 random vector with its entries be 4 factors: vaccination status, contact age, contact gender and exposure ranking; x is an observation of X; β is a 5×1 vector of coefficients (include coefficient of intercept).

Based on the coefficient of contact age and vaccination status, the odds ratio (OR) are computed for different age and vaccination status groups:

$$OR=e^{x_{1}^{T}\hat{\beta }-x_{0}^{T}\hat{\beta }}$$

where $\hat{\beta }$ is the fitted parameter vector of the logistic model; $x_{0}$ is the properties of reference population; $x_{1}$ is the properties of target population. (Note that the exposure ranking and the contact gender are the same in $x_{0}$ and $x_{1}$, and they are vanished by $x_{1}-x_{2}$)

### 2.3 Contact pattern analysis

We use the contact matrix to describe the current contact pattern. The ij-entry of the contact matrix is defined as the average number of contacts in group j that one individual in group i would produce during a period of one day. A bipartite graph model is developed to describe the properties of the contact matrix. We first construct the contact data matrix by simply fills all the case-contact data pairs in. Then, based on the bipartite graph model, three methods of least square, weighted least square, and maximum likelihood, are developed for estimating the contact matrix. The contact matrices are also estimated using the subset of case-contact data which contact occurs in specific scenarios of public transports, amusement, school, household, hospital, working area, service industry, community, catering industry, uncategorized setting, and empty settings in original data respectively.

### 2.4 Model development

To evaluate the effectiveness of vaccination and heterogeneity among age groups, a multi-group vaccinated-exposed-pre-symptomatic-symptomatic-asymptomatic-removed (VEFIAR) model is developed in ordinary differential equations. The vaccine efficacy (VE) is considered as coefficients of reducing susceptibility. In simulation, we use the odds ratio versus average age and average vaccination status, fitted in the logistic regression, to replace the coefficients (1–VE) in the newly infection term.

Modeling details including assumptions, interpretation of variable and parameters are all presented in Supplementary Tables S1–S4 and section 2.1 of the supplementary material.

The equations of the model is given by:

$$\begin{array}{rr} \frac{dV_{ij}}{dt}= & -\lambda_{i}\left(1-VE_{ij}\right)V_{ij}\\ \frac{dE_{ij}}{dt}= & \lambda_{i}\left(1-VE_{ij}\right)V_{ij}-\left(1-p_{ij}\right)\omega_{i}^{\prime }E_{ij}-p_{ij}\omega_{i}E_{ij}\\ \frac{dF_{ij}}{dt}= & \left(1-p_{ij}\right)\omega_{i}^{\prime }E_{ij}-\omega_{i}^{\prime \prime }F_{ij}\\ \frac{dI_{ij}}{dt}= & \omega_{i}^{\prime \prime }F_{ij}-\gamma_{i}I_{ij}\\ \frac{dA_{ij}}{dt}= & p_{ij}\omega_{i}E_{ij}-\gamma_{i}^{\prime }A_{ij}\\ \frac{dR_{ij}}{dt}= & \gamma_{i}^{\prime }A_{ij}+\gamma_{i}I_{ij} \end{array}$$

where subscript i=1,2,⋯,8 and j=0,1,2,3 denote the subgroups of age i and vaccination status j; $\lambda_{i}$ is the infection force acted equally on individuals in age group i produced by all infected population, and is defined as:

$$\lambda_{i}=\sum_{k=1}^{n}\beta_{ki}\left[\sum_{j=0}^{3}\left(I_{kj}+\kappa A_{kj}+\kappa^{\prime }F_{kj}\right)\right]\text{,}$$

where κ and $\kappa^{\prime }$ are coefficients of relative transmissibility of asymptomatic and pre-symptomatic population versus the symptomatic population. Then, $\lambda_{i}V_{ij}$ is the newly infection rate of subgroup ij. And by considering the vaccine efficacy inside age group i, we multiplies each $V_{ij}$ by a constant vaccine efficacy coefficient $1-VE_{ij}$. In practice, we use the data-estimated odd ratios $OR_{ij}$ versus average population (Table S7) to replace $1-VE_{ij}$. That is, the newly infection term is final obtained as: $-\lambda_{i}\left(1-VE_{ij}\right)V_{ij}=-\lambda_{i}OR_{ij}V_{ij}$.

To introduce the impact of contact patterns in the multi-group VEFIAR model, the transmission rate coefficients $\beta_{ij}$ are formularized as the products of the number of daily average contact freqency, the probability of infection after single one-time contact, and the susceptibility of people in the j-th group, that is,

$$\beta_{ij}N_{i}=c_{ji}q\sigma_{j}\text{,}$$

where $c_{ji}$ is the ji-th entry of contact matrix; q is the probability of infection by a single one-time contact; $\sigma_{j}$ is the susceptibility of group j.

The basic reproduction number $\left(R_{0}\right)$ computed by next-generation method is a function of all $\beta_{ij}$ and other parameters. With the assumption of the decomposition of $\beta_{ij}$ and the assumption of equal susceptibility in age groups, $R_{0}$ is indeed a function of contact matrix *C*, probability of contact infection q, all other parameters and the state of disease-free equilibrium. In simulations, the probability of infection q is solved from the pre-defined $R_{0}$, then all $\beta_{ij}$ are recovered from the contact matrix C and q.

The grouped VEFIAR model is tested by simulating with different $R_{0}$. The contact pattern involved for recovering all $\beta_{ij}$ from $R_{0}$ is set to be the contact matrix estimated via maximum likelihood estimation (MLE), and the first patient (indicator) is set to be in age group 30 to 39, with fully vaccinated status, just as it was in this outbreak. For simulations of each $R_{0}$, the daily incidence rates are shown for each vaccine group, and the total attack rates in 100-days are shown for each age and vaccine group in the supplementary material.

Table 1 interprets all parameters defined in the multi-group VEFIAR model.

**Table 1:** Parameter profiles.

Parameter	Interpretation	Value (Range)	Unit	Source
n	number of age groups	8	1	self-defined
$c_{ij}$	daily average number of contact in age group j that a individual in age group i will produce	estimated in Fig. 2	person⋅$day^{-1}$	data estimated
$\beta_{ij}$	transmission rate coefficient	–	$day^{-1}\cdot$$person^{-1}$	compute by $c_{ij}$ and $R_{0}$
$OR_{ij}$	the odd ratios of age group i in vaccination status group j versus the average population	–	1	by fitted logistic regression model
$\lambda_{i}$	force of infection uniformly acted on age group i	–	$day^{-1}$	compute from $\beta_{ij}$, $E_{ij}$, $A_{ij}$ and $I_{ij}$
$1/\omega_{ij}$	average latent period of those asymptomatic cases of vaccination status j in age group i	3	day	reference 25
$1/\omega_{ij}^{\prime }$	average latent period of those pre-symptomatic cases of vaccination status j in age group i	3	day	reference 25
$1/\omega_{ij}^{\prime \prime }$	average duration from pre-symptomatic $F_{ij}$ to symptomatic $I_{ij}$	2	day	reference 25
$p_{ij}$	probability that one exposed individual of vaccination status j in age group i will become asymptomatic	0.31 for un-vaccinated; 0.62 for vaccinated	1	reference 25
1/γ	average infectious period of those symptomatic of vaccination status j in age group i	5	day	reference 25
$1/\gamma^{\prime }$	average infectious period of those asymptomatic of vaccination status j in age group i	7	day	reference 25
κ	relative transmissibility of asymptomatic cases versus symptomatic cases	0.35	1	reference 25
$\kappa^{\prime }$	relative transmissibility of pre-symptomatic cases versus symptomatic cases	0.63	1	reference 25

### 2.5 Optimization for dose-wise vaccinating process

The optimization of dose-wise vaccinating process is indeed an optimal control problem that can be transformed into a nonlinear programming problem (NLP) (Betts, 2010) by discretizing the controls, and solved by a variety of numerical optimization methods. Typically, one may use back propagation for sub-gradient, and use stochastic gradient descent method for optimizing. However, in real world, the vaccinating process is not always parallel with abundant vaccines, rather than a small amount of step-wise vaccinating process. For this consideration, we use the greedy algorithm (Balaman and Yılmaz Balaman, 2019) to approximate the optimal vaccinating process under the current vaccine coverage. The function to be minimized (the objective function) is constructed as the cumulative number of cases by taking weighted summation of time integrals of all fluxes into $F_{ij}$ and $A_{ij}$ during the 14-days simulation. This objective can be also interpreted as if we assigned this dose to such group, then what reduction of cumulative cases are expected within the next 14-days. Other indicators of concerned, like morbidities and ICUs, can be obtained by weighting the cumulative cases by group-relevant morbidities and ICU ratios.

The directional derivative is introduced as an index for the effectiveness of the current dose vaccinated in different groups. The optimal step-wise vaccinating strategy is then obtained by minimizing the directional derivative for each dose. We visualized the optimal vaccine process by showing the population-size curves of each age groups and vaccine groups. Detail profiles of the optimization problem can be found in chapter 4 in the supplementary material.

All analysis and simulations are completed using MATLAB 2021b.

## 3 Result

### 3.1 Epidemiology characteristics and vaccine efficacy

**Fig. 1:**
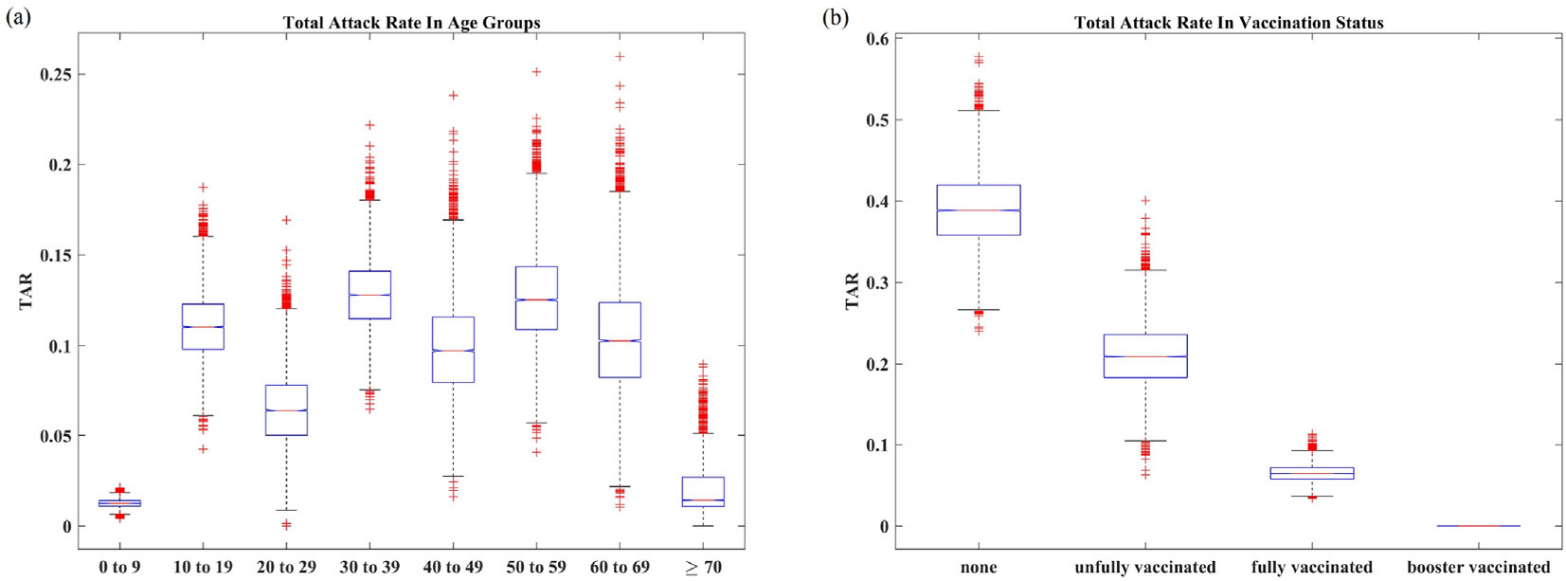
Total Attack Rates (TARs). (a) TAR in different age groups; (b) TAR in different vaccination status groups. The method of bootstrap is used to illustrate the distribution of TAR in specific age-vaccination groups. 10000 times of bootstrap of the close contact data is performed, with bootstrap sample size equals 10000. Each bootstrapped data set produces a sample matrix of TAR (of each age and vaccination status group).

The boxplot in Fig. 1(a) shows the different distribution of bootstrapped total attack rates (TARs) in age groups and vaccine groups. The TARs showed no clear trend in age groups, and does not change much among groups of middle age, which could be interpreted by the mixture effects of factors like different vaccine coverage among age groups. The medians of TARs in all age groups are around 10%. TARs in the youngest age group and the oldest age group are lowest, which can be explained by its lower contact rate. One can conclude TAR in one group is greater than the other group with 95% confidence by inspecting whether the notches of boxplots overlaps.

For the vaccine groups in Fig. 1(b), similarly by inspecting the notches, we conclude with 95% confidence that the median of TAR decrease by doses.

The coefficients fitted in the logistic regression model are all significantly differ from 0, except the contact gender (Table S5). The odds ratios versus the un-vaccinated of age greater than 70 and the odds ratios versus the average age (38.6 year old) of average vaccine status are then computed using the fitted coefficients (Table 2 and Table S7).

**Table 2:** Odds Ratio for Age and Vaccination Groups (versus ≥70, un-vaccinated).

	non−vaccinated	un−fullyvaccinated	fullyvaccinated	boostervaccinated
0to9	7.4867e−01	3.8872e−01	2.0182e−01	1.0479e−01
10to19	7.8028e−01	4.0513e−01	2.1034e−01	1.0921e−01
20to29	8.1322e−01	4.2223e−01	2.1922e−01	1.1382e−01
30to39	8.4755e−01	4.4006e−01	2.2848e−01	1.1863e−01
40to49	8.8333e−01	4.5863e−01	2.3813e−01	1.2364e−01
50to59	9.2063e−01	4.7800e−01	2.4818e−01	1.2886e−01
60to69	9.5949e−01	4.9818e−01	2.5866e−01	1.3430e−01
≥70	1.0000e+00	5.1921e−01	2.6958e−01	1.3997e−01

### 3.2 Analysis of contact pattern

**Fig. 2:**
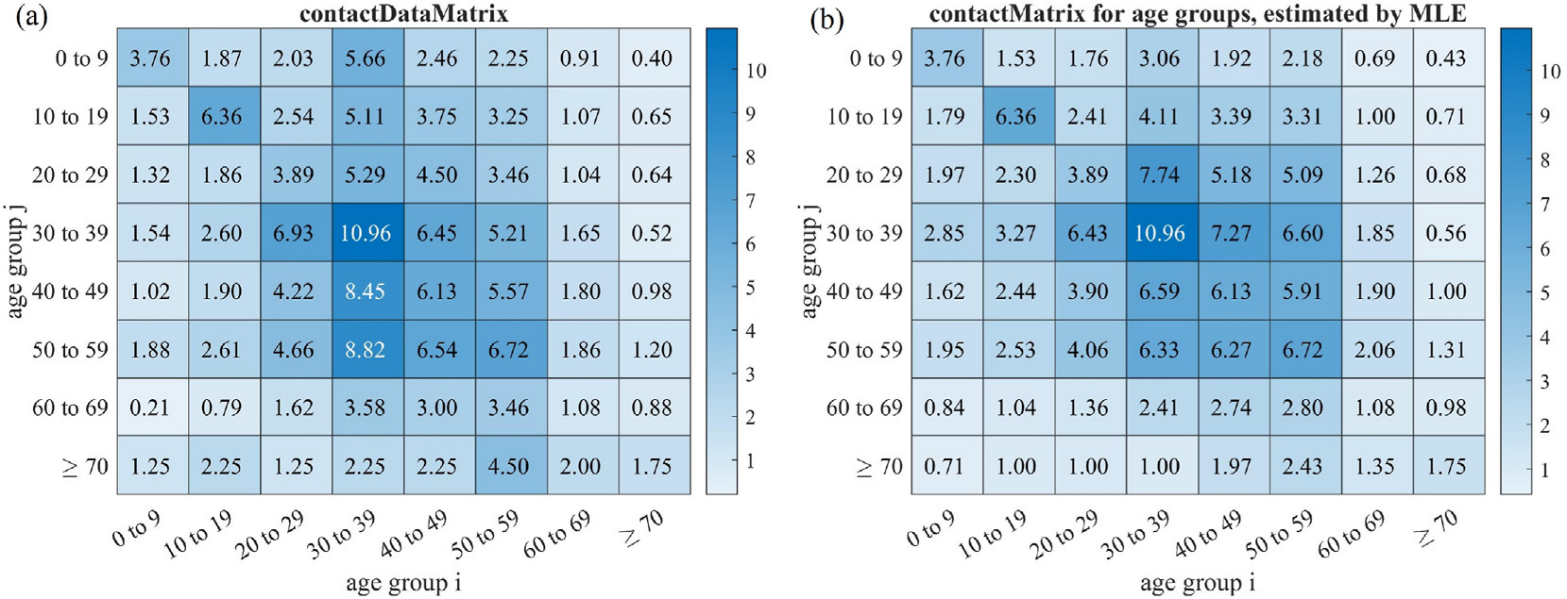
Contact Data Matrix and Contact Matrix. (a) Contact Data Matrix filled with contact data. (b) Contact Matrix estimated by maximum likelihood estimation (based on a bipartite graph model). The model and other estimation of contact matrices are presented in the supplementary material.

The estimated contact matrix in Fig. 2(b) showed a small adjustment compared with the contact data matrix in Fig. 2(a). Both of the matrices showed a concentration of contacts in diagonal entries and middle entries, which indicates people in the same age group are more likely to make contact, and people in middle ages are more likely to contact with others (middle ages are more likely to have children and parents to feed). Our matrices are coherent with the result of the contact matrix in Wuhan and Shanghai, two cities in China (Zhang et al., 2020) (Zhang et al., 2021). The scenario-specific contact matrices are presented in the supplementary material.

### 3.3 Optimal vaccinating process

The optimal vaccinating process is computed for different basic reproduction number $R_{0}=1,2,\cdots ,12$; different objective function of cumulative cases, hospitalization, and fatality; and different parameters of variants Delta and Omicron in reference (COVID-19 Incidence and Death Rates, 2022). We included all those results in the supplementary material, and present only the result of Delta variant with $R_{0}=8$.

**Fig. 3:**
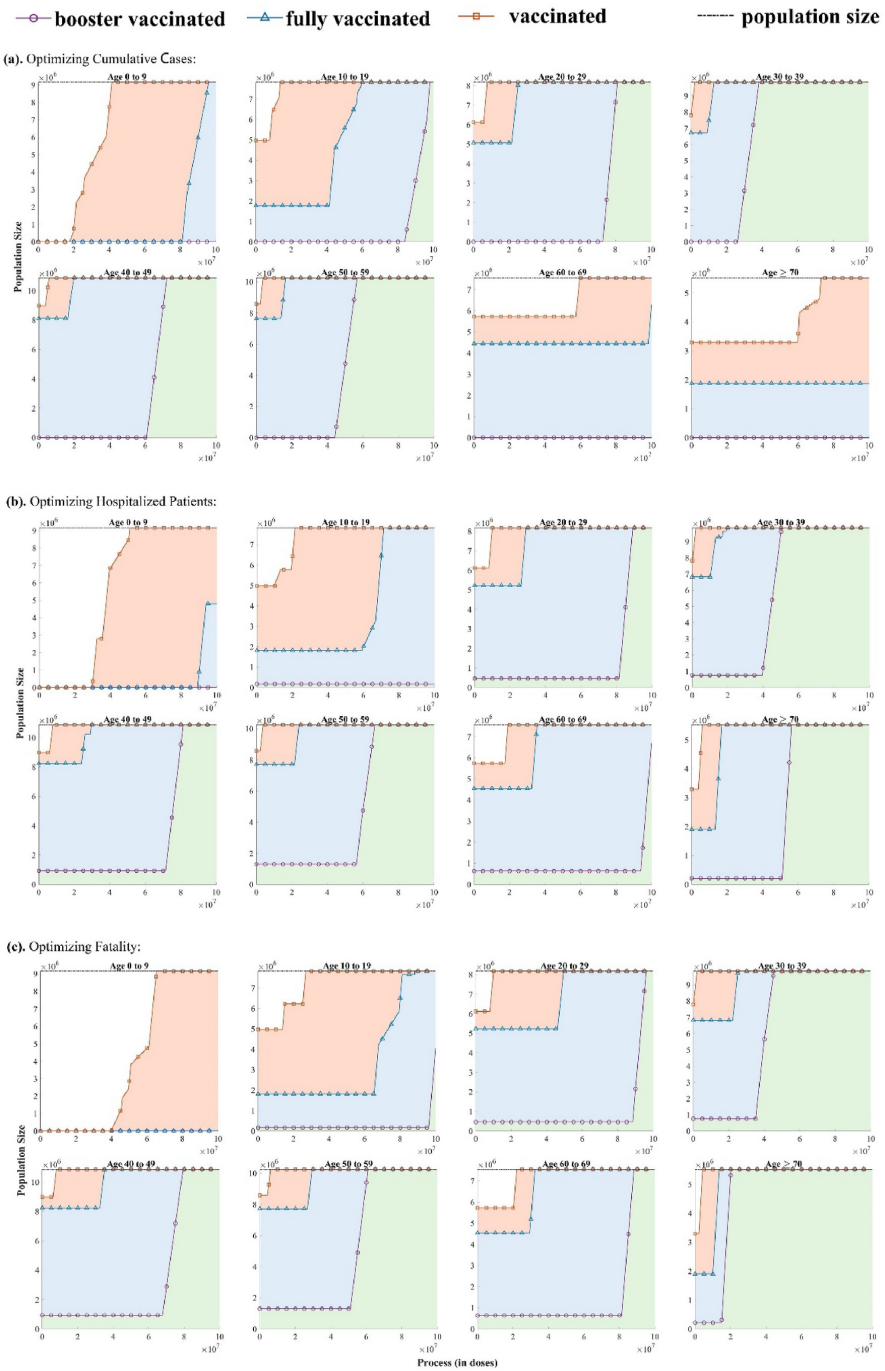
Optimized Vaccinating Strategy Under Current Contact Pattern and Vaccine Coverage. With R_0 = 8 and parameters of Delta variant. (a): minimize cumulative cases; (b): minimize hospitalization; (c): minimize fatality. For each of (a), (b), (c), 8 sub-figures represent the optimal vaccination process in 8 age groups. The x-axis represent the vaccination process in doses; the y-axis represents the population size of four vaccination status. The purple line with circles denote the population size of booster vaccinated; the blue line with triangles denote the population size of at least fully-vaccinated (including fully vaccinated and booster vaccinated); the red line with squares denote the population size of at least vaccinated (including un-fully vaccinated, fully vaccinated, and booster vaccinated). These lines depicts how population size, i.e. the coverage changes with optimized vaccination process. The line increased from the first dose gives the specific information about which population should be vaccinated first.

Fig. 3 answers the question which age-vaccine group should be vaccinated first, and showed how the priority of each age-vaccination group changes with doses, which gives a hint for future vaccine distribution.

In the computed optimal vaccinating process, three objective functions give a coherent result which requires an immediate vaccination to those un-vaccinated in the age group 30 to 39 (Fig. 3(a)). This can be seem from the upper-right sub-figure of age 30 to 39, where the population size of at least vaccinated one dose increased since the first dose (x-axis = 0), which means the un-vaccinated of 30–39 should be firstly vaccinated into the un-fully vaccinated group. Since the age group 30 to 39 is of highest contact frequency with other groups, this strategy could be interpreted as to reduce transmissions at an early stage. The objective of reducing hospitalization and fatality, however, requires vaccination on the oldest just after the age group of 30–39 are all covered with at least one dose for higher $R_{0}$, and interchange order for lower $R_{0}$. This can be interpreted as to reducing cases in high-risk populations when the transmission is somehow controlled.

Despite the age, and focusing only on the vaccination status groups, we found that the un-vaccinated group should be vaccinated first to reduce the cumulative cases, since, in all 8 sub-figures in Fig. 3 (a), the un-vaccinated are always first to be vaccinated.

## 4 Discussion

### 4.1 Validity of close-contact matrix

In simulating the transmission of air-borne disease like SARS-Cov-2, the age-specific contact pattern is typically obtained from a survey of susceptible population (Tran Kiem et al., 1038). However, in this paper, the dataset of close-contact survey from reported cases is firstly used to reflect the contact pattern of cases. This estimation may be more suitable for simulating transmissions if significant proportion of cases behave differ from the susceptible population, and that is the current circumstance with public health and social measures (PHSMs).

Our estimation of contact matrix considers the nature of connection between two sub-groups by using a bipartite graph model. The resulted estimation is robust for lack of cases (participants) in some age groups, i.e. the circumstances that one or more rows of the contact data matrix contains little or even no cases. This method solves the problem by calibrating such rows by its symmetric columns.

The estimated contact matrices are consistent with results from an age-specific contact surveyed by a study (Zhang et al., 2021), suggesting the feasibility of simulating scenarios like sex, age, school, workplace by our estimated setting-specific contact matrices. The concentration of contacts in middle age groups reveals characteristics of social functions in each population.

The disadvantage of case-contact data may be described by its difficulty of collection. Without a comprehensive case-wise survey, it is almost impossible to construct a reliable contact matrix. Also, the number of cases can not be too small, which makes it not always available at the beginning of an outbreak.

### 4.2 The immune barrier

By November 2021, 50% of people has been fully vaccinated in China (Data, 2022). We found a relatively high vaccine coverage with 27.5% of none vaccinated, 13.4% of vaccinated but un-fully, 59.1% of fully vaccinated and very little booster vaccinated in Hunan Province, leading to an immune barrier that contains the outbreak of SARS-CoV-2. The vaccine effectiveness had been increased with the enhancing of vaccination doses, which is similar to reports in study (Mrak et al., 2022).

The immune barrier is of great importance to restrain transmission and reducing social burden of hospitalization and morbidities. In epidemics, new variants, decay of vaccine efficacy lead us a great challenge of maintaining immune barrier of population within a relatively high level. As a new variants become dominant, epidemiologists must check whether the current immune barrier is adequate or not. From the point of view of optimization, we can always compute the optimal vaccination strategy and observe its effectiveness using parameters of new variants, new vaccine efficacy and the decayed equivalent vaccine coverage.

### 4.3 Validity of multi-group transmission model

The multi-group VEFIAR model considers the heterogeneity of age and vaccination status in population, which is a improvement of our previous studies (Zhao et al., 2020). The model is simulated with real-world vaccine coverage and immunity using data from Hunan Province.

The simulation gives highest incidence rate in people aged 30–39 years old, which was consistent with a 27.56% proportion reported cases in this age group. This concentration is similar to a study indicating that transmission mainly occurred in the middle ages and old people (Zhao et al., 2020).

### 4.4 Effectiveness of the optimized vaccination process

The optimized dose-wise vaccination strategy present a time varying decision-making progress, showing the changing priority about which age group and vaccination status group should firstly be vaccinated in further vaccination. Sometimes it may be more instructive than the results of optimized static distribution in (Matrajt et al., 2021b), especially for developing countries with small vaccinating capacity stream. The greedy algorithm we used is simpler, and easier implemented than other studies that transform the optimal control problem of real-time vaccination optimization to nonlinear programming problems (NLPs) (Han et al., 2021). The optimization method we used can be easily extended for decision variables of higher dimensions, considering heterogenity of age-vaccination-region subgroups. Although the optimization method of greedy algorithm can hardly found the global minimum for general optimization problems, its objective of optimize efficiency for each dose meets the real-world's needs of reducing the epidemic cost at any time within and before endemics.

The objective function decrease smoothly under the computed optimal vaccination strategy, which support the stability of the optimization.

In practice, if local contact pattern is obtained, then we can optimize the further vaccination strategy under current immune barrier for many epidemic indices.

### 4.5 Limitations

Vaccine efficacy decays over time, however, such consideration involving assumptions about the speed of local vaccination capacity, and the decay curve of average vaccine efficacy. The decay of individual vaccine efficacy can be introduced into the dynamical model by using the equivalent decay of vaccinated population size in dynamic models. However, we can omitted this for the amazing vaccination speed in Hunan.

Since the contact pattern and odds ratios are estimated using data collected in Hunan Province, the extension of such optimization may be limited to Hunan Province. Regional contact pattern and local vaccine coverage are recommended when extending similar optimization to other regions.

The optimal solution given by greedy algorithm is more likely to be a local optimizer rather than a global optimizer.

In practice, the high-risk occupations should be vaccinated with priority, however, it is not considered in the age-vaccination grouped model.

## 5 Conclusion

The resulting dose-wise vaccination strategy illustrate the changing priority about which age group and vaccination status group should firstly be vaccinated in further vaccination, which may present constructive suggestions. Under the current vaccine coverage and vaccine efficacy, the objective of reducing transmission requires vaccination in age groups of highest contact with other groups, with more priority for un-vaccinated than un-fully or fully vaccinated. However, the objective of reducing total hospitalization and fatality requires not only to reduce of transmission but also to protect the vulnerable population of the olders.

The contact pattern estimated by the case-contact data depicts the behavior of cases rather than the un-infected population, and will be more suitable for transmission simulation, especially for diseases where significant proportion of patients behaves differ from un-infected population. In simulation of emerging infectious diseases, although the data-driven contact pattern is hardly available, the uncertainty of differences between contact pattern that cause actually transmission and the contact pattern of un-infected population in ordinary period must be considered with suitable calibrations.

The optimization method of greedy algorithm is easy to extended for heterogeneity consideration of futher partitioning the whole population into more heterogeneous sub-groups.

## Consent for publication

Not applicable.

## Ethics approval and consent to participate

Not applicable.

## Availability of data and materials

A PDF file contains modeling details and profiles of numerical solutions. All codes and figures are available in author's github: https://github.com/1095912686/SupplementaryCodesForOptimalVaccinationStrategyInHunanForSARS-CoV-2.

## Funding

This study was partly supported by the 10.13039/501100012166National Key Research and Development Program of China (2021YFC2301604), the Research Project on Education and Teaching Reform of Undergraduate Universities of Fujian Province, China (FBJG20210260), the Self-supporting Program of Guangzhou Laboratory (Grant No. SRPG22-007), the 10.13039/100000865Bill & Melinda Gates Foundation (Grant INV-005834 to T.C.), the Research on the Precise Prevention and Control System of SARS-Cov-2 (Grant No. 35022022YJ07, Topic No.2022YJ-3). All funders of this study had no role in study design, data collection, data analysis, data interpretation or writing of the report.

## Author's contributions

XHG conceived and designed the study, conducted the analysis and the framework of the study, wrote the first draft of the manuscript, wrote MATLAB codes. ZYL provided original data, wrote the first draft of the manuscript, improved this research and edited the manuscript. STY conducted the analysis and the framework of the study, wrote the first draft of the manuscript. ZYZ conceived and designed the study, conducted the analysis and the framework of the study, wrote the first draft of the manuscript, improved this research and edited the manuscript. YCG wrote the first draft of the manuscript, improved this research and edited the manuscript. GA wrote the first draft of the manuscript, improved this research and edited the manuscript. SLZ provided original data, wrote the first draft of the manuscript, improved this research and edited the manuscript. GZ provided original data, wrote the first draft of the manuscript, improved this research and edited the manuscript. SXH conceived and designed the study, provided original data, wrote the first draft of the manuscript, improved this research and edited the manuscript. KWL provided original data, improved this research and edited the manuscript. TMC conceived and designed the study, improved this research and edited the manuscript.

All authors contributed to revising subsequent versions of the manuscript. All authors read and approved the final manuscript.

## Declaration of competing interest

The authors declare that they have no known competing financial interests or personal relationships that could have appeared to influence the work reported in this paper.
