# Supplementary material for: Contact pattern, current immune barrier, and pathogen virulence determines the optimal strategy of further vaccination
Source: Infect Dis Model. 2023 Jan 13;8(1):192–202. doi: 10.1016/j.idm.2023.01.003 (PMC9836995; doi:10.1016/j.idm.2023.01.003)
Supplement: Multimedia component 1 [file mmc1.pdf]

Contents

1 Contact Matrices **3**

1.1 Construction of the Contact Matrix . . . 3

1.2 Graph Model of the Contact Matrix . . . 3

1.3 Construction of the Contact Data Matrix . . . 5

1.4 Estimations of Contact Matrix . . . 5

1.4.1 Least Square Estimation of the Contact Matrix . . . 5

1.4.2 Weighted Least Square Estimation of the Contact Matrix . . . 6

1.4.3 Maximum Likelihood Estimation of the Contact Matrix . . . 7

1.5 Estimated Contact Matrices . . . 8

2 An Introduction to The Multi-Group $SEIAR$ Model **10**

2.1 Formulation of The Multi-Group $SEIAR$ Model . . . 11

2.2 Formulation of The Newly Infection Term . . . 12

2.3 Feasibility of The Grouped Models——$SEIAR$ as an Example . . . 14

2.4 Solve q From R_0 . . . 14

2.5 Profiles of Simulation . . . 15

3 Extend $SEIAR$ to $VEFIAR$ by Considering Heterogeneity **15**

3.1 Flowchart of The Multi-Group $VEFIAR$ Model . . . 15

3.2 Parameters of The Multi-Group $VEFIAR$ Model . . . 17

3.3 Vaccine Coverage . . . 20

3.4 Vaccine Efficacy . . . 22

3.4.1 Single-Variable Analysis of Total Attact Rate (TAR) . . . 22

3.4.2 Multi-Variable Analysis of Odds Ratio (OR) . . . 23

3.5 Solve q From R_0 . . . 26

3.5.1 Step 1 . . . 26

3.5.2 Step 2 . . . 26

3.5.3 Step 3 . . . 27

3.5.4 Step 4 . . . 27

3.5.5 Step 5 . . . 28

3.5.6 Step 6 . . . 28

3.5.7 Step 7 28
3.6 Simulation of The Multi-Group $VEFIAR$ Model 29

4 Optimization of Time-Varying Vaccination **33**
4.1 The Optimization Method 33
4.2 Directional Derivatives of All Possible Update 35
4.3 Greedy Algorithm for Optimization of Vaccinate Process 36
4.3.1 Pseudo-Code 36
4.4 Optimization Profiles 36
4.5 Optimized Results 37
4.5.1 Cumulative Cases for Delta Variant 38
4.5.2 Hospitalization for Delta Variant 51
4.5.3 Fatality for Delta Variant 64
4.5.4 Cumulative Cases for Omicron Variant 77
4.5.5 Hospitalization for Omicron Variant 90
4.5.6 Fatality for Omicron Variant 103

5 Contact Data Decomposition **115**
5.1 NPI Simulations 120

Supplementary Material

October 13, 2022

1 Contact Matrices

1.1 Construction of the Contact Matrix

Definition:(Contact Data Matrix)

The contact data matrix A is a square matrix with its ij-th entry a_{ij} denotes the daily average number of contacts in group j that one case in group i produces.

Definition:(Contact Matrix)

The contact matrix C is a square matrix with its ij-th entry c_{ij} denotes the average number of daily contacts in group j produced by a individual in group i.

1.2 Graph Model of the Contact Matrix

Figure S1 illustrates the relation between c_{ij} and c_{ji}. We consider a bipartite graph of group i and j, by omitting their inner edges and edges connect other groups. In group i, there are N_i individuals denoted by vertices $V_1, V_2, \cdots, V_{N_i}$; and for group j, the N_j individuals are denoted by vertices $W_1, W_2, \cdots, W_{N_j}$. Each contact data pair is denoted by an edge connect group i and group j.

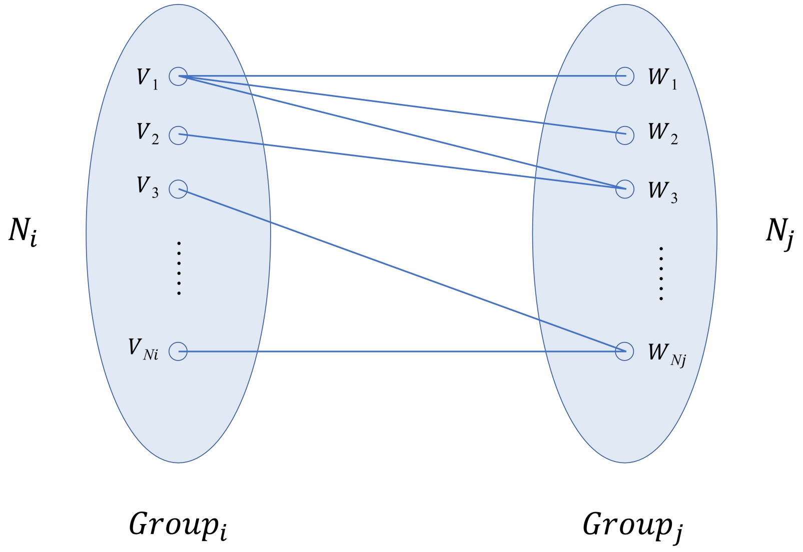

Figure. S1: Bipartite Graph Model

By definition of contact matrix, c_{ij} is the average degree of vertices in group i (in this bipartite graph):

$$c_{ij} = d_i \overset{def}{=} \frac{1}{N_i} \sum_{k=1}^{N_i} deg(V_k) \tag{1}$$

$$c_{ji} = d_j \overset{def}{=} \frac{1}{N_j} \sum_{k=1}^{N_j} deg(W_k) \tag{2}$$

where $deg()$ is the the degree of a vertex, i.e. the number of edges attached to the vertex.

In order to establish the relation between c_{ij} and c_{ji}, it is naturally to consider the conservative quantity in this system. It is found that for these two groups of vertices, the total number of edges between them is constant, no matter how the connective pattern changes. Therefore:

$$N_i d_i = N_j d_j \tag{3}$$

That is,

$$d_j = \frac{N_i}{N_j} d_i \tag{4}$$

i.e.

$$c_{ji} = \frac{N_i}{N_j} c_{ij} \tag{5}$$

1.3 Construction of the Contact Data Matrix

In construction of contact matrix C, the contact data matrix A is firstly filled using (case, contact) data pairs——after initialized $A = 0$, we locate the age group for each case, then add its close contacts to the corresponding row of A. After all data pairs are filled, we divide each row of A by the total number of cases belongs to this group (of averagely one individual), and then divide a 4 day period of the close contact survey.

Matrix A reflects the contact pattern of the (caseAge, contactAge) pairs. However, it may has significant noise due small sample size of incident cases. Therefore, a correction for A is needed to approximate the real contact matrix C.

The following are data matrix A known, and the contact matrix C with $\frac{n(n+1)}{2}$ parameters to be fitted:

$$A = \begin{array}{|c|c|c|c|c|c|c|} \hline a_{11} & a_{12} & a_{13} & \cdots & \cdots & \cdots & a_{1n} \\ \hline a_{21} & a_{22} & a_{23} & \cdots & \cdots & \cdots & a_{2n} \\ \hline a_{31} & a_{32} & a_{33} & \cdots & \cdots & \cdots & a_{3n} \\ \hline \vdots & \vdots & \vdots & \ddots & & & \vdots \\ \hline \vdots & \vdots & \vdots & & \cdots & & \vdots \\ \hline \vdots & \vdots & \vdots & & & \ddots & \vdots \\ \hline a_{n1} & a_{12} & a_{13} & \cdots & \cdots & \cdots & a_{nn} \\ \hline \end{array} \qquad C = \begin{array}{|c|c|c|c|c|c|c|} \hline c_{11} & c_{12} & c_{13} & \cdots & \cdots & \cdots & c_{1n} \\ \hline \frac{N_1}{N_2}c_{12} & c_{22} & c_{23} & \cdots & \cdots & \cdots & c_{2n} \\ \hline \frac{N_1}{N_3}c_{13} & \frac{N_2}{N_3}c_{23} & c_{33} & \cdots & \cdots & \cdots & c_{3n} \\ \hline \vdots & \vdots & \vdots & \ddots & & & \vdots \\ \hline \vdots & \vdots & \vdots & & \cdots & & \vdots \\ \hline \vdots & \vdots & \vdots & & & \ddots & \vdots \\ \hline \frac{N_1}{N_n}c_{1n} & \frac{N_2}{N_n}c_{2n} & \frac{N_3}{N_n}c_{3n} & \cdots & \cdots & \cdots & c_{nn} \\ \hline \end{array}$$

1.4 Estimations of Contact Matrix

Here we use one of the three following methods to estimate the contact matrix C from contact data matrix A.

- least square estimation
- weighted least square estimation
- maximum likelihood estimation

1.4.1 Least Square Estimation of the Contact Matrix

The simplest estimation of those $c_{ij}, \quad i \leq j, \quad i,j = 1,2,\cdots,n$ is the least square fitting described by the following optimization problem:

$$\underset{\forall c_{ij},\ i \leq j}{minimize} \|A - C\|_F \tag{6}$$

where $\| \cdot \|_F$ denotes the Frobenius norm of matrix.

The optimization problem 6 can be reduced to sub-problems to divide and conquer:

$$\hat{c}_{ij} = \underset{c_{ij}}{argmin} \;\; (c_{ij} - a_{ij})^2 + [(\frac{N_i}{N_j})c_{ij} - a_{ji}]^2 \tag{7}$$

$$= \underset{c_{ij}}{argmin} \;\; (1 + \frac{N_i^2}{N_j^2})c_{ij}^2 - (2a_{ij} + 2\frac{N_i}{N_j}a_{ji})c_{ij} + (a_{ij}^2 + a_{ji}^2) \tag{8}$$

$$= \frac{a_{ij} + \frac{N_i}{N_j}a_{ji}}{1 + \frac{N_i^2}{N_j^2}}, \quad \forall ij, \; i < j \tag{9}$$

$$\tag{10}$$

$$\hat{c}_{ji} = \frac{N_i}{N_j}\hat{c}_{ij}, \quad \forall ij, \; i < j \tag{11}$$

$$\tag{12}$$

$$\hat{c}_{ii} = \underset{c_{ii}}{argmin} \;\; (c_{ii} - a_{ii})^2 \tag{13}$$

$$= a_{ii}, \quad i = 1, 2, \cdots, n \tag{14}$$

We can see that the contact matrix C and its estimation are both symmetric if the population of groups are equal, i.e. $N_i = N_j$.

1.4.2 Weighted Least Square Estimation of the Contact Matrix

The unbalanced sample size of cases in each groups requires different importance in errors of each entry of C. Therefore, we weight the errors of each entries by the sample size, i.e. number of cases in each groups, which leads

the following adjusted optimization problems:

$$\hat{c}_{ij} = \underset{c_{ij}}{argmin} \;\; K_i(c_{ij} - a_{ij})^2 + K_j[(\frac{N_i}{N_j})c_{ij} - a_{ji}]^2 \tag{15}$$

$$= \underset{c_{ij}}{argmin} \;\; (K_i + \frac{N_i^2}{N_j^2}K_j)c_{ij}^2 - (2K_i a_{ij} + 2K_j\frac{N_i}{N_j}a_{ji})c_{ij} + (K_i a_{ij}^2 + K_j a_{ji}^2) \tag{16}$$

$$= \frac{K_i a_{ij} + K_j \frac{N_i}{N_j} a_{ji}}{K_i^2 + \frac{N_i^2}{N_j^2}K_j^2} \tag{17}$$

$$\tag{18}$$

$$\hat{c}_{ji} = \frac{N_i}{N_j}\hat{c}_{ij} \tag{19}$$

$$\tag{20}$$

$$\hat{c}_{ii} = \underset{c_{ii}}{argmin} \;\; (c_{ii} - a_{ii})^2 \tag{21}$$

$$= a_{ii} \tag{22}$$

where K_i and K_j denote the number of cases in group i and j.

1.4.3 Maximum Likelihood Estimation of the Contact Matrix

Since the Poisson distribution is frequently used to describe phenomena that have few positive outcomes over many repeating trials, we assume that:

$$c_{ij} \sim Poisson(\lambda) \tag{23}$$

$$c_{ji} \sim Poisson(\frac{N_i}{N_j}\lambda) \tag{24}$$

Let $b_{ij}(k)$ denotes the daily number of contact in group j produced by k-th observed case in group i .

Then the data set for training c_{ij} can be represented as:

$$b_{ij}(k), \;\; k = 1, 2, \cdots, K_i \tag{25}$$

$$b_{ji}(k), \;\; k = 1, 2, \cdots, K_j \tag{26}$$

where K_i is the total number of cases lies in group i, K_j is the total number of cases lies in group j.

The likelihood function:

$$Likelihood(\lambda) = (\prod_{k=1}^{K_i} \frac{\lambda^{b_{ij}(k)}e^{-\lambda}}{b_{ij}(k)!})(\prod_{k=1}^{K_j} \frac{(\frac{N_i}{N_j}\lambda)^{b_{ji}(k)}e^{-\frac{N_i}{N_j}\lambda}}{b_{ji}(k)!}) \tag{27}$$

The log likelihood function:

$$LogLikelihood(\lambda) \propto \sum_{k=1}^{K_i} log(\lambda^{b_{ij}(k)} e^{-\lambda}) + \sum_{k=1}^{K_j} log((\frac{N_i}{N_j}\lambda)^{b_{ji}(k)} e^{-\frac{N_i}{N_j}\lambda}) \tag{28}$$

$$= \sum_{k=1}^{K_i} log(\lambda^{b_{ij}(k)}) - K_i\lambda + \sum_{k=1}^{K_j} log((\frac{N_i}{N_j}\lambda)^{b_{ji}(k)}) - \frac{N_i}{N_j}K_j\lambda \tag{29}$$

$$= \sum_{k=1}^{K_i} log(\lambda^{b_{ij}(k)}) + \sum_{k=1}^{K_j} log((\lambda)^{b_{ji}(k)}) - (K_i + \frac{N_i}{N_j}K_j)\lambda + \sum_{k=1}^{K} log((\frac{N_i}{N_j})^{b_{ji}(k)}) \tag{30}$$

$$\stackrel{def}{=} LL(\lambda) \tag{31}$$

Taking derivative of $LL(\lambda)$:

$$\frac{d}{d\lambda}LL(\lambda) = \sum_{k=1}^{K_i} \frac{(b_{ij}(k))\lambda^{b_{ij}(k)-1}}{\lambda^{b_{ij}(k)}} + \sum_{k=1}^{K_j} \frac{(b_{ji}(k))\lambda^{b_{ji}(k)-1}}{\lambda^{b_{ji}(k)}} - (K_i + \frac{N_i}{N_j}K_j) \tag{32}$$

$$= \sum_{k=1}^{K_i} \frac{b_{ij}(k)}{\lambda} + \sum_{k=1}^{K_j} \frac{b_{ji}(k)}{\lambda} - (K_i + \frac{N_i}{N_j}K_j) \tag{33}$$

$$= \frac{1}{\lambda}(\sum_{k=1}^{K_i} b_{ij}(k) + \sum_{k=1}^{K_j} b_{ji}(k)) - (K_i + \frac{N_i}{N_j}K_j) \tag{34}$$

The maximum likelihood estimation of λ (λ that maximizing $LL(\lambda)$) is obtained by letting $\frac{d}{d\lambda}LL(\lambda) = 0$:

$$\hat{\lambda} = \frac{\sum_{k=1}^{K_i} b_{ij}(k) + \sum_{k=1}^{K_j} b_{ji}(k)}{K_i + \frac{N_i}{N_j}K_j} \tag{35}$$

Noticed that $a_{ij} = \frac{1}{K_i}\sum_{k=1}^{K_i} b_{ij}(k)$, we have,

$$\hat{c}_{ij} = \hat{\lambda} \tag{36}$$

$$= \frac{K_i a_{ij} + K_j a_{ji}}{K_i + \frac{N_i}{N_j}K_j} \tag{37}$$

$$\hat{c}_{ji} = \frac{N_i}{N_j}\hat{c}_{ij} \tag{38}$$

$$\tag{39}$$

1.5 Estimated Contact Matrices

Three proposed methods (LSE, WLSE, MLE) are adopted to the data set. The contact data matrix A and estimated contact matrices C are shown as follows (Figure S2). Further more, by using the MLE, the 95% confidential intervals for all entries of contact matrix are given in Figure S3.

We also split the data set by the diagnosed date of cases at August 4, 00:00 (the peak of daily new cases), and estimating the contact matrices C_1, C_2 (contact matrix before and after the peak, see figure S4) using these

two data set. The entries of C_1, C_2 shows a strong linear correlation, therefore, we may use a entry-wise linear interpolation of C_1 and C_2 to estimate the value of entries at a given time instance. There is an expected reduction of maximum eigenvalues of C_1 and C_2: from $\lambda_{max}(C_1) = 34.599$ to $\lambda_{max}(C_2) = 25.404$.

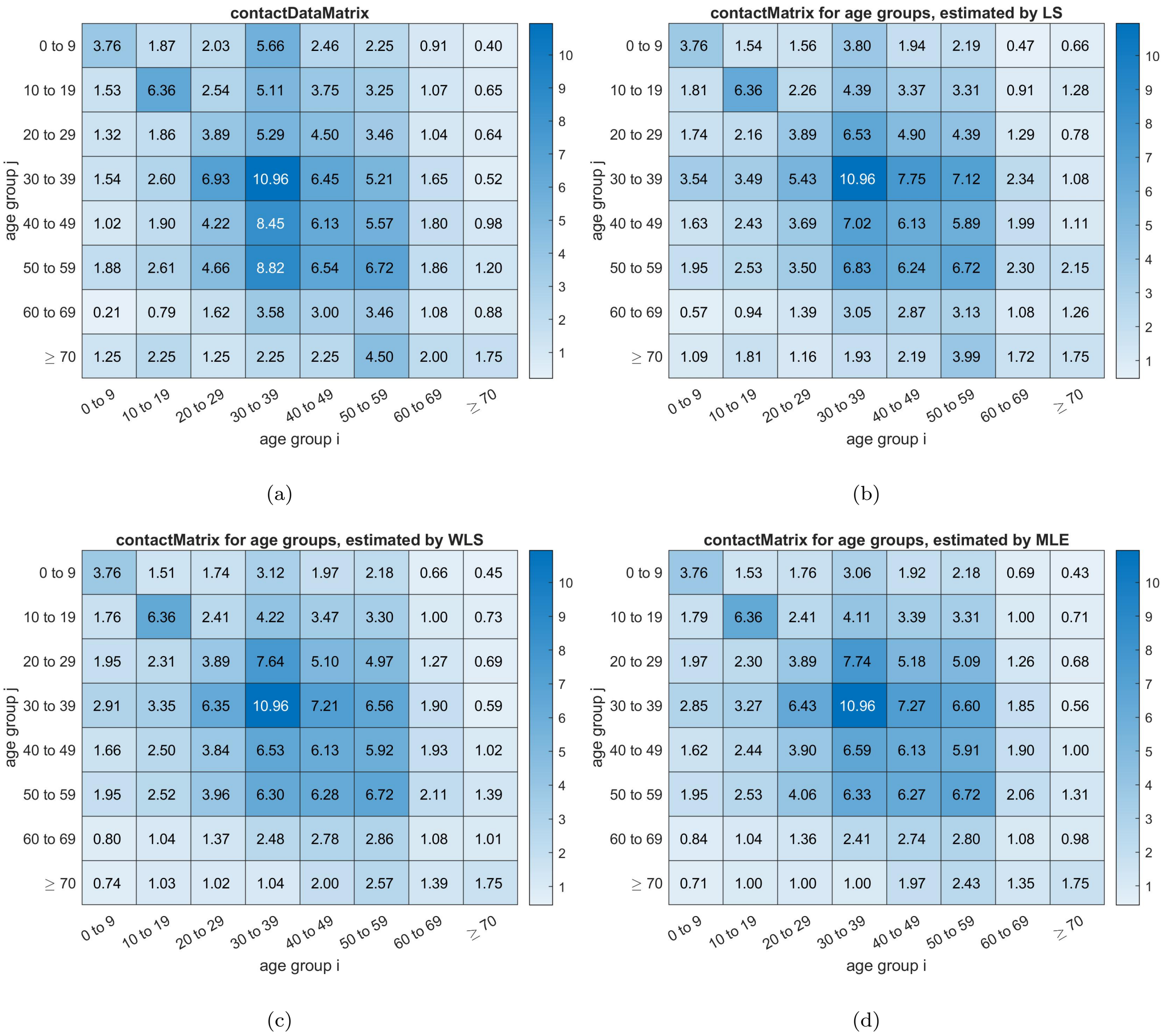

Figure. S2: Contact Data Matrix and Contact Matrices Estimated by different Methods: (a): The contact data matrix for age groups, filled with the case-contact data pairs, with maximum eigenvalue 28.861; (b): The contact matrix for age groups, estimated by least square estimation, with maximum eigenvalue 29.358; (c): The contact matrix for age groups, estimated by weighted least square estimation, with maximum eigenvalue 28.915; (d): The contact matrix for age groups, estimated by maximum likelihood estimation, with maximum eigenvalue 28.951.

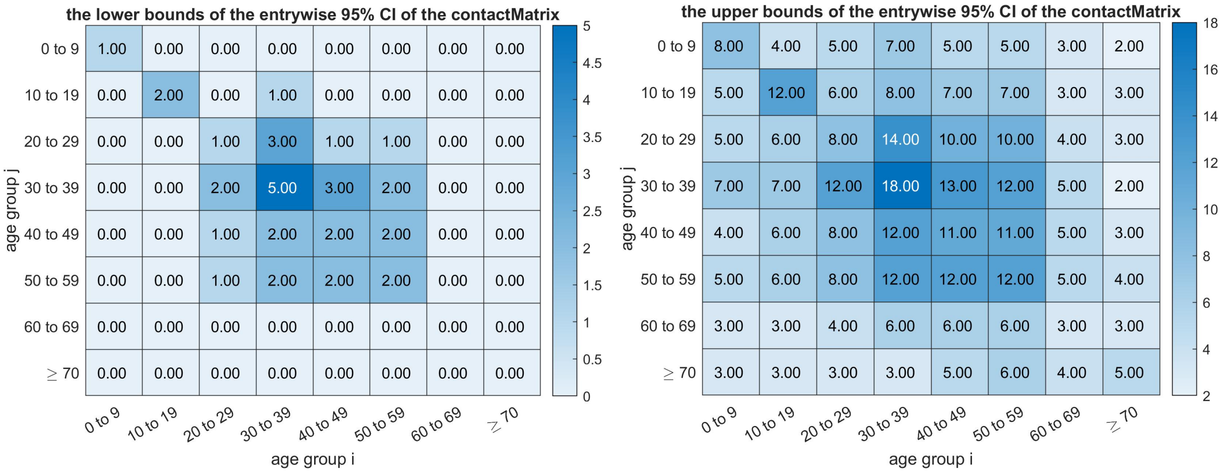

(a) (b)

Figure. S3: The entrywise estimation of 95% confidential interval of contact matrix, (a): the lower bound, with maximum eigenvalue 8.7263, (b): the upper bound, with maximum eigenvalue 57.642

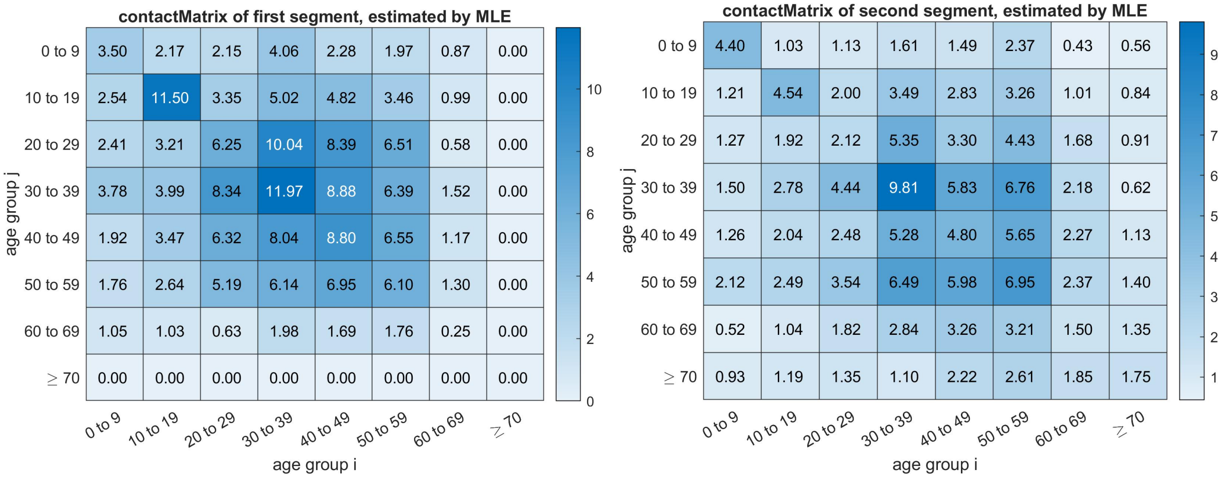

(a) (b)

Figure. S4: The estimated contact matrix before and after the peak of daily new incidents, (a): before the peak, with its eigenvalue equals to 34.599 person per day; (b): after the peak, with its eigenvalue equals to 25.404 person per day

2 An Introduction to The Multi-Group $SEIAR$ Model

The multi-group model considers the heterogeneity of populations by partitioning the all compartments (variables) in classic SIR, $SEIR$, $SEIAR$ models into several sub-groups.

In this study, we extended the multi-group $SEIAR$ model into a multi-group $VEFIAQR$ model by inspect the natural history properties of Covid-19.

For the better understanding and stability of our model, we shall start with the introduction of the multi-group $SEIAR$ model, and extend most of model structures parallelly to the multi-group $VEFIAR$ model in next section.

2.1 Formulation of The Multi-Group $SEIAR$ Model

The multi-group $SEIAR$ model is a deterministic dynamic model based two assumptions: (1) the homogeneity of populations inside each compartment, (2) populations of groups are fully mixed (inside and between them), and described by the following ordinary differential equations (ODEs):

$$\begin{aligned}
\frac{dS_i}{dt} &= br_iN - S_i\sum_{j=1}^{n}\beta_{ji}\left(I_j + \kappa A_j\right) - dr_iS_i \\
\frac{dE_i}{dt} &= S_i\sum_{j=1}^{n}\beta_{ji}\left(I_j + \kappa A_j\right) - p_i\omega_i'E_i - \left(1-p_i\right)\omega_iE_i - dr_iE_i \\
\frac{dI_i}{dt} &= \left(1-p_i\right)\omega_iE_i - \left(dr_i + f_i + \gamma_i\right)I_i \\
\frac{dA_i}{dt} &= p_i{\omega_i}'E_i - \left(dr_i + \gamma_i'\right)A_i \\
\frac{dR_i}{dt} &= \gamma_iI_i + \gamma_i'A_i - dr_iR_i \\
i &= 1, 2, \cdots, n
\end{aligned} \tag{40}$$

where

- subscript i denotes the variable or parameter is specified to the i-th group
- n denotes the total number of groups
- S_i denotes the number of susceptible population in group i
- E_i denotes the number of exposed population (i.e, infected but not infectious) population in group i
- I_i denotes the number of symptomatic infectious population in group i
- A_i denotes the number of asymptomatic infectious population in group i
- R_i denotes the number of fully immunized (i.e. impossible to be infected) population in group i
- $N_i = S_i + E_i + I_i + A_i + R_i$ is the population size of group i
- $N = N_1 + N_2 + \cdots + N_n$ is the total population size

- br_i denotes the age specific birth rate (for age-grouped model, only one br_i of the youngest age group is nonzero)

- dr_i denotes the mortality rate of group i

- β_{ij} is the coefficients describing the daily transmission rate from group i to group j

- κ is the relatively of transmission ability of asymptomatic cases compared with the symptomatic cases, it is assumed to be group irrelevant

- f_i is the case fatality rate of group i

- p_i is the probability that one infected individual in group i will developed into a asymptomatic case

- ω_i is inverse of the average latent period of the symptomatic population in the i-th group, it is used to quantify the remove rate of compartment E_i. The inverse of incubation period is usually adopted for practice

- $\omega_i^{'}$ is the inverse of the average latent period of the asymptomatic population in the i-th group

- γ_i is the inverse of average infectious period for symptomatic cases

- $\gamma_i^{'}$ is the inverse of average infectious period for asymptomatic cases

The variables S_i, E_i, I_i, A_i, R_i are also called as compartments.

2.2 Formulation of The Newly Infection Term

The widely used newly infection in group i, $S_i \sum_{j=1}^{n} \beta_{ji} \left(I_j + \kappa A_j\right)$, is based on the assumption that the newly infections is proportional to the number of susceptible individuals and the number of infectious individuals, therefore, the constants β_{ji} are introduced as coefficients of such proportion.
(Note: if the change of population size N_i is neglectable, then the bi-linear $S_i \sum_{j=1}^{n} \beta_{ji} \left(I_j + \kappa A_j\right)$ and $S_i \sum_{j=1}^{n} \beta_{ji} \frac{I_j+\kappa A_j}{N_j}$ makes no difference.)

To introduce the impact of contact patterns in the grouped SEIAR model, the transmission rate coefficients β_{ij} are formularized as the product of three part:

- daily average number of contact in group j that a single individual in group i will produce

- the probability of infection via a single contact

- the relatively susceptibility of group i

that is,

$$\beta_{ji} N_j = c_{ij} q \sigma_i \tag{41}$$

where c_{ij} is the ij-th entry of contact matrix; q is the probability of infection after a single contact; σ_j is the susceptibility of group j.

With this decomposition of β_{ij}, the newly infection term $S_i \sum_{j=1}^{n} \beta_{ji} (I_j + \kappa A_j)$ is formulated as:

$$S_i \sum_{j=1}^{n} c_{ij} q \sigma_i \frac{I_j + \kappa A_j}{N_j} \tag{42}$$

This formulation of newly infections can be interpreted as follows.

- At the beginning of an outbreak, the infected take only a small proportion in population, which means a arbitrarily picked individual x is more likely to be susceptible (rather than infected).
- Hence, we shall consider how many infectious individuals that a susceptible may contact, instead of the number of susceptibles that an infectious individual will contact (the infected are saturated in susceptibles in this time period).
- Let x_i denotes a susceptible individual in group i. Among all $\sum_{j=1}^{n} c_{ij}$ individuals x_i had contacted (during a time duration of one day), there will be averagely $\sum_{j=1}^{n} c_{ij} \frac{I_j + \kappa A_j}{N_j}$ infectious individuals (multiplies by the proportion of infectious $\frac{I_j + \kappa A_j}{N_j}$, where κ can be viewed as an convert coefficients that regard all A_j as I_j by a coefficient κ). (Note that $\sum_{j=1}^{n} c_{ij} \frac{I_j + \kappa A_j}{N_j}$ is very small quantity, but it still showed significant results by summing up all susceptible individuals x_i).
- Then, product by the probability q of infection via a single contact to infectious, and summing up all S_i susceptible individuals, we have the expected number of infections in group i:

$$q S_i \sum_{j=1}^{n} c_{ij} \frac{I_j + \kappa A_j}{N_j} \tag{43}$$

- If we want to consider the different susceptibilities in different groups, then a parameter of relative susceptibility σ_i could be introduced for each group:

$$q \sigma_i S_i \sum_{j=1}^{n} c_{ij} \frac{I_j + \kappa A_j}{N_j} \tag{44}$$

  Note that σ_i is a relative quantity, and it should has a magnitude of $O(1)$.

2.3 Feasibility of The Grouped Models——$SEIAR$ as an Example

What would happened if perform both the grouped and un-grouped model on the same scenario without considering the heterogeneity of different groups? We can see from the following lemma that the result would be equivalent, which ensures the validity of group model.

Lemma 1: (feasibility of grouped $SEIAR$ model)

The grouped $SEIAR$ model is equivalent to the un-grouped $SEIAR$ model, if all parameters except parameter in newly infections terms are group irrelevant.

Proof:

In equation 40, we denote $S = S_1 + S_2 + \cdots + S_n$ and similar for E, I, A, R. If all parameters except β_{ij} are group irrelevant, then by summing up all equations of $\frac{dS_i}{dt}$, we have:

$$\frac{dS}{dt} = b_r N - \sum_{i=1}^{n}\sum_{j=1}^{n}\beta_{ji}(I_j + \kappa A_j)S_i - d_r S \tag{45}$$

$$= b_r N - \sum_{i=1}^{n}\sum_{j=1}^{n}\frac{c_{ij}q\sigma}{N_j}(I_j + \kappa A_j)S_i - d_r S \tag{46}$$

Noticed that if the population is homogeneity, full-mixed and groups makes no difference, then c_{ij}, the average number of contact in group j produced by one individual in group i, is proportional to the population size in group j. That is, c_{ij}/N_j is a constant. We denote this constant by c, and we have:

$$\frac{dS}{dt} = b_r N - \sum_{i=1}^{n}\sum_{j=1}^{n} q\sigma(I_j + \kappa A_j)S_i - d_r S \tag{47}$$

$$= b_r N - cq\sigma(I + \kappa A)S - d_r S \tag{48}$$

We can see that the equation of entire $S = S_1 + S_2 + \cdots + S_n$ is mutually equivalent to the equation of un-grouped model (where $\beta = cq\sigma$). Similarly, the equation for E, I, A, R of group and un-grouped model are mutually equivalent.

Lemma 1 ensures the coherence of the grouped model, that is, in the simplest case, grouped $SEIAR$ model is degenerated to the un-grouped $SEIAR$ model.

2.4 Solve q From R_0

The probability q of infection via a single contact is a crucial parameter, that can be solved from given R_0 under current contact matrix C and other parameters in ODEs.

By using the next generation method, the interactive R_{ij} (expectation of secondary infections in group j that one infected individual in group i will produce during its life span as infectious) is computed as:

$$R_{ij} = \frac{\beta_{ij} N_j}{d_{r_j} + p_j \omega_j' + (1 - p_j)\omega_j} \left[\frac{\kappa_j p_j \omega_j'}{\left(\gamma_j' + d_{r_j}\right)} + \frac{(1 - p_j)\omega_j}{\left(\gamma_j + d_{r_j} + f_j\right)} \right] \tag{49}$$

If parameters d_{r_j}, p_j, $\omega_j^{'}$, γ_j, $\gamma_j^{'}$, ω_j are group-irrelevant, then the basic reproduction number R_0 of whole population can be expressed as:

$$R_0 = \lambda_{max}(\begin{bmatrix} R_{11} & R_{12} & \cdots & R_{1n} \\ R_{21} & R_{22} & \cdots & R_{2n} \\ \vdots & \vdots & \ddots & \vdots \\ R_{n1} & R_{n2} & \cdots & R_{nn} \end{bmatrix}) \tag{50}$$

where $\lambda_{\max}(\cdot)$ denotes the leading eigenvalue (with maximum real part) of a matrix.

By substituting $\beta_{ij} = c_{ji} q \sigma_j / N_i$, equation 50 is indeed a linear equation with one unknown q, and can be easily solved by program.

2.5 Profiles of Simulation

Once the probability of infection q is solved form the expression of R_0, the coefficients of transmission β_{ij} can be easily determined by $\beta_{ij} = c_{ij} q \sigma_i / N_i$. Therefore, we have all parameter needed for simulation (i.e. solve the ODE with given initial point).

One may define a series basic reproduction number $R_0 = [1, 2, 3, \cdots, 8]$ for simulating.

3 Extend $SEIAR$ to $VEFIAR$ by Considering Heterogeneity

This section, we extend the classic multi-group $SEIAR$ model to a $VEFIAR$ model by considering the vaccination effects and the natural history of Covid-19.

3.1 Flowchart of The Multi-Group $VEFIAR$ Model

Due to the effectiveness of the social distancing policy, there are only small-scale outbreaks scattering as the vaccinating campaign proceeds steadily. Therefore, for considering a short term outbreak, we might omit the vaccinate progress and treat the vaccine converge rate as constant in each age groups.

Such simplification is natural, however, makes it possible to consider the efficiency of vaccine without introducing extra compartments in the grouped model or adopting partial differential equations.

For the significant proportion of infectious but not yet symptomatic cases in Covid-19 patients, a pre-symptomatic compartment F is introduced between E and A for each groups.

The flowchart of the $VEFIAR$ model is showed in Figure S5. Noticed that there is a bijective mapping (a one-to-one correspondence) between the flowchart and the ODEs.

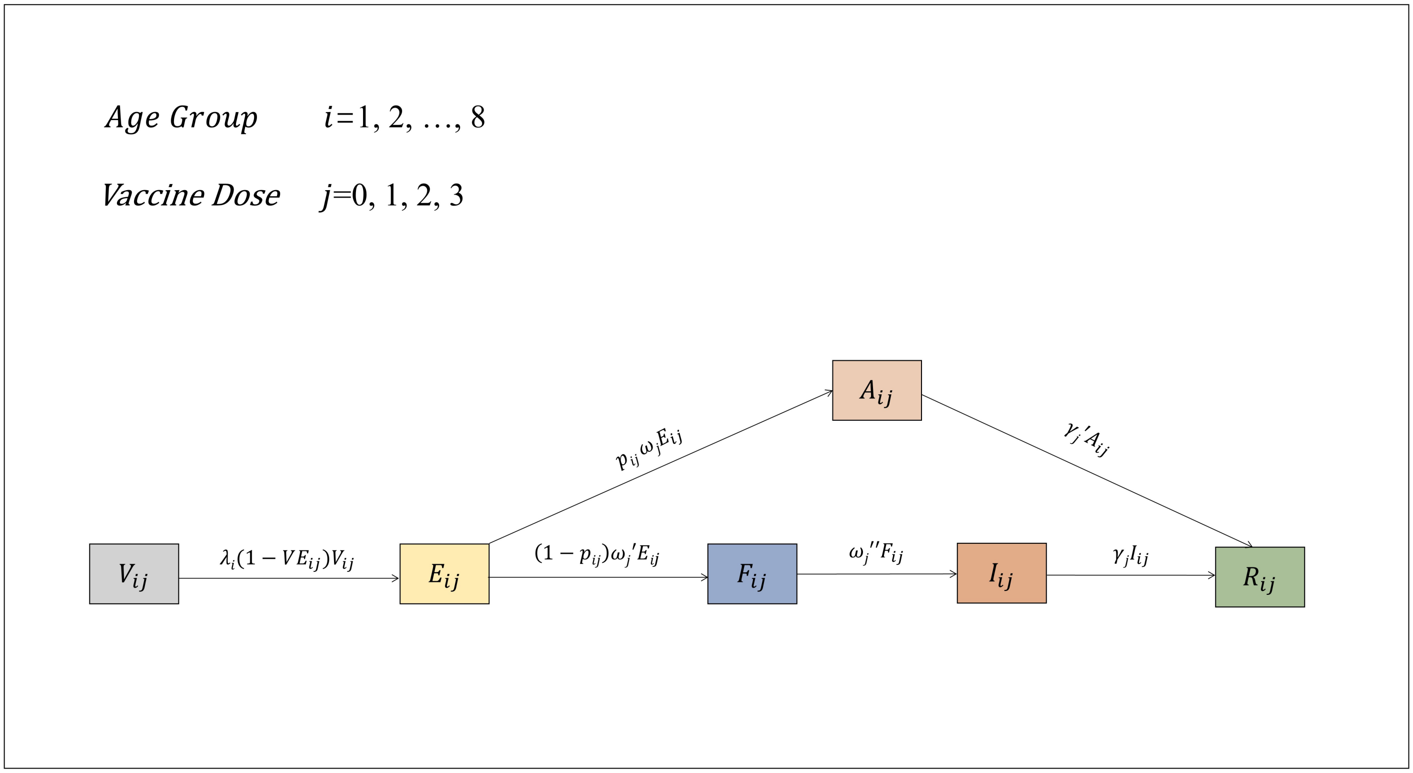

Figure. S5: Flowchart of The VEFIAR Model. There are 8 age groups, with each age group partitioned into 4 vaccine groups (0, 1, 2, 3 represent none-vaccinated, un-fully vaccinated, fully vaccinated, booster vaccinated). Thus there are $8 \times 4 \times 6 = 192$ compartments in all. Variables V_{ij}, E_{ij}, F_{ij}, I_{ij}, A_{ij}, R_{ij} represent susceptible, exposed, pre-symptomatic, symptomatic, asymptomatic, removed population in group ij respectively. Profiles of parameters are presented in Table S2.

The ordinary differential equations of the model is given by:

$$\frac{dV_{ij}}{dt} =-\lambda_i(1-VE_{ij})V_{ij} \tag{51}$$
$$\frac{dE_{ij}}{dt} =\lambda_i(1-VE_{ij})V_{ij}-(1-p_{ij})\omega_i^{'}E_{ij}-p_{ij}\omega_i E_{ij} \tag{52}$$
$$\frac{dF_{ij}}{dt} =(1-p_{ij})\omega_i^{'}E_{ij}-\omega_i^{''}F_{ij} \tag{53}$$
$$\frac{dI_{ij}}{dt} =\omega_i^{''}F_{ij}-\gamma_i I_{ij} \tag{54}$$
$$\frac{dA_{ij}}{dt} =p_{ij}\omega_i E_{ij}-\gamma_i^{'}A_{ij} \tag{55}$$
$$\frac{dR_{ij}}{dt} =\gamma_i^{'}A_{ij}+\gamma_i I_{ij} \tag{56}$$

where subscript $i=1,2,\cdots,8$ and $j=0,1,2,3$ denote the subgroups of age i and vaccination status j; λ_i is the infection force acted on age group i produced by all infected population, and is defined as:

$$\lambda_i=\sum_{k=1}^{n}\beta_{ki}\left[\sum_{j=0}^{3}(I_{kj}+\kappa A_{kj}+\kappa^{'}F_{kj})\right], \tag{57}$$

where κ and $\kappa^{'}$ denote different transmissibility of asymptomatic and pre-symptomatic populations.

Then, $\lambda_i V_{ij}$ is the newly infection rate of subgroup ij. And by considering the vaccine effect inside age group i, we multiplies each V_{ij} by a constant vaccine effect coefficient $1-VE_{ij}$ (where the odd ratios are used practically). That is, the newly infection term in S5.

3.2 Parameters of The Multi-Group $VEFIAR$ Model

Variable interpretations of the $VEFIAR$ model are given in Table S1.

Table. S1: Variable Interpretations

Variables	Interpretation
group i	age group i
group ij	population of those vaccinated j doses in age group i
V_{ij}	population of susceptible in group ij
E_{ij}	those infected but not yet infectious in group ij
F_{ij}	pre-symptom state in group ij, which is infectious
I_{ij}	symptomatic cases in group ij
A_{ij}	asymptomatic cases in group ij
R_{ij}	removed in group ij

Parameter source, values and interpretations of the $VEFIAR$ model are given in Table S2.

Table. S2: Parameter Profiles

Parameter	Interpretation	Value (Range)	Unit	Source
n	number of age groups	8	1	self-defined
c_{ij}	daily average number of contact in age group j that a individual in age group i will produce	Figure. S2(d)	person$\cdot$ day^{-1}	data estimated
β_{ij}	transmission rate	-	day$^{-1}\cdot$ person^{-1}	compute by c_{ij} and R_0
VE_{ij}	the average vaccine efficacy of age group i with j-th doses	-	1	by OR via logistic regression
λ_i	force of infection acted to group i	-	day^{-1}	compute from β_{ij}, E_{ij}, A_{ij} and I_{ij}
$1/\omega_{ij}$	average latent period of those asymptomatic cases vaccinated j doses in age group i	3	day	reference?
$1/\omega_{ij}^{'}$	average latent period of those pre-symptomatic cases of vaccination status j in age group i	3	day	reference?
$1/\omega_{ij}^{''}$	average duration from pre-symptomatic F_{ij} to symptomatic I_{ij}	2	day	reference?
p_{ij}	probability that one exposed individual of vaccination status j in age group i will become symptomatic	0.31 for un-vaccinated; 0.62 for vaccinated	1	reference?
$1/\gamma$	average infectious period of those symptomatic with vaccination status j in age group i	5	day	reference?
$1/\gamma^{'}$	average infectious period of those asymptomatic of vaccination status j in age group i	7	day	reference?
κ	relative transmission ability of asymptomatic cases	0.35	1	reference?
$\kappa^{'}$	relative transmission ability of pre-symptomatic cases	0.63	1	reference?

3.3 Vaccine Coverage

Consider the vaccine coverage, we divide people by doses (0, 1, 2, 3 doses) and by vaccination status (none vaccinated, un-fully vaccinated, fully vaccinated and booster vaccinated) respectively. We present the coverage of vaccination among 14892 close contacts with contact age recorded. The coverage result are shown in Table S3 and S4.

The vaccination status of close contacts are obtained by comparing the maximum doses demanded for fully vaccination among brand of doses this contact vaccinated with the number of doses this close contact had vaccinated.

By the consideration of full information, we present only the sample count in each group instead of percentages (compressed data that can degenerated from sample counts).

Table. S3: Age-Stratified Vaccine Count via Data (by doses)

age group (years old)	0 dose	1 dose	2 doses	3 doses
0 to 9	911	0	0	0
10 to 19	563	624	326	32
20 to 29	576	251	1332	127
30 to 39	860	384	2494	307
40 to 49	461	180	1774	226
50 to 59	381	195	1453	293
60 to 69	182	119	389	63
$\geq$ 70	157	98	119	15

Table. S4: Age-Stratified Vaccine Count via Data (by states of vaccination)

age group (years old)	none vaccinated	un-fully vaccinated	fully vaccinated	booster vaccinated
0 to 9	911	0	0	0
10 to 19	563	632	350	0
20 to 29	576	292	1417	1
30 to 39	860	423	2762	0
40 to 49	461	209	1971	0
50 to 59	381	212	1729	0
60 to 69	182	128	443	0
$\geq$ 70	157	100	132	0

The following Figure S6 visualize the vaccine coverage in Table S4.

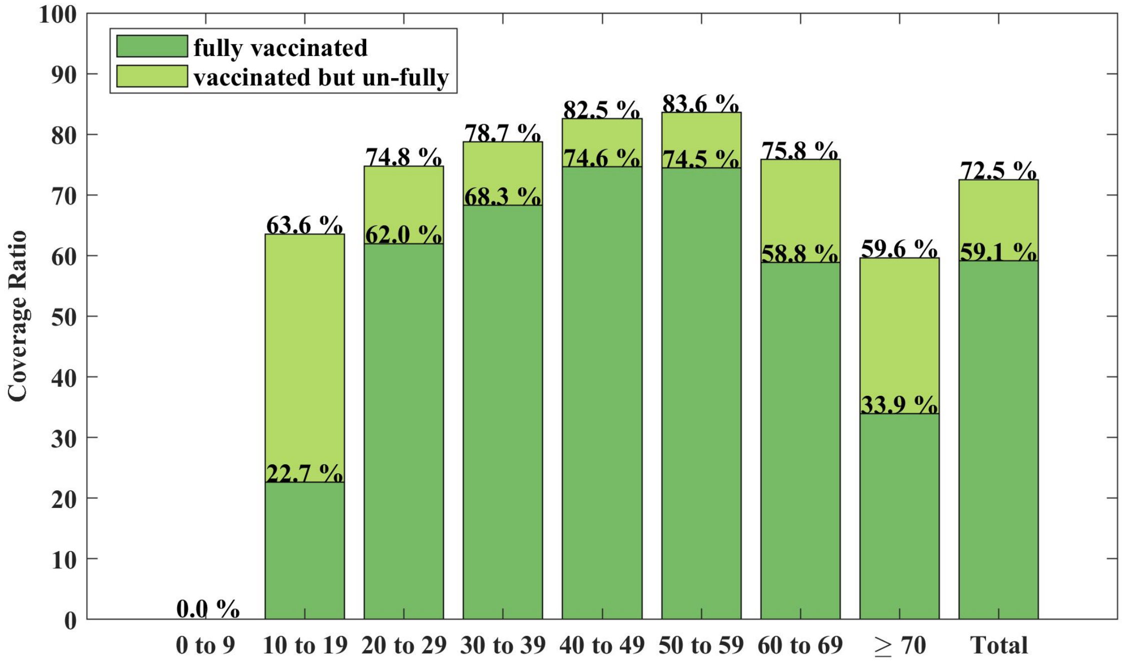

Figure. S6: Bar-plot of Vaccine Coverage

3.4 Vaccine Efficacy

3.4.1 Single-Variable Analysis of Total Attact Rate (TAR)

To handle the uncertainty of the collected data, we use bootstrap to construct the empirical distribution of all these TARs. We performed the bootstrap 10000 times on the contact data set, with each bootstrapped data set containing equally 10000 contacts. Each of those bootstrapped data sets produces a 8×4 matrix of TAR in age groups (in rows) and vaccine groups (in columns). Based on those resampled TARs, we depict the distribution of TAR in age groups and vaccine groups in box-plot respectively.

The box-plot in Figure S7 shows the different distribution of bootstrapped TAR in age groups and vaccine groups. By inspecting the notches, we conclude with 95% confidence that the median of TAR decrease by vaccination status. The TAR of age groups is more complicated and we shall inspect all possible impact factors in the next section.

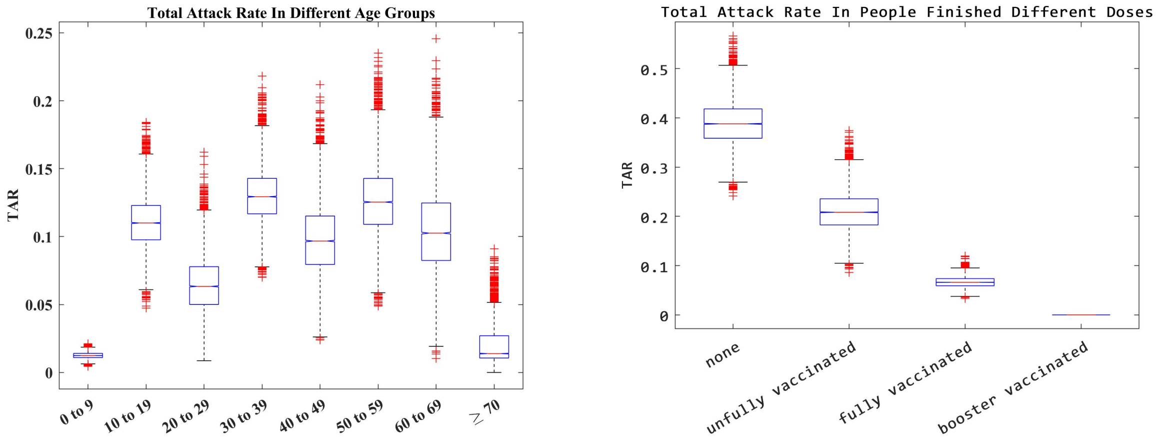

(a) (b)

Figure. S7: Total Attack Rate In Different Age Groups. (a): Total Attack Rate In Different Age Groups. The method of bootstrap is used to illustrate the distribution of TAR in specific groups. 10000 times of bootstrap of the close contact data are performed, with each bootstrap sample size equal 10000. Each bootstrapped data set produces a sample matrix of TAR (of each age/dose group). (b): Total Attack Rate In People Finished Different Doses. The method of bootstrap is used to illustrate the distribution of TAR in specific groups. 10000 times of bootstrap of the close contact data is performed, with bootstrap sample size equals 10000. Each bootstrapped data set produces a sample matrix of TAR (of each age/dose group)

3.4.2 Multi-Variable Analysis of Odds Ratio (OR)

We focus on the logistic regression model:

$$logit(\mathbb{E}\{Y|\mathbf{X}=\mathbf{x}\}) = [1, \mathbf{X}^T]\beta \tag{58}$$

$$\{Y|\mathbf{X}=\mathbf{x}\} \sim \text{binomial distribution} \tag{59}$$

where $logit(x) = ln(\frac{x}{1-x})$ is the canonical link function of binomial distribution; Y is the 0-1 random variable of whether a close contact is infected (1 represent infected, 0 represent un-infected); $\mathbf{X}$ is a 4×1 random vector with its entries be 4 factors: vaccinationStatus, contactAge, contactGender and exposureRanking; $\mathbf{x}$ is an observation of $\mathbf{X}$; β is a 5×1 vector of coefficients (include coefficient of intercept).

The coefficients are fitted using MATLAB:

Table. S5: Fitted Coefficients of Logistic Regression Model

	Estimate	SE	tStat	pValue
(Intercept)	1.1583 e+00	4.7690 e-02	2.4289 e+01	2.5497 e-130
contactAge	4.1351 e-03	9.4730 e-04	4.3651 e+00	1.2707 e-05
exposureRanking	-1.3681 e-01	4.5267 e-03	-3.0223 e+01	1.1734 e-200
contactGender	-5.9738 e-02	3.2193 e-02	-1.8556 e+00	6.3511 e-02
vaccinationStatus	-6.5545 e-01	1.7842 e-02	-3.6736 e+01	1.9437 e-295

We can see from Table that coefficients of all factors are significantly differ from 0, except the contactGender.

Based on the coefficient of contactAge and vaccinationStatus, the the odds ratio (OR) are computed for different age and vaccination groups by:

$$OR = e^{x_1^T \hat{\beta} - x_0^T \hat{\beta}} \tag{60}$$

where $\hat{\beta}$ is the fitted parameter vector of the logistic model; x_0 is the properties of reference population; x_1 is the properties of target population. (Note that the exposureRanking and the contactGender are the same in x_0 and x_1, and they are vanished by $x_1 - x_2$)

The odds ratios versus the un-vaccinated aged greater than 70 are shown as Table S6. Odds ratios versus the average age (38.6 year old) and average vaccine status (1.32) in Table S4 are shown in Table S7.

Table. S6: Odds Ratio for Age and Vaccination Groups (versus ≥ 70, un-vaccinated)

	non-vaccinated	un-fully vaccinated	fully vaccinated	booster vaccinated
0 to 9	7.4867 e-01	3.8872 e-01	2.0182 e-01	1.0479 e-01
10 to 19	7.8028 e-01	4.0513 e-01	2.1034 e-01	1.0921 e-01
20 to 29	8.1322 e-01	4.2223 e-01	2.1922 e-01	1.1382 e-01
30 to 39	8.4755 e-01	4.4006 e-01	2.2848 e-01	1.1863 e-01
40 to 49	8.8333 e-01	4.5863 e-01	2.3813 e-01	1.2364 e-01
50 to 59	9.2063 e-01	4.7800 e-01	2.4818 e-01	1.2886 e-01
60 to 69	9.5949 e-01	4.9818 e-01	2.5866 e-01	1.3430 e-01
≥ 70	1.0000 e+00	5.1921 e-01	2.6958 e-01	1.3997 e-01

Table. S7: Odds Ratio for Age and Vaccination Groups (versus average age of 38.6 years old and average vaccination status of 1.32

	non-vaccinated	un-fully vaccinated	fully vaccinated	booster vaccinated
0 to 9	2.0672 e+00	1.0733 e+00	5.5727 e-01	2.8934 e-01
10 to 19	2.1545 e+00	1.1186 e+00	5.8080 e-01	3.0155 e-01
20 to 29	2.2454 e+00	1.1658 e+00	6.0532 e-01	3.1429 e-01
30 to 39	2.3402 e+00	1.2151 e+00	6.3087 e-01	3.2755 e-01
40 to 49	2.4390 e+00	1.2664 e+00	6.5751 e-01	3.4138 e-01
50 to 59	2.5420 e+00	1.3198 e+00	6.8526 e-01	3.5579 e-01
60 to 69	2.6493 e+00	1.3755 e+00	7.1419 e-01	3.7082 e-01
≥ 70	2.7612 e+00	1.4336 e+00	7.4435 e-01	3.8647 e-01

Based on the odds ratio obtained by coefficients of logistic regression, we define $VE = 1 - OR$ to describe the reduction of newly infections in model. That is, multiply $\lambda_i V_{ij}$ by $(1 - VE_{ij})$. Note that this 'VE' is not any one of vaccination efficacy in common sense, instead, a mixture of age effect, reduction of susceptibility and reduction of transmissibility. In the simulation, odd ratios in S7 are used to replace coefficients $1 - VE$ in newly infections in Figure S5

To obtain the distribution of coefficients in logistic model, we use pair bootstrap, which resamples the factor-

outcome pairs of all contacts 1000 times, with each bootstrap sample size equals to 10000. For each bootstrapped data set, coefficients of the logistic model is fitted, and all those coefficients forms the empirical distribution of coefficients. The resulted distribution is visualized by box-chart in Figure S8.

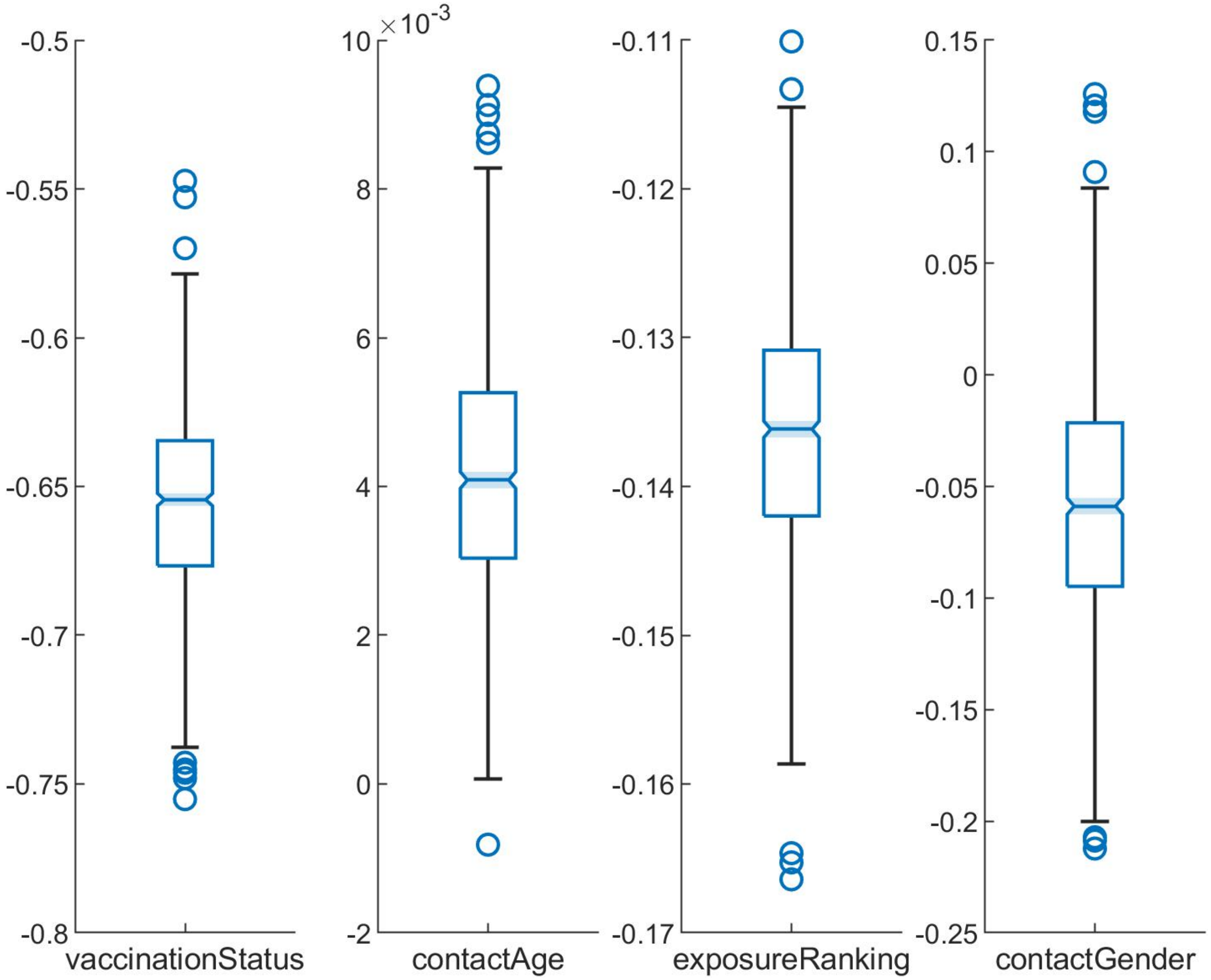

Figure. S8: Distribution of Coefficients in Logistic Model. By pair bootstrap of 1000 times with each resampled data set of size 10000.

One can conclude whether a coefficient is significantly differ from 0 by inspecting whether the notches of box-chart overlap zero.

The distribution of odds ratios is then computed using those 1000 coefficients pairs (vaccinationStatus, contacAge) trained on 1000 bootstrapped samples.

3.5 Solve q From R_0

As Lemma 2.3 pointed out, we can combine the vaccination groups inside each age group in the 8×4 age-vaccination grouped $VEFIAR$ model. The R_0 under this model framework is then computed using the 8 age group $VEFIAR$ model, with parameters of un-vaccinated in each groups.

We compute the basic reproduction number R_0 using the Van den Driessche and Watmough approach in next-generation methods as follow.

3.5.1 Step 1

The basic reproduction number is defined on a entirely susceptible population, that we will consider all age groups are un-vaccinated. Assume that the population been divided into n age-groups. First, we divide the $6 * n$ variables (or sometimes called 'compartments') $(V_i, E_i, F_i, I_i, A_i, R_i), \quad i = 1, 2, \cdots, n$ into two categories: the first $(E_i, F_i, I_i, A_i), \quad i = 1, 2, \cdots, n$ are infected compartments, and the second $(V_i, R_i), \quad i = 1, 2, \cdots, n$ are non-infected compartments.

3.5.2 Step 2

Divide the derivatives of $(E_i, F_i, I_i, A_i), \quad i = 1, 2, \cdots, n$ into two parts: the first part $\mathscr{F}$ denotes the rate of newly infection, and the second part $\mathcal{V}$ denotes the transition inside the infected compartments:

$$\frac{d}{dt}\begin{bmatrix} E_i \\ F_i \\ I_i \\ A_i \end{bmatrix} = \begin{bmatrix} \sum_{j=1}^{n} \beta_{ji} S_i (I_j + \kappa A_j + \kappa' F_j) - p_i \omega_i E_i - (1 - p_i)\omega_i' E_i \\ (1 - p_i)\omega_i' E_i - \omega_i'' F_i \\ \omega_i'' F_i - \gamma_i I_i \\ p_i \omega_i E_i - \gamma_i' A_i \end{bmatrix} \tag{61}$$

$$= \begin{bmatrix} \sum_{j=1}^{n} \beta_{ji} S_i (I_j + \kappa A_j + \kappa' F_j) \\ 0 \\ 0 \\ 0 \end{bmatrix} - \begin{bmatrix} p_i \omega_i E_i + (1 - p_i)\omega_i' E_i \\ -(1 - p_i)\omega_i' E_i + \omega_i'' F_i \\ -\omega_i'' F_i + \gamma_i I_i \\ -p_i \omega_i E_i + \gamma_i' A_i \end{bmatrix} \tag{62}$$

$$\stackrel{def}{=} \mathscr{F}_i - \mathscr{V}_i \tag{63}$$

3.5.3 Step 3

Taking derivatives with respect to infected variables (E_i, F_i, I_i, A_i), $i = 1, 2, \cdots, n$ for vector $\mathscr{F}$ and $\mathscr{V}$, the jacobi matrices F and V are obtained:

$$F = \begin{bmatrix} F_{11} & F_{12} & \cdots & F_{1n} \\ F_{21} & F_{22} & \cdots & F_{2n} \\ \vdots & \vdots & \ddots & \vdots \\ F_{n1} & F_{n2} & \cdots & F_{nn} \end{bmatrix}, \quad V = \begin{bmatrix} V_{11} & V_{12} & \cdots & V_{1n} \\ V_{21} & V_{22} & \cdots & V_{2n} \\ \vdots & \vdots & \ddots & \vdots \\ V_{n1} & V_{n2} & \cdots & V_{nn} \end{bmatrix}$$

where

$$F_{ij} = \begin{bmatrix} \partial\mathcal{F}_i(1)/\partial E_j & \partial\mathcal{F}_i(1)/\partial F_j & \partial\mathcal{F}_i(1)/\partial I_j & \partial\mathcal{F}_i(1)/\partial A_j \\ \partial\mathcal{F}_i(2)/\partial E_j & \partial\mathcal{F}_i(2)/\partial F_j & \partial\mathcal{F}_i(2)/\partial I_j & \partial\mathcal{F}_i(2)/\partial A_j \\ \partial\mathcal{F}_i(3)/\partial E_j & \partial\mathcal{F}_i(3)/\partial F_j & \partial\mathcal{F}_i(3)/\partial I_j & \partial\mathcal{F}_i(3)/\partial A_j \\ \partial\mathcal{F}_i(4)/\partial E_j & \partial\mathcal{F}_i(4)/\partial F_j & \partial\mathcal{F}_i(4)/\partial I_j & \partial\mathcal{F}_i(4)/\partial A_j \end{bmatrix} = \begin{bmatrix} 0 & \kappa^{'}\beta_{ji}S_i & \beta_{ji}S_i & \kappa\beta_{ji}S_i \\ 0 & 0 & 0 & 0 \\ 0 & 0 & 0 & 0 \\ 0 & 0 & 0 & 0 \end{bmatrix},$$

$$V_{ij} = \begin{bmatrix} \partial\mathcal{V}_i(1)/\partial E_j & \partial\mathcal{V}_i(1)/\partial F_j & \partial\mathcal{V}_i(1)/\partial I_j & \partial\mathcal{V}_i(1)/\partial A_j \\ \partial\mathcal{V}_i(2)/\partial E_j & \partial\mathcal{V}_i(2)/\partial F_j & \partial\mathcal{V}_i(2)/\partial I_j & \partial\mathcal{V}_i(2)/\partial A_j \\ \partial\mathcal{V}_i(3)/\partial E_j & \partial\mathcal{V}_i(3)/\partial F_j & \partial\mathcal{V}_i(3)/\partial I_j & \partial\mathcal{V}_i(3)/\partial A_j \\ \partial\mathcal{V}_i(4)/\partial E_j & \partial\mathcal{V}_i(4)/\partial F_j & \partial\mathcal{V}_i(4)/\partial I_j & \partial\mathcal{V}_i(4)/\partial A_j \end{bmatrix} = \delta_{ij} \begin{bmatrix} p_i\omega_i + (1-p_i)\omega_i^{'} & 0 & 0 & 0 \\ -(1-p_i)\omega_i^{'} & \omega_i^{''} & 0 & 0 \\ 0 & -\omega_i^{''} & \gamma_i & 0 \\ -p_i\omega_i & 0 & 0 & \gamma_i^{'} \end{bmatrix},$$

δ_{ij} is the Kronecker Delta.

The inverse of V_{jj} is further computed:

$$V_{jj}^{-1} = \begin{bmatrix} \frac{1}{p_i\omega_i+(1-p_i)\omega_i^{'}} & 0 & 0 & 0 \\ \frac{\omega_i^{'}\,(1-p_i)}{\omega_i^{''}\left(\omega_i\,p_i+(1-p_i)\omega_i^{'}\right)} & \frac{1}{\omega_i^{''}} & 0 & 0 \\ \frac{\omega_i^{'}\,\omega_i^{''}\,(1-p_i)}{\gamma_i\omega_i^{''}\left(\omega_i\,p_i+(1-p_i)\omega_i^{'}\right)} & \frac{\omega_i^{''}}{\gamma_i\omega_i^{''}} & \frac{1}{\gamma_i} & 0 \\ \frac{\omega_i\,p_i}{\gamma\left(+\omega_i\,p_i+(1-p_i)\omega_i^{'}\right)} & 0 & 0 & \frac{1}{\gamma_i^{'}} \end{bmatrix} \tag{64}$$

3.5.4 Step 4

Construct the next generation matrix $M = FV^{-1}$.

Block matrices M, F and V:

$$M = \begin{bmatrix} M_{11} & M_{12} & \cdots & M_{1n} \\ M_{21} & M_{22} & \cdots & M_{2n} \\ \vdots & \vdots & \ddots & \vdots \\ M_{n1} & M_{n2} & \cdots & M_{nn} \end{bmatrix} = \begin{bmatrix} F_{11} & F_{12} & \cdots & F_{1n} \\ F_{21} & F_{22} & \cdots & F_{2n} \\ \vdots & \vdots & \ddots & \vdots \\ F_{n1} & F_{n2} & \cdots & F_{nn} \end{bmatrix} \begin{bmatrix} V_{11}^{-1} & & & \\ & V_{22}^{-1} & & \\ & & \ddots & \\ & & & V_{nn}^{-1} \end{bmatrix},$$

where

$$M_{ij} = \sum_{k=1}^{n} F_{ik} V_{kj}^{-1} = F_{ij} V_{jj}^{-1} = \begin{bmatrix} a_{ij} & b_{ij} & c_{ij} & d_{ij} \\ 0 & 0 & 0 & 0 \\ 0 & 0 & 0 & 0 \\ 0 & 0 & 0 & 0 \end{bmatrix},$$

$$\begin{aligned} a_{ij} =& \frac{\omega_i^{'}\,(1-p_i)\,\kappa^{'}\beta_{ji}S_i}{\omega_i^{''}\left(\omega_i\,p_i + (1-p_i)\omega_i^{'}\right)} + \frac{\omega_i^{'}\,\omega_i^{''}\,(1-p_i)\,\beta_{ji}S_i}{+\gamma_i\omega_i^{''}\left(\omega_i\,p_i + (1-p_i)\omega_i^{'}\right)} + \frac{\omega_i\,p_i\kappa\beta_{ji}S_i}{+\gamma\left(\omega_i\,p_i + (1-p_i)\omega_i^{'}\right)}, \\ b_{ij} =& \frac{\kappa^{'}\beta_{ji}S_i}{\omega_i^{''}} + \frac{\omega_i^{''}\beta_{ji}S_i}{\gamma_i\omega_i^{''}}, \\ c_{ij} =& \frac{\beta_{ji}S_i}{\gamma_i}, \\ d_{ij} =& \frac{\kappa\beta_{ji}S_i}{\gamma}. \end{aligned}$$

3.5.5 Step 5

Compute the eigenvalues:

$$R_{eff} = \lambda_{max}\,(M)$$

3.5.6 Step 6

R_0 is obtained by substitute the population vector of disease-free equilibrium in the R_{eff} expression.

3.5.7 Step 7

By substituting $\beta_{ij} = c_{ji}q\sigma_j/N_i$, equation is indeed a linear equation of one unknown variable q. With contact matrix and other parameters in model known, one can always solve q from this equation.

Typically, if all parameters except those in newly infection term are group irrelevant, then using notation

$b_{ij} = N_i\beta_{ji}, \quad i,j = 1,2,\cdots,n$, we have a simple and explicit expression of q:

$$q = R_0 / \left\{ \frac{\lambda_{\max}(B/q)}{p\omega + (1-p)\omega'} \left[\frac{\kappa p\omega}{\gamma'} + \frac{(1-p)\omega'}{\gamma} + \frac{\kappa'(1-p)\omega^{'}}{\omega''} \right] \right\} \tag{65}$$

$$= R_0 / \left\{ \frac{\lambda_{\max}(C^T. * \vec{\sigma}./\vec{N}^T)}{p\omega + (1-p)\omega'} \left[\frac{\kappa p\omega}{\gamma'} + \frac{(1-p)\omega'}{\gamma} + \frac{\kappa'(1-p)\omega^{'}}{\omega''} \right] \right\} \tag{66}$$

where

$$B = \begin{bmatrix} b_{11} & b_{12} & \cdots & b_{1n} \\ b_{21} & b_{22} & \cdots & b_{2n} \\ \vdots & \vdots & \ddots & \vdots \\ b_{n1} & b_{n2} & \cdots & b_{nn} \end{bmatrix} \tag{67}$$

Note that the right hand side of the above expression is irrelevant to q (q is eliminated in B/q), that is, one R_0 corresponding one probability of infection once the model parameters are fixed.

Based on parameters taken in section 3.2 and vaccine coverage estimated in section 3.3, the probability q under different R_0 are shown in table S8 below:

Table. S8: Probability of Infection via Single Contact

R_0	q_NGM	q_DBM
1.0	0.02163	0.0149
2.0	0.04325	0.02979
3.0	0.06488	0.0525
4.0	0.0865	0.07923
5.0	0.1081	0.1042
6.0	0.1298	0.1268
7.0	0.1514	0.1484
8.0	0.173	0.1696

All β_{ij} are then reconstructed from contact matrix C and q via the decomposition $\beta_{ij} = c_{ji} q \sigma_j / N_i$.

3.6 Simulation of The Multi-Group $VEFIAR$ Model

We performed simulation using the $VEFIAR$ model for verifying the stability of model. The simulation start with one symptomatic case in age group 30 to 39, fully vaccinated, corresponding to the case of earliest illness onset among 129 cases (38 years old, female, fully vaccinated, moderate clinical outcome). We replaced the coefficients

$1 - VE$ in newly infections in Figure S5 by the odd ratios in Table S7. The initial population state is obtained by distributing population of each age group into 4 vaccination status using vaccine coverage in Table S4.

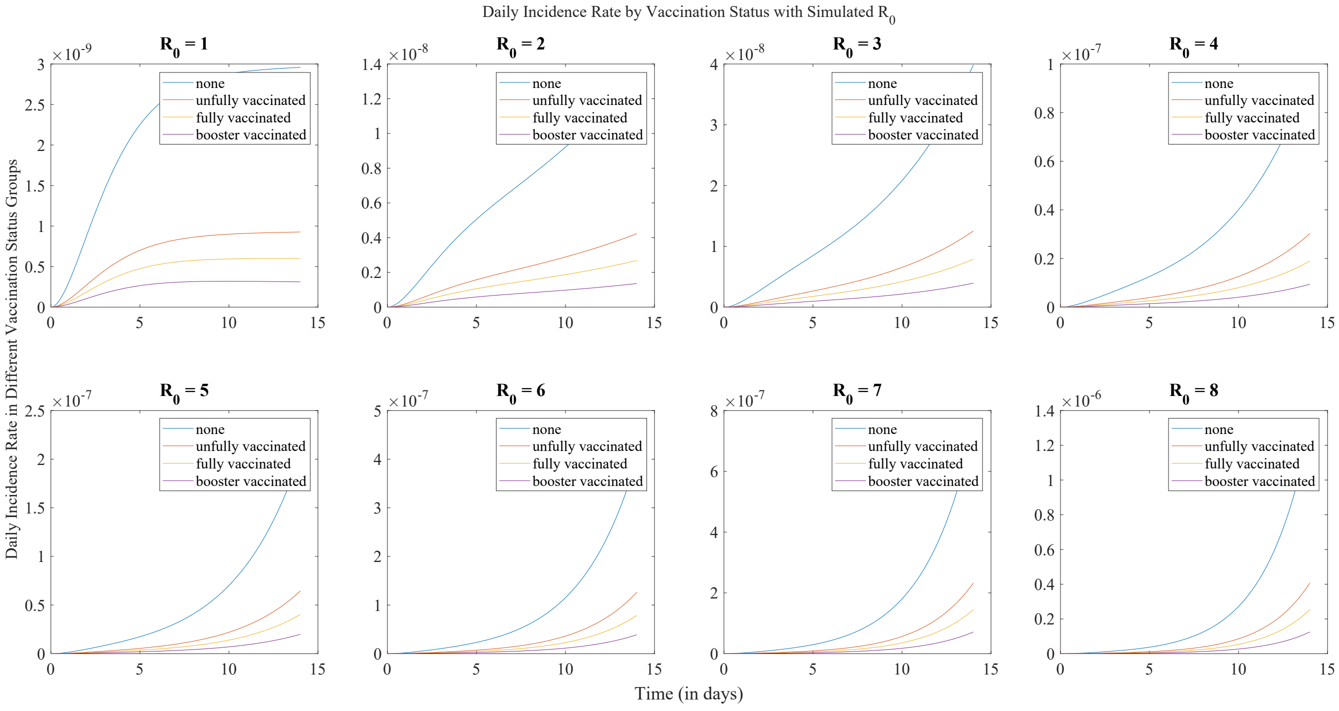

(a)

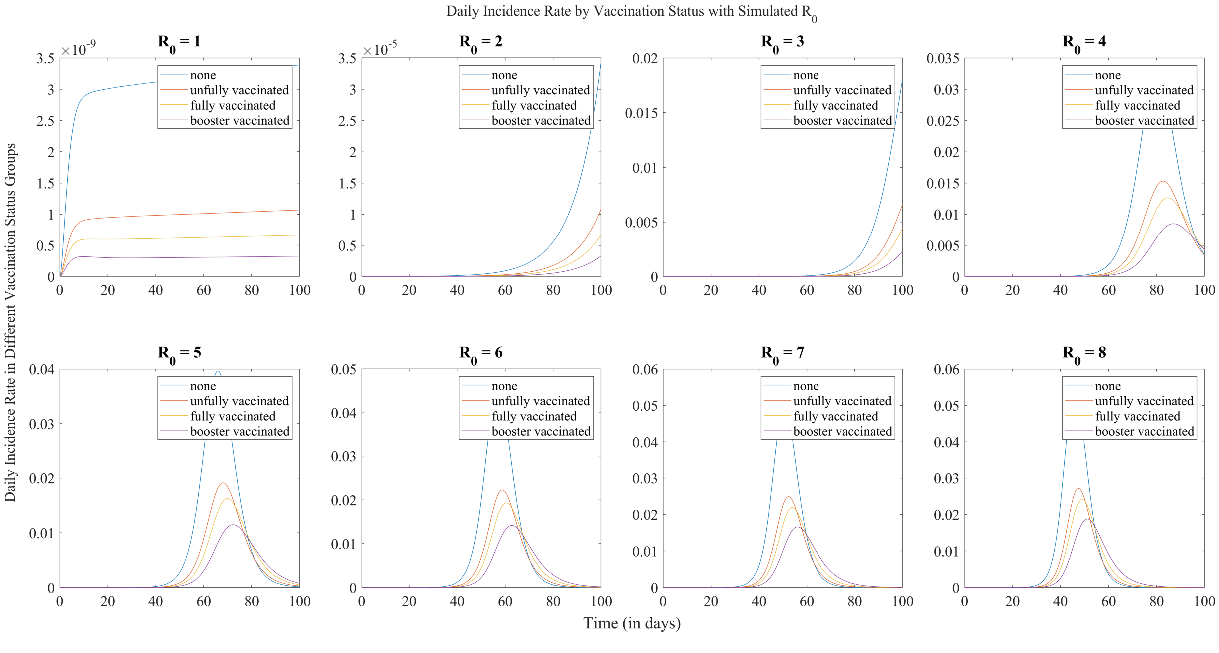

(b)

Figure. S9: Daily Incidence Rate by Vaccination Status with Simulated R_0; (a): A 14-Days Simulation, (b): A 100-days Simulation

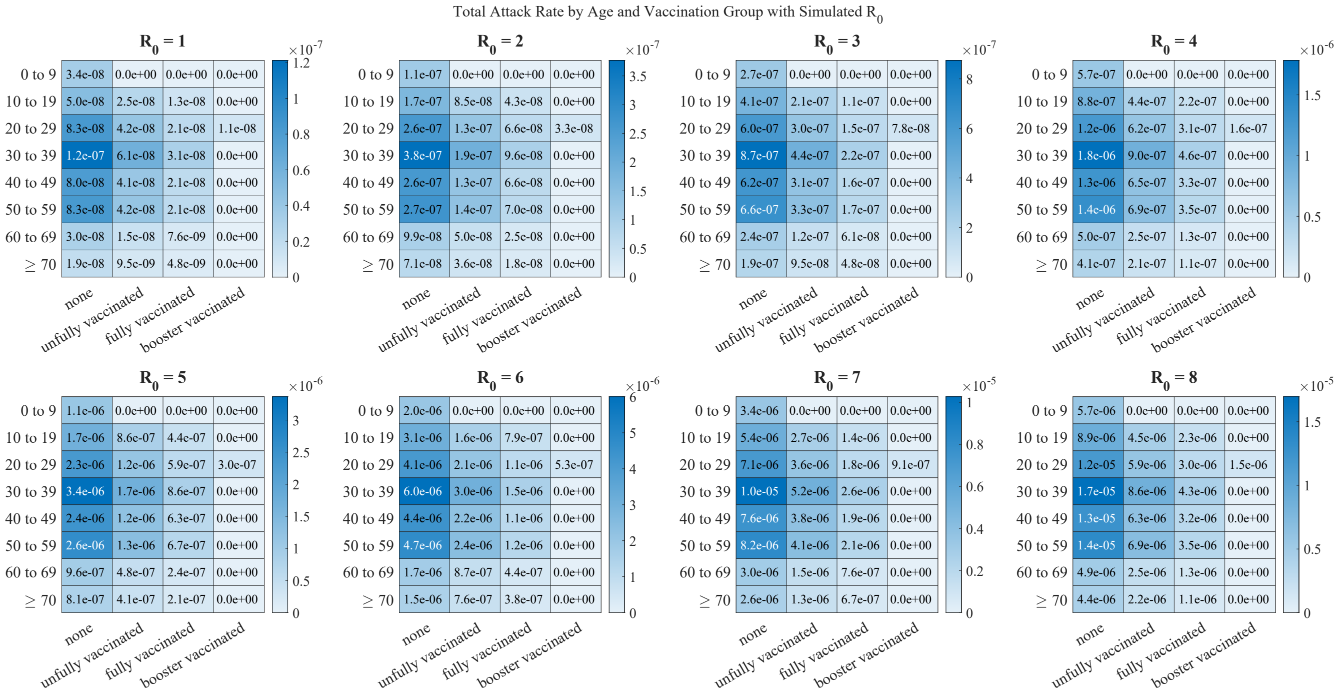

(a)

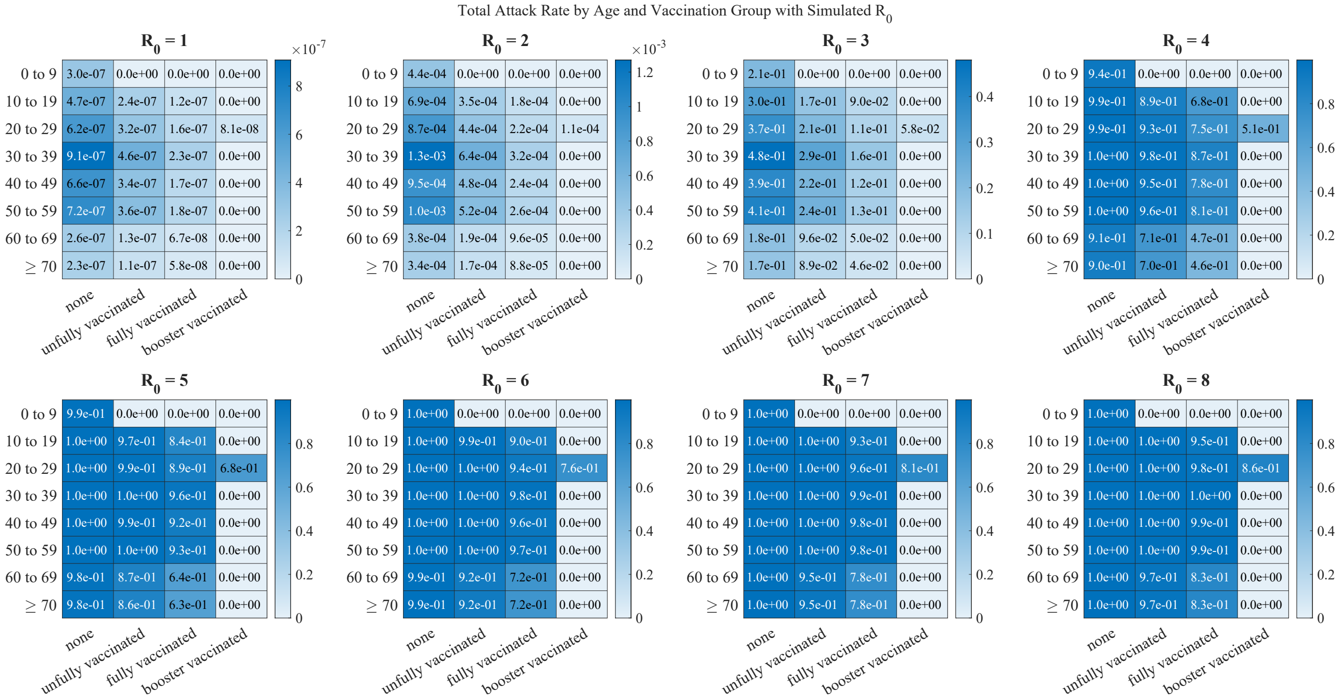

(b)

Figure. S10: Total Attack Rate By Age and Vaccination Group With Simulated R_0; (a): A 14-Days Simulation, (b): A 100-days Simulation. The lower right corner of entries are zeros because its zero vaccination coverage, which leads the population size be zeros in these groups.

4 Optimization of Time-Varying Vaccination

There are two questions:

- If one has 1 dose vaccine available, then who should got this dose?
- If there are 100 million doses available, then how shall we distribute them? (to each age group and to those already vaccinated 0, 1, or 2 doses)

To answer those two questions, define the following optimization problem, and use the directional derivative as a criterion for vaccine distributing.

4.1 The Optimization Method

All accumulative cases in a given time period is an important index for effectiveness of local disease control. In the construction of the objective function, we can weight the cost of cases by the age-partitioned mobility rate or other severity rankings (asymptomatic, mild, severe, ICU).

Objective Function:

$$f = \sum \text{Weighted Costs of all accumulative cases in a given time period} \tag{68}$$
$$= \sum_{i=1}^{n} \sum_{j=0}^{3} \int_0^T g(E_{ij}, I_{ij}, A_{ij}, Q_{ij}, parameters)dt \tag{69}$$

where $g(\cdot)$ denote the weight function; i denote the i-th age group; j denote group of those finished j-th doses; E_{ij}, I_{ij}, A_{ij}, Q_{ij} denotes the solution of the systems of ODEs; parameters denotes the constant parameters in the systems of ODEs.

The objective function f is implicit since there is no explicit solutions of E_{ij}, I_{ij}, A_{ij}, Q_{ij}. Therefore, as a good approximation of the original objective function f, the numerical solution is adopted with a given method and a given step size choosing method. That is, we use the following objective function $\tilde{f}$ instead:

$$\tilde{f} = \sum_{i=1}^{n} \sum_{j=0}^{3} \int_0^T g(\tilde{E}_{ij}, \tilde{I}_{ij}, \tilde{A}_{ij}, \tilde{Q}_{ij}, parameters, method, stepSize)dt \tag{70}$$

here $\int$ denotes the numerical integration; $\tilde{E}_{ij}, \tilde{I}_{ij}, \tilde{A}_{ij}, \tilde{Q}_{ij}$ denotes the numerical solution of the systems of ODEs using given numerical method and stepSize choosing method. For the sake of convenience, we use symbol f repre-

sent $\tilde{f}$ in following sections.

In the dose-wise vaccination process, we use the following objective function:

$$f = \sum_{i=1}^{n}\sum_{j=0}^{3} \tau_{ij} \int_{0}^{T} \left[p_{ij}\omega_{ij}E_{ij} + (1-p_{ij})\omega_{ij}^{'}E_{ij} \right] dt, \tag{71}$$

where τ_{ij} is the group-wise weight, taken values of ones for cumulative cases, and take values in table S10 for hospitalized and fatality respectively.

We treated those compartment as continuous variable, and use 4-order Runge-Kuatt method with fixed step size to perform numerical integrals to construct the objective function. Step sizes are set to be fixed 1.

Decision Variable:

The population size nVC_{ij} of group ij (intersection of age group i and vaccination group j) at discrete dose grids $d_0, d_1, \cdots, d_M$. Typically, these instances are set to be grids of the discretization scheme in the numerical method.

$$nVC(d) = \begin{pmatrix} \mathrm{nVC}_{10} & \mathrm{nVC}_{11} & \mathrm{nVC}_{12} & \mathrm{nVC}_{13} \\ \mathrm{nVC}_{20} & \mathrm{nVC}_{21} & \mathrm{nVC}_{22} & \mathrm{nVC}_{23} \\ \mathrm{nVC}_{30} & \mathrm{nVC}_{31} & \mathrm{nVC}_{32} & \mathrm{nVC}_{33} \\ \mathrm{nVC}_{40} & \mathrm{nVC}_{41} & \mathrm{nVC}_{42} & \mathrm{nVC}_{43} \\ \mathrm{nVC}_{50} & \mathrm{nVC}_{51} & \mathrm{nVC}_{52} & \mathrm{nVC}_{53} \\ \mathrm{nVC}_{60} & \mathrm{nVC}_{61} & \mathrm{nVC}_{62} & \mathrm{nVC}_{63} \\ \mathrm{nVC}_{70} & \mathrm{nVC}_{71} & \mathrm{nVC}_{72} & \mathrm{nVC}_{73} \\ \mathrm{nVC}_{80} & \mathrm{nVC}_{81} & \mathrm{nVC}_{82} & \mathrm{nVC}_{83} \end{pmatrix} (d) \tag{72}$$

Note that f is viewed as a function of nVC, that is, $f(nVC)$.

Feasible Set:

We may normalize each entry of nVC by population size of corresponding age group, and obtain a vaccine coverage ratio VC.

The feasible set is a set of all point VC that is feasible. Those points satisfies: 1) each entry of VC is non-negative, 2) summation of each row of VC equals to 1 (for nVC, this summation equals to the population size of the corresponding age group). Such feasible set is indeed a probabilistic simplex with linear constrains (i.e. intersection of a probabilistic simplex and a hyperplane). Therefore, one may consider the optimization on manifolds, use the Riemann Gradient instead of Euclidean Gradient for updating, or to consider the alternative projection between

the simplex and the hyperplane.

4.2 Directional Derivatives of All Possible Update

This is optimal control problem, a difficult optimization problem that without the gradient or hessian or any other information that how f behaves on the feasible set, only the computation of the objective function itself is available. One may consider the Intelligent Optimization Algorithms (Genetic Algorithm, Simulated annealing algorithm, Evolutionary computation, Ant colony optimization algorithms, Immune Algorithm, Tabu Search Algorithm), Nelder-Mead simplex algorithm, and other direct method that only computations of objective function are required.

Unlike those global methods that searching on the entire feasible set, we use the greedy algorithm that updates with optimal directional derivative (the action of gradient on specific doses assignments) in each iteration. Here the directional derivative is a criteria for effectiveness of distributing a certain dose. Numerical directional derivatives is computed in each iteration for choosing a direction for update.

Assume that the current vaccine coverage is describe by nVC, then for each newly vaccinated dose, the coverage nVC is added with a one-dose perturbation ΔnVC_{ij}, a 8×4 matrix with its (i, j)-th entry equals to -1, and $(i, j+1)$ entry equals to 1, and otherwise 0. Those ΔnVC_{ij} contains all possible direction for updating.
(Note that here we do not distinguish dose-demand for different vaccines. A simplification is made by assuming that non-vaccinated, un-fully vaccinated, fully vaccinated, booster vaccinated are corresponding to 0, 1, 2, 3 doses.)

For example, when one individual of age group 7 who has already finished 1 dose is further vaccinated 1 dose, then the following perturbation is added to the current vaccine coverage nVC:

$$\Delta nVC_{71} = \begin{pmatrix} 0 & 0 & 0 & 0 \\ 0 & 0 & 0 & 0 \\ 0 & 0 & 0 & 0 \\ 0 & 0 & 0 & 0 \\ 0 & 0 & 0 & 0 \\ 0 & 0 & 0 & 0 \\ 0 & -1 & 1 & 0 \\ 0 & 0 & 0 & 0 \end{pmatrix} \tag{73}$$

The differences of objective function:

$$\Delta f_{ij} = f(nVC) - f(nVC + \Delta nVC_{ij}) \tag{74}$$

represent the effectiveness of this dose distributed to a individual of age group i who has already finished j doses. Hence, by computing the maximum Δf_{ij} in each step, a series of vaccinating decision is obtained.

4.3 Greedy Algorithm for Optimization of Vaccinate Process

4.3.1 Pseudo-Code

Algorithm 1 Greedy Alogrithm for Vaccinating Process

Input: Objective function $f(nVC)$ for minimization; Current vaccine coverage nVC; Number of available doses M; Batch-size m for vaccination; Parameters of the VEFIAR dynamical system; Days T for simulation.

Output: A series of vaccination coverage nVC, that each corresponding to one vaccination decision.

for $i = 1$ to ceil(M/m) **do**
  for all possible decision ΔnVC of the i-th dose distributing **do**
    Compute and save $\Delta f = f(nVC) - f(nVC + m \cdot \Delta nVC)$, the decrease of the specific decision
  end for
  Find the decision ΔnVC_i that produce maximum reduction of Δf
  Update the current coverage nVC by $nVC = nVC + m \cdot \Delta nVC_i$
  Save current nVC
end for
Output a series of nVC, with each nVC corresponding to one vaccination decision.

where ceil() represent rounding toward the infinitesimal large.

4.4 Optimization Profiles

The parameter settings of the optimization algorithm are listed in Table S9.

Table. S9: Parameters In Optimization

Parameter	Interpretation	Value	unit
T	time span of integration in objective function	14	day
M	number of doses to be vaccinated	1e8	dose
m	batch-size for vaccination	5e4	dose
method	method of discretization of ODE	fixed step-size Runge-Kutta of order 4	-
stepSize	step-size of discretization method	1	day

We use three kinds of weights for objective function 71: average weights for cumulative cases, weights for hospitalization and weights for fatality. The last two weights are computed from table S10 summarized form reference ??. Note that the objective function does not change when multiplied by a constant. Thus weights of each groups will be obtained by multiplying the relative ratio of its age group and its vaccination status group.

Table. S10: Weight In Objective Function

relative ratio	Omicron Fatality	Delta Fatality	Omicron Hospitalized	Delta Hospitalized
$0-9$	1.1	0.46	0.005	0.001
$10-19$	0.38	0.33	0.001	0.001
$20-29$	0.6	1.33	0.002	0.004
$30-39$	0.66	1.5	0.005	0.04
$40-49$	0.59	1.4	0.01	0.05
$50-59$	0.77	2.38	0.05	0.26
$60-69$	1.39	5.29	0.2	1.12
$70-79$	3.57	13.4	0.83	4.95
$80-$	11.1	25.4	5.12	15.9
un-vaccinated	0.01337	0.02344	0.1034	0.0278
fully vaccinated	0.002165	0.01351	0.02309	0.01054
booster vaccinated	0.004		0.0166	0.0043

4.5 Optimized Results

Based on parameters in Table S9, the Optimal Vaccinating Process Under Current Contact Pattern and Vaccine Coverage are shown in following 12 figures for 12 different R_0 of $[1, 2, \cdots, 12]$. For each figure, 8 sub-figures represent the optimal vaccination process in 8 age groups. The x-axis represent the dose-wise vaccination process; y-axis represent vaccine coverage inside the age group (population size of a vaccination status or its high status).

In the upper-right sub-figure (age group 30-39), the population size vaccinated increase since the first dose (un-vaccinated changed into those un-fully vaccinated), which indicates those un-vaccinated should be vaccinated first. This result is stable for $R_0 = [1, 2, \cdots, 12]$.

4.5.1 Cumulative Cases for Delta Variant

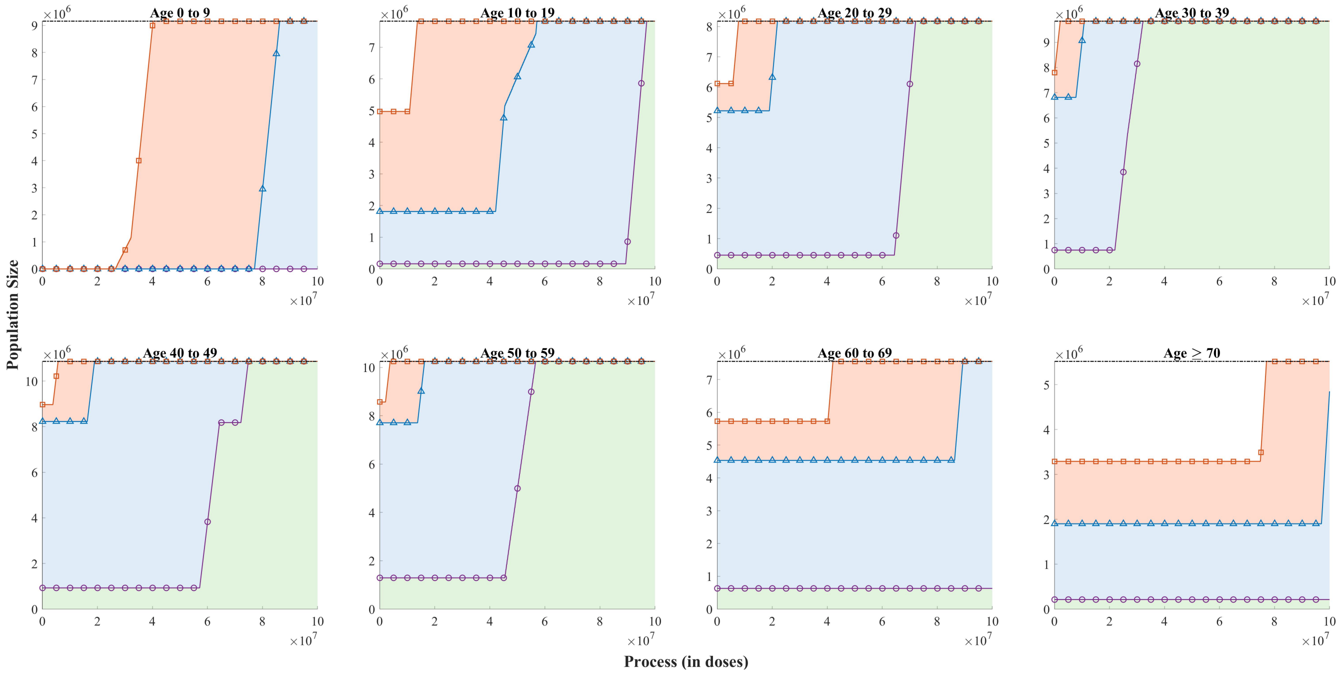

Figure. S11: Optimized Vaccinating Strategy Under Current Contact Pattern and Vaccine Coverage. For minimizing cumulative cases of Delta variant with $R_0 = 1$. 8 subfigures represent the optimal vaccination process in 8 age groups. The x-axis represent the dose-wise vaccination process; y-axis represents vaccine coverage inside the age group (populatioon size of four vaccination status). The purple line with circles denote the population size of booster vaccinated; the blue line with triangles denote the population size of at least fully-vaccinated (including fully vaccinated and booster vaccinated); the red line with squares denote the population size of at least vaccinated (including un-fully vaccinated, fully vaccinated, and booster vaccinated). These lines depicts how coverage changes with optimized vaccination process. The line increased from the first dose gives the specific information about which should be vaccinated first.

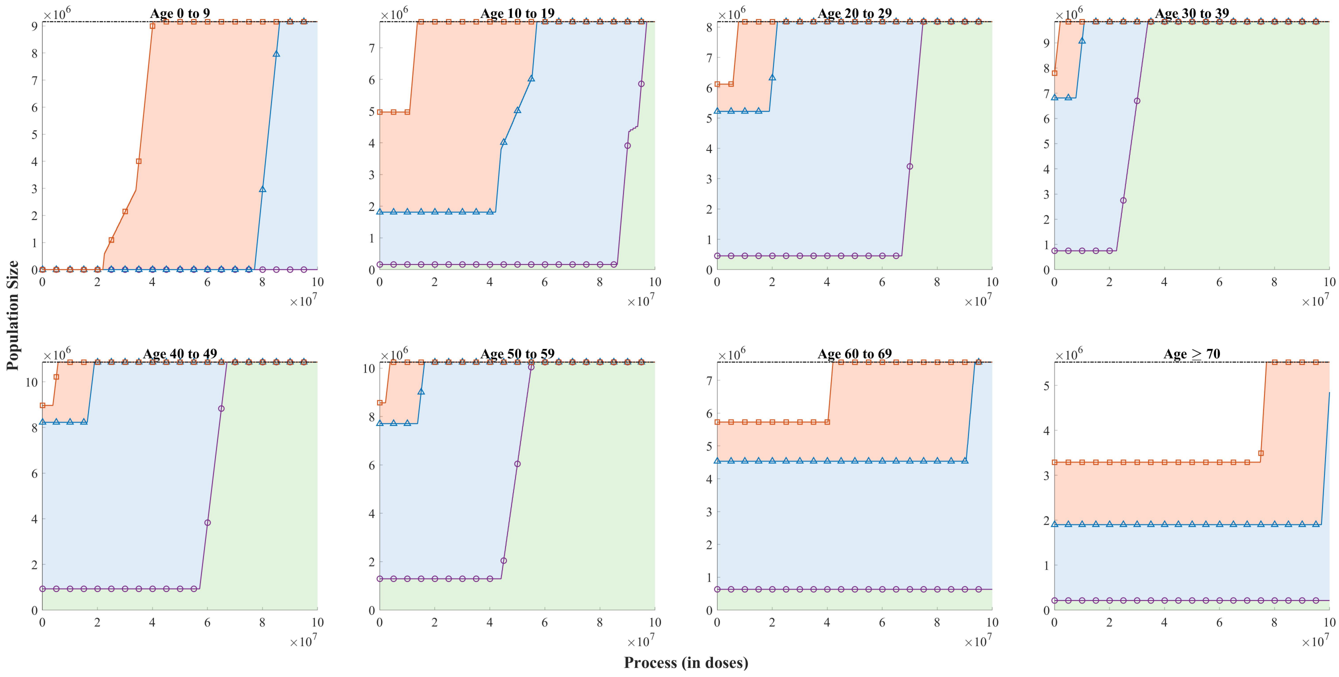

Figure. S12: Optimized Vaccinating Strategy Under Current Contact Pattern and Vaccine Coverage. For minimizing cumulative cases of Delta variant with $R_0 = 2$. 8 subfigures represent the optimal vaccination process in 8 age groups. The x-axis represent the dose-wise vaccination process; y-axis represents vaccine coverage inside the age group (populatioon size of four vaccination status). The purple line with circles denote the population size of booster vaccinated; the blue line with triangles denote the population size of at least fully-vaccinated (including fully vaccinated and booster vaccinated); the red line with squares denote the population size of at least vaccinated (including un-fully vaccinated, fully vaccinated, and booster vaccinated). These lines depicts how coverage changes with optimized vaccination process. The line increased from the first dose gives the specific information about which should be vaccinated first.

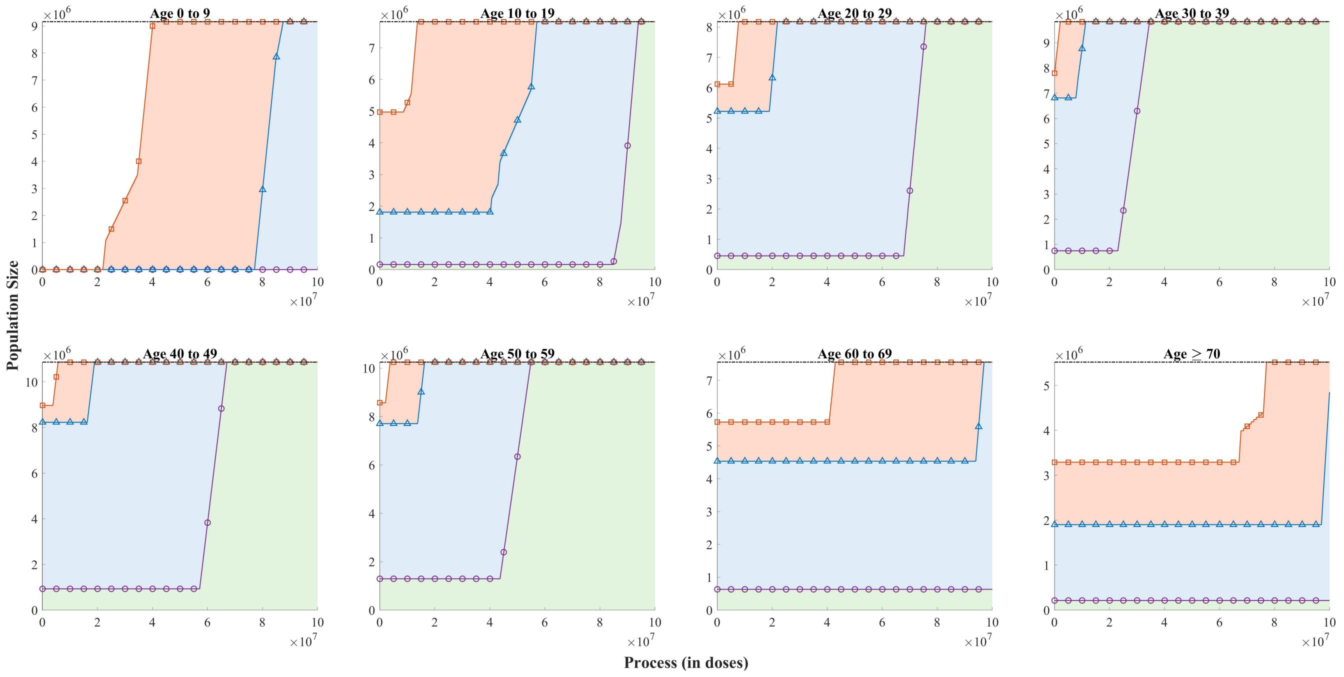

Figure. S13: Optimized Vaccinating Strategy Under Current Contact Pattern and Vaccine Coverage. For minimizing cumulative cases of Delta variant with $R_0 = 3$. 8 subfigures represent the optimal vaccination process in 8 age groups. The x-axis represent the dose-wise vaccination process; y-axis represents vaccine coverage inside the age group (populatioon size of four vaccination status). The purple line with circles denote the population size of booster vaccinated; the blue line with triangles denote the population size of at least fully-vaccinated (including fully vaccinated and booster vaccinated); the red line with squares denote the population size of at least vaccinated (including un-fully vaccinated, fully vaccinated, and booster vaccinated). These lines depicts how coverage changes with optimized vaccination process. The line increased from the first dose gives the specific information about which should be vaccinated first.

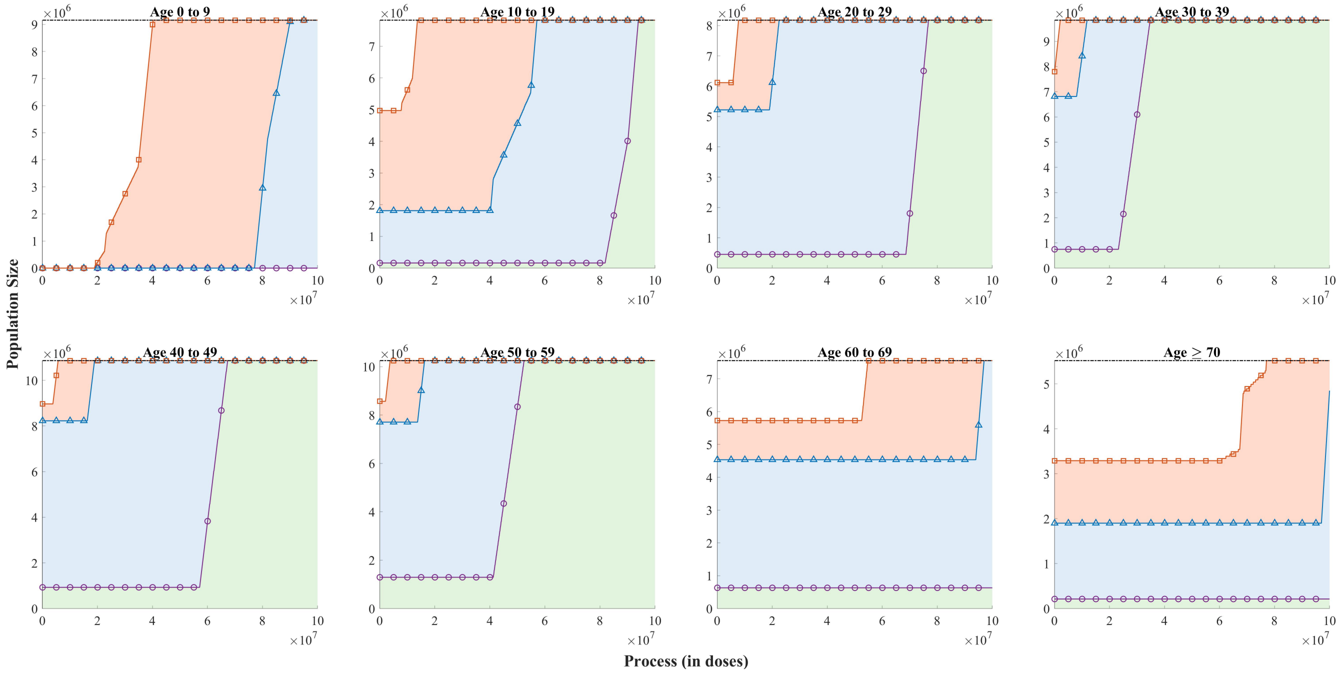

Figure. S14: Optimized Vaccinating Strategy Under Current Contact Pattern and Vaccine Coverage. For minimizing cumulative cases of Delta variant with $R_0 = 4$. 8 subfigures represent the optimal vaccination process in 8 age groups. The x-axis represent the dose-wise vaccination process; y-axis represents vaccine coverage inside the age group (populatioon size of four vaccination status). The purple line with circles denote the population size of booster vaccinated; the blue line with triangles denote the population size of at least fully-vaccinated (including fully vaccinated and booster vaccinated); the red line with squares denote the population size of at least vaccinated (including un-fully vaccinated, fully vaccinated, and booster vaccinated). These lines depicts how coverage changes with optimized vaccination process. The line increased from the first dose gives the specific information about which should be vaccinated first.

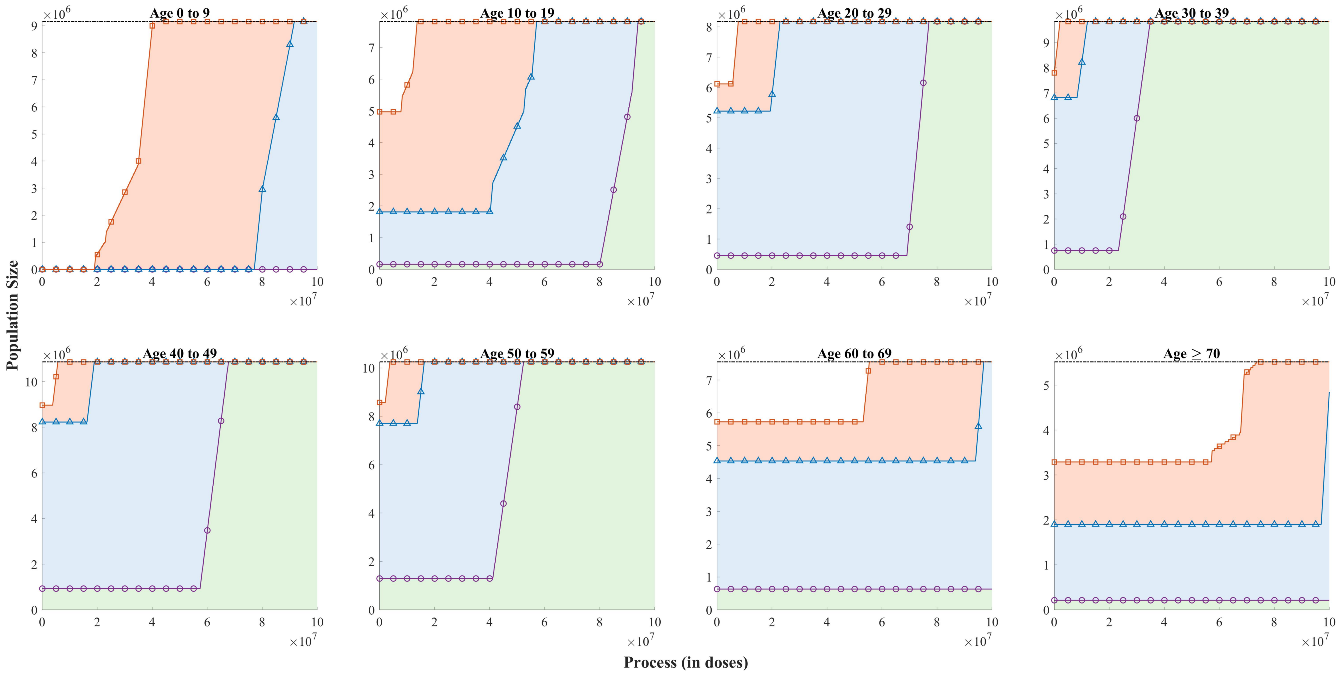

Figure. S15: Optimized Vaccinating Strategy Under Current Contact Pattern and Vaccine Coverage. For minimizing cumulative cases of Delta variant with $R_0 = 5$. 8 subfigures represent the optimal vaccination process in 8 age groups. The x-axis represent the dose-wise vaccination process; y-axis represents vaccine coverage inside the age group (populatioon size of four vaccination status). The purple line with circles denote the population size of booster vaccinated; the blue line with triangles denote the population size of at least fully-vaccinated (including fully vaccinated and booster vaccinated); the red line with squares denote the population size of at least vaccinated (including un-fully vaccinated, fully vaccinated, and booster vaccinated). These lines depicts how coverage changes with optimized vaccination process. The line increased from the first dose gives the specific information about which should be vaccinated first.

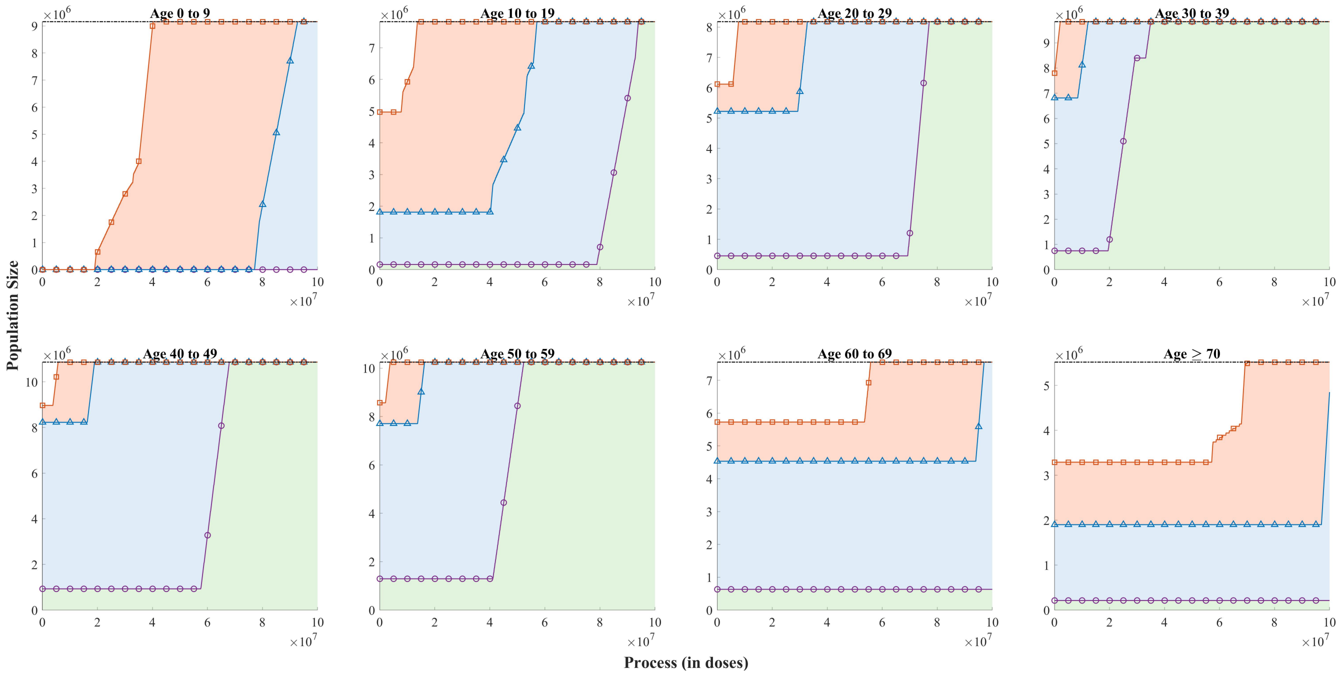

Figure. S16: Optimized Vaccinating Strategy Under Current Contact Pattern and Vaccine Coverage. For minimizing cumulative cases of Delta variant with $R_0 = 6$. 8 subfigures represent the optimal vaccination process in 8 age groups. The x-axis represent the dose-wise vaccination process; y-axis represents vaccine coverage inside the age group (populatioon size of four vaccination status). The purple line with circles denote the population size of booster vaccinated; the blue line with triangles denote the population size of at least fully-vaccinated (including fully vaccinated and booster vaccinated); the red line with squares denote the population size of at least vaccinated (including un-fully vaccinated, fully vaccinated, and booster vaccinated). These lines depicts how coverage changes with optimized vaccination process. The line increased from the first dose gives the specific information about which should be vaccinated first.

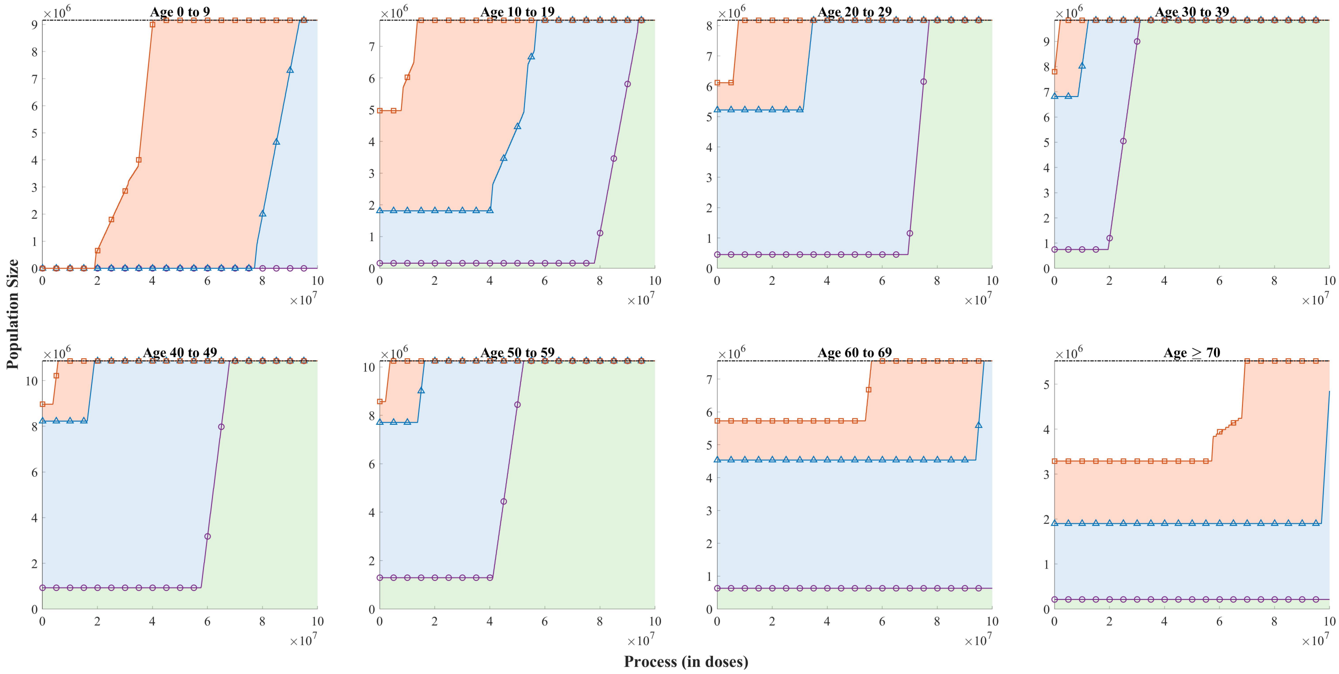

Figure. S17: Optimized Vaccinating Strategy Under Current Contact Pattern and Vaccine Coverage. For minimizing cumulative cases of Delta variant with $R_0 = 7$. 8 subfigures represent the optimal vaccination process in 8 age groups. The x-axis represent the dose-wise vaccination process; y-axis represents vaccine coverage inside the age group (populatioon size of four vaccination status). The purple line with circles denote the population size of booster vaccinated; the blue line with triangles denote the population size of at least fully-vaccinated (including fully vaccinated and booster vaccinated); the red line with squares denote the population size of at least vaccinated (including un-fully vaccinated, fully vaccinated, and booster vaccinated). These lines depicts how coverage changes with optimized vaccination process. The line increased from the first dose gives the specific information about which should be vaccinated first.

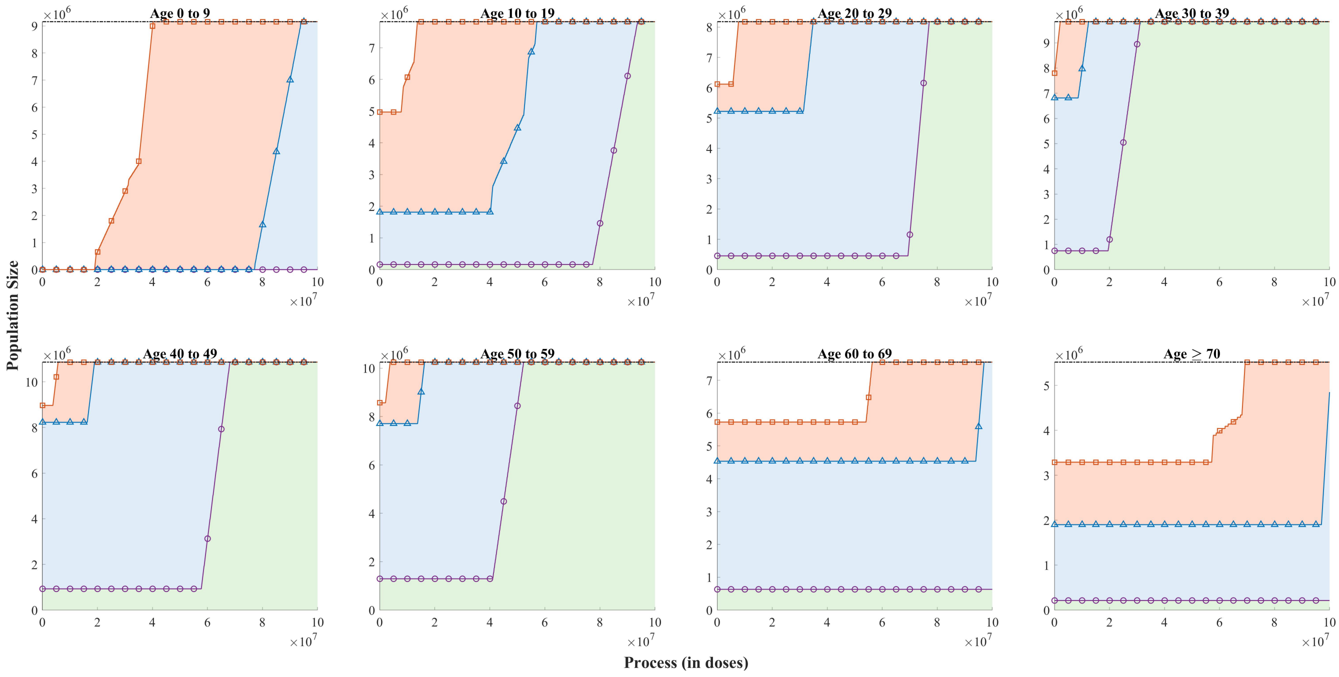

Figure. S18: Optimized Vaccinating Strategy Under Current Contact Pattern and Vaccine Coverage. For minimizing cumulative cases of Delta variant with $R_0 = 8$. 8 subfigures represent the optimal vaccination process in 8 age groups. The x-axis represent the dose-wise vaccination process; y-axis represents vaccine coverage inside the age group (populatioon size of four vaccination status). The purple line with circles denote the population size of booster vaccinated; the blue line with triangles denote the population size of at least fully-vaccinated (including fully vaccinated and booster vaccinated); the red line with squares denote the population size of at least vaccinated (including un-fully vaccinated, fully vaccinated, and booster vaccinated). These lines depicts how coverage changes with optimized vaccination process. The line increased from the first dose gives the specific information about which should be vaccinated first.

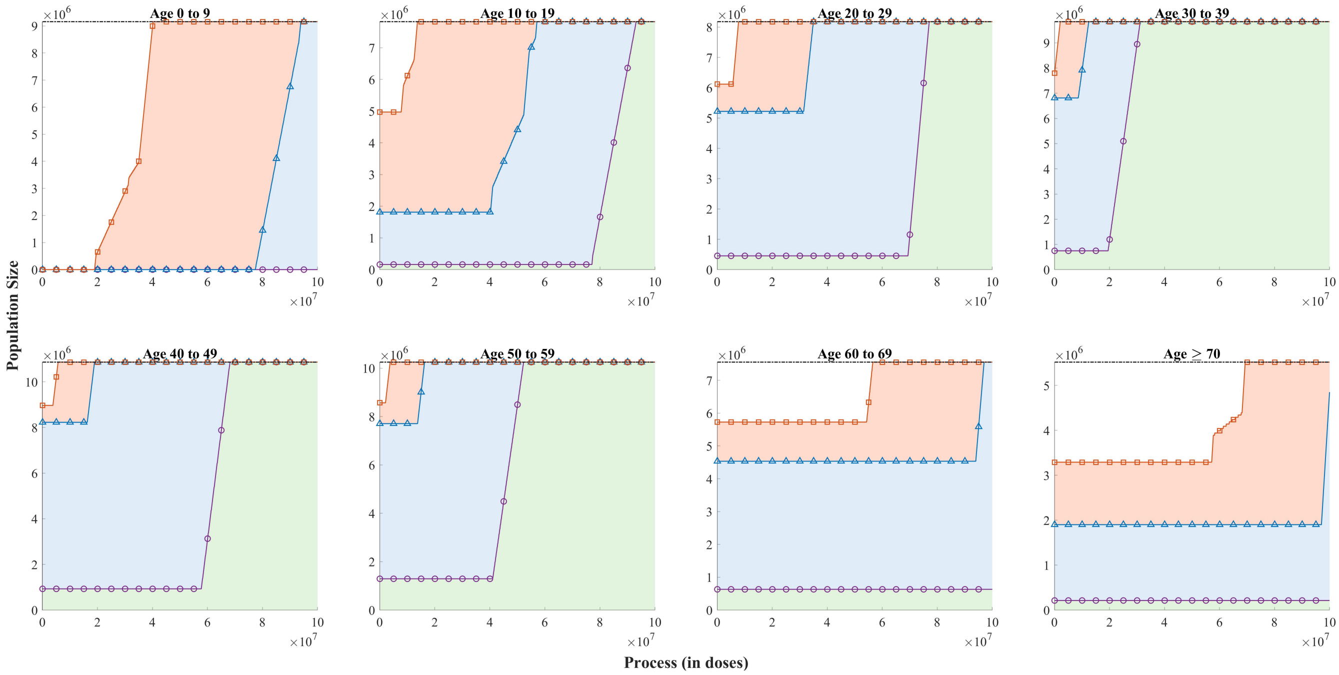

Figure. S19: Optimized Vaccinating Strategy Under Current Contact Pattern and Vaccine Coverage. For minimizing cumulative cases of Delta variant with $R_0 = 9$. 8 subfigures represent the optimal vaccination process in 8 age groups. The x-axis represent the dose-wise vaccination process; y-axis represents vaccine coverage inside the age group (populatioon size of four vaccination status). The purple line with circles denote the population size of booster vaccinated; the blue line with triangles denote the population size of at least fully-vaccinated (including fully vaccinated and booster vaccinated); the red line with squares denote the population size of at least vaccinated (including un-fully vaccinated, fully vaccinated, and booster vaccinated). These lines depicts how coverage changes with optimized vaccination process. The line increased from the first dose gives the specific information about which should be vaccinated first.

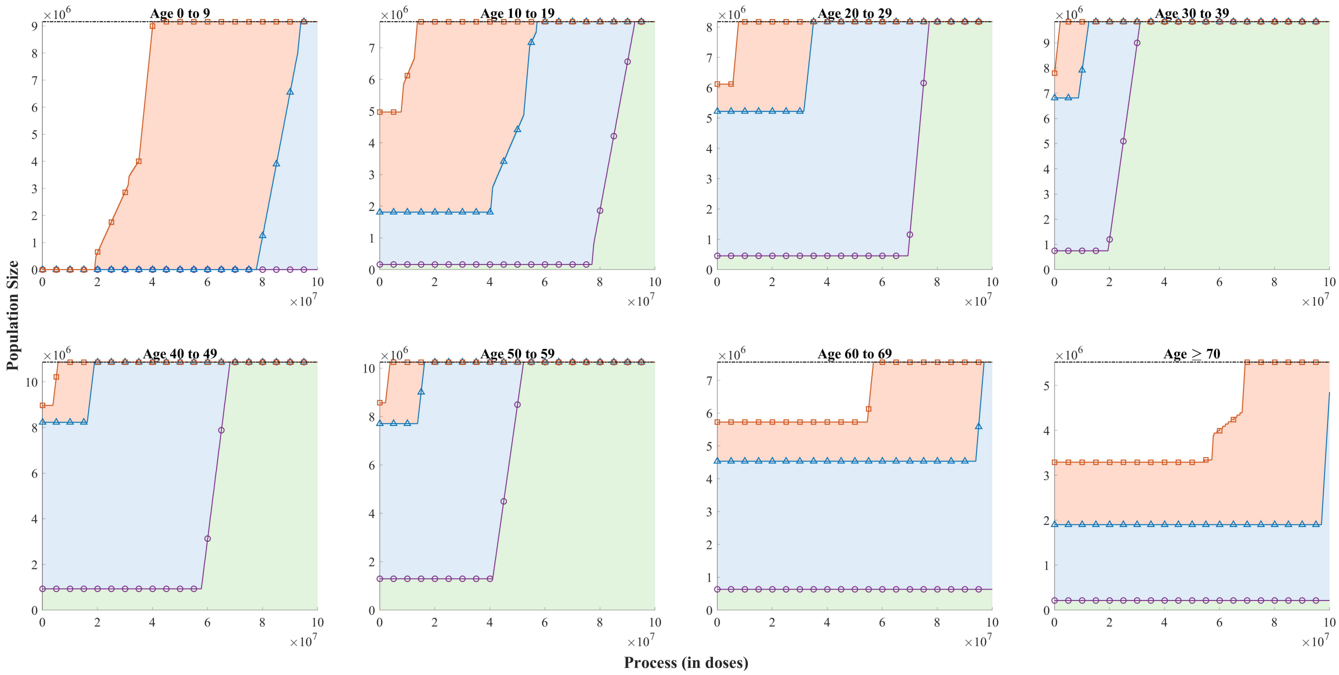

Figure. S20: Optimized Vaccinating Strategy Under Current Contact Pattern and Vaccine Coverage. For minimizing cumulative cases of Delta variant with $R_0 = 10$. 8 subfigures represent the optimal vaccination process in 8 age groups. The x-axis represent the dose-wise vaccination process; y-axis represents vaccine coverage inside the age group (populatioon size of four vaccination status). The purple line with circles denote the population size of booster vaccinated; the blue line with triangles denote the population size of at least fully-vaccinated (including fully vaccinated and booster vaccinated); the red line with squares denote the population size of at least vaccinated (including un-fully vaccinated, fully vaccinated, and booster vaccinated). These lines depicts how coverage changes with optimized vaccination process. The line increased from the first dose gives the specific information about which should be vaccinated first.

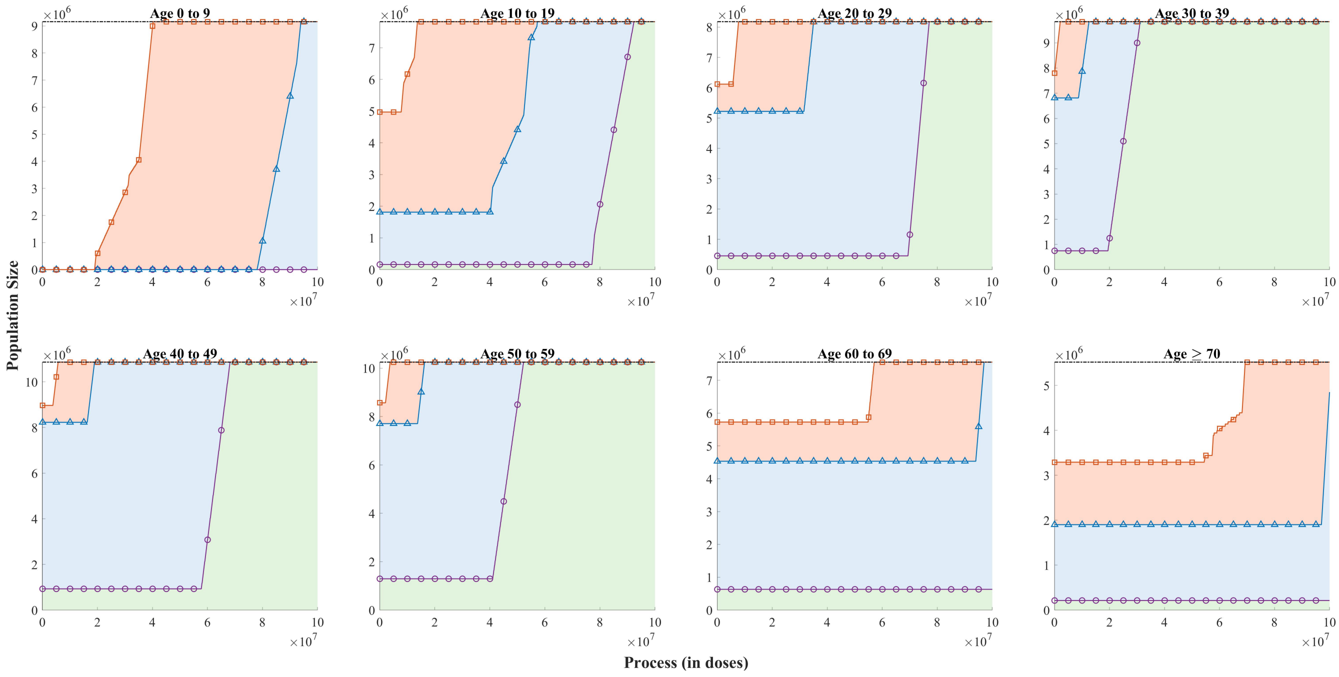

Figure. S21: Optimized Vaccinating Strategy Under Current Contact Pattern and Vaccine Coverage. For minimizing cumulative cases of Delta variant with $R_0 = 11$. 8 subfigures represent the optimal vaccination process in 8 age groups. The x-axis represent the dose-wise vaccination process; y-axis represents vaccine coverage inside the age group (populatioon size of four vaccination status). The purple line with circles denote the population size of booster vaccinated; the blue line with triangles denote the population size of at least fully-vaccinated (including fully vaccinated and booster vaccinated); the red line with squares denote the population size of at least vaccinated (including un-fully vaccinated, fully vaccinated, and booster vaccinated). These lines depicts how coverage changes with optimized vaccination process. The line increased from the first dose gives the specific information about which should be vaccinated first.

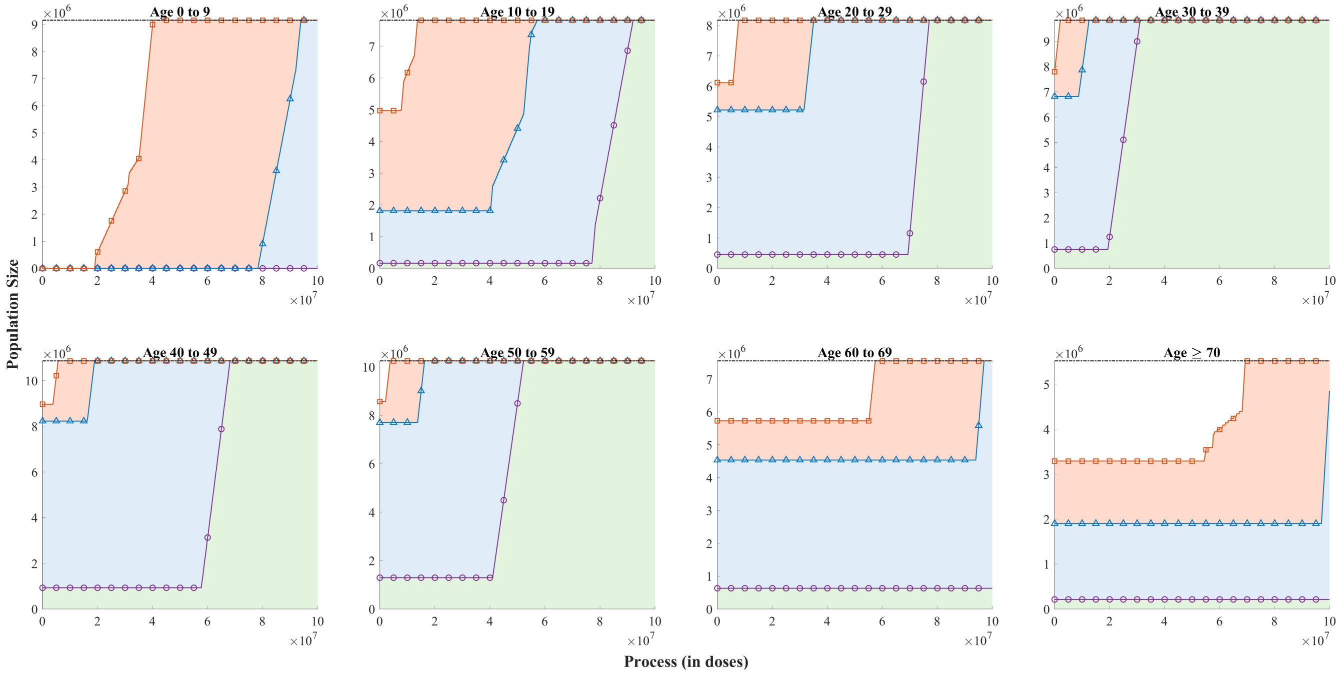

Figure. S22: Optimized Vaccinating Strategy Under Current Contact Pattern and Vaccine Coverage. For minimizing cumulative cases of Delta variant with $R_0 = 12$. 8 subfigures represent the optimal vaccination process in 8 age groups. The x-axis represent the dose-wise vaccination process; y-axis represents vaccine coverage inside the age group (populatioon size of four vaccination status). The purple line with circles denote the population size of booster vaccinated; the blue line with triangles denote the population size of at least fully-vaccinated (including fully vaccinated and booster vaccinated); the red line with squares denote the population size of at least vaccinated (including un-fully vaccinated, fully vaccinated, and booster vaccinated). These lines depicts how coverage changes with optimized vaccination process. The line increased from the first dose gives the specific information about which should be vaccinated first.

With the optimal vaccination process, the cumulative cases in whole population within 14 days from the first illness onset is shown in figure 4.5.1.

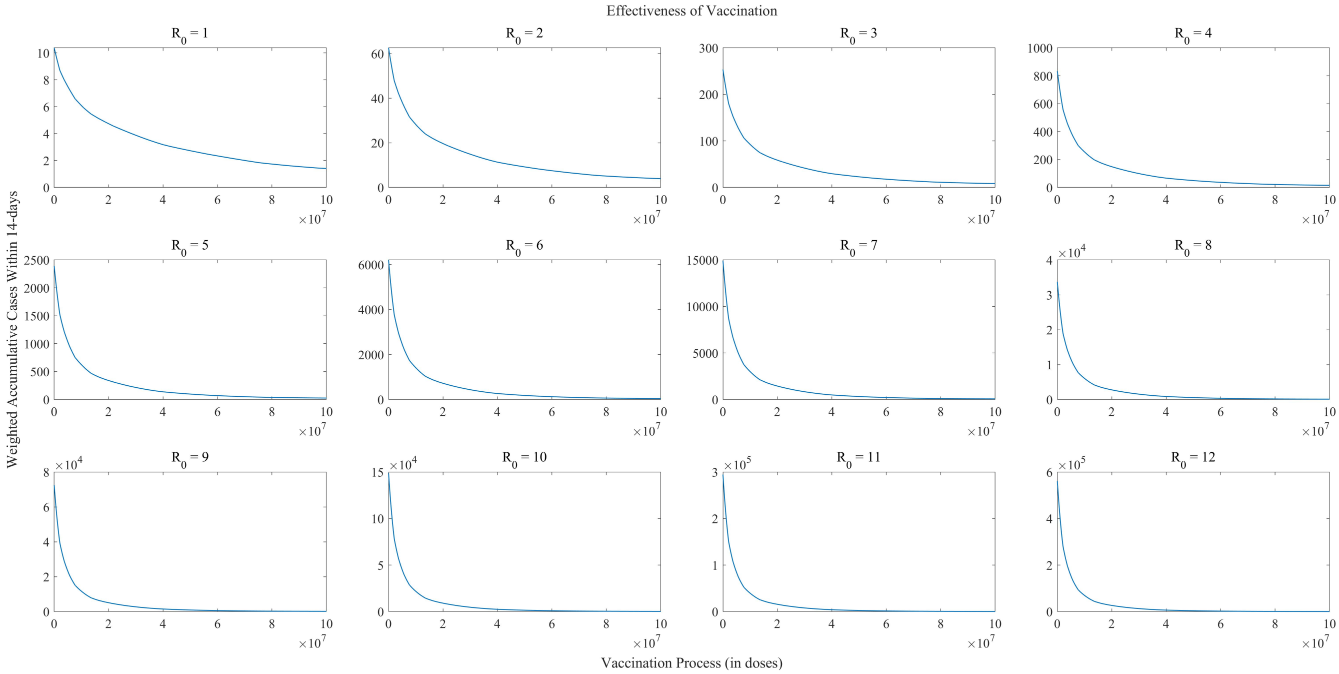

Figure. S23: Effectiveness of Optimized Vaccination. Under Current Contact Pattern and Vaccine Coverage. With parameters of Delta variant.

4.5.2 Hospitalization for Delta Variant

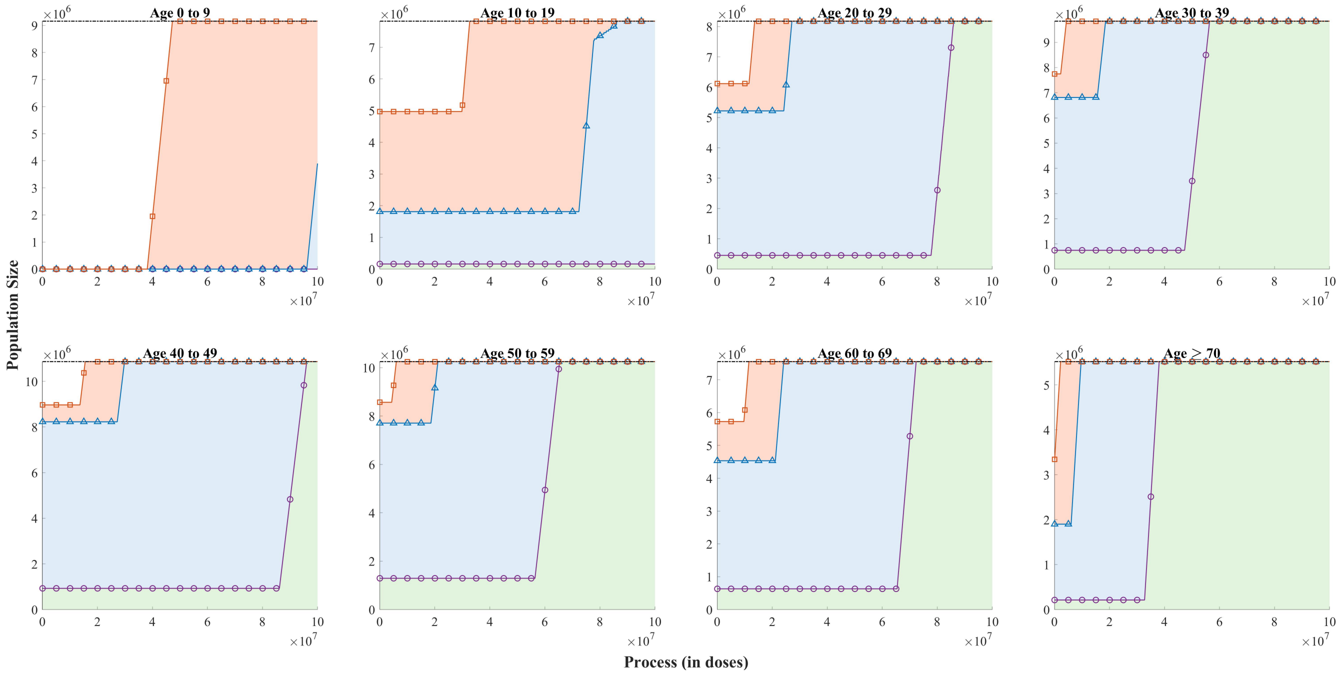

Figure. S24: Optimized Vaccinating Strategy Under Current Contact Pattern and Vaccine Coverage.For minimizing hospitalization of Delta variant with $R_0 = 1$ 8 subfigures represent the optimal vaccination process in 8 age groups. The x-axis represent the dose-wise vaccination process; y-axis represents vaccine coverage inside the age group (populatioon size of four vaccination status). The purple line with circles denote the population size of booster vaccinated; the blue line with triangles denote the population size of at least fully-vaccinated (including fully vaccinated and booster vaccinated); the red line with squares denote the population size of at least vaccinated (including un-fully vaccinated, fully vaccinated, and booster vaccinated). These lines depicts how coverage changes with optimized vaccination process. The line increased from the first dose gives the specific information about which should be vaccinated first.

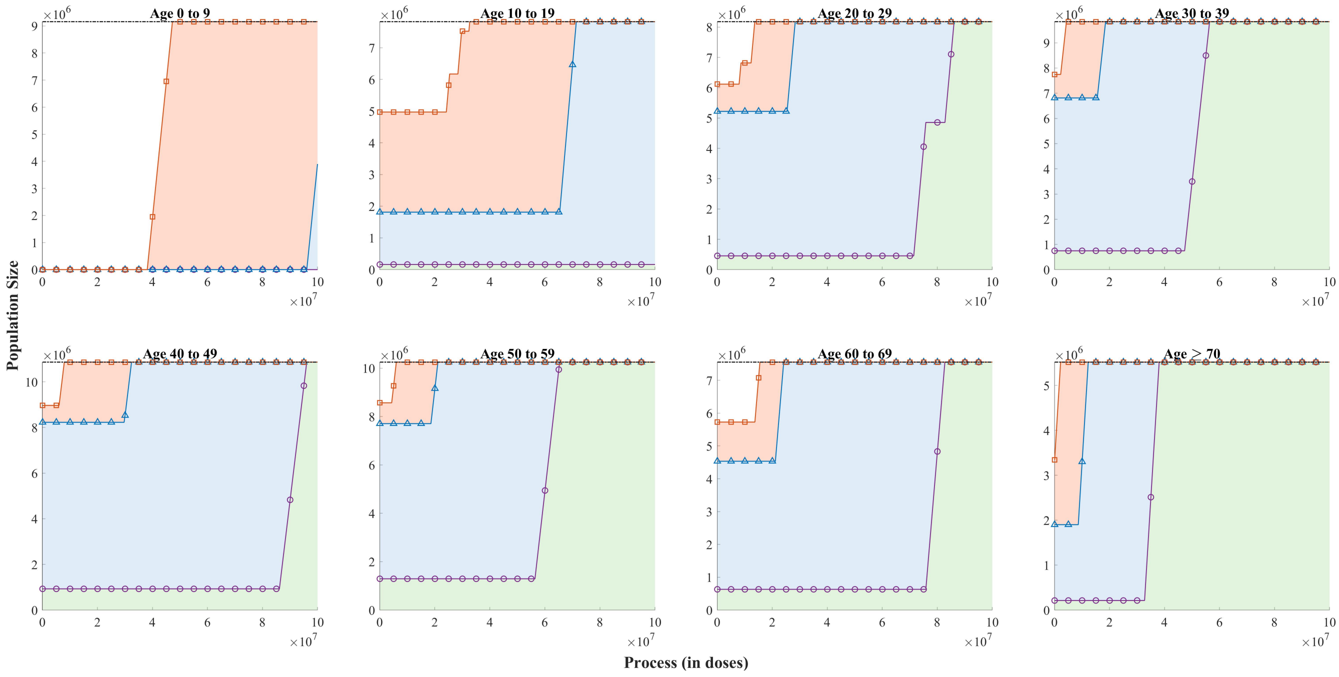

Figure. S25: Optimized Vaccinating Strategy Under Current Contact Pattern and Vaccine Coverage.For minimizing hospitalization of Delta variant with $R_0 = 2$ 8 subfigures represent the optimal vaccination process in 8 age groups. The x-axis represent the dose-wise vaccination process; y-axis represents vaccine coverage inside the age group (populatioon size of four vaccination status). The purple line with circles denote the population size of booster vaccinated; the blue line with triangles denote the population size of at least fully-vaccinated (including fully vaccinated and booster vaccinated); the red line with squares denote the population size of at least vaccinated (including un-fully vaccinated, fully vaccinated, and booster vaccinated). These lines depicts how coverage changes with optimized vaccination process. The line increased from the first dose gives the specific information about which should be vaccinated first.

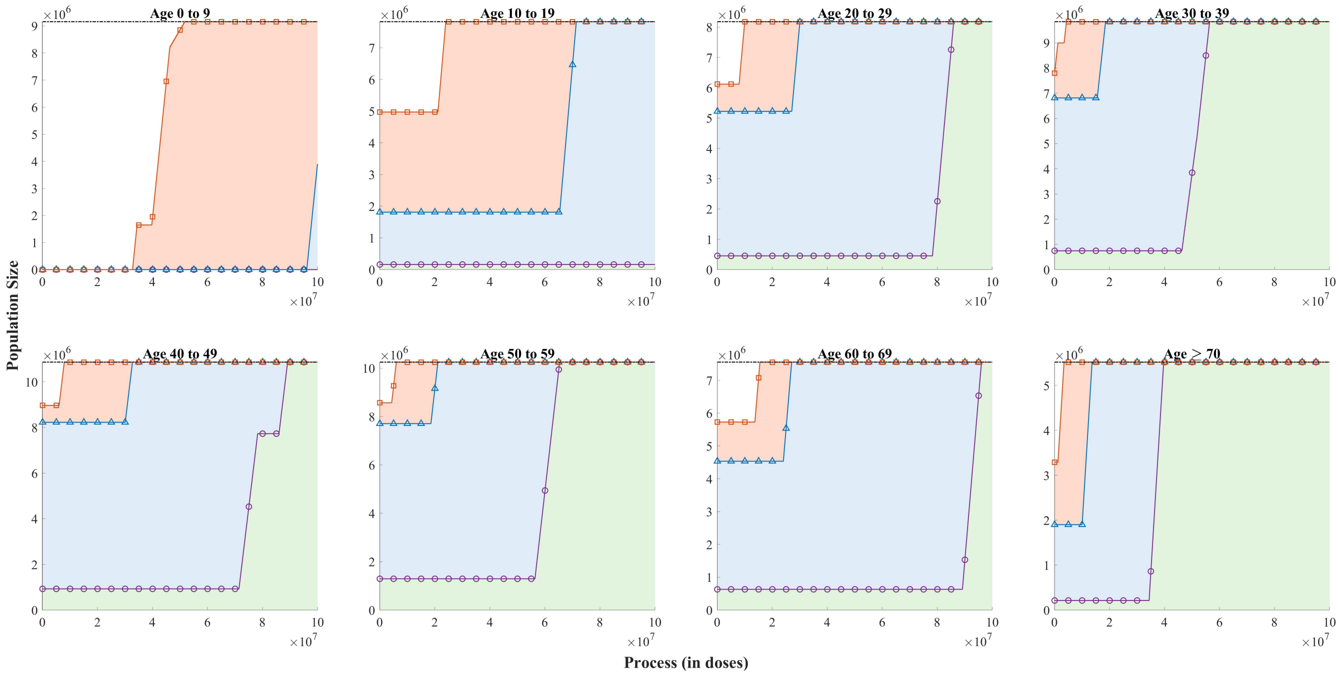

Figure. S26: Optimized Vaccinating Strategy Under Current Contact Pattern and Vaccine Coverage.For minimizing hospitalization of Delta variant with $R_0 = 3$ 8 subfigures represent the optimal vaccination process in 8 age groups. The x-axis represent the dose-wise vaccination process; y-axis represents vaccine coverage inside the age group (populatioon size of four vaccination status). The purple line with circles denote the population size of booster vaccinated; the blue line with triangles denote the population size of at least fully-vaccinated (including fully vaccinated and booster vaccinated); the red line with squares denote the population size of at least vaccinated (including un-fully vaccinated, fully vaccinated, and booster vaccinated). These lines depicts how coverage changes with optimized vaccination process. The line increased from the first dose gives the specific information about which should be vaccinated first.

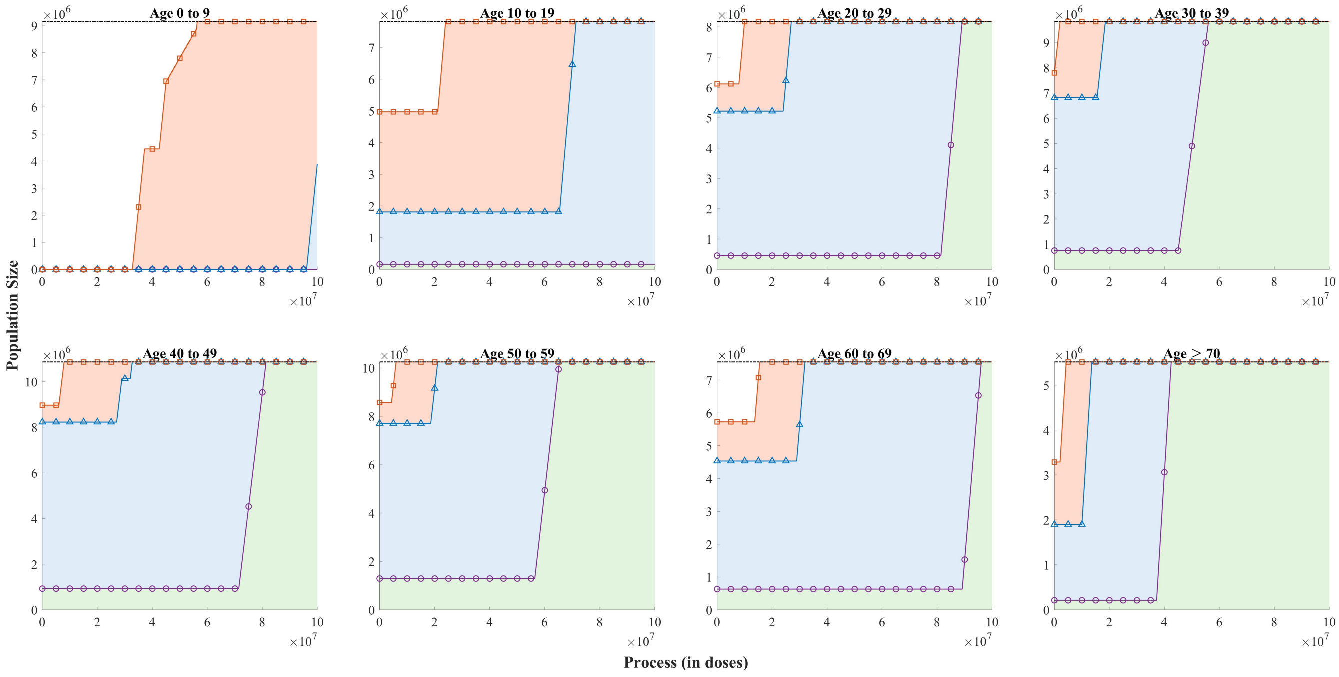

Figure. S27: Optimized Vaccinating Strategy Under Current Contact Pattern and Vaccine Coverage.For minimizing hospitalization of Delta variant with $R_0 = 4$ 8 subfigures represent the optimal vaccination process in 8 age groups. The x-axis represent the dose-wise vaccination process; y-axis represents vaccine coverage inside the age group (populatioon size of four vaccination status). The purple line with circles denote the population size of booster vaccinated; the blue line with triangles denote the population size of at least fully-vaccinated (including fully vaccinated and booster vaccinated); the red line with squares denote the population size of at least vaccinated (including un-fully vaccinated, fully vaccinated, and booster vaccinated). These lines depicts how coverage changes with optimized vaccination process. The line increased from the first dose gives the specific information about which should be vaccinated first.

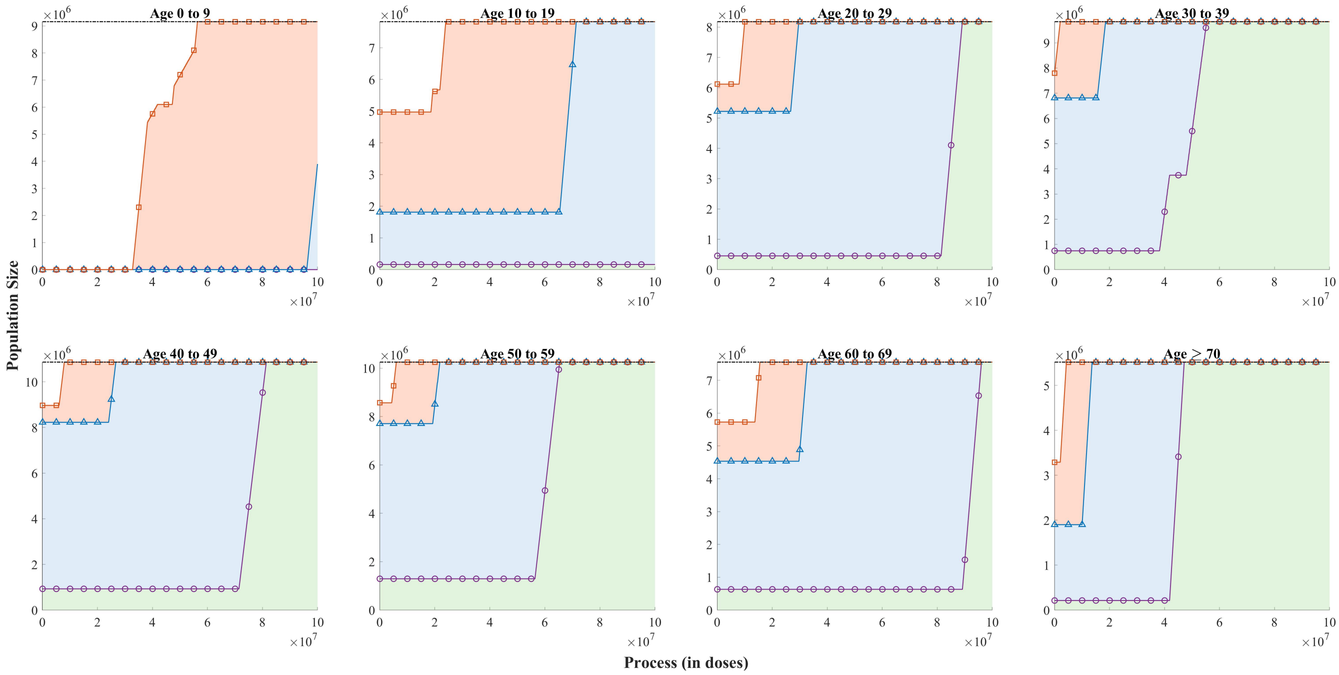

Figure. S28: Optimized Vaccinating Strategy Under Current Contact Pattern and Vaccine Coverage.For minimizing hospitalization of Delta variant with $R_0 = 5$ 8 subfigures represent the optimal vaccination process in 8 age groups. The x-axis represent the dose-wise vaccination process; y-axis represents vaccine coverage inside the age group (populatioon size of four vaccination status). The purple line with circles denote the population size of booster vaccinated; the blue line with triangles denote the population size of at least fully-vaccinated (including fully vaccinated and booster vaccinated); the red line with squares denote the population size of at least vaccinated (including un-fully vaccinated, fully vaccinated, and booster vaccinated). These lines depicts how coverage changes with optimized vaccination process. The line increased from the first dose gives the specific information about which should be vaccinated first.

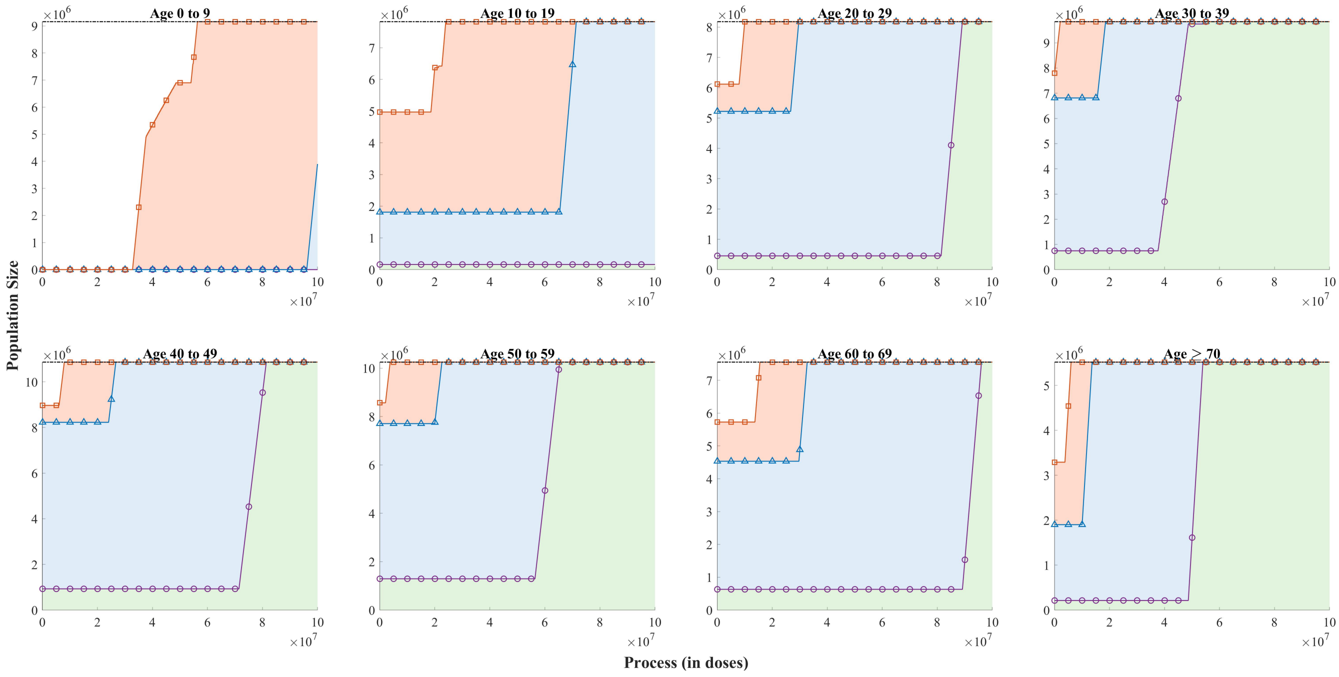

Figure. S29: Optimized Vaccinating Strategy Under Current Contact Pattern and Vaccine Coverage.For minimizing hospitalization of Delta variant with $R_0 = 6$ 8 subfigures represent the optimal vaccination process in 8 age groups. The x-axis represent the dose-wise vaccination process; y-axis represents vaccine coverage inside the age group (populatioon size of four vaccination status). The purple line with circles denote the population size of booster vaccinated; the blue line with triangles denote the population size of at least fully-vaccinated (including fully vaccinated and booster vaccinated); the red line with squares denote the population size of at least vaccinated (including un-fully vaccinated, fully vaccinated, and booster vaccinated). These lines depicts how coverage changes with optimized vaccination process. The line increased from the first dose gives the specific information about which should be vaccinated first.

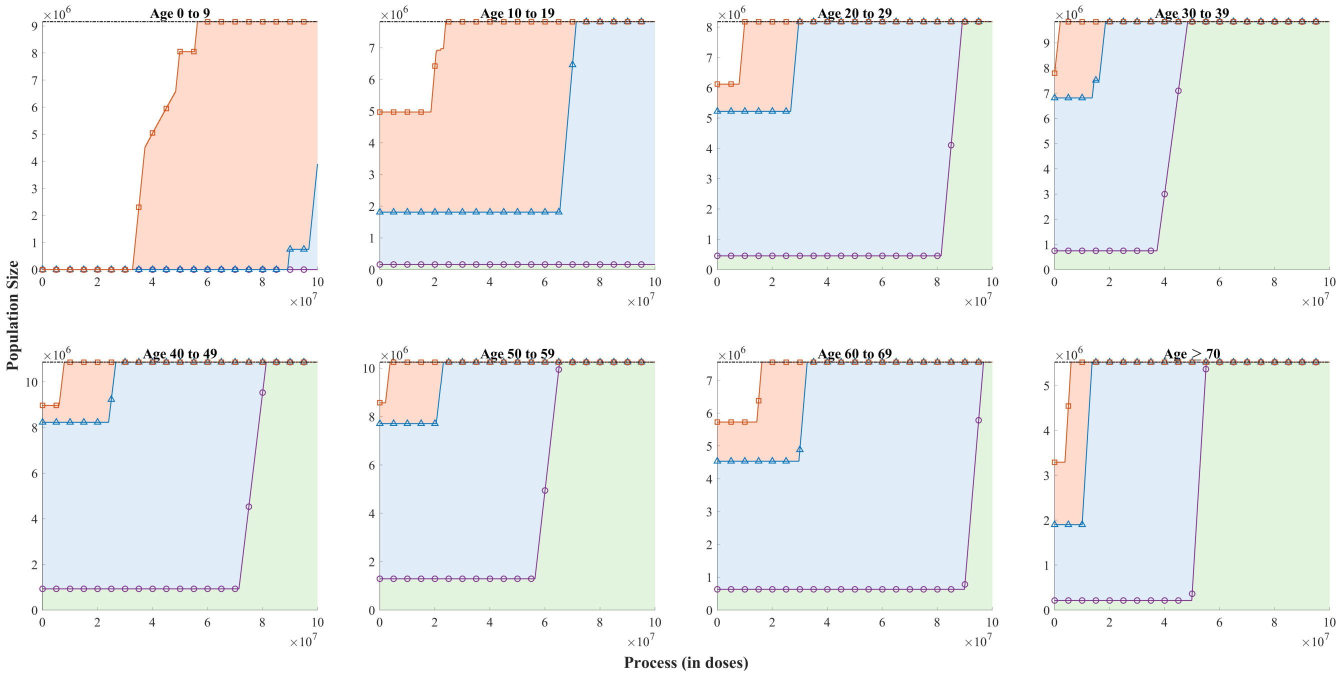

Figure. S30: Optimized Vaccinating Strategy Under Current Contact Pattern and Vaccine Coverage.For minimizing hospitalization of Delta variant with $R_0 = 7$ 8 subfigures represent the optimal vaccination process in 8 age groups. The x-axis represent the dose-wise vaccination process; y-axis represents vaccine coverage inside the age group (populatioon size of four vaccination status). The purple line with circles denote the population size of booster vaccinated; the blue line with triangles denote the population size of at least fully-vaccinated (including fully vaccinated and booster vaccinated); the red line with squares denote the population size of at least vaccinated (including un-fully vaccinated, fully vaccinated, and booster vaccinated). These lines depicts how coverage changes with optimized vaccination process. The line increased from the first dose gives the specific information about which should be vaccinated first.

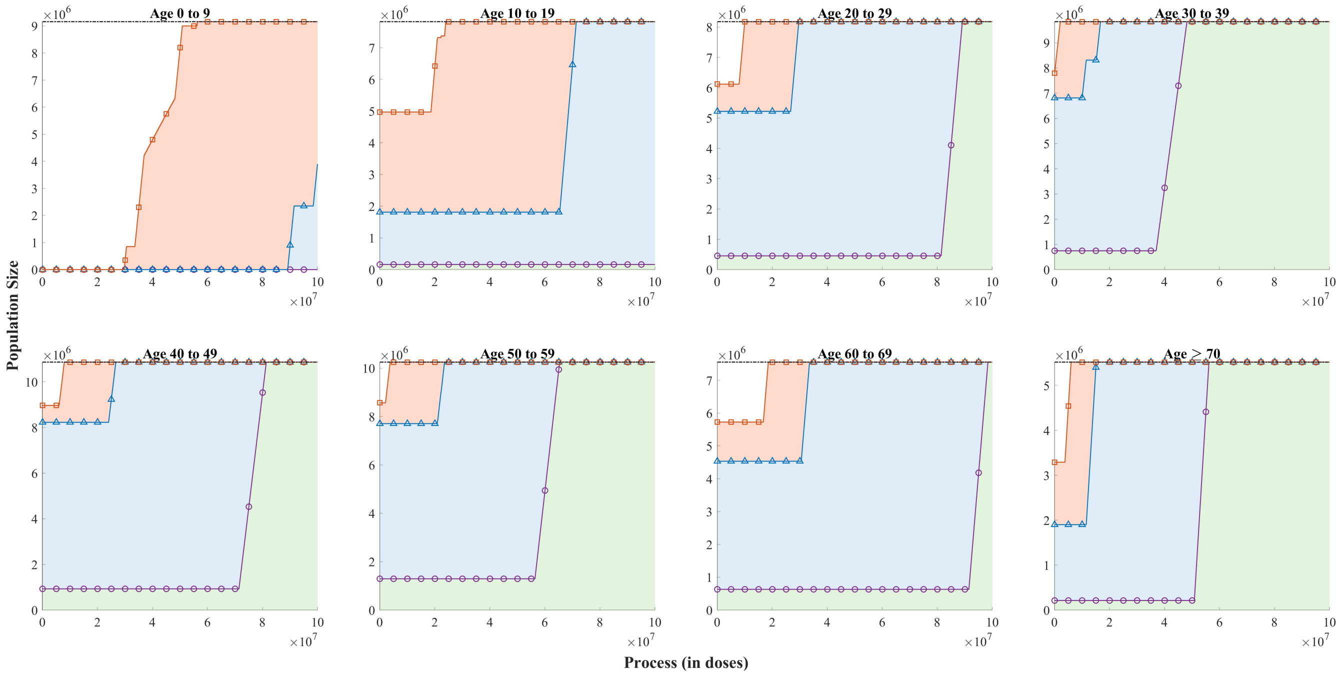

Figure. S31: Optimized Vaccinating Strategy Under Current Contact Pattern and Vaccine Coverage.For minimizing hospitalization of Delta variant with $R_0 = 8$ 8 subfigures represent the optimal vaccination process in 8 age groups. The x-axis represent the dose-wise vaccination process; y-axis represents vaccine coverage inside the age group (populatioon size of four vaccination status). The purple line with circles denote the population size of booster vaccinated; the blue line with triangles denote the population size of at least fully-vaccinated (including fully vaccinated and booster vaccinated); the red line with squares denote the population size of at least vaccinated (including un-fully vaccinated, fully vaccinated, and booster vaccinated). These lines depicts how coverage changes with optimized vaccination process. The line increased from the first dose gives the specific information about which should be vaccinated first.

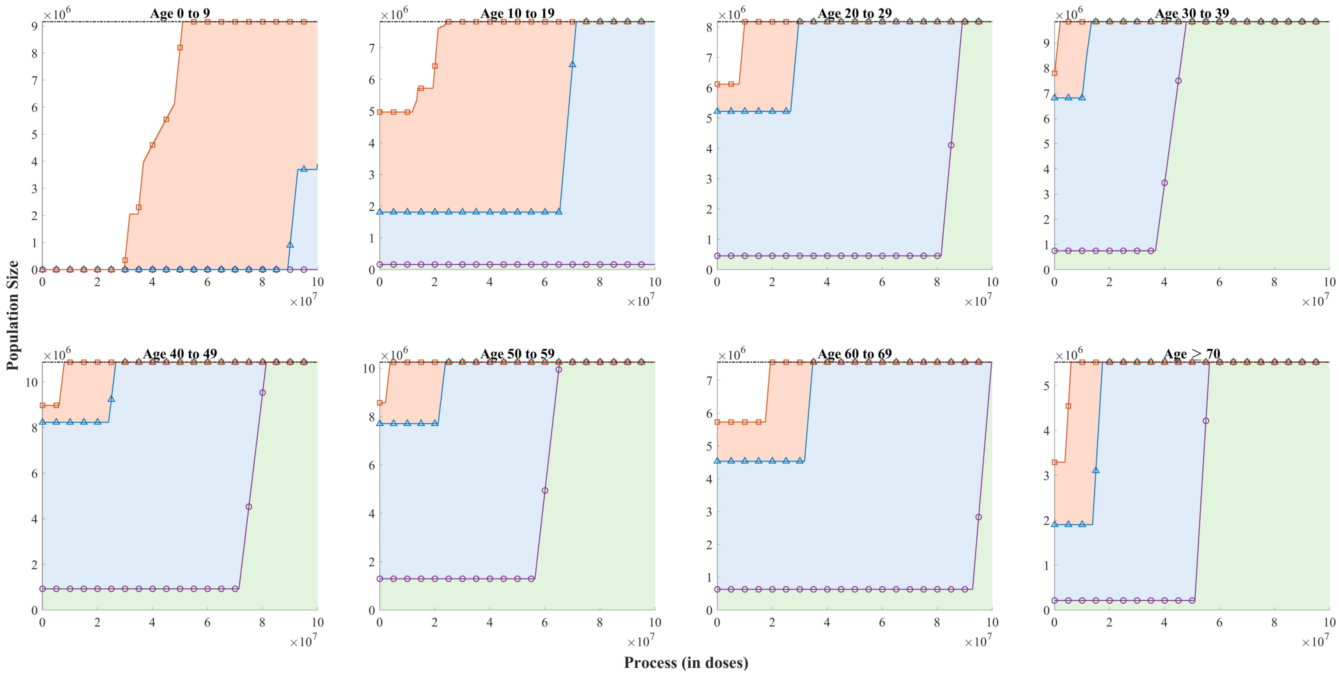

Figure. S32: Optimized Vaccinating Strategy Under Current Contact Pattern and Vaccine Coverage.For minimizing hospitalization of Delta variant with $R_0 = 9$ 8 subfigures represent the optimal vaccination process in 8 age groups. The x-axis represent the dose-wise vaccination process; y-axis represents vaccine coverage inside the age group (populatioon size of four vaccination status). The purple line with circles denote the population size of booster vaccinated; the blue line with triangles denote the population size of at least fully-vaccinated (including fully vaccinated and booster vaccinated); the red line with squares denote the population size of at least vaccinated (including un-fully vaccinated, fully vaccinated, and booster vaccinated). These lines depicts how coverage changes with optimized vaccination process. The line increased from the first dose gives the specific information about which should be vaccinated first.

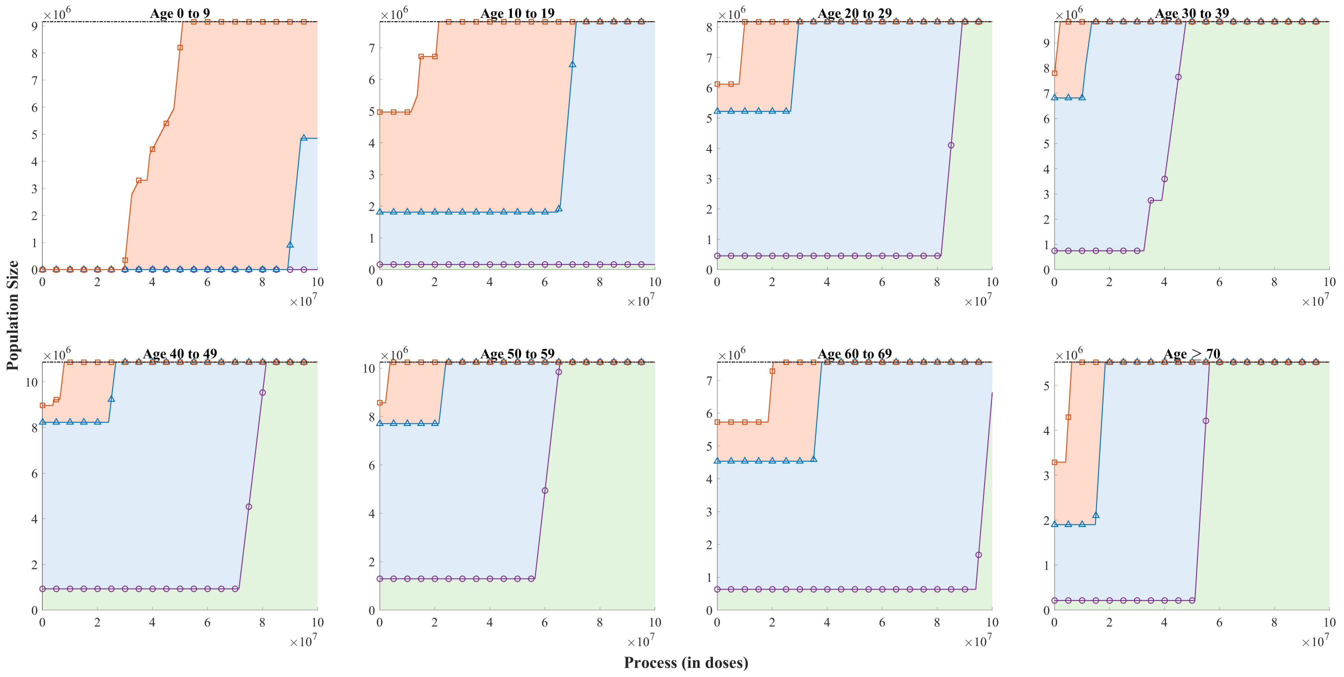

Figure. S33: Optimized Vaccinating Strategy Under Current Contact Pattern and Vaccine Coverage.For minimizing hospitalization of Delta variant with $R_0 = 10$ 8 subfigures represent the optimal vaccination process in 8 age groups. The x-axis represent the dose-wise vaccination process; y-axis represents vaccine coverage inside the age group (populatioon size of four vaccination status). The purple line with circles denote the population size of booster vaccinated; the blue line with triangles denote the population size of at least fully-vaccinated (including fully vaccinated and booster vaccinated); the red line with squares denote the population size of at least vaccinated (including un-fully vaccinated, fully vaccinated, and booster vaccinated). These lines depicts how coverage changes with optimized vaccination process. The line increased from the first dose gives the specific information about which should be vaccinated first.

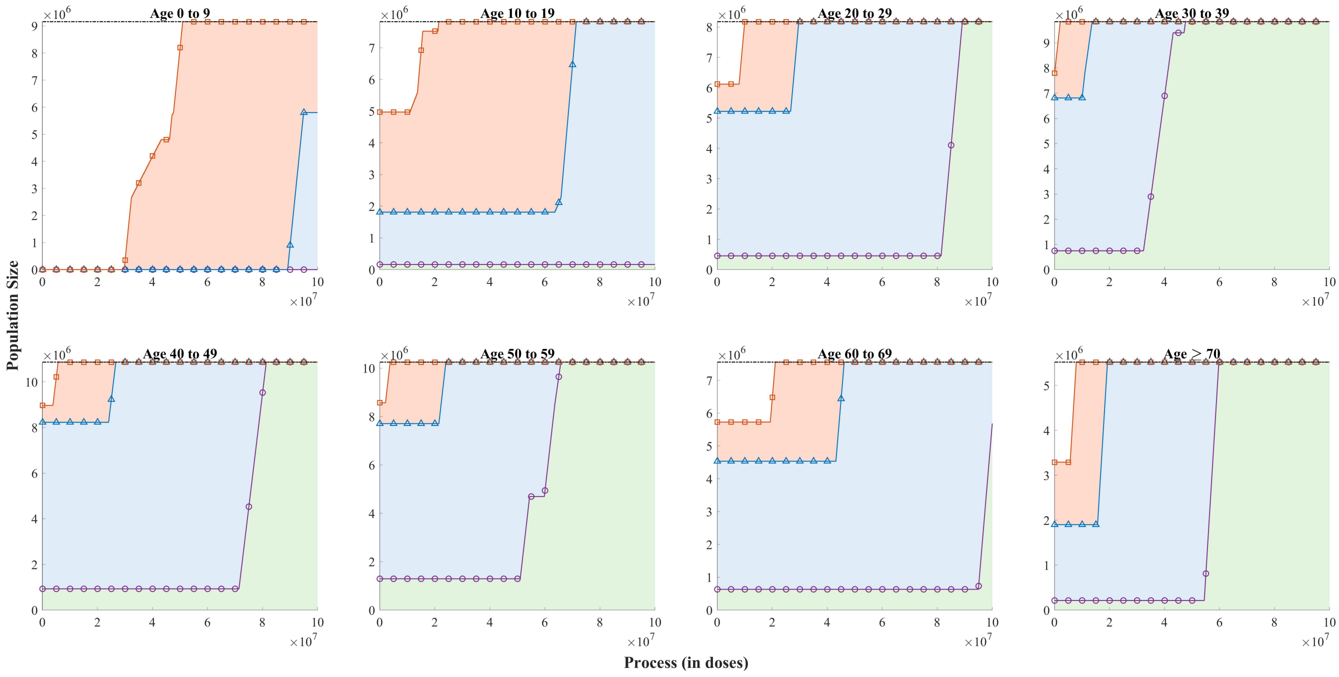

Figure. S34: Optimized Vaccinating Strategy Under Current Contact Pattern and Vaccine Coverage.For minimizing hospitalization of Delta variant with $R_0 = 11$ 8 subfigures represent the optimal vaccination process in 8 age groups. The x-axis represent the dose-wise vaccination process; y-axis represents vaccine coverage inside the age group (populatioon size of four vaccination status). The purple line with circles denote the population size of booster vaccinated; the blue line with triangles denote the population size of at least fully-vaccinated (including fully vaccinated and booster vaccinated); the red line with squares denote the population size of at least vaccinated (including un-fully vaccinated, fully vaccinated, and booster vaccinated). These lines depicts how coverage changes with optimized vaccination process. The line increased from the first dose gives the specific information about which should be vaccinated first.

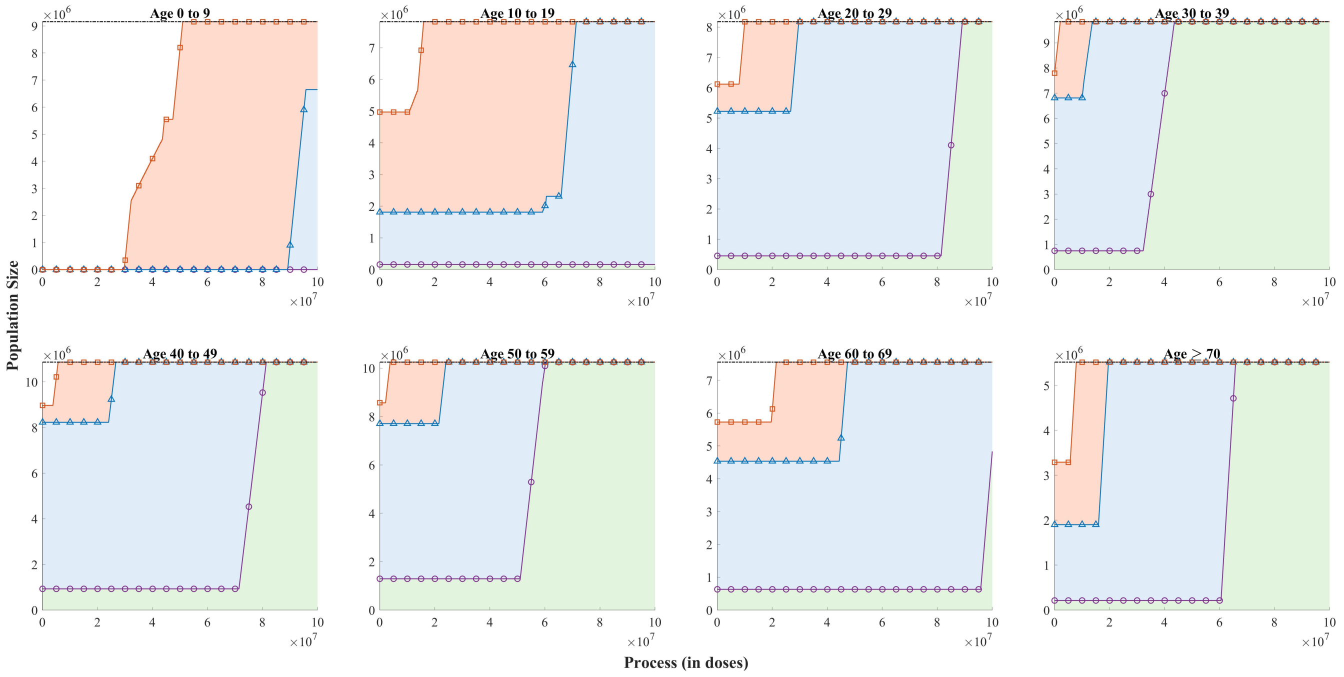

Figure. S35: Optimized Vaccinating Strategy Under Current Contact Pattern and Vaccine Coverage.For minimizing hospitalization of Delta variant with $R_0 = 12$ 8 subfigures represent the optimal vaccination process in 8 age groups. The x-axis represent the dose-wise vaccination process; y-axis represents vaccine coverage inside the age group (populatioon size of four vaccination status). The purple line with circles denote the population size of booster vaccinated; the blue line with triangles denote the population size of at least fully-vaccinated (including fully vaccinated and booster vaccinated); the red line with squares denote the population size of at least vaccinated (including un-fully vaccinated, fully vaccinated, and booster vaccinated). These lines depicts how coverage changes with optimized vaccination process. The line increased from the first dose gives the specific information about which should be vaccinated first.

With the optimal vaccination process, the hospitalization in whole population within 14 days from the first illness onset is shown in figure 4.5.1.

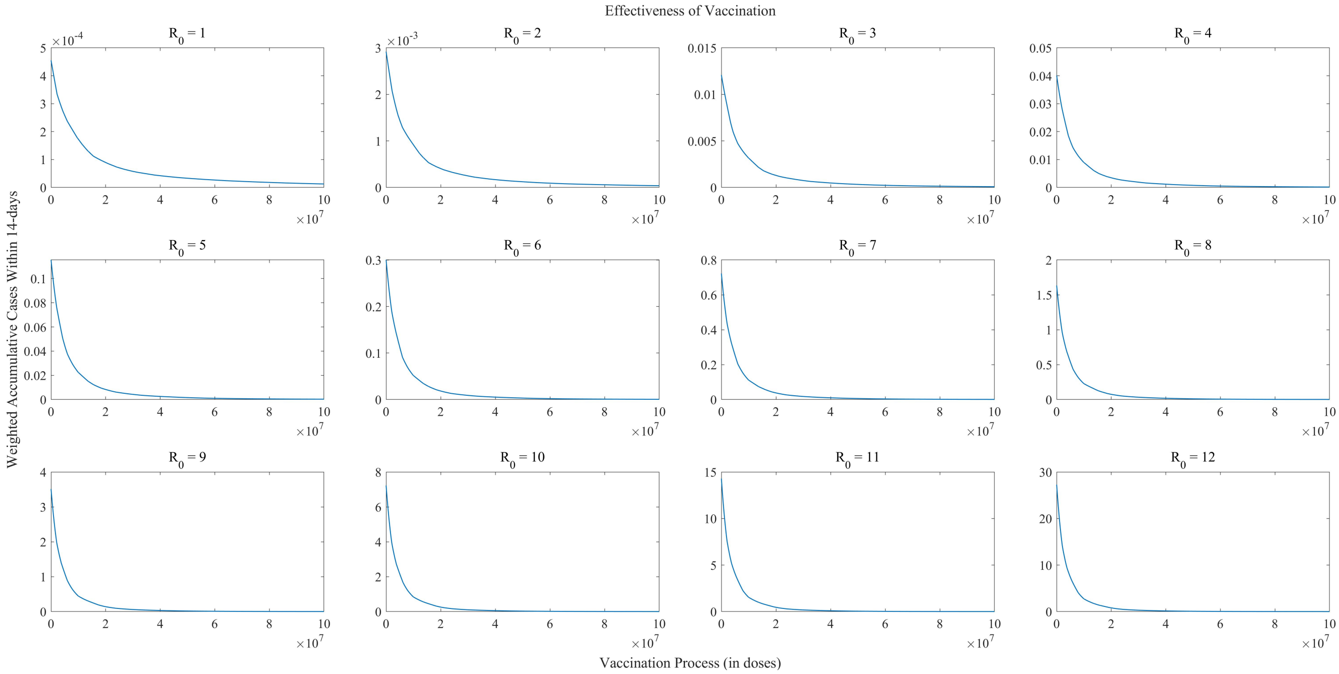

Figure. S36: Effectiveness of Optimized Vaccination. Under Current Contact Pattern and Vaccine Coverage. With parameters of Delta variant.

4.5.3 Fatality for Delta Variant

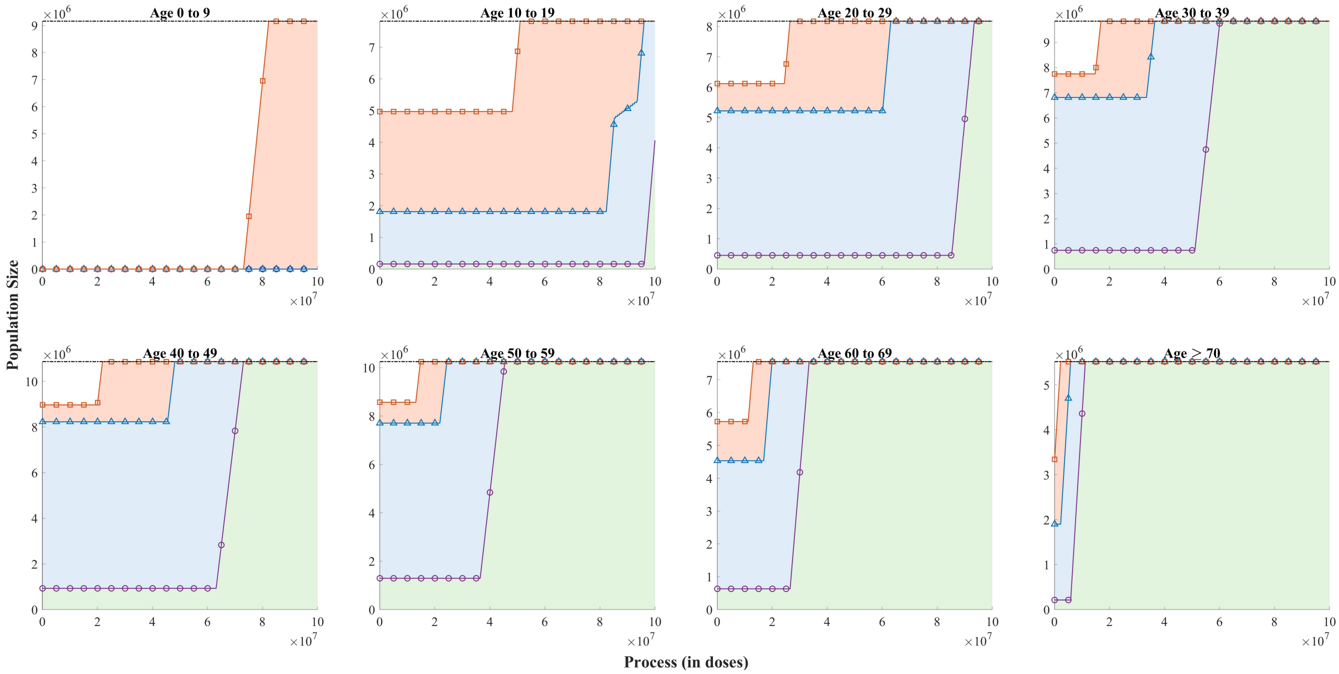

Figure. S37: Optimized Vaccinating Strategy Under Current Contact Pattern and Vaccine Coverage. For minimizing fatality of Delta variant with $R_0 = 1$. 8 subfigures represent the optimal vaccination process in 8 age groups. The x-axis represent the dose-wise vaccination process; y-axis represents vaccine coverage inside the age group (populatioon size of four vaccination status). The purple line with circles denote the population size of booster vaccinated; the blue line with triangles denote the population size of at least fully-vaccinated (including fully vaccinated and booster vaccinated); the red line with squares denote the population size of at least vaccinated (including un-fully vaccinated, fully vaccinated, and booster vaccinated). These lines depicts how coverage changes with optimized vaccination process. The line increased from the first dose gives the specific information about which should be vaccinated first.

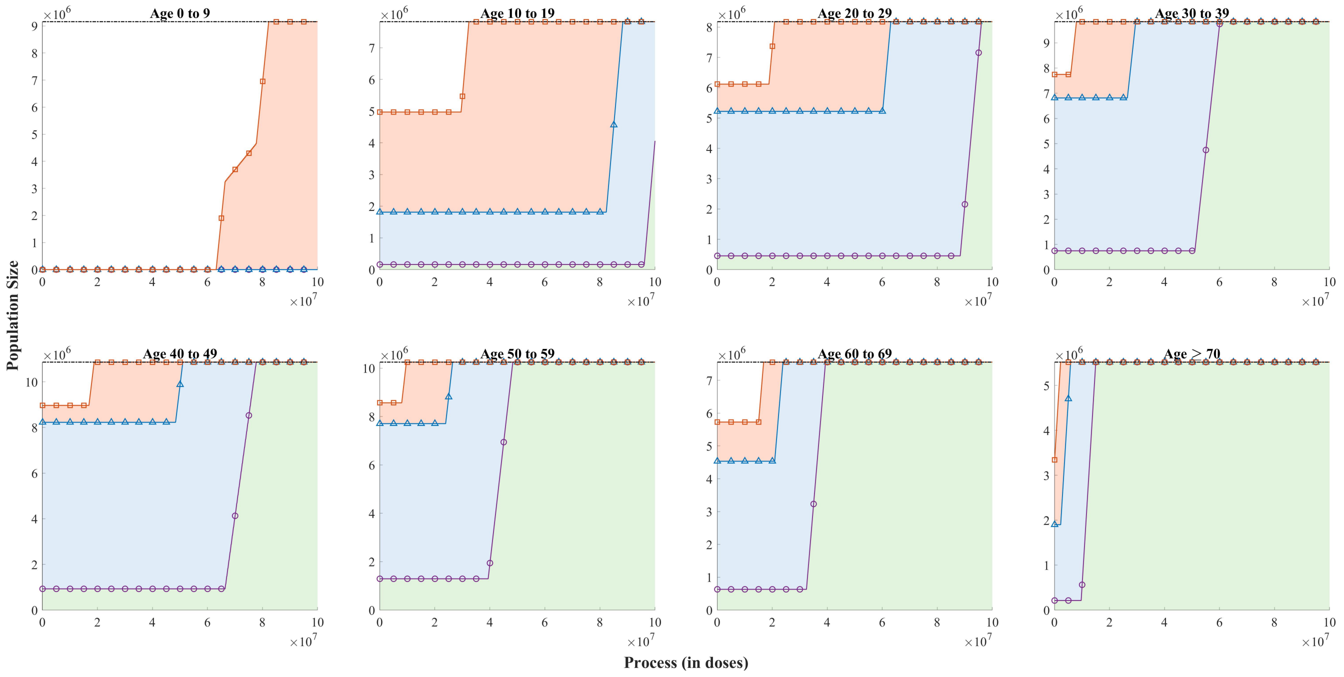

Figure. S38: Optimized Vaccinating Strategy Under Current Contact Pattern and Vaccine Coverage. For minimizing fatality of Delta variant with $R_0 = 2$. 8 subfigures represent the optimal vaccination process in 8 age groups. The x-axis represent the dose-wise vaccination process; y-axis represents vaccine coverage inside the age group (populatioon size of four vaccination status). The purple line with circles denote the population size of booster vaccinated; the blue line with triangles denote the population size of at least fully-vaccinated (including fully vaccinated and booster vaccinated); the red line with squares denote the population size of at least vaccinated (including un-fully vaccinated, fully vaccinated, and booster vaccinated). These lines depicts how coverage changes with optimized vaccination process. The line increased from the first dose gives the specific information about which should be vaccinated first.

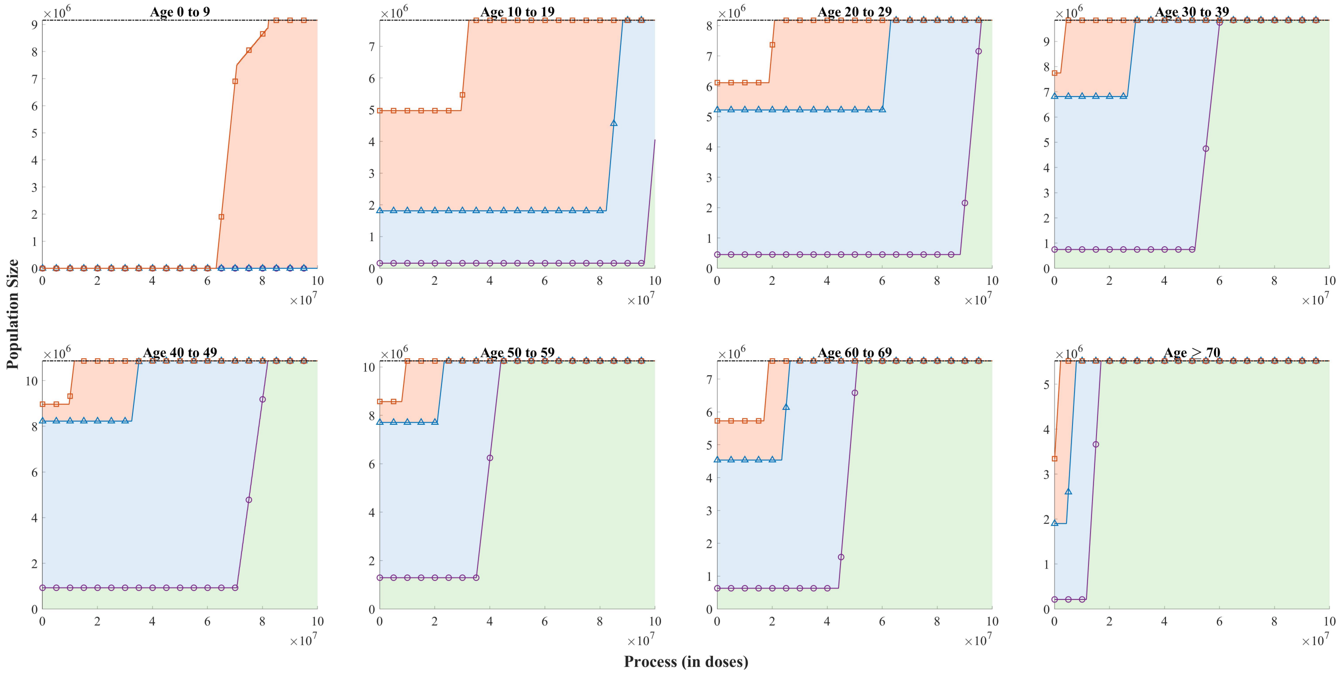

Figure. S39: Optimized Vaccinating Strategy Under Current Contact Pattern and Vaccine Coverage. For minimizing fatality of Delta variant with $R_0 = 3$. 8 subfigures represent the optimal vaccination process in 8 age groups. The x-axis represent the dose-wise vaccination process; y-axis represents vaccine coverage inside the age group (populatioon size of four vaccination status). The purple line with circles denote the population size of booster vaccinated; the blue line with triangles denote the population size of at least fully-vaccinated (including fully vaccinated and booster vaccinated); the red line with squares denote the population size of at least vaccinated (including un-fully vaccinated, fully vaccinated, and booster vaccinated). These lines depicts how coverage changes with optimized vaccination process. The line increased from the first dose gives the specific information about which should be vaccinated first.

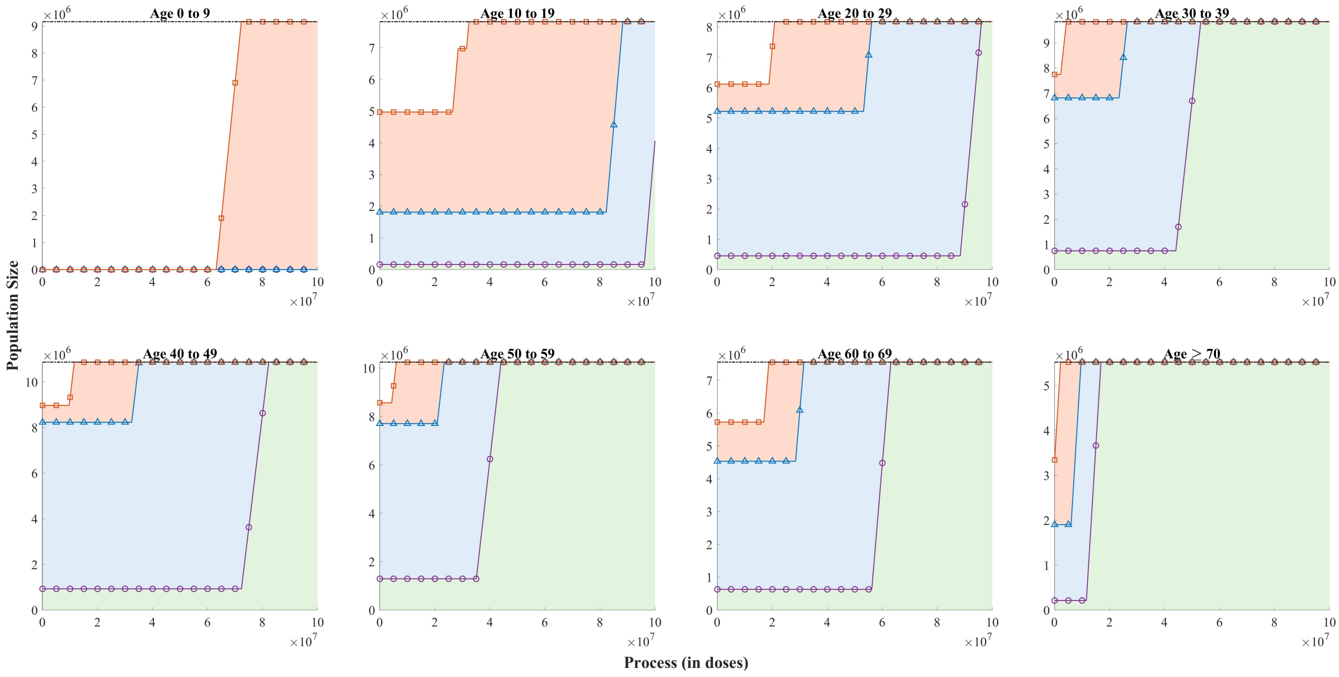

Figure. S40: Optimized Vaccinating Strategy Under Current Contact Pattern and Vaccine Coverage. For minimizing fatality of Delta variant with $R_0 = 4$. 8 subfigures represent the optimal vaccination process in 8 age groups. The x-axis represent the dose-wise vaccination process; y-axis represents vaccine coverage inside the age group (populatioon size of four vaccination status). The purple line with circles denote the population size of booster vaccinated; the blue line with triangles denote the population size of at least fully-vaccinated (including fully vaccinated and booster vaccinated); the red line with squares denote the population size of at least vaccinated (including un-fully vaccinated, fully vaccinated, and booster vaccinated). These lines depicts how coverage changes with optimized vaccination process. The line increased from the first dose gives the specific information about which should be vaccinated first.

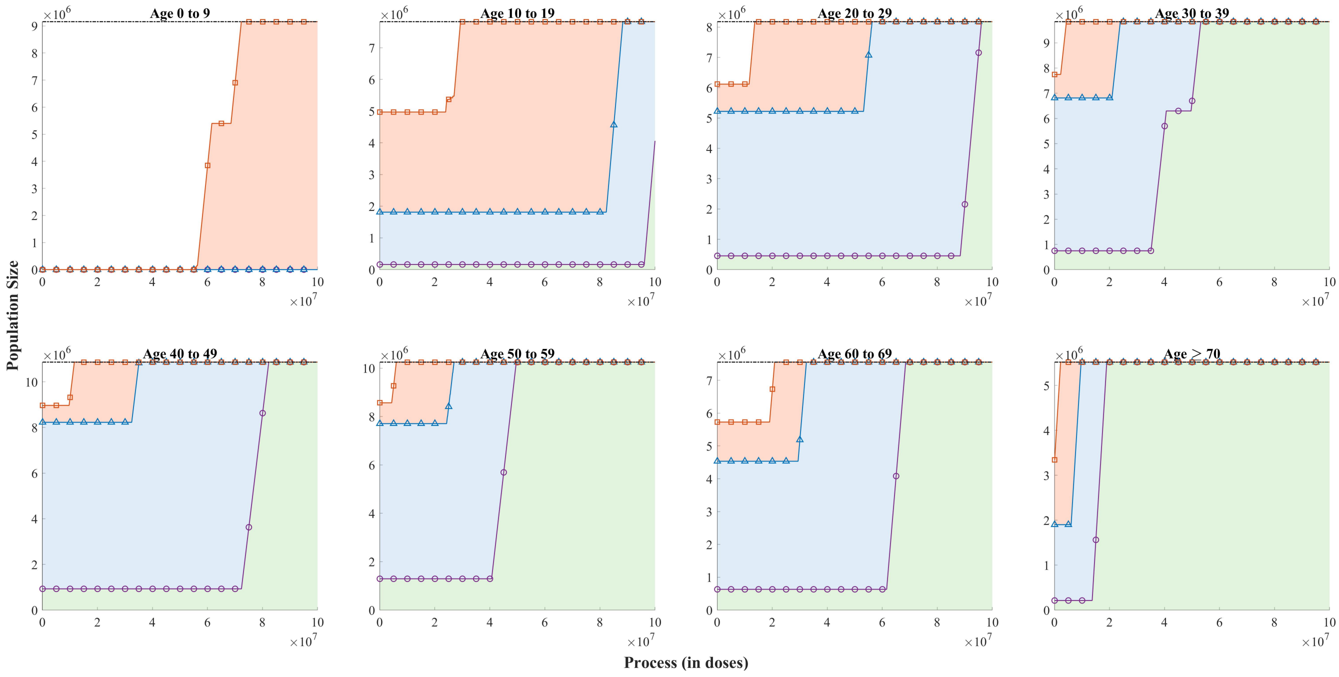

Figure. S41: Optimized Vaccinating Strategy Under Current Contact Pattern and Vaccine Coverage. For minimizing fatality of Delta variant with $R_0 = 5$. 8 subfigures represent the optimal vaccination process in 8 age groups. The x-axis represent the dose-wise vaccination process; y-axis represents vaccine coverage inside the age group (populatioon size of four vaccination status). The purple line with circles denote the population size of booster vaccinated; the blue line with triangles denote the population size of at least fully-vaccinated (including fully vaccinated and booster vaccinated); the red line with squares denote the population size of at least vaccinated (including un-fully vaccinated, fully vaccinated, and booster vaccinated). These lines depicts how coverage changes with optimized vaccination process. The line increased from the first dose gives the specific information about which should be vaccinated first.

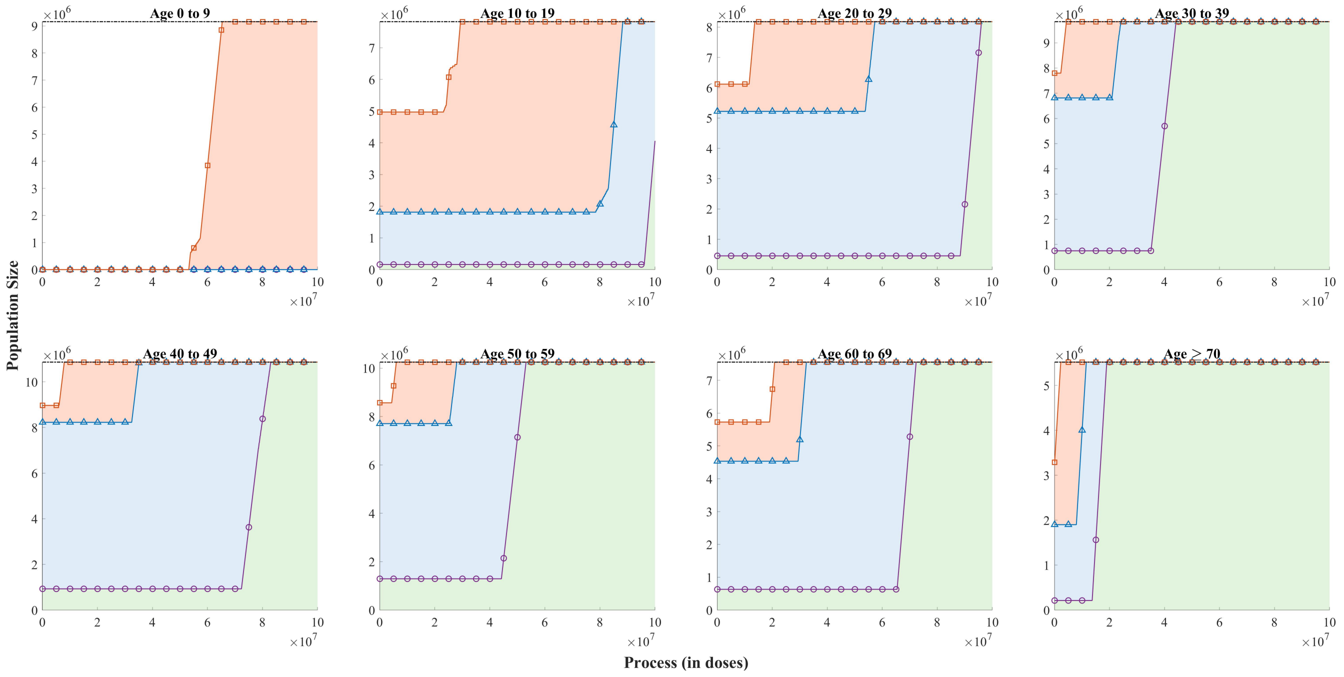

Figure. S42: Optimized Vaccinating Strategy Under Current Contact Pattern and Vaccine Coverage. For minimizing fatality of Delta variant with $R_0 = 6$. 8 subfigures represent the optimal vaccination process in 8 age groups. The x-axis represent the dose-wise vaccination process; y-axis represents vaccine coverage inside the age group (populatioon size of four vaccination status). The purple line with circles denote the population size of booster vaccinated; the blue line with triangles denote the population size of at least fully-vaccinated (including fully vaccinated and booster vaccinated); the red line with squares denote the population size of at least vaccinated (including un-fully vaccinated, fully vaccinated, and booster vaccinated). These lines depicts how coverage changes with optimized vaccination process. The line increased from the first dose gives the specific information about which should be vaccinated first.

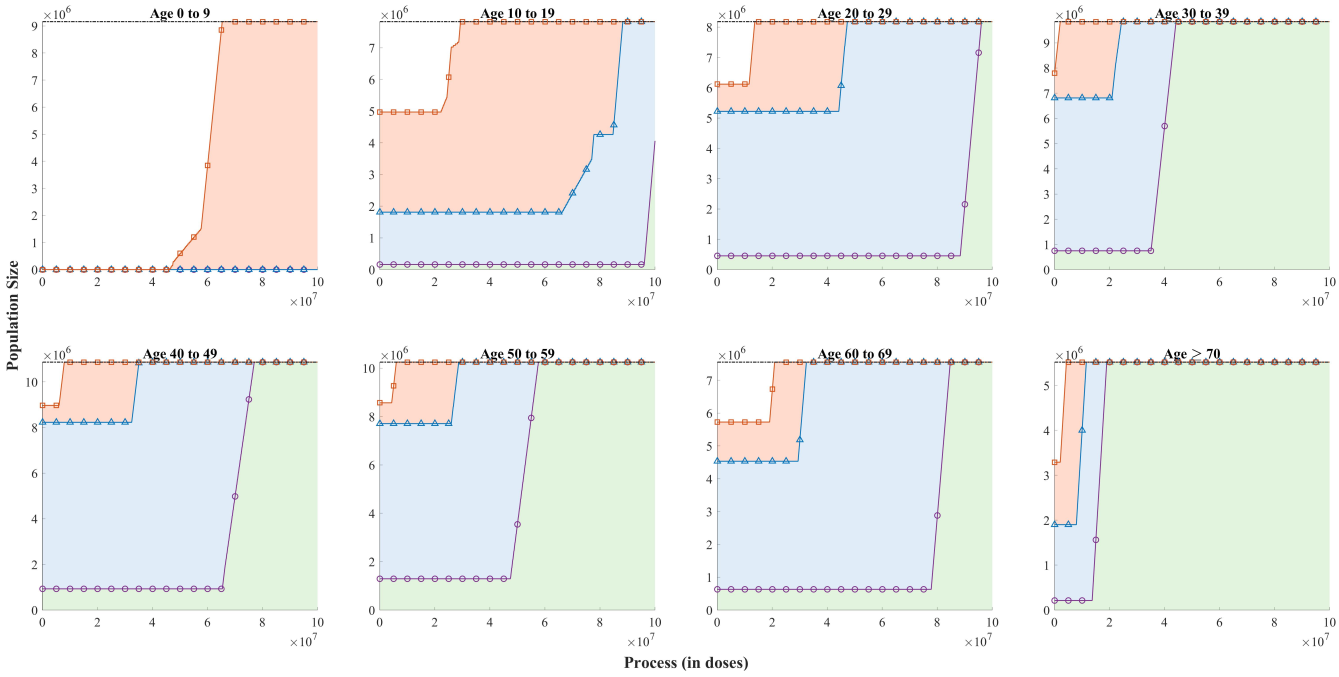

Figure. S43: Optimized Vaccinating Strategy Under Current Contact Pattern and Vaccine Coverage. For minimizing fatality of Delta variant with $R_0 = 7$. 8 subfigures represent the optimal vaccination process in 8 age groups. The x-axis represent the dose-wise vaccination process; y-axis represents vaccine coverage inside the age group (populatioon size of four vaccination status). The purple line with circles denote the population size of booster vaccinated; the blue line with triangles denote the population size of at least fully-vaccinated (including fully vaccinated and booster vaccinated); the red line with squares denote the population size of at least vaccinated (including un-fully vaccinated, fully vaccinated, and booster vaccinated). These lines depicts how coverage changes with optimized vaccination process. The line increased from the first dose gives the specific information about which should be vaccinated first.

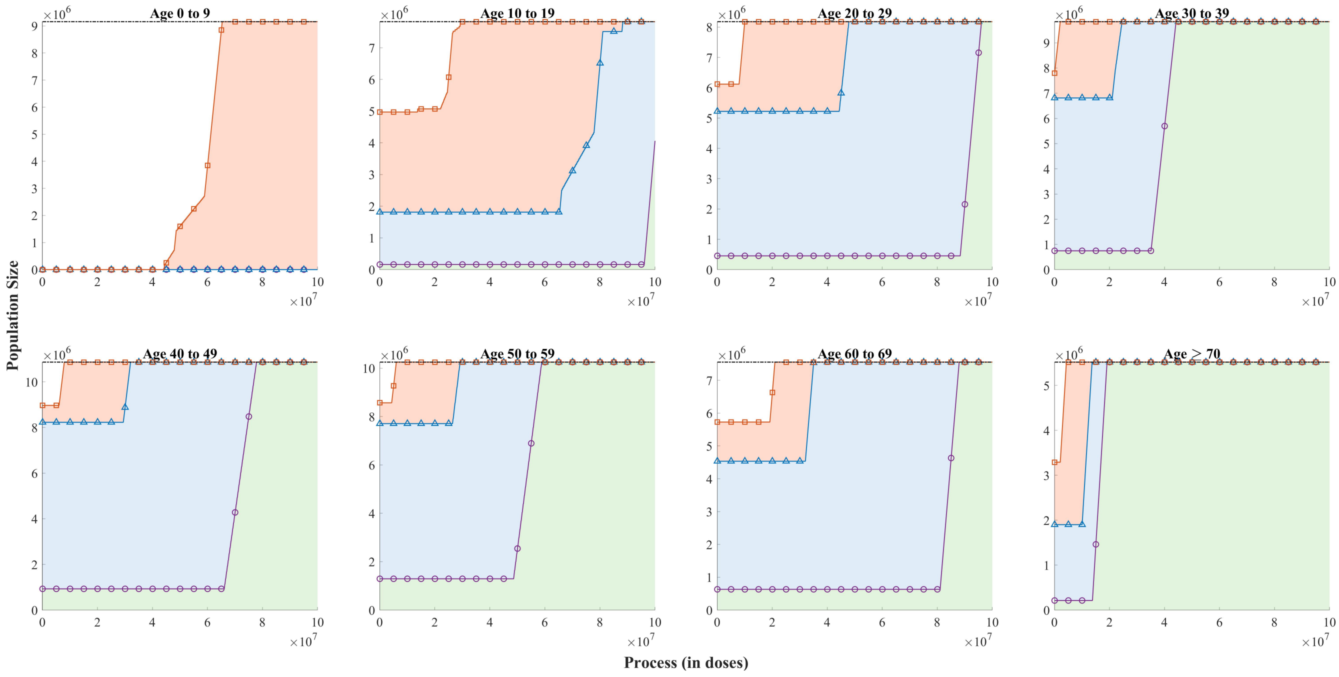

Figure. S44: Optimized Vaccinating Strategy Under Current Contact Pattern and Vaccine Coverage. For minimizing fatality of Delta variant with $R_0 = 8$. 8 subfigures represent the optimal vaccination process in 8 age groups. The x-axis represent the dose-wise vaccination process; y-axis represents vaccine coverage inside the age group (populatioon size of four vaccination status). The purple line with circles denote the population size of booster vaccinated; the blue line with triangles denote the population size of at least fully-vaccinated (including fully vaccinated and booster vaccinated); the red line with squares denote the population size of at least vaccinated (including un-fully vaccinated, fully vaccinated, and booster vaccinated). These lines depicts how coverage changes with optimized vaccination process. The line increased from the first dose gives the specific information about which should be vaccinated first.

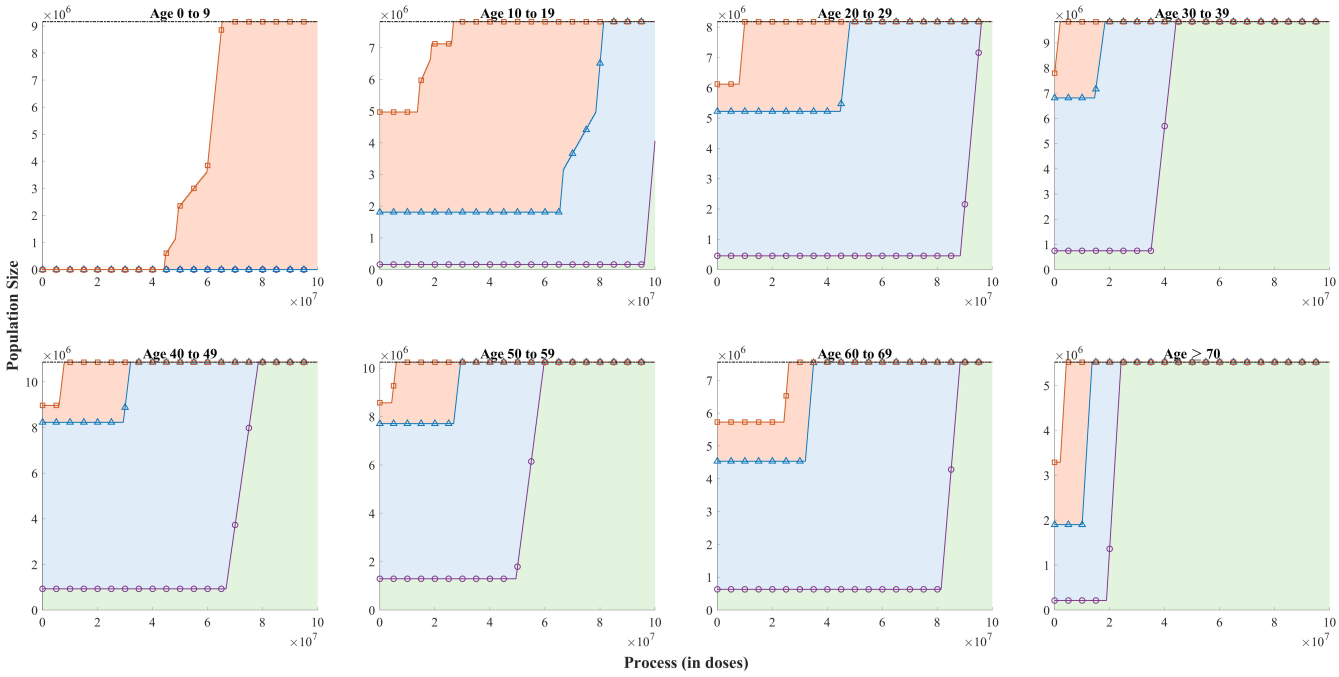

Figure. S45: Optimized Vaccinating Strategy Under Current Contact Pattern and Vaccine Coverage. For minimizing fatality of Delta variant with $R_0 = 9$. 8 subfigures represent the optimal vaccination process in 8 age groups. The x-axis represent the dose-wise vaccination process; y-axis represents vaccine coverage inside the age group (populatioon size of four vaccination status). The purple line with circles denote the population size of booster vaccinated; the blue line with triangles denote the population size of at least fully-vaccinated (including fully vaccinated and booster vaccinated); the red line with squares denote the population size of at least vaccinated (including un-fully vaccinated, fully vaccinated, and booster vaccinated). These lines depicts how coverage changes with optimized vaccination process. The line increased from the first dose gives the specific information about which should be vaccinated first.

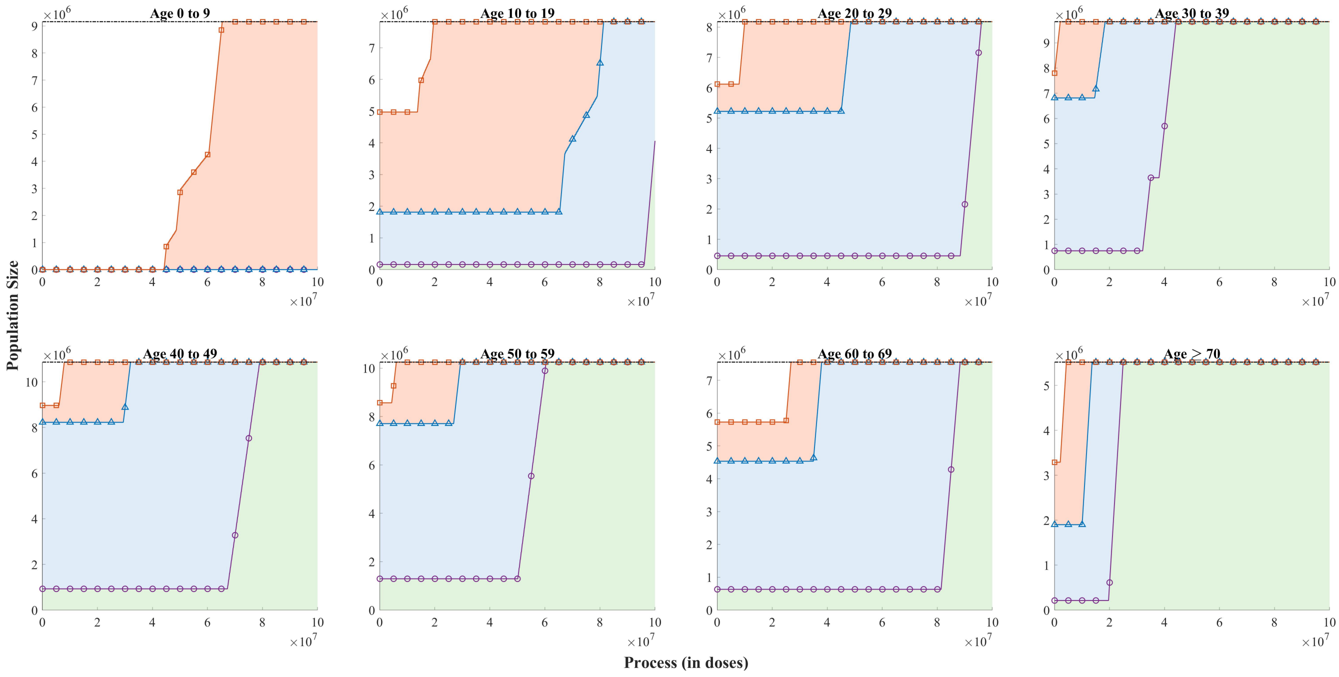

Figure. S46: Optimized Vaccinating Strategy Under Current Contact Pattern and Vaccine Coverage. For minimizing fatality of Delta variant with $R_0 = 10$. 8 subfigures represent the optimal vaccination process in 8 age groups. The x-axis represent the dose-wise vaccination process; y-axis represents vaccine coverage inside the age group (populatioon size of four vaccination status). The purple line with circles denote the population size of booster vaccinated; the blue line with triangles denote the population size of at least fully-vaccinated (including fully vaccinated and booster vaccinated); the red line with squares denote the population size of at least vaccinated (including un-fully vaccinated, fully vaccinated, and booster vaccinated). These lines depicts how coverage changes with optimized vaccination process. The line increased from the first dose gives the specific information about which should be vaccinated first.

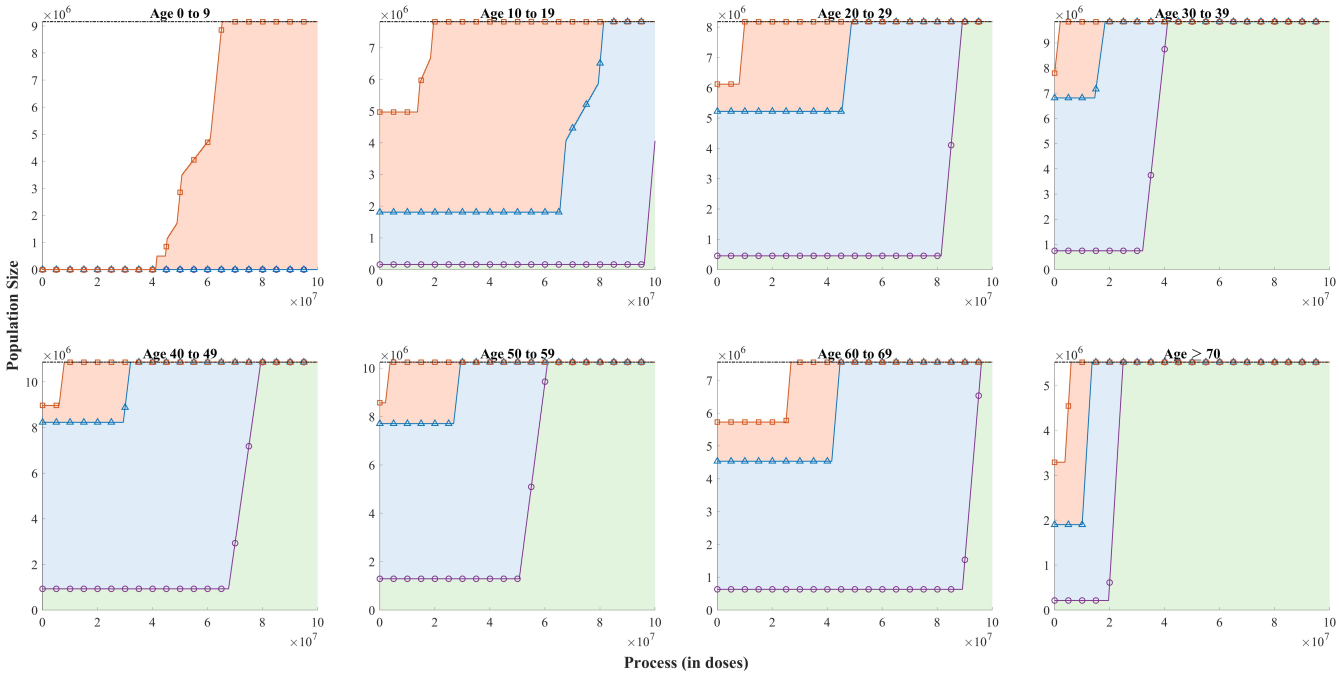

Figure. S47: Optimized Vaccinating Strategy Under Current Contact Pattern and Vaccine Coverage. For minimizing fatality of Delta variant with $R_0 = 11$. 8 subfigures represent the optimal vaccination process in 8 age groups. The x-axis represent the dose-wise vaccination process; y-axis represents vaccine coverage inside the age group (populatioon size of four vaccination status). The purple line with circles denote the population size of booster vaccinated; the blue line with triangles denote the population size of at least fully-vaccinated (including fully vaccinated and booster vaccinated); the red line with squares denote the population size of at least vaccinated (including un-fully vaccinated, fully vaccinated, and booster vaccinated). These lines depicts how coverage changes with optimized vaccination process. The line increased from the first dose gives the specific information about which should be vaccinated first.

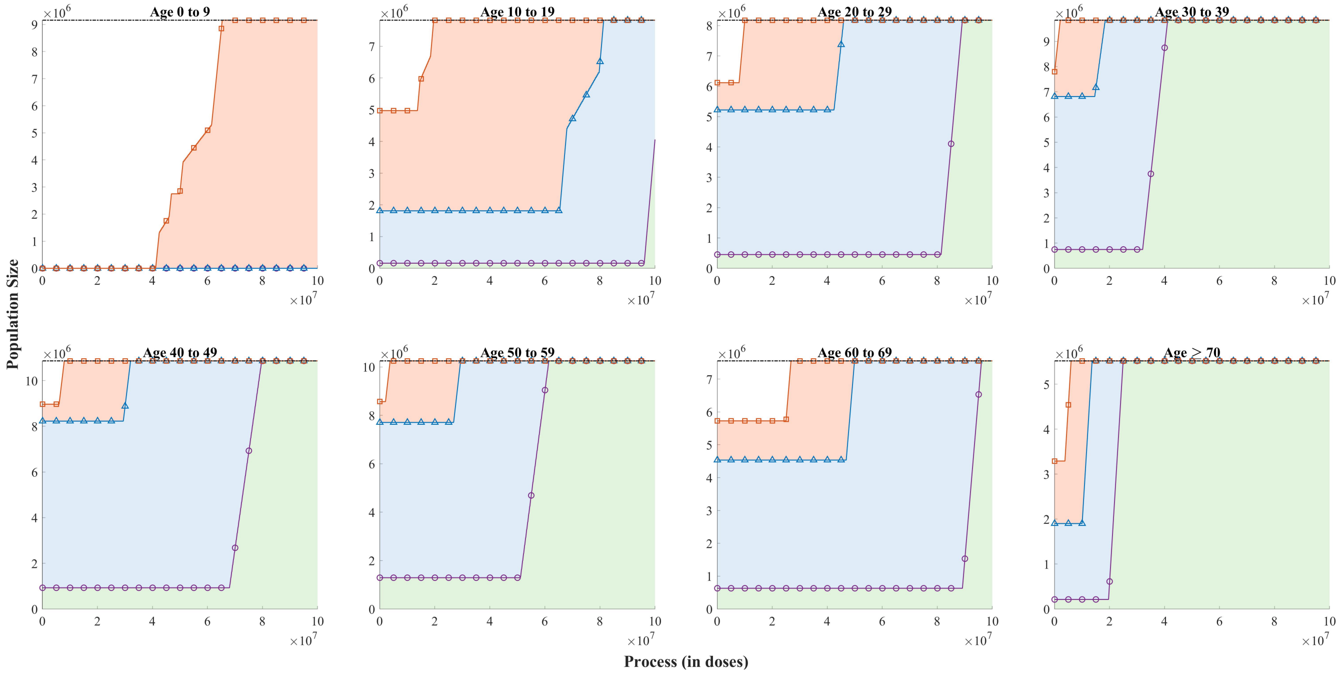

Figure. S48: Optimized Vaccinating Strategy Under Current Contact Pattern and Vaccine Coverage. For minimizing fatality of Delta variant with $R_0 = 12$. 8 subfigures represent the optimal vaccination process in 8 age groups. The x-axis represent the dose-wise vaccination process; y-axis represents vaccine coverage inside the age group (populatioon size of four vaccination status). The purple line with circles denote the population size of booster vaccinated; the blue line with triangles denote the population size of at least fully-vaccinated (including fully vaccinated and booster vaccinated); the red line with squares denote the population size of at least vaccinated (including un-fully vaccinated, fully vaccinated, and booster vaccinated). These lines depicts how coverage changes with optimized vaccination process. The line increased from the first dose gives the specific information about which should be vaccinated first.

With the optimal vaccination process, the fatality in whole population within 14 days from the first illness onset is shown in figure 4.5.1.

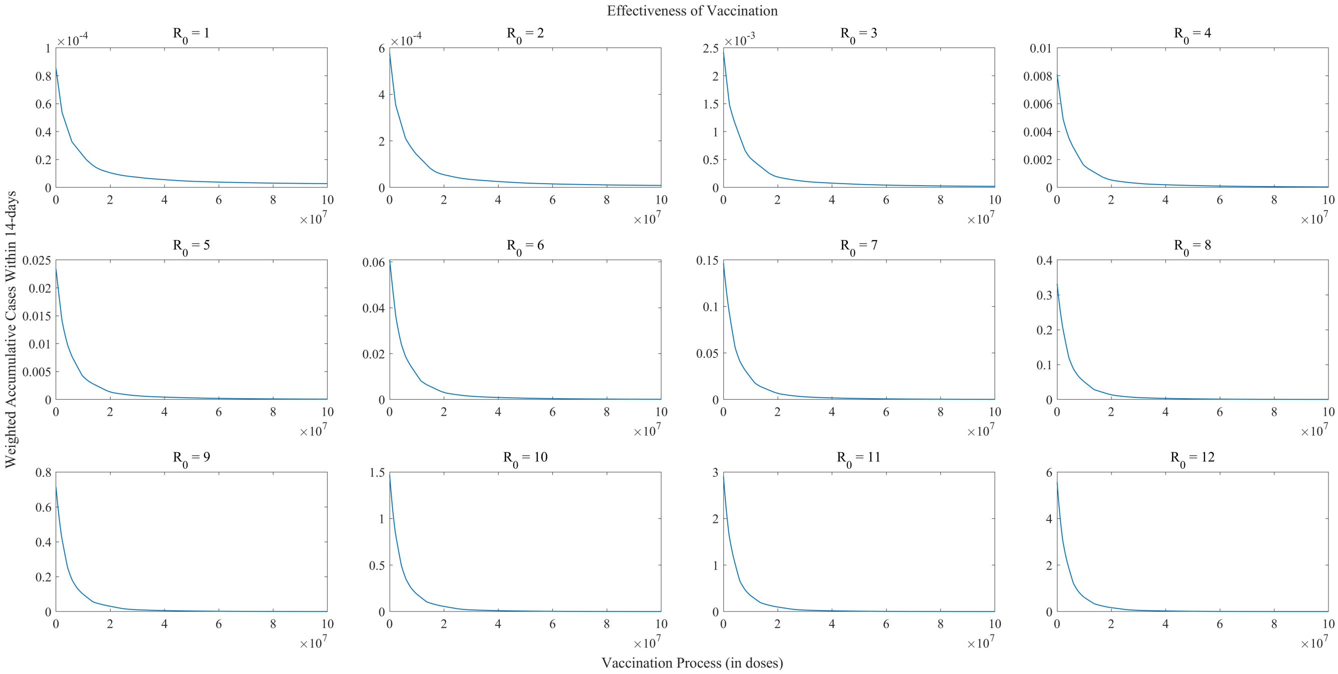

Figure. S49: Effectiveness of Optimized Vaccination. Under Current Contact Pattern and Vaccine Coverage. With parameters of Delta variant.

4.5.4 Cumulative Cases for Omicron Variant

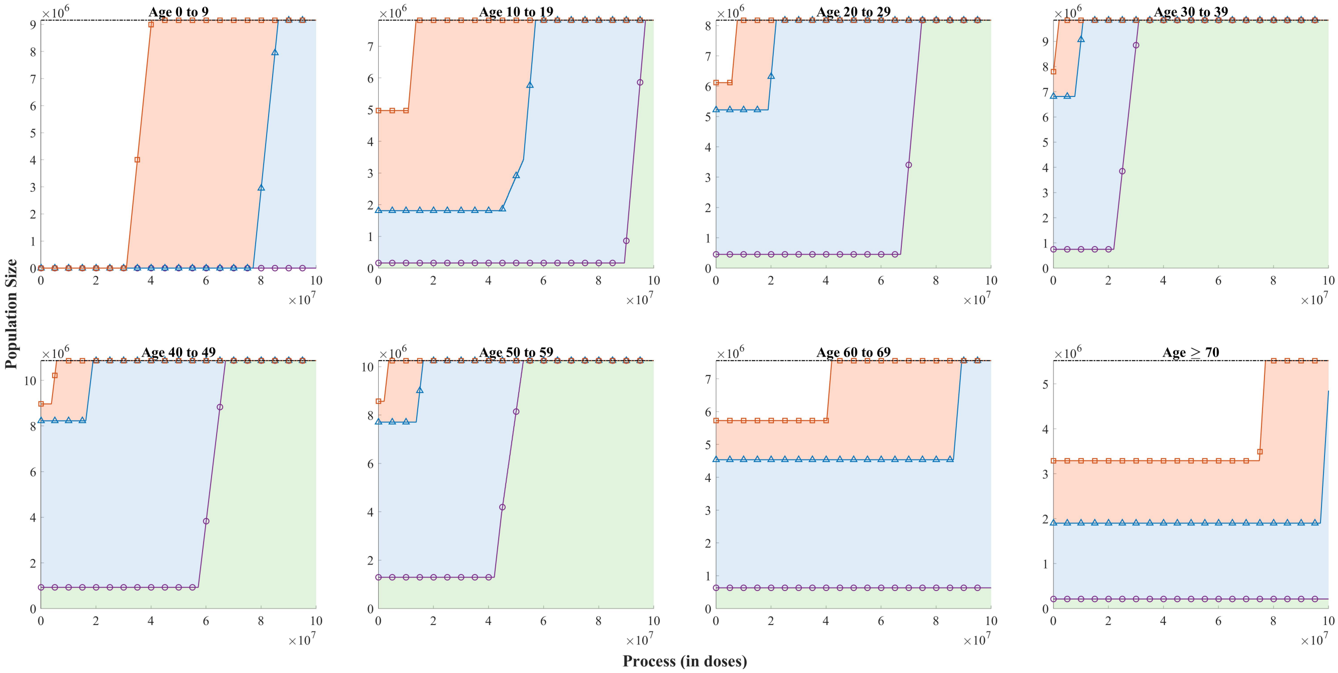

Figure. S50: Optimized Vaccinating Strategy Under Current Contact Pattern and Vaccine Coverage. For minimizing cumulative cases of Omicron variant with $R_0 = 1$. 8 subfigures represent the optimal vaccination process in 8 age groups. The x-axis represent the dose-wise vaccination process; y-axis represents vaccine coverage inside the age group (populatioon size of four vaccination status). The purple line with circles denote the population size of booster vaccinated; the blue line with triangles denote the population size of at least fully-vaccinated (including fully vaccinated and booster vaccinated); the red line with squares denote the population size of at least vaccinated (including un-fully vaccinated, fully vaccinated, and booster vaccinated). These lines depicts how coverage changes with optimized vaccination process. The line increased from the first dose gives the specific information about which should be vaccinated first.

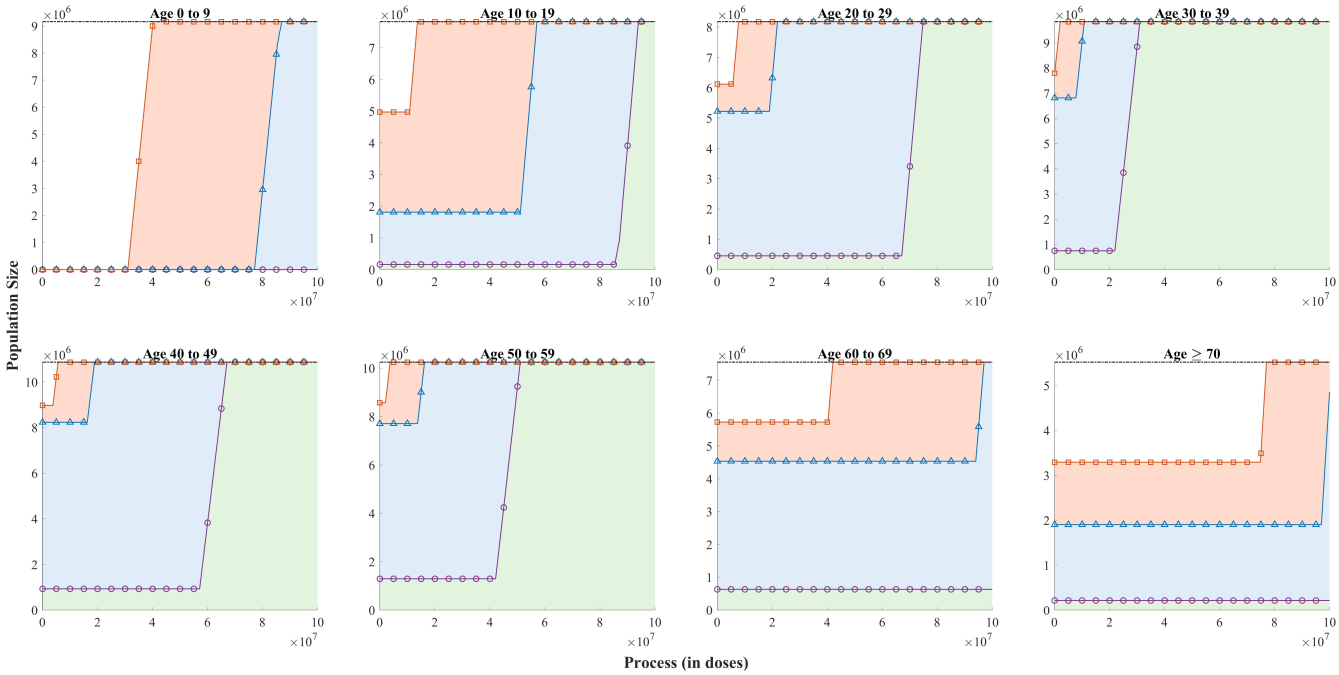

Figure. S51: Optimized Vaccinating Strategy Under Current Contact Pattern and Vaccine Coverage. For minimizing cumulative cases of Omicron variant with $R_0 = 2$. 8 subfigures represent the optimal vaccination process in 8 age groups. The x-axis represent the dose-wise vaccination process; y-axis represents vaccine coverage inside the age group (populatioon size of four vaccination status). The purple line with circles denote the population size of booster vaccinated; the blue line with triangles denote the population size of at least fully-vaccinated (including fully vaccinated and booster vaccinated); the red line with squares denote the population size of at least vaccinated (including un-fully vaccinated, fully vaccinated, and booster vaccinated). These lines depicts how coverage changes with optimized vaccination process. The line increased from the first dose gives the specific information about which should be vaccinated first.

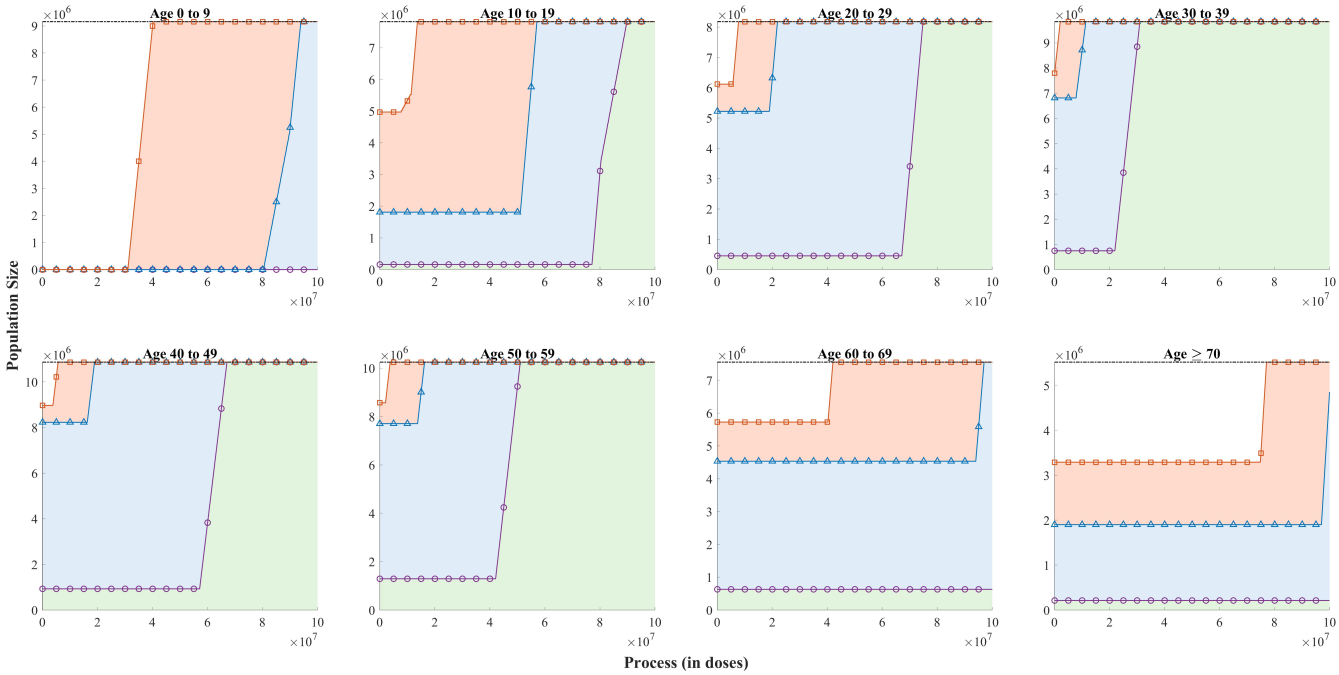

Figure. S52: Optimized Vaccinating Strategy Under Current Contact Pattern and Vaccine Coverage. For minimizing cumulative cases of Omicron variant with $R_0 = 3$. 8 subfigures represent the optimal vaccination process in 8 age groups. The x-axis represent the dose-wise vaccination process; y-axis represents vaccine coverage inside the age group (populatioon size of four vaccination status). The purple line with circles denote the population size of booster vaccinated; the blue line with triangles denote the population size of at least fully-vaccinated (including fully vaccinated and booster vaccinated); the red line with squares denote the population size of at least vaccinated (including un-fully vaccinated, fully vaccinated, and booster vaccinated). These lines depicts how coverage changes with optimized vaccination process. The line increased from the first dose gives the specific information about which should be vaccinated first.

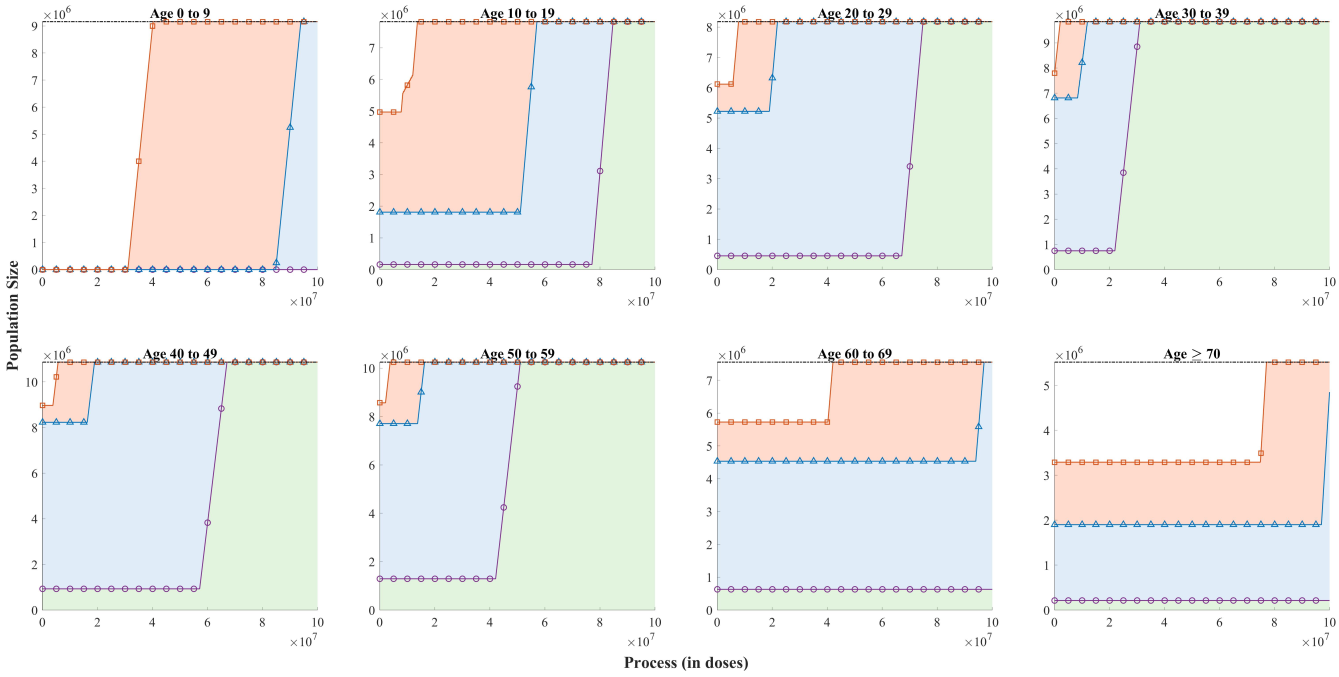

Figure. S53: Optimized Vaccinating Strategy Under Current Contact Pattern and Vaccine Coverage. For minimizing cumulative cases of Omicron variant with $R_0 = 4$. 8 subfigures represent the optimal vaccination process in 8 age groups. The x-axis represent the dose-wise vaccination process; y-axis represents vaccine coverage inside the age group (populatioon size of four vaccination status). The purple line with circles denote the population size of booster vaccinated; the blue line with triangles denote the population size of at least fully-vaccinated (including fully vaccinated and booster vaccinated); the red line with squares denote the population size of at least vaccinated (including un-fully vaccinated, fully vaccinated, and booster vaccinated). These lines depicts how coverage changes with optimized vaccination process. The line increased from the first dose gives the specific information about which should be vaccinated first.

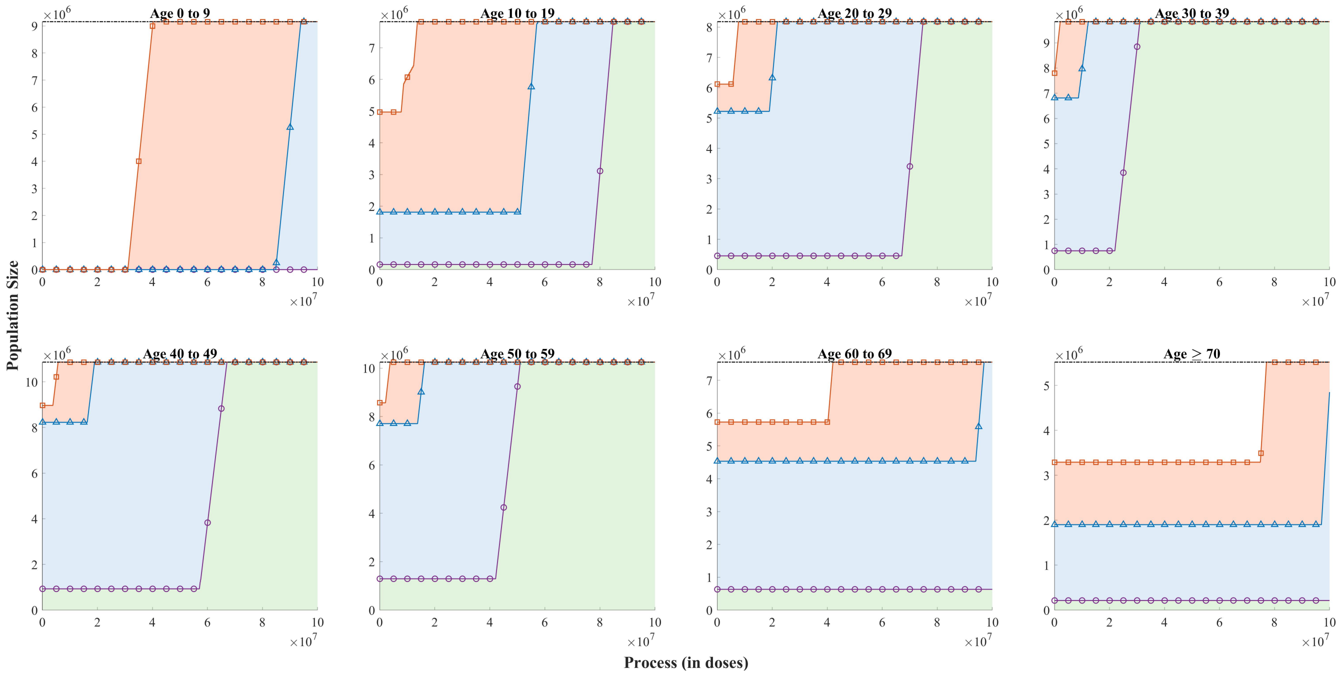

Figure. S54: Optimized Vaccinating Strategy Under Current Contact Pattern and Vaccine Coverage. For minimizing cumulative cases of Omicron variant with $R_0 = 5$. 8 subfigures represent the optimal vaccination process in 8 age groups. The x-axis represent the dose-wise vaccination process; y-axis represents vaccine coverage inside the age group (populatioon size of four vaccination status). The purple line with circles denote the population size of booster vaccinated; the blue line with triangles denote the population size of at least fully-vaccinated (including fully vaccinated and booster vaccinated); the red line with squares denote the population size of at least vaccinated (including un-fully vaccinated, fully vaccinated, and booster vaccinated). These lines depicts how coverage changes with optimized vaccination process. The line increased from the first dose gives the specific information about which should be vaccinated first.

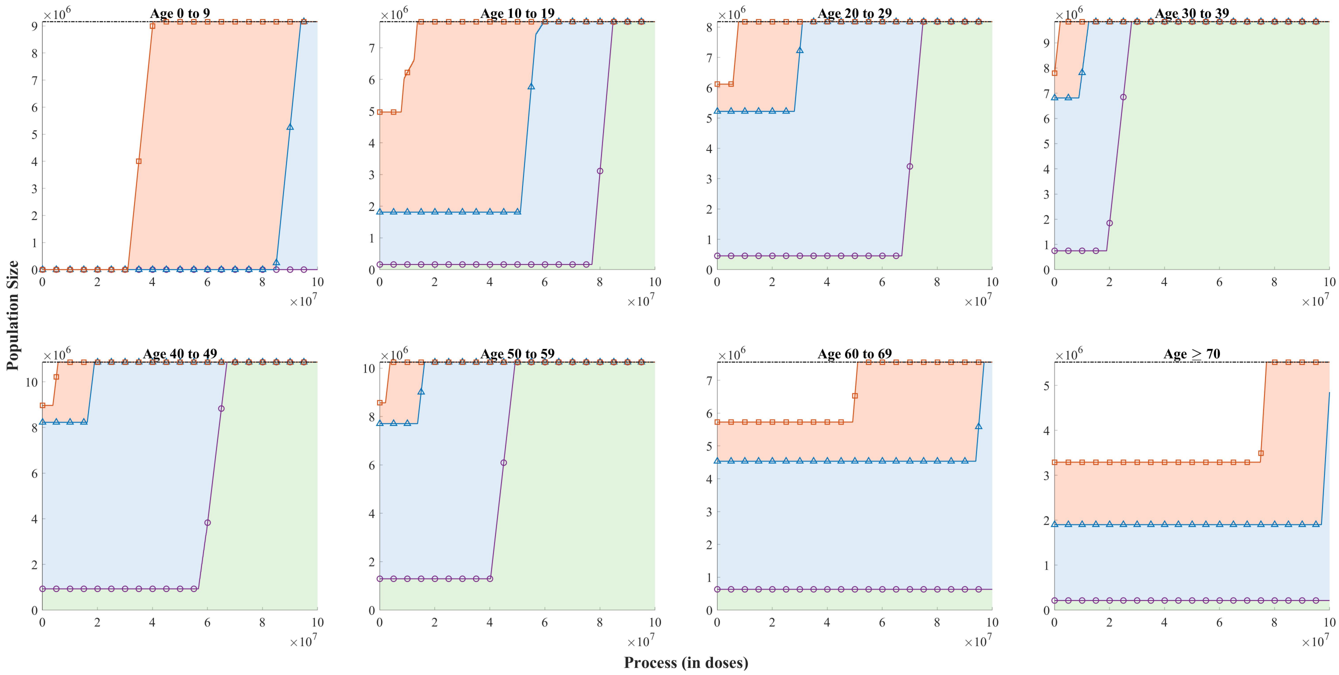

Figure. S55: Optimized Vaccinating Strategy Under Current Contact Pattern and Vaccine Coverage. For minimizing cumulative cases of Omicron variant with $R_0 = 6$. 8 subfigures represent the optimal vaccination process in 8 age groups. The x-axis represent the dose-wise vaccination process; y-axis represents vaccine coverage inside the age group (populatioon size of four vaccination status). The purple line with circles denote the population size of booster vaccinated; the blue line with triangles denote the population size of at least fully-vaccinated (including fully vaccinated and booster vaccinated); the red line with squares denote the population size of at least vaccinated (including un-fully vaccinated, fully vaccinated, and booster vaccinated). These lines depicts how coverage changes with optimized vaccination process. The line increased from the first dose gives the specific information about which should be vaccinated first.

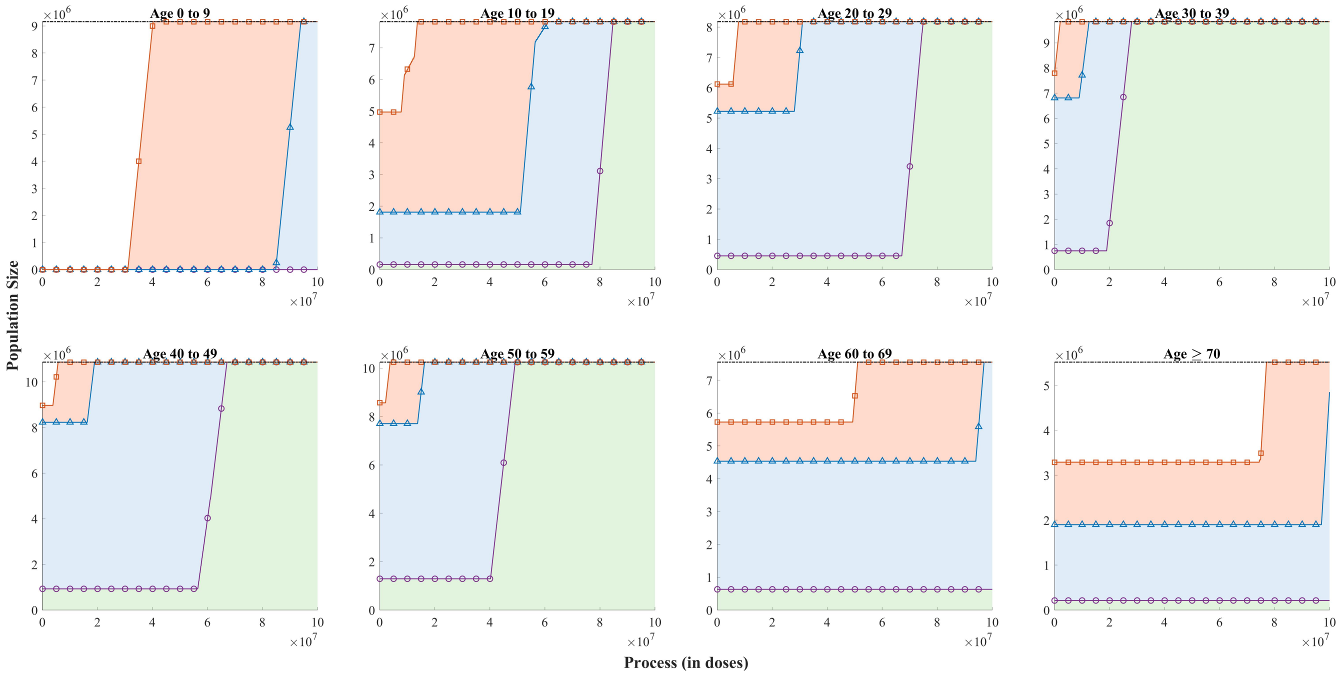

Figure. S56: Optimized Vaccinating Strategy Under Current Contact Pattern and Vaccine Coverage. For minimizing cumulative cases of Omicron variant with $R_0 = 7$. 8 subfigures represent the optimal vaccination process in 8 age groups. The x-axis represent the dose-wise vaccination process; y-axis represents vaccine coverage inside the age group (populatioon size of four vaccination status). The purple line with circles denote the population size of booster vaccinated; the blue line with triangles denote the population size of at least fully-vaccinated (including fully vaccinated and booster vaccinated); the red line with squares denote the population size of at least vaccinated (including un-fully vaccinated, fully vaccinated, and booster vaccinated). These lines depicts how coverage changes with optimized vaccination process. The line increased from the first dose gives the specific information about which should be vaccinated first.

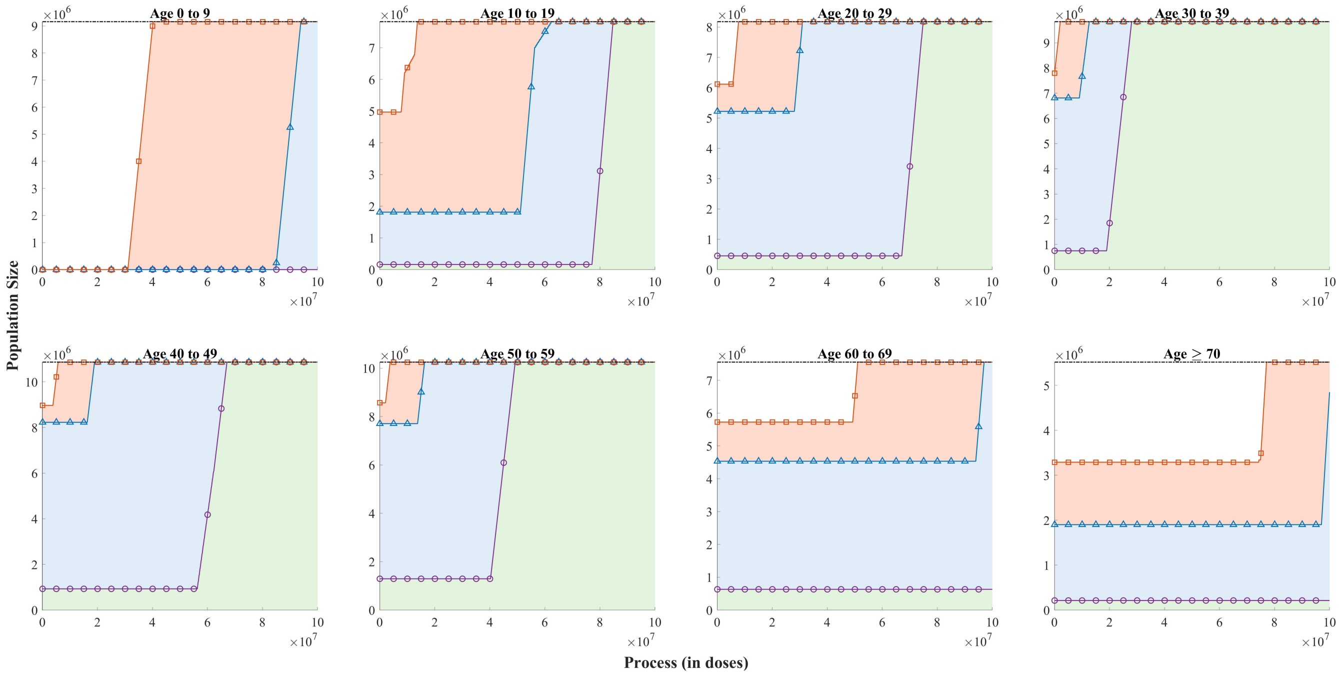

Figure. S57: Optimized Vaccinating Strategy Under Current Contact Pattern and Vaccine Coverage. For minimizing cumulative cases of Omicron variant with $R_0 = 8$. 8 subfigures represent the optimal vaccination process in 8 age groups. The x-axis represent the dose-wise vaccination process; y-axis represents vaccine coverage inside the age group (populatioon size of four vaccination status). The purple line with circles denote the population size of booster vaccinated; the blue line with triangles denote the population size of at least fully-vaccinated (including fully vaccinated and booster vaccinated); the red line with squares denote the population size of at least vaccinated (including un-fully vaccinated, fully vaccinated, and booster vaccinated). These lines depicts how coverage changes with optimized vaccination process. The line increased from the first dose gives the specific information about which should be vaccinated first.

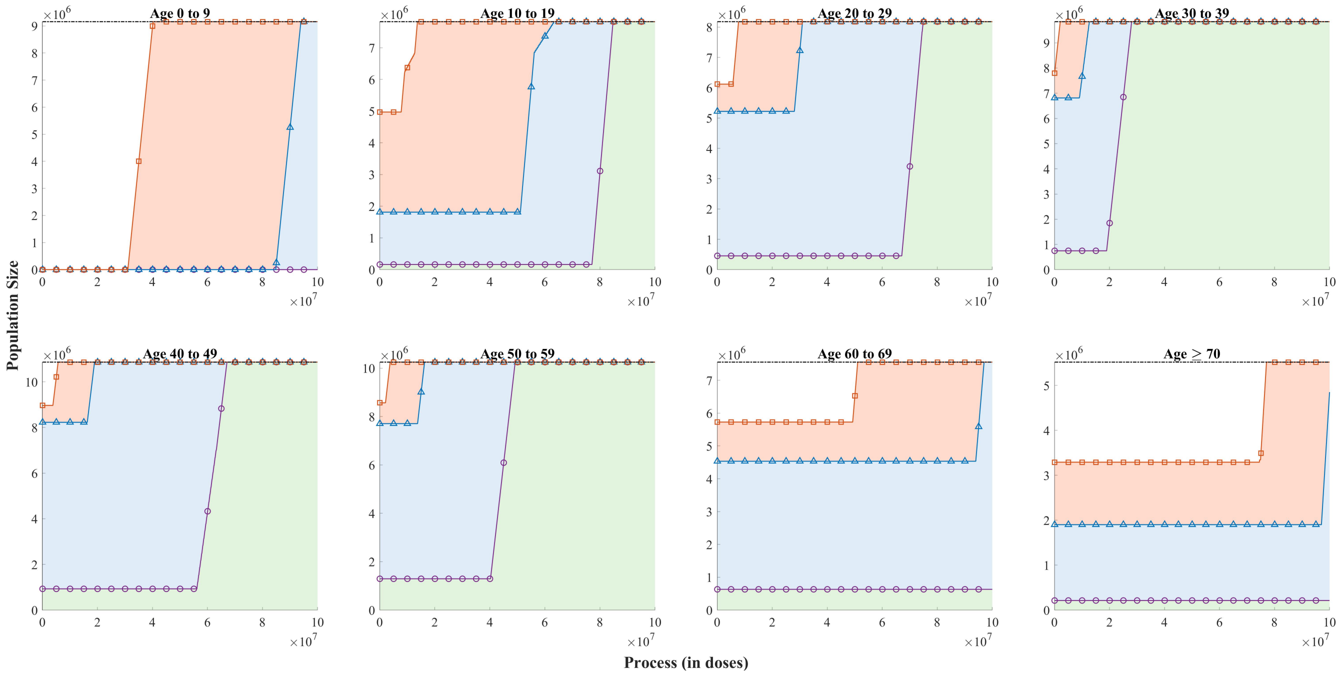

Figure. S58: Optimized Vaccinating Strategy Under Current Contact Pattern and Vaccine Coverage. For minimizing cumulative cases of Omicron variant with $R_0 = 9$. 8 subfigures represent the optimal vaccination process in 8 age groups. The x-axis represent the dose-wise vaccination process; y-axis represents vaccine coverage inside the age group (populatioon size of four vaccination status). The purple line with circles denote the population size of booster vaccinated; the blue line with triangles denote the population size of at least fully-vaccinated (including fully vaccinated and booster vaccinated); the red line with squares denote the population size of at least vaccinated (including un-fully vaccinated, fully vaccinated, and booster vaccinated). These lines depicts how coverage changes with optimized vaccination process. The line increased from the first dose gives the specific information about which should be vaccinated first.

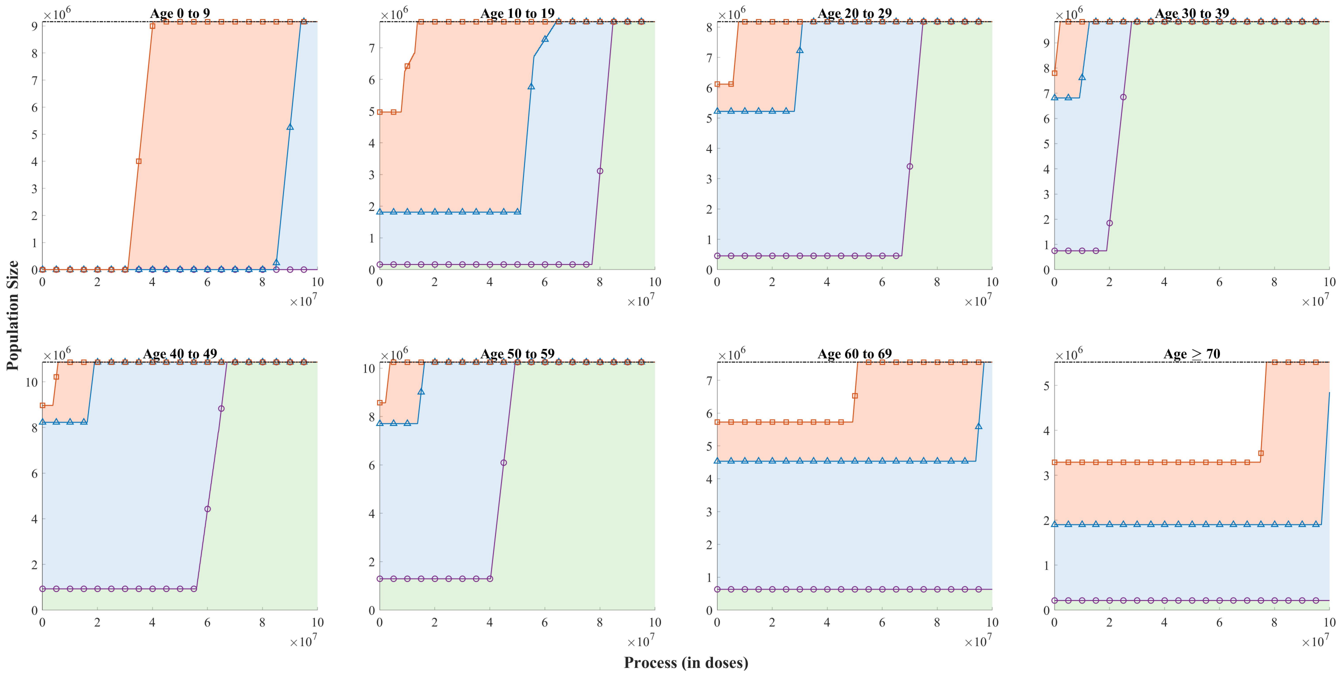

Figure. S59: Optimized Vaccinating Strategy Under Current Contact Pattern and Vaccine Coverage. For minimizing cumulative cases of Omicron variant with $R_0 = 10$. 8 subfigures represent the optimal vaccination process in 8 age groups. The x-axis represent the dose-wise vaccination process; y-axis represents vaccine coverage inside the age group (populatioon size of four vaccination status). The purple line with circles denote the population size of booster vaccinated; the blue line with triangles denote the population size of at least fully-vaccinated (including fully vaccinated and booster vaccinated); the red line with squares denote the population size of at least vaccinated (including un-fully vaccinated, fully vaccinated, and booster vaccinated). These lines depicts how coverage changes with optimized vaccination process. The line increased from the first dose gives the specific information about which should be vaccinated first.

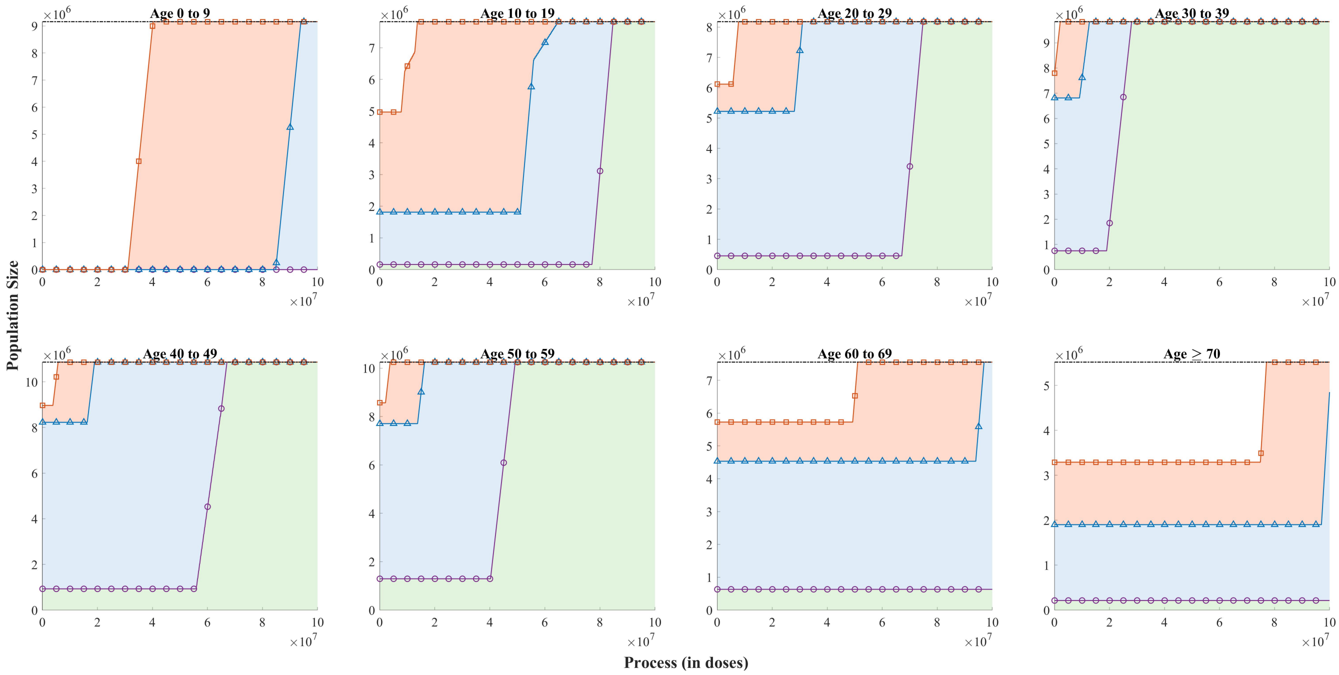

Figure. S60: Optimized Vaccinating Strategy Under Current Contact Pattern and Vaccine Coverage. For minimizing cumulative cases of Omicron variant with $R_0 = 11$. 8 subfigures represent the optimal vaccination process in 8 age groups. The x-axis represent the dose-wise vaccination process; y-axis represents vaccine coverage inside the age group (populatioon size of four vaccination status). The purple line with circles denote the population size of booster vaccinated; the blue line with triangles denote the population size of at least fully-vaccinated (including fully vaccinated and booster vaccinated); the red line with squares denote the population size of at least vaccinated (including un-fully vaccinated, fully vaccinated, and booster vaccinated). These lines depicts how coverage changes with optimized vaccination process. The line increased from the first dose gives the specific information about which should be vaccinated first.

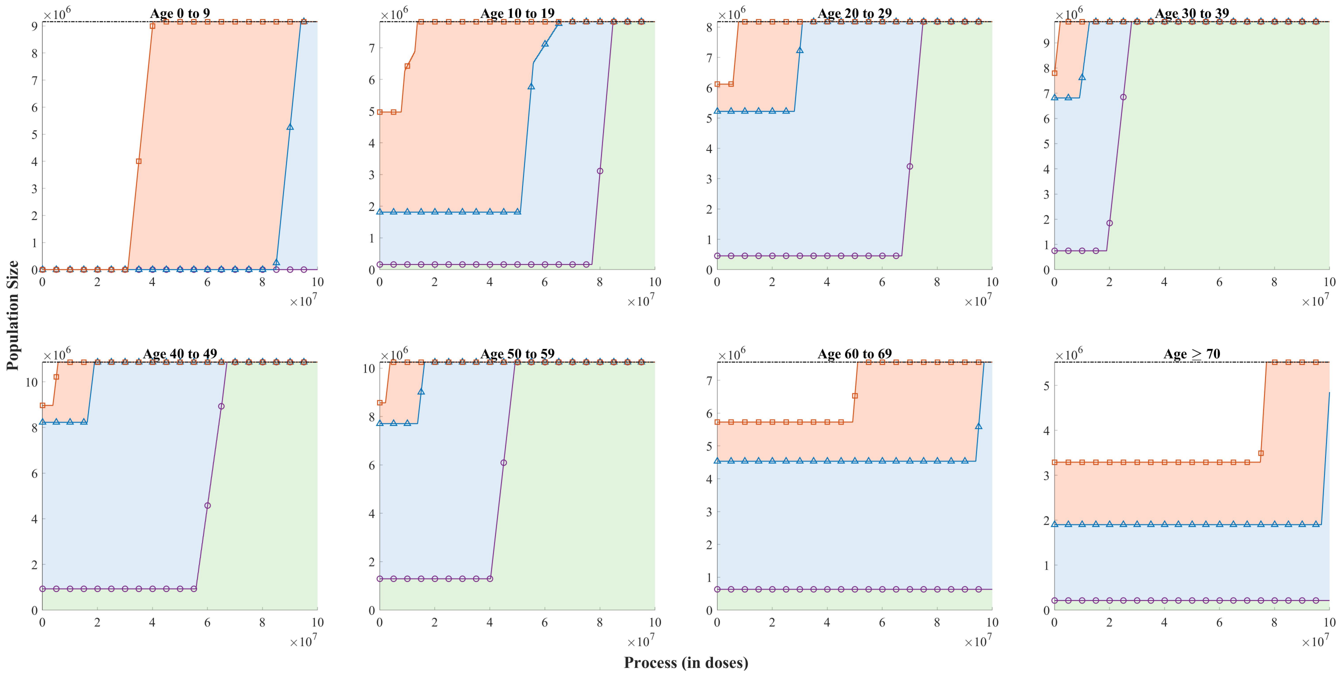

Figure. S61: Optimized Vaccinating Strategy Under Current Contact Pattern and Vaccine Coverage. For minimizing cumulative cases of Omicron variant with $R_0 = 12$. 8 subfigures represent the optimal vaccination process in 8 age groups. The x-axis represent the dose-wise vaccination process; y-axis represents vaccine coverage inside the age group (populatioon size of four vaccination status). The purple line with circles denote the population size of booster vaccinated; the blue line with triangles denote the population size of at least fully-vaccinated (including fully vaccinated and booster vaccinated); the red line with squares denote the population size of at least vaccinated (including un-fully vaccinated, fully vaccinated, and booster vaccinated). These lines depicts how coverage changes with optimized vaccination process. The line increased from the first dose gives the specific information about which should be vaccinated first.

With the optimal vaccination process, the fatality in whole population within 14 days from the first illness onset is shown in figure ??.

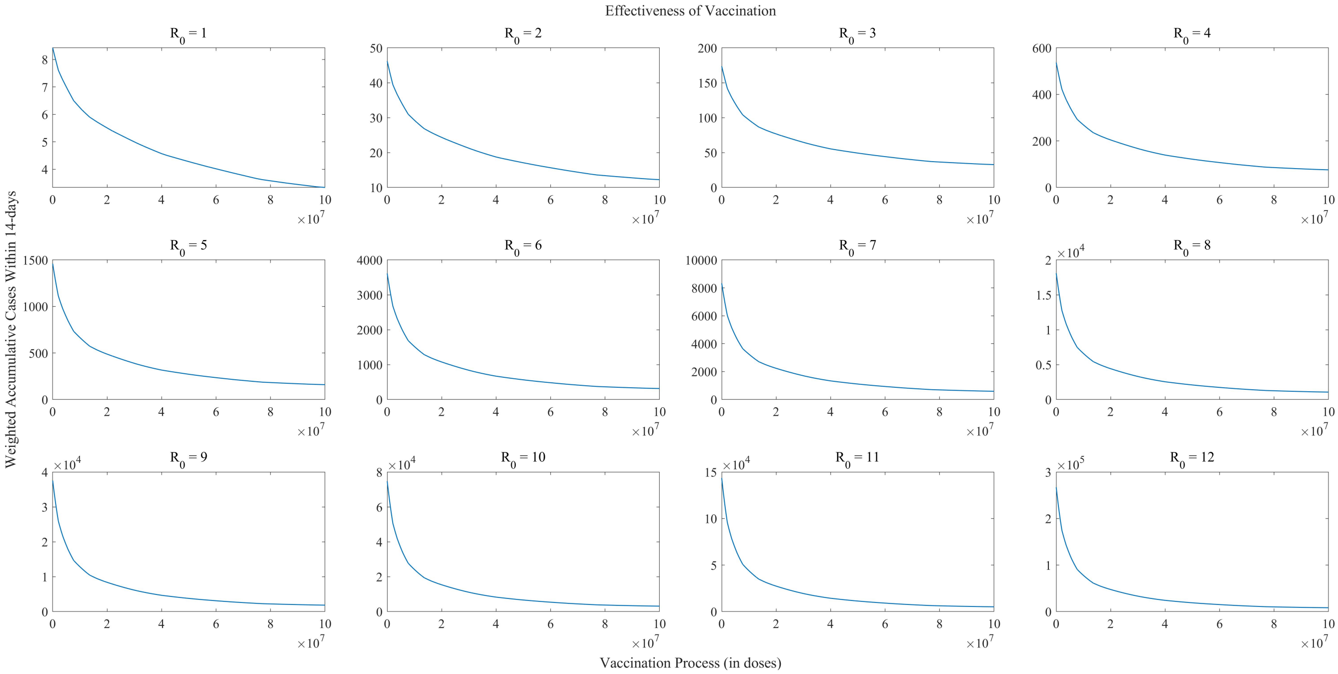

Figure. S62: Effectiveness of Optimized Vaccination. Under Current Contact Pattern and Vaccine Coverage. With parameters of Delta variant.

4.5.5 Hospitalization for Omicron Variant

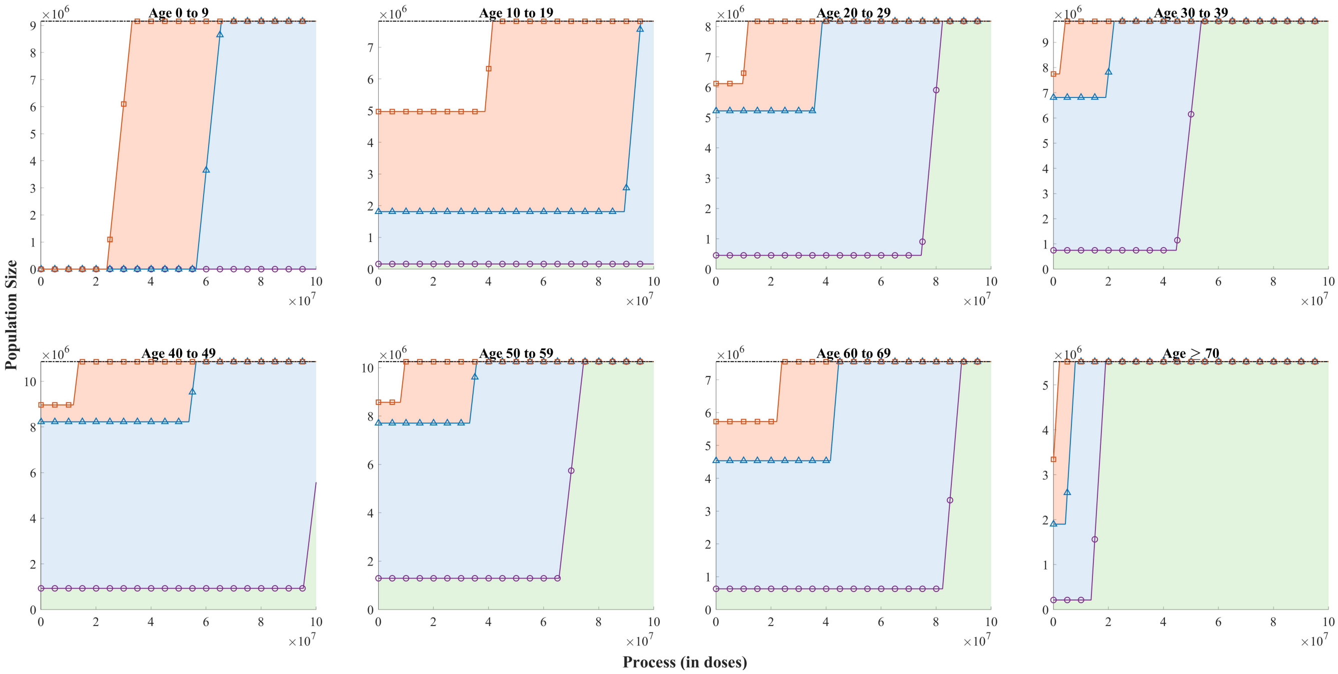

Figure. S63: Optimized Vaccinating Strategy Under Current Contact Pattern and Vaccine Coverage. For minimizing hospitalization of Omicron variant with $R_0 = 1$. 8 subfigures represent the optimal vaccination process in 8 age groups. The x-axis represent the dose-wise vaccination process; y-axis represents vaccine coverage inside the age group (populatioon size of four vaccination status). The purple line with circles denote the population size of booster vaccinated; the blue line with triangles denote the population size of at least fully-vaccinated (including fully vaccinated and booster vaccinated); the red line with squares denote the population size of at least vaccinated (including un-fully vaccinated, fully vaccinated, and booster vaccinated). These lines depicts how coverage changes with optimized vaccination process. The line increased from the first dose gives the specific information about which should be vaccinated first.

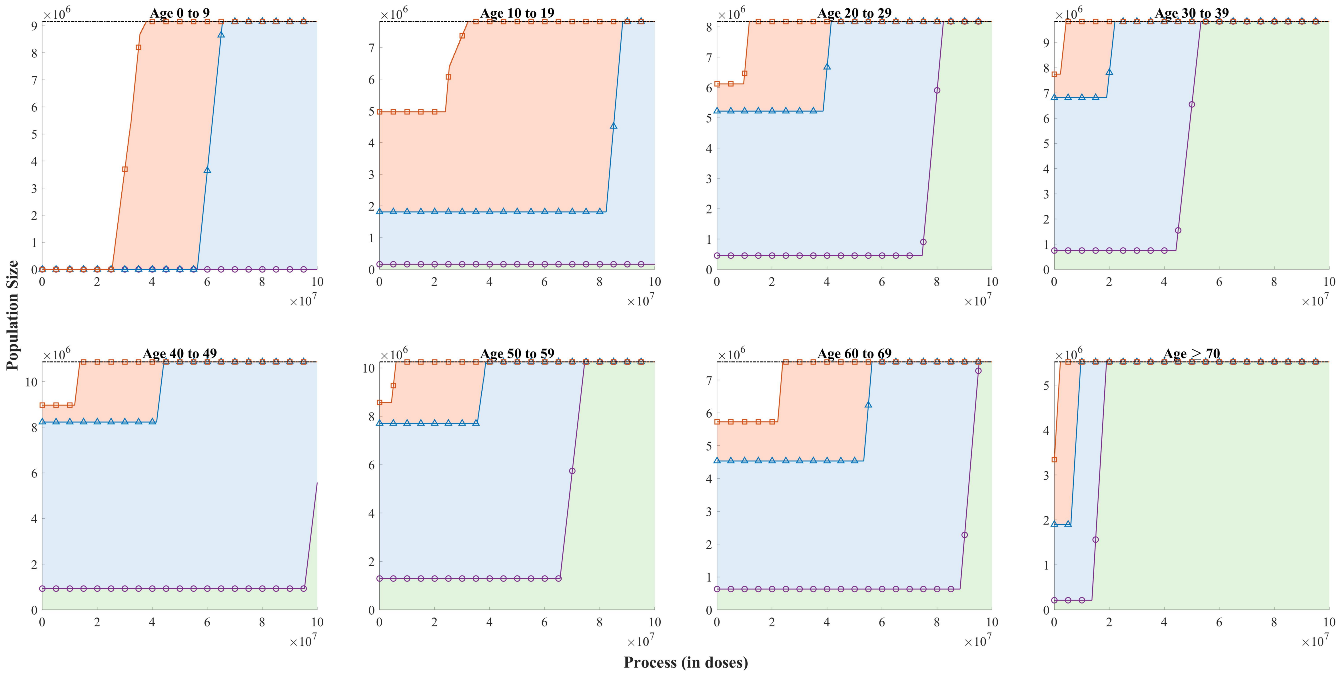

Figure. S64: Optimized Vaccinating Strategy Under Current Contact Pattern and Vaccine Coverage. For minimizing hospitalization of Omicron variant with $R_0 = 2$. 8 subfigures represent the optimal vaccination process in 8 age groups. The x-axis represent the dose-wise vaccination process; y-axis represents vaccine coverage inside the age group (populatioon size of four vaccination status). The purple line with circles denote the population size of booster vaccinated; the blue line with triangles denote the population size of at least fully-vaccinated (including fully vaccinated and booster vaccinated); the red line with squares denote the population size of at least vaccinated (including un-fully vaccinated, fully vaccinated, and booster vaccinated). These lines depicts how coverage changes with optimized vaccination process. The line increased from the first dose gives the specific information about which should be vaccinated first.

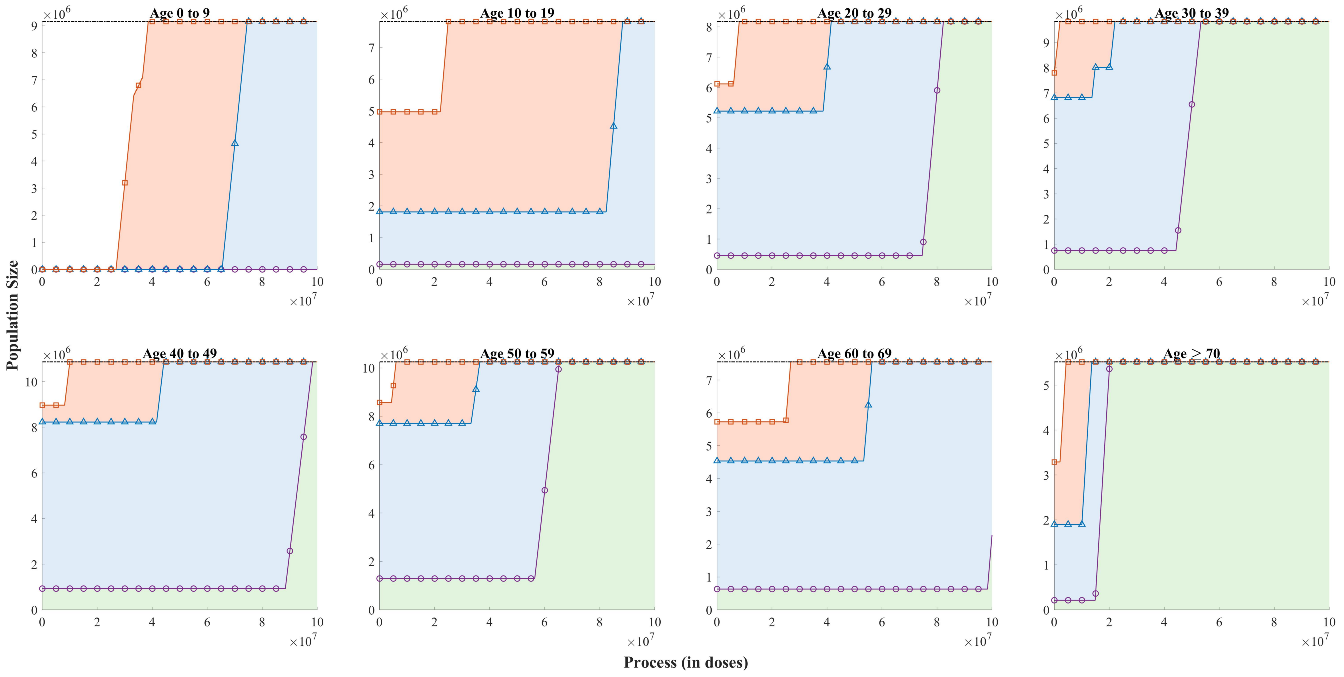

Figure. S65: Optimized Vaccinating Strategy Under Current Contact Pattern and Vaccine Coverage. For minimizing hospitalization of Omicron variant with $R_0 = 3$. 8 subfigures represent the optimal vaccination process in 8 age groups. The x-axis represent the dose-wise vaccination process; y-axis represents vaccine coverage inside the age group (populatioon size of four vaccination status). The purple line with circles denote the population size of booster vaccinated; the blue line with triangles denote the population size of at least fully-vaccinated (including fully vaccinated and booster vaccinated); the red line with squares denote the population size of at least vaccinated (including un-fully vaccinated, fully vaccinated, and booster vaccinated). These lines depicts how coverage changes with optimized vaccination process. The line increased from the first dose gives the specific information about which should be vaccinated first.

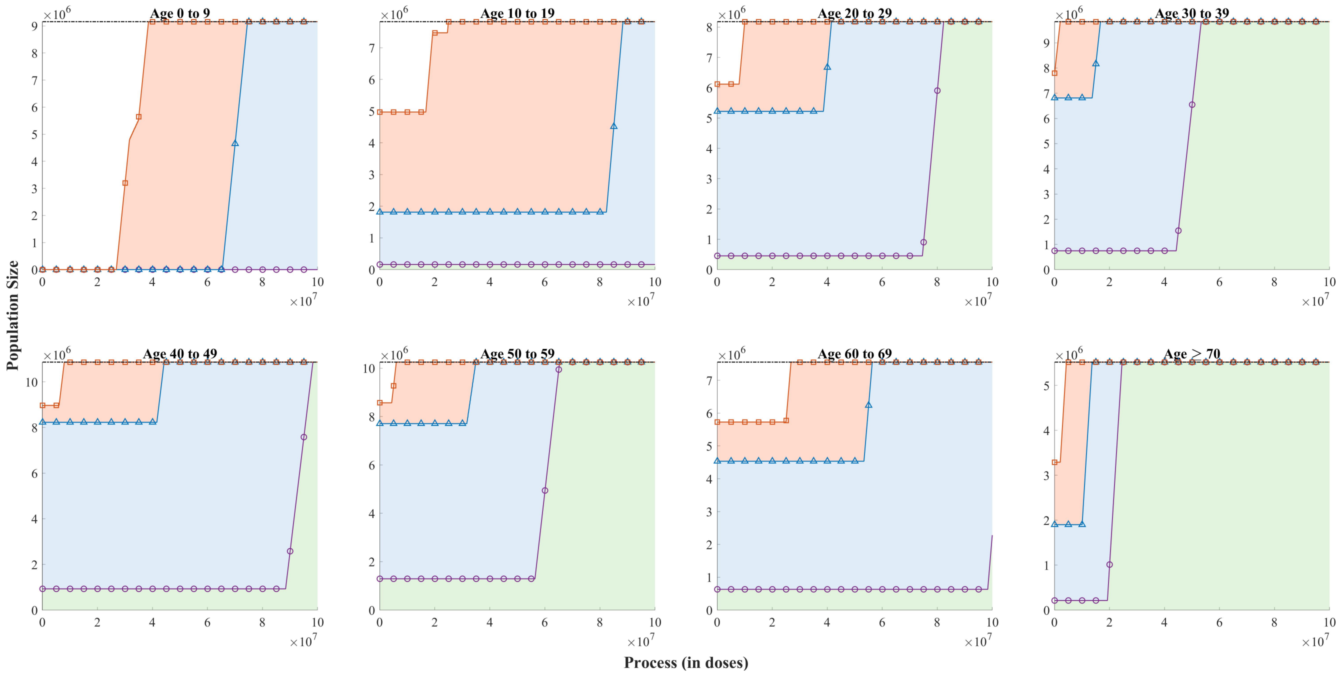

Figure. S66: Optimized Vaccinating Strategy Under Current Contact Pattern and Vaccine Coverage. For minimizing hospitalization of Omicron variant with $R_0 = 4$. 8 subfigures represent the optimal vaccination process in 8 age groups. The x-axis represent the dose-wise vaccination process; y-axis represents vaccine coverage inside the age group (populatioon size of four vaccination status). The purple line with circles denote the population size of booster vaccinated; the blue line with triangles denote the population size of at least fully-vaccinated (including fully vaccinated and booster vaccinated); the red line with squares denote the population size of at least vaccinated (including un-fully vaccinated, fully vaccinated, and booster vaccinated). These lines depicts how coverage changes with optimized vaccination process. The line increased from the first dose gives the specific information about which should be vaccinated first.

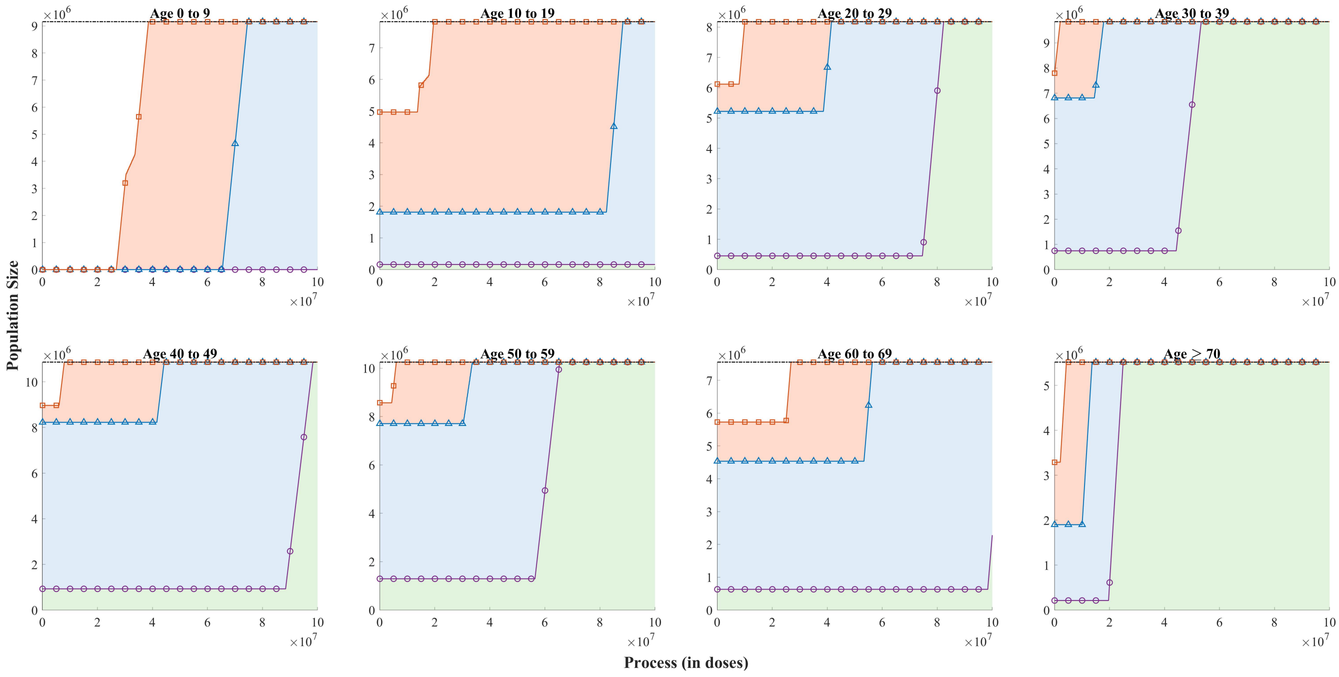

Figure. S67: Optimized Vaccinating Strategy Under Current Contact Pattern and Vaccine Coverage. For minimizing hospitalization of Omicron variant with $R_0 = 5$. 8 subfigures represent the optimal vaccination process in 8 age groups. The x-axis represent the dose-wise vaccination process; y-axis represents vaccine coverage inside the age group (populatioon size of four vaccination status). The purple line with circles denote the population size of booster vaccinated; the blue line with triangles denote the population size of at least fully-vaccinated (including fully vaccinated and booster vaccinated); the red line with squares denote the population size of at least vaccinated (including un-fully vaccinated, fully vaccinated, and booster vaccinated). These lines depicts how coverage changes with optimized vaccination process. The line increased from the first dose gives the specific information about which should be vaccinated first.

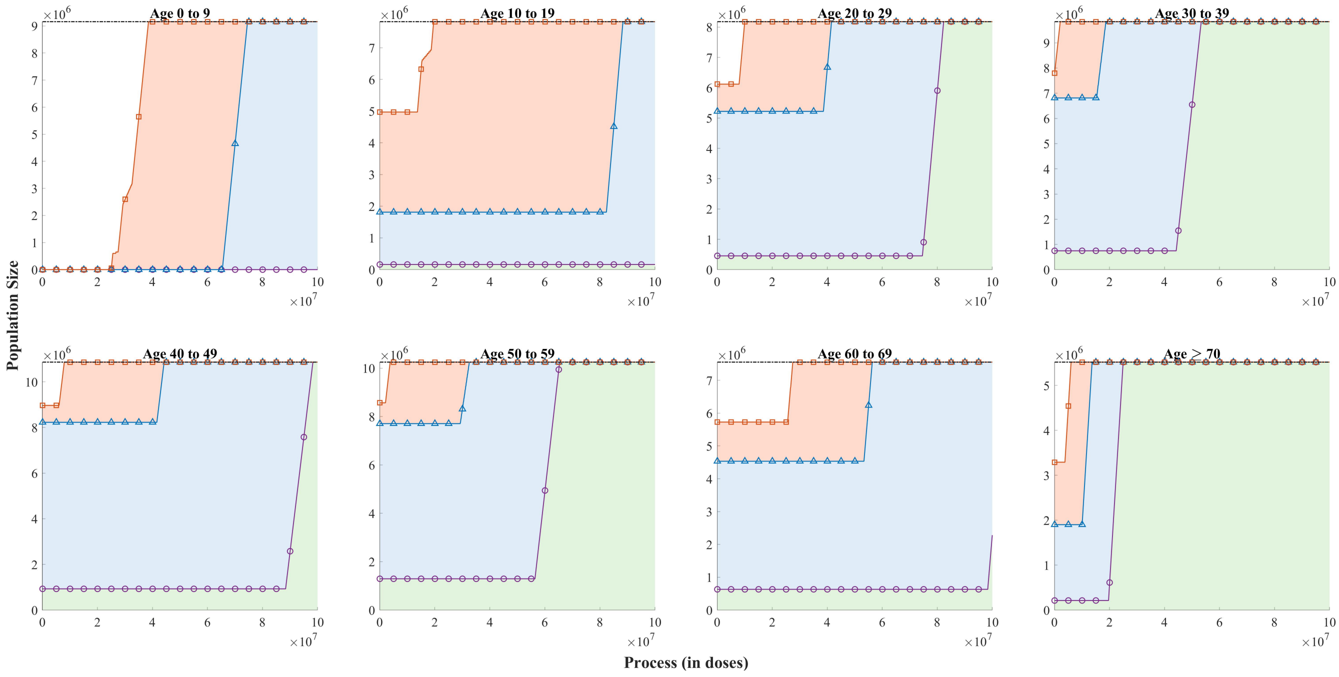

Figure. S68: Optimized Vaccinating Strategy Under Current Contact Pattern and Vaccine Coverage. For minimizing hospitalization of Omicron variant with $R_0 = 6$. 8 subfigures represent the optimal vaccination process in 8 age groups. The x-axis represent the dose-wise vaccination process; y-axis represents vaccine coverage inside the age group (populatioon size of four vaccination status). The purple line with circles denote the population size of booster vaccinated; the blue line with triangles denote the population size of at least fully-vaccinated (including fully vaccinated and booster vaccinated); the red line with squares denote the population size of at least vaccinated (including un-fully vaccinated, fully vaccinated, and booster vaccinated). These lines depicts how coverage changes with optimized vaccination process. The line increased from the first dose gives the specific information about which should be vaccinated first.

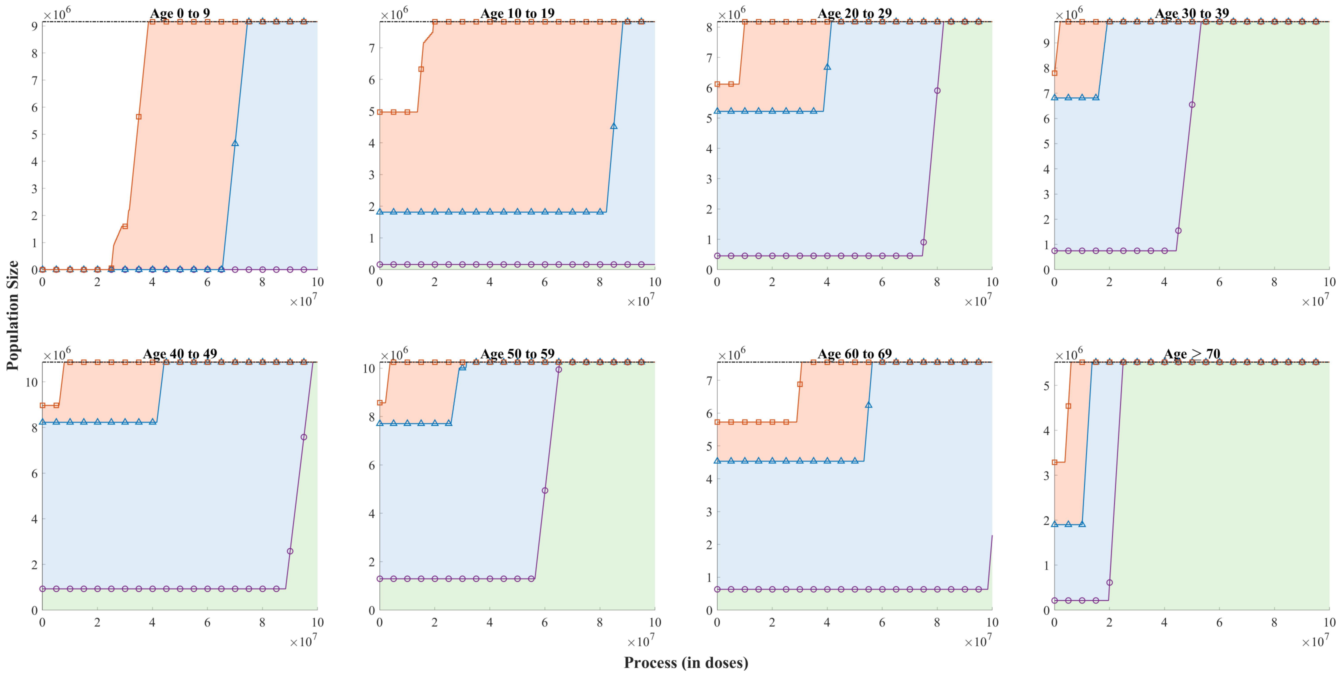

Figure. S69: Optimized Vaccinating Strategy Under Current Contact Pattern and Vaccine Coverage. For minimizing hospitalization of Omicron variant with $R_0 = 7$. 8 subfigures represent the optimal vaccination process in 8 age groups. The x-axis represent the dose-wise vaccination process; y-axis represents vaccine coverage inside the age group (populatioon size of four vaccination status). The purple line with circles denote the population size of booster vaccinated; the blue line with triangles denote the population size of at least fully-vaccinated (including fully vaccinated and booster vaccinated); the red line with squares denote the population size of at least vaccinated (including un-fully vaccinated, fully vaccinated, and booster vaccinated). These lines depicts how coverage changes with optimized vaccination process. The line increased from the first dose gives the specific information about which should be vaccinated first.

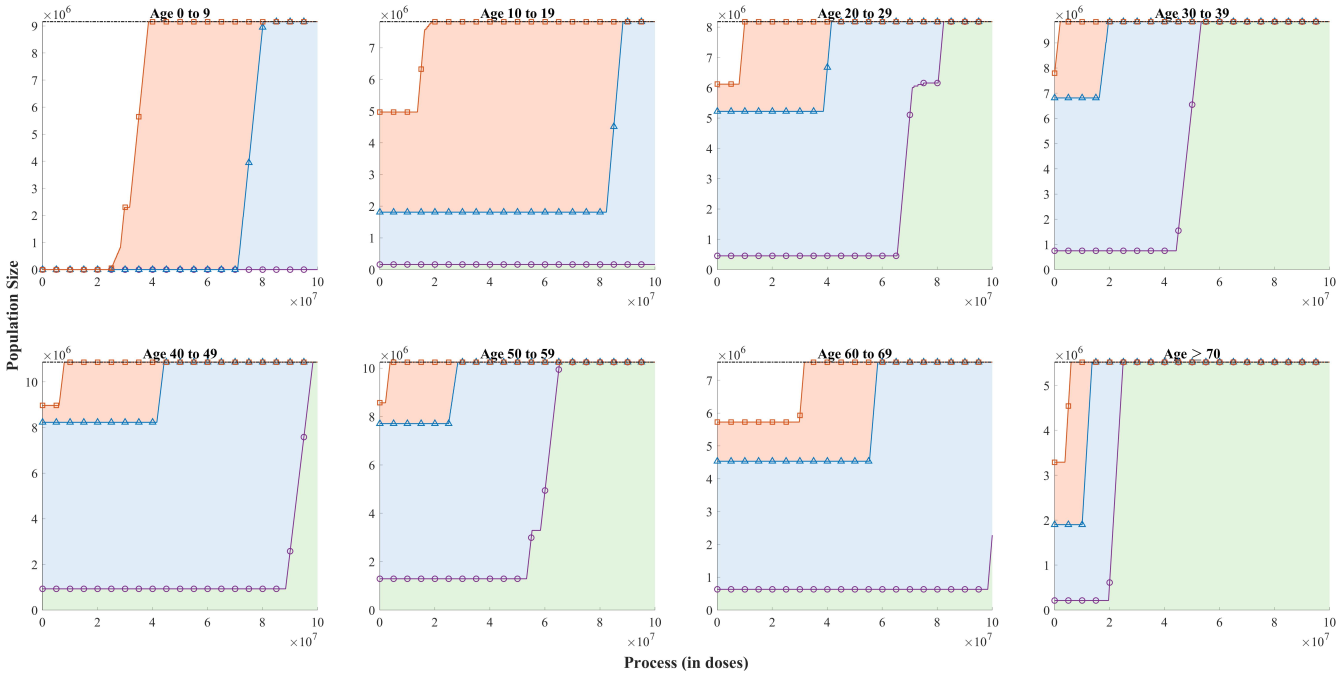

Figure. S70: Optimized Vaccinating Strategy Under Current Contact Pattern and Vaccine Coverage. For minimizing hospitalization of Omicron variant with $R_0 = 8$. 8 subfigures represent the optimal vaccination process in 8 age groups. The x-axis represent the dose-wise vaccination process; y-axis represents vaccine coverage inside the age group (populatioon size of four vaccination status). The purple line with circles denote the population size of booster vaccinated; the blue line with triangles denote the population size of at least fully-vaccinated (including fully vaccinated and booster vaccinated); the red line with squares denote the population size of at least vaccinated (including un-fully vaccinated, fully vaccinated, and booster vaccinated). These lines depicts how coverage changes with optimized vaccination process. The line increased from the first dose gives the specific information about which should be vaccinated first.

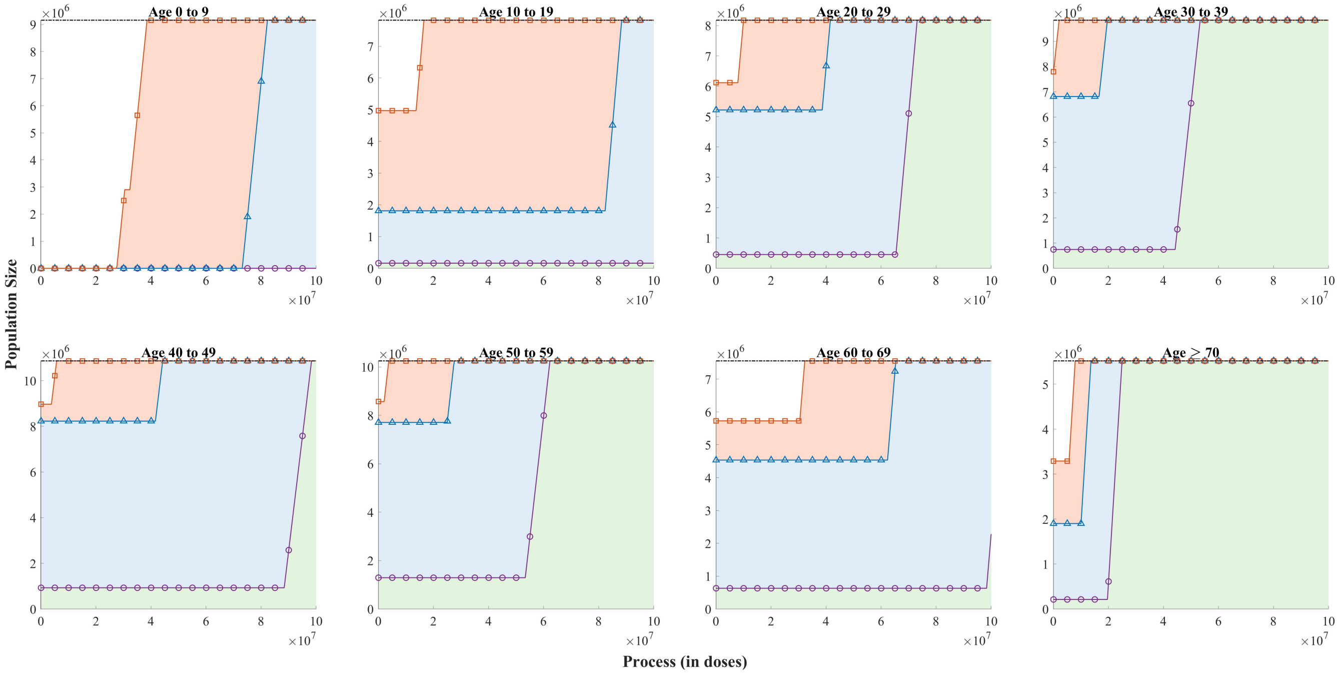

Figure. S71: Optimized Vaccinating Strategy Under Current Contact Pattern and Vaccine Coverage. For minimizing hospitalization of Omicron variant with $R_0 = 9$. 8 subfigures represent the optimal vaccination process in 8 age groups. The x-axis represent the dose-wise vaccination process; y-axis represents vaccine coverage inside the age group (populatioon size of four vaccination status). The purple line with circles denote the population size of booster vaccinated; the blue line with triangles denote the population size of at least fully-vaccinated (including fully vaccinated and booster vaccinated); the red line with squares denote the population size of at least vaccinated (including un-fully vaccinated, fully vaccinated, and booster vaccinated). These lines depicts how coverage changes with optimized vaccination process. The line increased from the first dose gives the specific information about which should be vaccinated first.

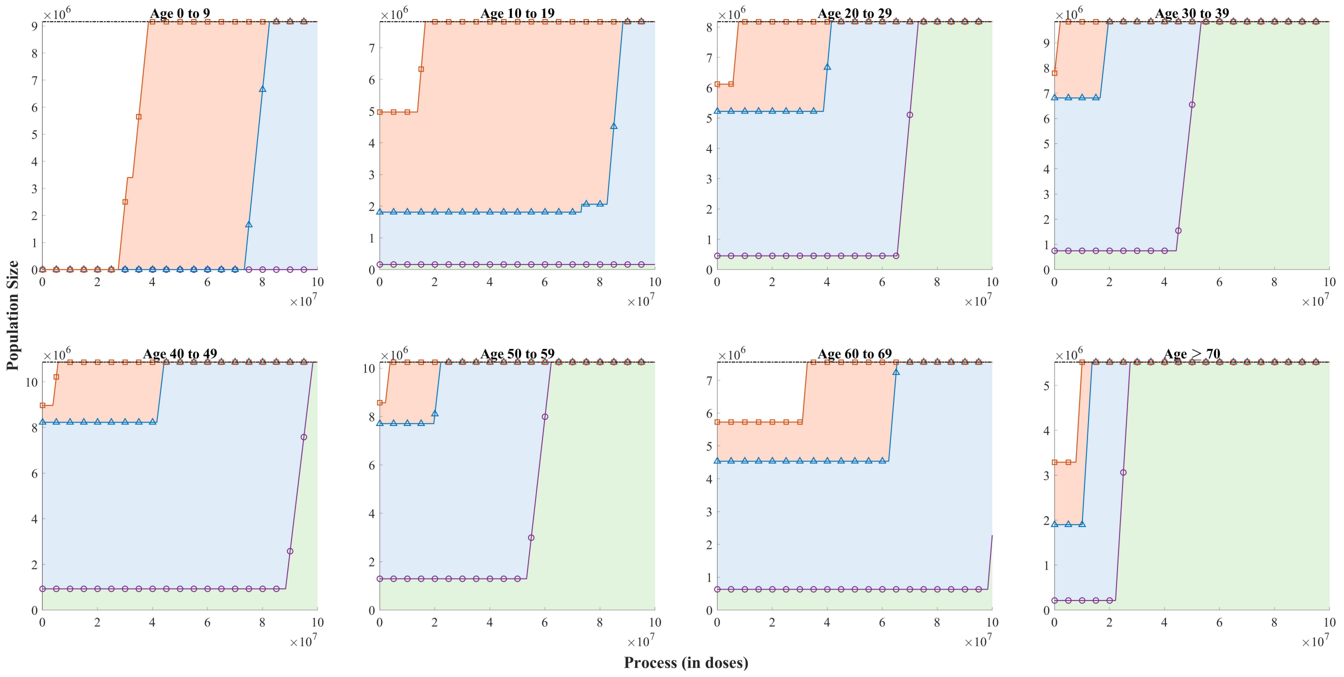

Figure. S72: Optimized Vaccinating Strategy Under Current Contact Pattern and Vaccine Coverage. For minimizing hospitalization of Omicron variant with $R_0 = 10$. 8 subfigures represent the optimal vaccination process in 8 age groups. The x-axis represent the dose-wise vaccination process; y-axis represents vaccine coverage inside the age group (populatioon size of four vaccination status). The purple line with circles denote the population size of booster vaccinated; the blue line with triangles denote the population size of at least fully-vaccinated (including fully vaccinated and booster vaccinated); the red line with squares denote the population size of at least vaccinated (including un-fully vaccinated, fully vaccinated, and booster vaccinated). These lines depicts how coverage changes with optimized vaccination process. The line increased from the first dose gives the specific information about which should be vaccinated first.

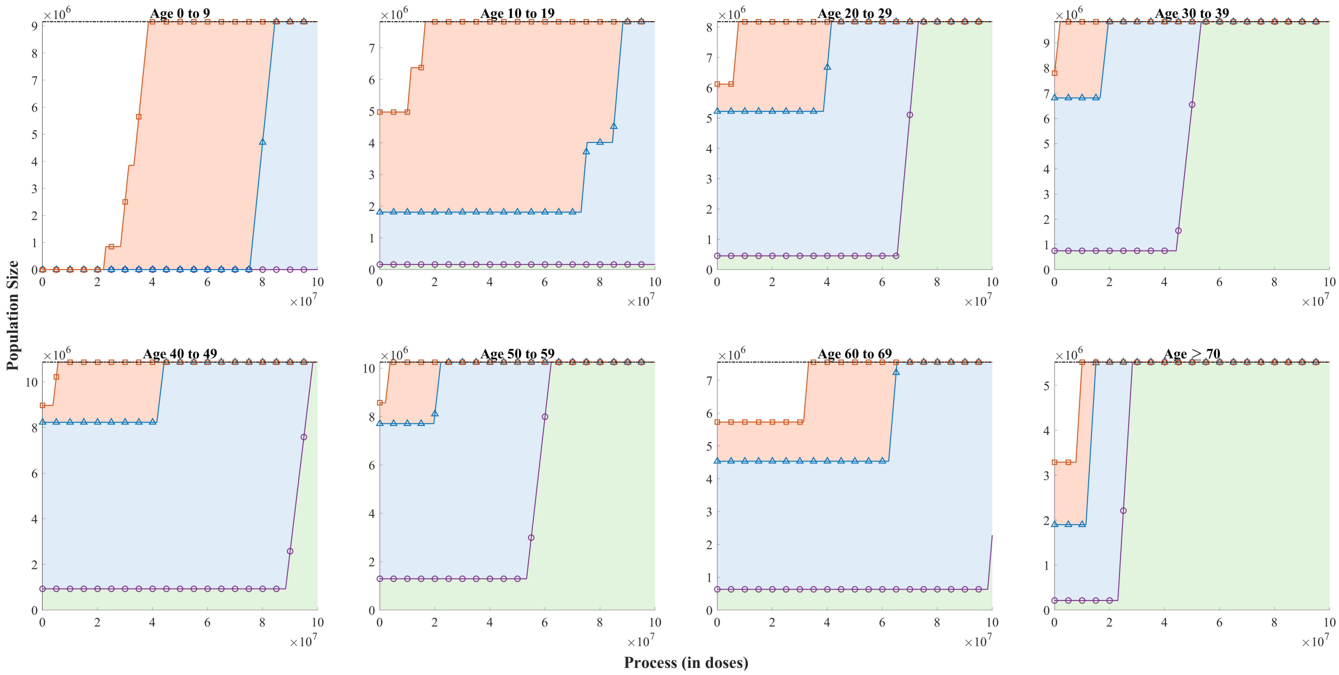

Figure. S73: Optimized Vaccinating Strategy Under Current Contact Pattern and Vaccine Coverage. For minimizing hospitalization of Omicron variant with $R_0 = 11$. 8 subfigures represent the optimal vaccination process in 8 age groups. The x-axis represent the dose-wise vaccination process; y-axis represents vaccine coverage inside the age group (populatioon size of four vaccination status). The purple line with circles denote the population size of booster vaccinated; the blue line with triangles denote the population size of at least fully-vaccinated (including fully vaccinated and booster vaccinated); the red line with squares denote the population size of at least vaccinated (including un-fully vaccinated, fully vaccinated, and booster vaccinated). These lines depicts how coverage changes with optimized vaccination process. The line increased from the first dose gives the specific information about which should be vaccinated first.

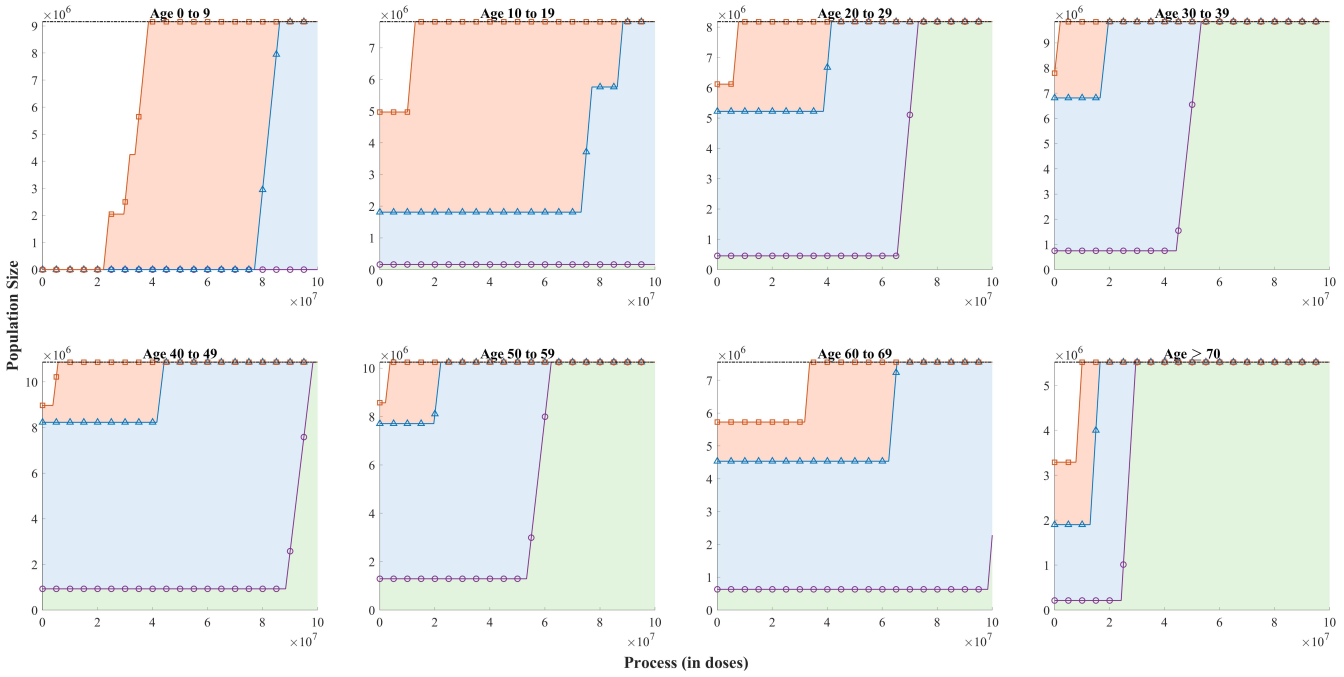

Figure. S74: Optimized Vaccinating Strategy Under Current Contact Pattern and Vaccine Coverage. For minimizing hospitalization of Omicron variant with $R_0 = 12$. 8 subfigures represent the optimal vaccination process in 8 age groups. The x-axis represent the dose-wise vaccination process; y-axis represents vaccine coverage inside the age group (populatioon size of four vaccination status). The purple line with circles denote the population size of booster vaccinated; the blue line with triangles denote the population size of at least fully-vaccinated (including fully vaccinated and booster vaccinated); the red line with squares denote the population size of at least vaccinated (including un-fully vaccinated, fully vaccinated, and booster vaccinated). These lines depicts how coverage changes with optimized vaccination process. The line increased from the first dose gives the specific information about which should be vaccinated first.

With the optimal vaccination process, the fatality in whole population within 14 days from the first illness onset is shown in figure 4.5.1.

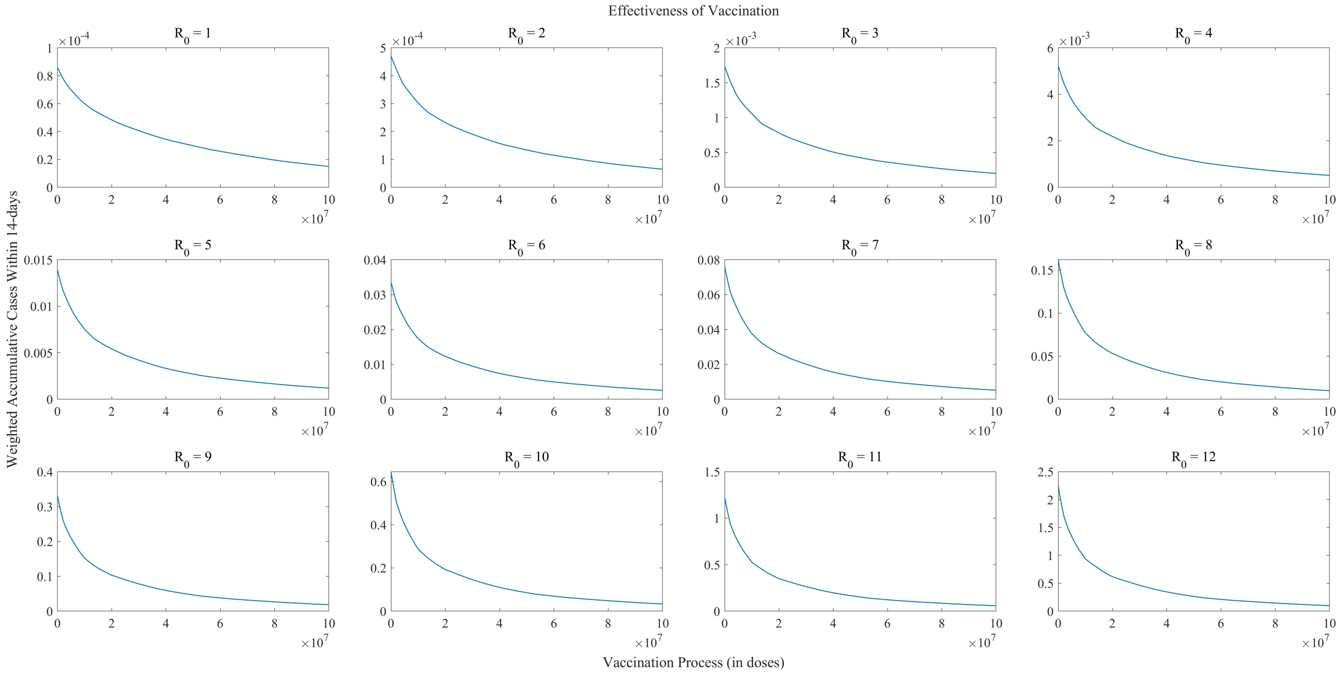

Figure. S75: Effectiveness of Optimized Vaccination. Under Current Contact Pattern and Vaccine Coverage. With parameters of Delta variant.

4.5.6 Fatality for Omicron Variant

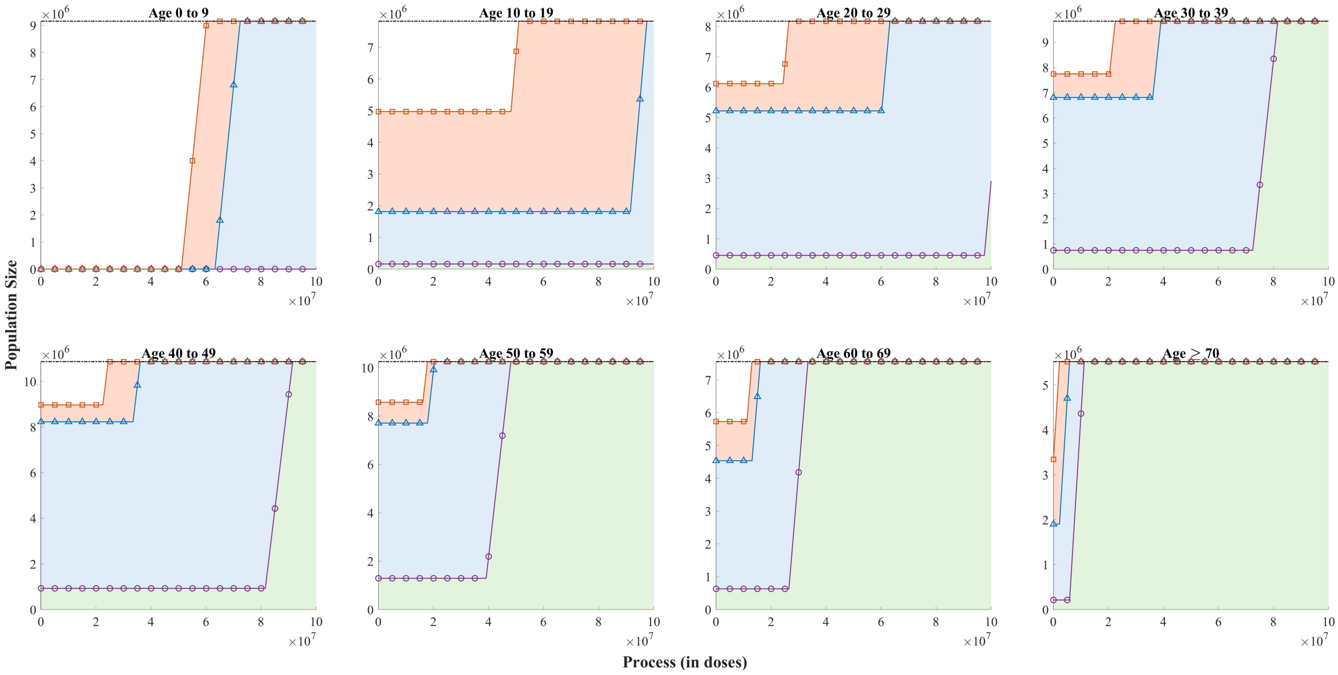

Figure. S76: Optimized Vaccinating Strategy Under Current Contact Pattern and Vaccine Coverage. For minimizing fatality of Omicron variant with $R_0 = 1$. 8 subfigures represent the optimal vaccination process in 8 age groups. The x-axis represent the dose-wise vaccination process; y-axis represents vaccine coverage inside the age group (populatioon size of four vaccination status). The purple line with circles denote the population size of booster vaccinated; the blue line with triangles denote the population size of at least fully-vaccinated (including fully vaccinated and booster vaccinated); the red line with squares denote the population size of at least vaccinated (including un-fully vaccinated, fully vaccinated, and booster vaccinated). These lines depicts how coverage changes with optimized vaccination process. The line increased from the first dose gives the specific information about which should be vaccinated first.

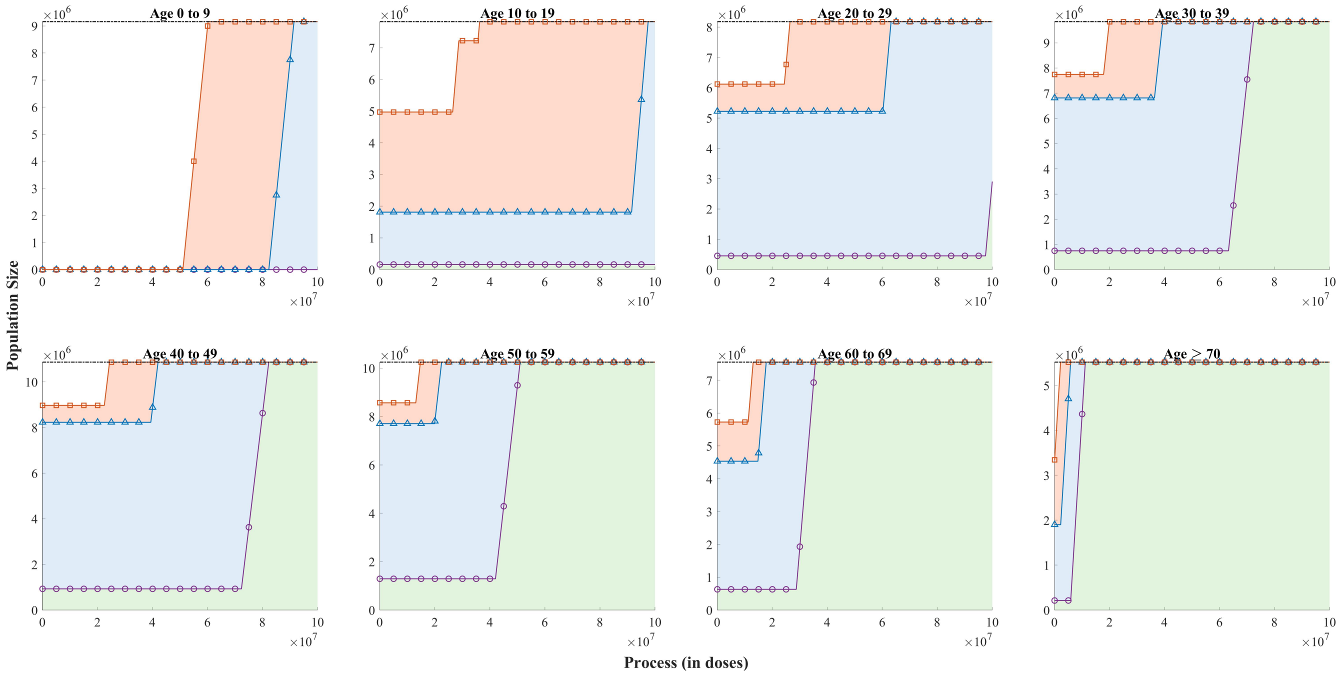

Figure. S77: Optimized Vaccinating Strategy Under Current Contact Pattern and Vaccine Coverage. For minimizing fatality of Omicron variant with $R_0 = 2$. 8 subfigures represent the optimal vaccination process in 8 age groups. The x-axis represent the dose-wise vaccination process; y-axis represents vaccine coverage inside the age group (populatioon size of four vaccination status). The purple line with circles denote the population size of booster vaccinated; the blue line with triangles denote the population size of at least fully-vaccinated (including fully vaccinated and booster vaccinated); the red line with squares denote the population size of at least vaccinated (including un-fully vaccinated, fully vaccinated, and booster vaccinated). These lines depicts how coverage changes with optimized vaccination process. The line increased from the first dose gives the specific information about which should be vaccinated first.

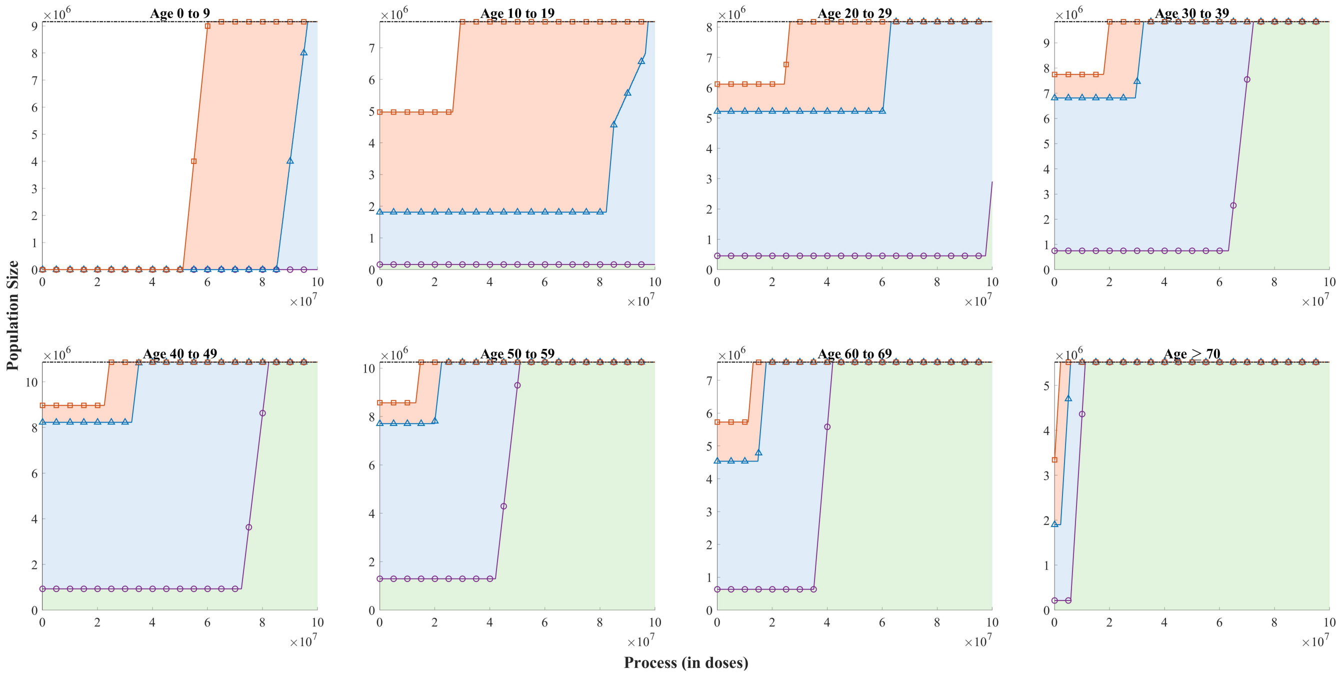

Figure. S78: Optimized Vaccinating Strategy Under Current Contact Pattern and Vaccine Coverage. For minimizing fatality of Omicron variant with $R_0 = 3$. 8 subfigures represent the optimal vaccination process in 8 age groups. The x-axis represent the dose-wise vaccination process; y-axis represents vaccine coverage inside the age group (populatioon size of four vaccination status). The purple line with circles denote the population size of booster vaccinated; the blue line with triangles denote the population size of at least fully-vaccinated (including fully vaccinated and booster vaccinated); the red line with squares denote the population size of at least vaccinated (including un-fully vaccinated, fully vaccinated, and booster vaccinated). These lines depicts how coverage changes with optimized vaccination process. The line increased from the first dose gives the specific information about which should be vaccinated first.

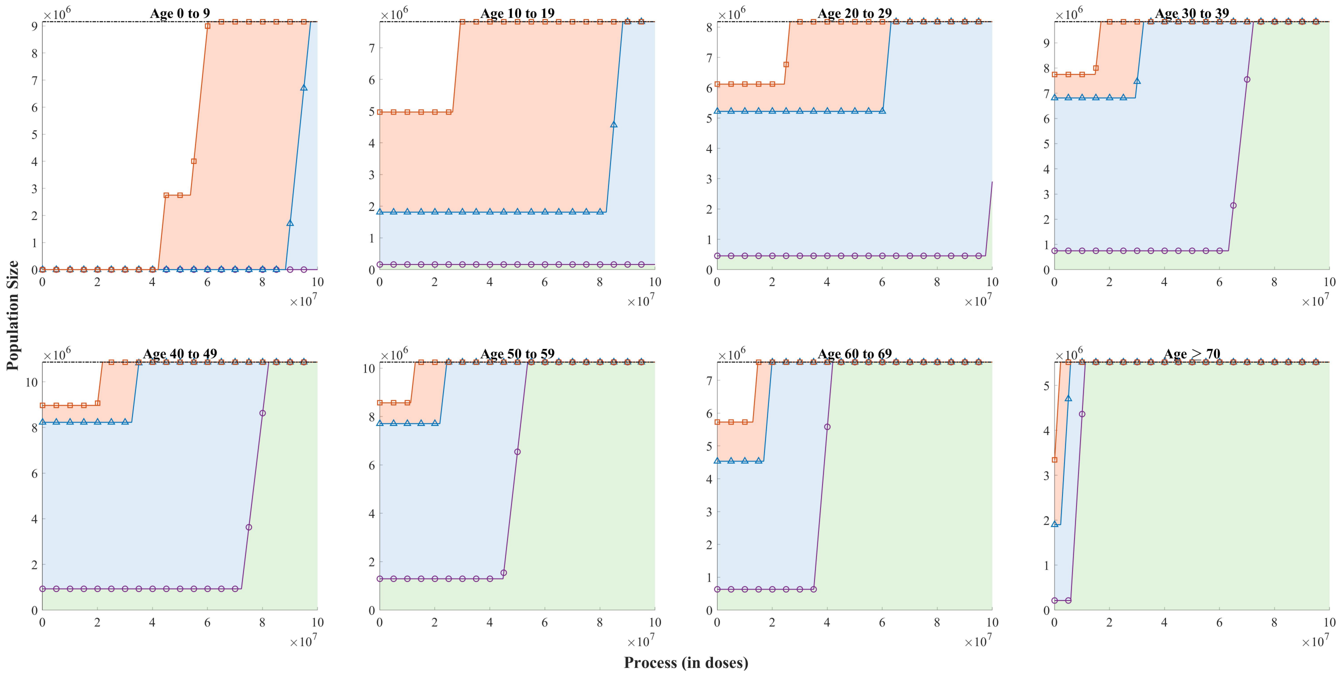

Figure. S79: Optimized Vaccinating Strategy Under Current Contact Pattern and Vaccine Coverage. For minimizing fatality of Omicron variant with $R_0 = 4$. 8 subfigures represent the optimal vaccination process in 8 age groups. The x-axis represent the dose-wise vaccination process; y-axis represents vaccine coverage inside the age group (populatioon size of four vaccination status). The purple line with circles denote the population size of booster vaccinated; the blue line with triangles denote the population size of at least fully-vaccinated (including fully vaccinated and booster vaccinated); the red line with squares denote the population size of at least vaccinated (including un-fully vaccinated, fully vaccinated, and booster vaccinated). These lines depicts how coverage changes with optimized vaccination process. The line increased from the first dose gives the specific information about which should be vaccinated first.

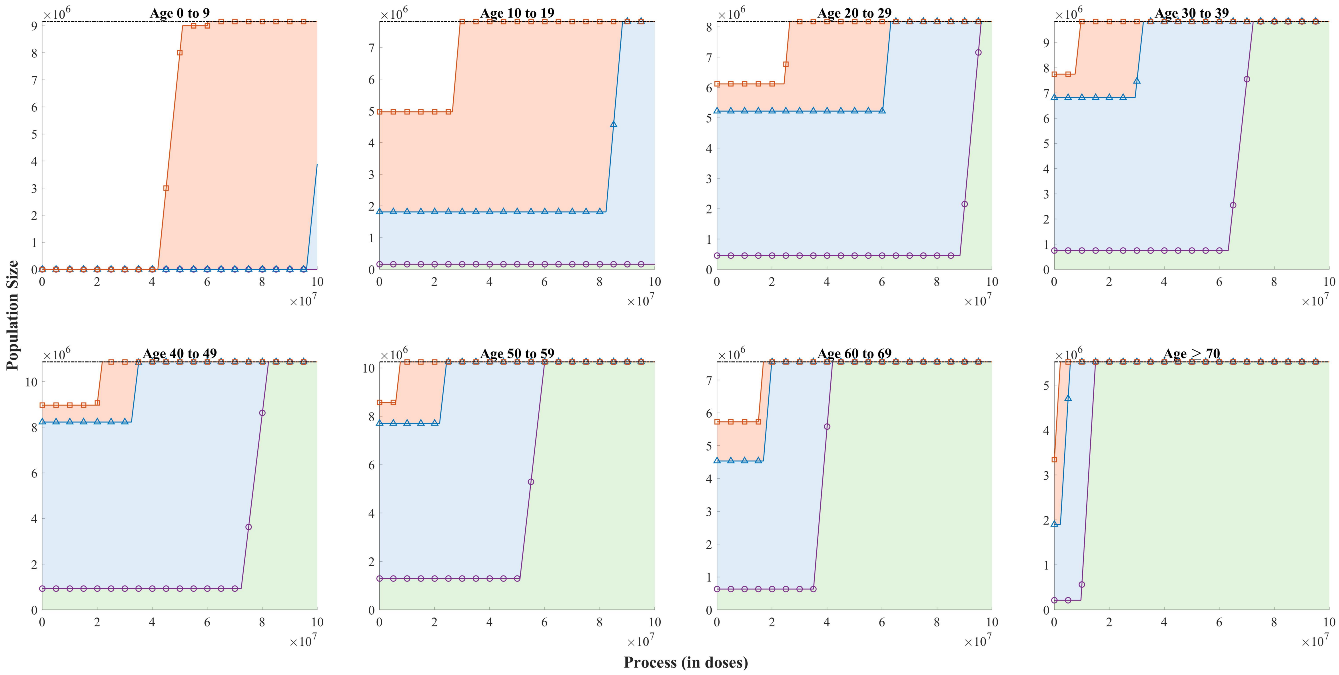

Figure. S80: Optimized Vaccinating Strategy Under Current Contact Pattern and Vaccine Coverage. For minimizing fatality of Omicron variant with $R_0 = 5$. 8 subfigures represent the optimal vaccination process in 8 age groups. The x-axis represent the dose-wise vaccination process; y-axis represents vaccine coverage inside the age group (populatioon size of four vaccination status). The purple line with circles denote the population size of booster vaccinated; the blue line with triangles denote the population size of at least fully-vaccinated (including fully vaccinated and booster vaccinated); the red line with squares denote the population size of at least vaccinated (including un-fully vaccinated, fully vaccinated, and booster vaccinated). These lines depicts how coverage changes with optimized vaccination process. The line increased from the first dose gives the specific information about which should be vaccinated first.

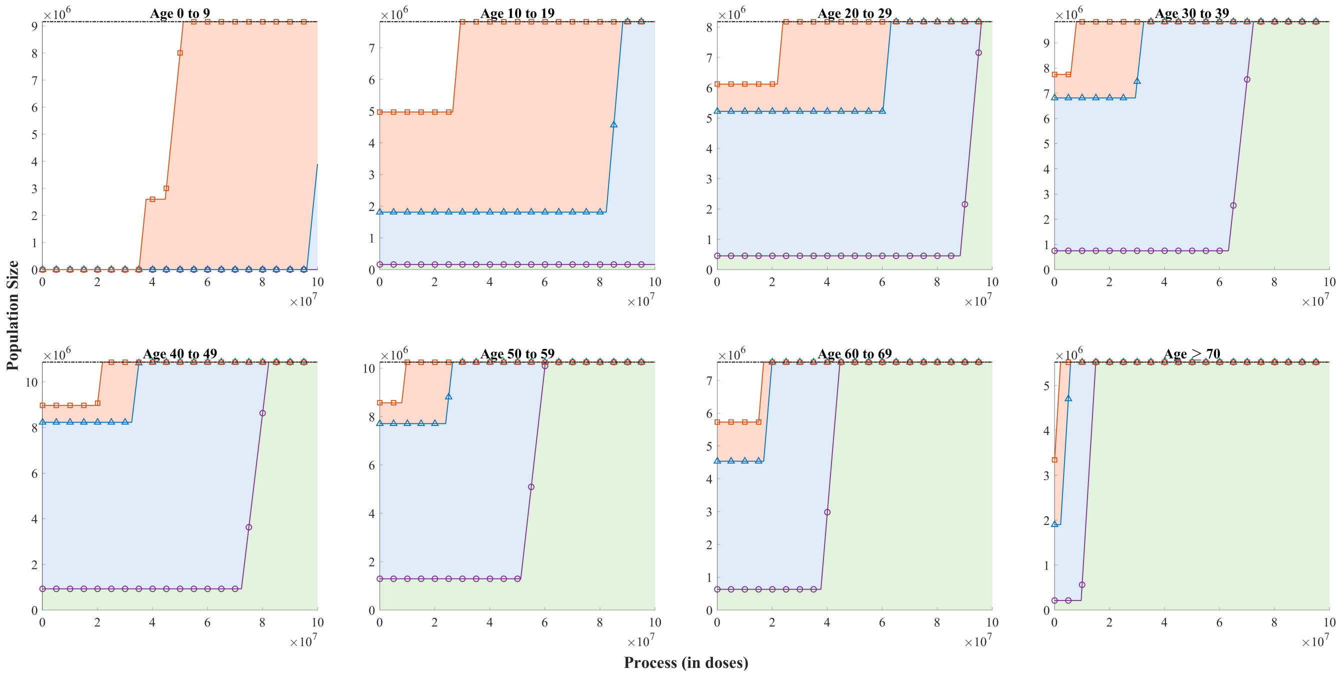

Figure. S81: Optimized Vaccinating Strategy Under Current Contact Pattern and Vaccine Coverage. For minimizing fatality of Omicron variant with $R_0 = 6$. 8 subfigures represent the optimal vaccination process in 8 age groups. The x-axis represent the dose-wise vaccination process; y-axis represents vaccine coverage inside the age group (populatioon size of four vaccination status). The purple line with circles denote the population size of booster vaccinated; the blue line with triangles denote the population size of at least fully-vaccinated (including fully vaccinated and booster vaccinated); the red line with squares denote the population size of at least vaccinated (including un-fully vaccinated, fully vaccinated, and booster vaccinated). These lines depicts how coverage changes with optimized vaccination process. The line increased from the first dose gives the specific information about which should be vaccinated first.

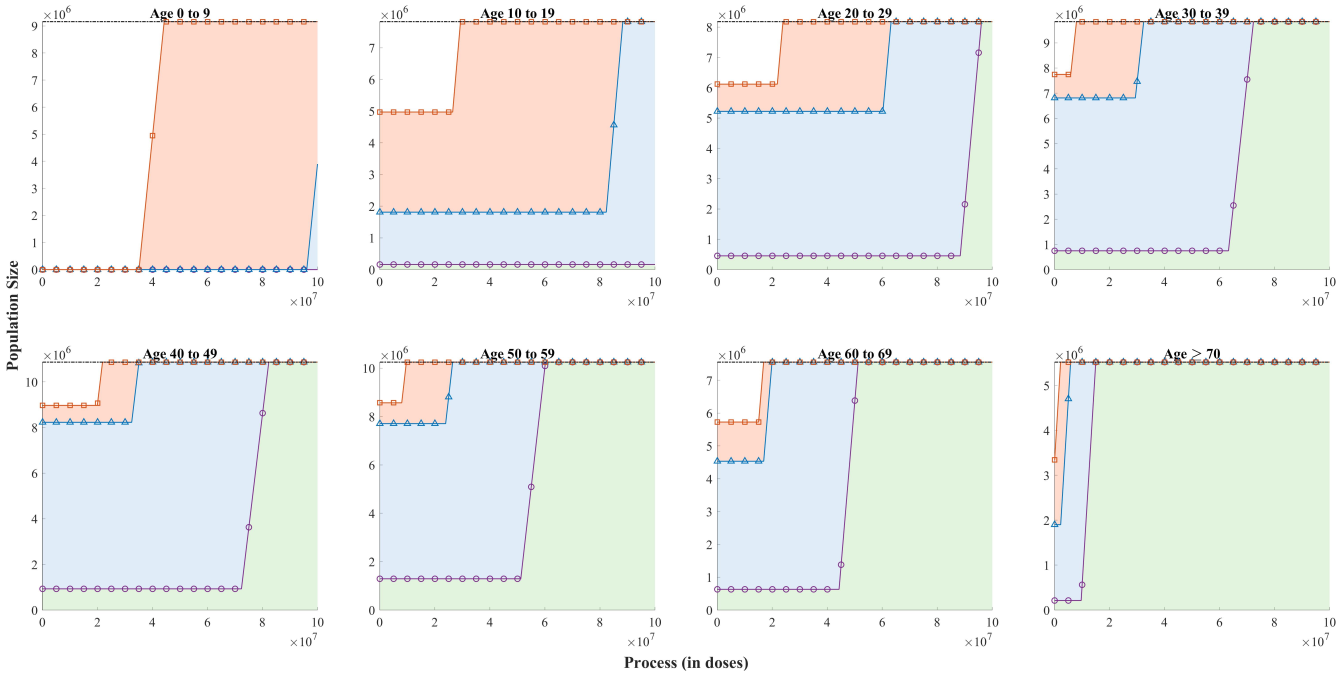

Figure. S82: Optimized Vaccinating Strategy Under Current Contact Pattern and Vaccine Coverage. For minimizing fatality of Omicron variant with $R_0 = 7$. 8 subfigures represent the optimal vaccination process in 8 age groups. The x-axis represent the dose-wise vaccination process; y-axis represents vaccine coverage inside the age group (populatioon size of four vaccination status). The purple line with circles denote the population size of booster vaccinated; the blue line with triangles denote the population size of at least fully-vaccinated (including fully vaccinated and booster vaccinated); the red line with squares denote the population size of at least vaccinated (including un-fully vaccinated, fully vaccinated, and booster vaccinated). These lines depicts how coverage changes with optimized vaccination process. The line increased from the first dose gives the specific information about which should be vaccinated first.

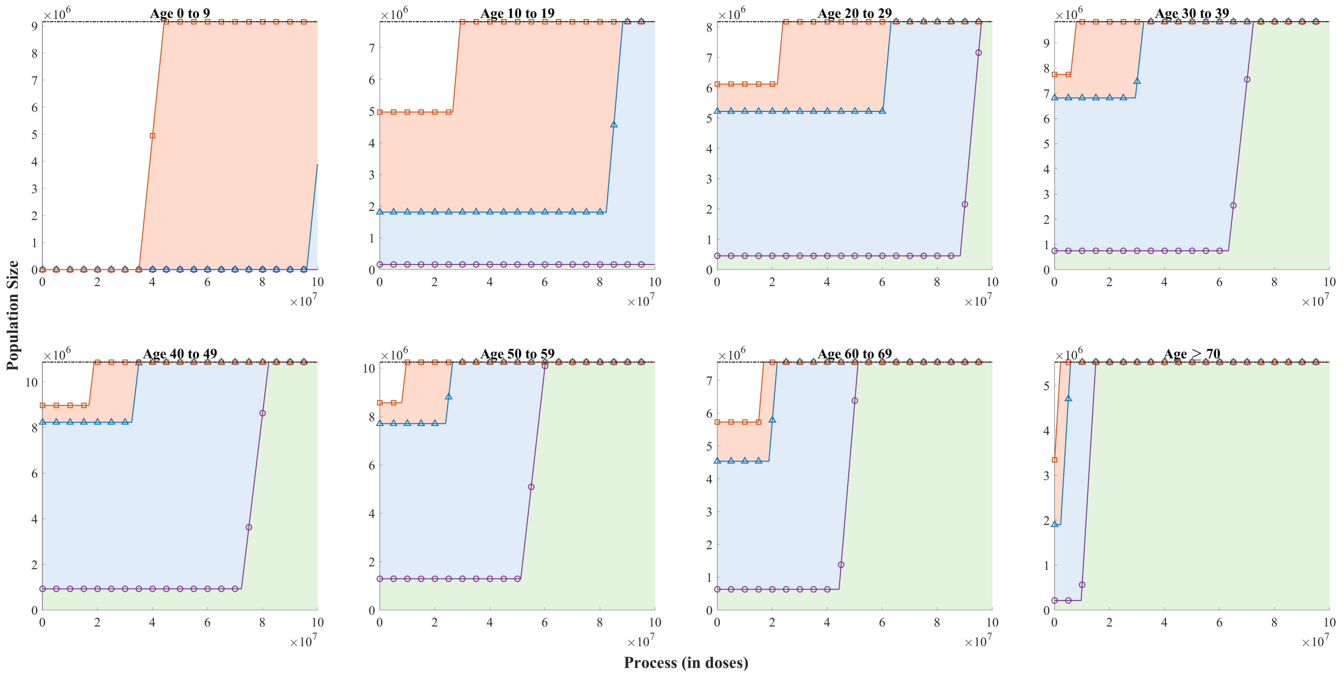

Figure. S83: Optimized Vaccinating Strategy Under Current Contact Pattern and Vaccine Coverage. For minimizing fatality of Omicron variant with $R_0 = 8$. 8 subfigures represent the optimal vaccination process in 8 age groups. The x-axis represent the dose-wise vaccination process; y-axis represents vaccine coverage inside the age group (populatioon size of four vaccination status). The purple line with circles denote the population size of booster vaccinated; the blue line with triangles denote the population size of at least fully-vaccinated (including fully vaccinated and booster vaccinated); the red line with squares denote the population size of at least vaccinated (including un-fully vaccinated, fully vaccinated, and booster vaccinated). These lines depicts how coverage changes with optimized vaccination process. The line increased from the first dose gives the specific information about which should be vaccinated first.

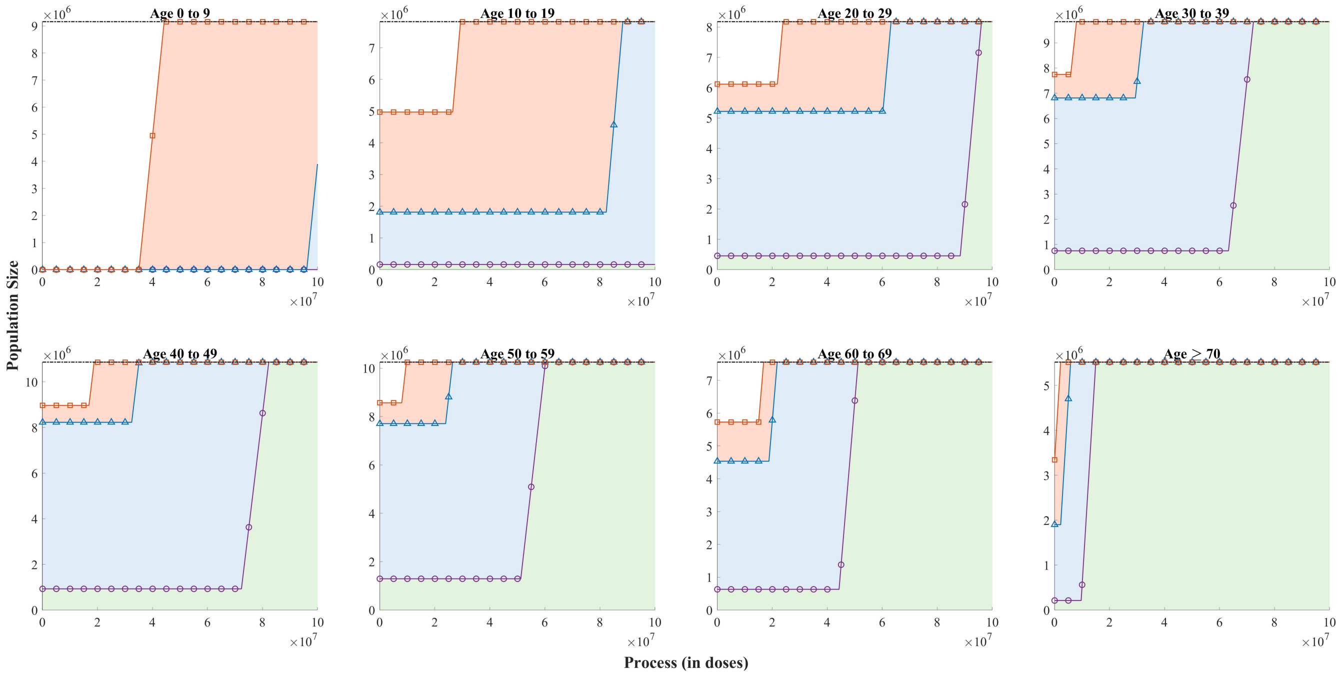

Figure. S84: Optimized Vaccinating Strategy Under Current Contact Pattern and Vaccine Coverage. For minimizing fatality of Omicron variant with $R_0 = 9$. 8 subfigures represent the optimal vaccination process in 8 age groups. The x-axis represent the dose-wise vaccination process; y-axis represents vaccine coverage inside the age group (populatioon size of four vaccination status). The purple line with circles denote the population size of booster vaccinated; the blue line with triangles denote the population size of at least fully-vaccinated (including fully vaccinated and booster vaccinated); the red line with squares denote the population size of at least vaccinated (including un-fully vaccinated, fully vaccinated, and booster vaccinated). These lines depicts how coverage changes with optimized vaccination process. The line increased from the first dose gives the specific information about which should be vaccinated first.

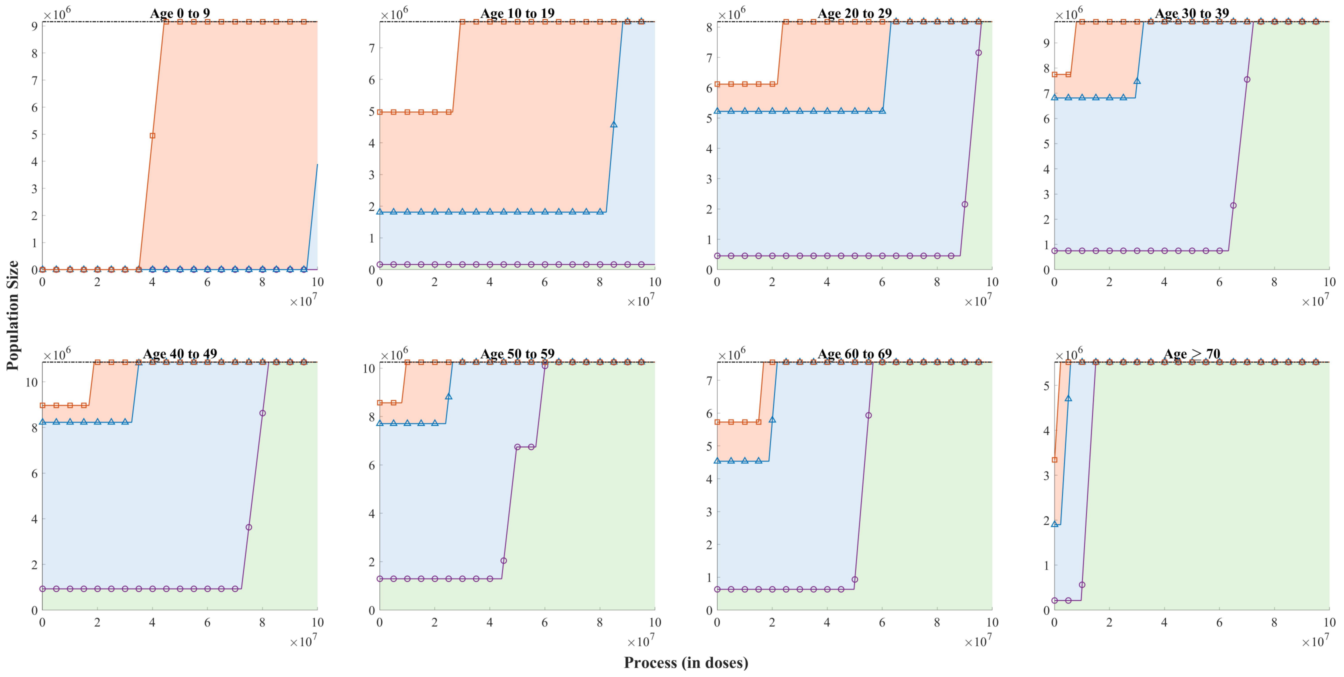

Figure. S85: Optimized Vaccinating Strategy Under Current Contact Pattern and Vaccine Coverage. For minimizing fatality of Omicron variant with $R_0 = 10$. 8 subfigures represent the optimal vaccination process in 8 age groups. The x-axis represent the dose-wise vaccination process; y-axis represents vaccine coverage inside the age group (populatioon size of four vaccination status). The purple line with circles denote the population size of booster vaccinated; the blue line with triangles denote the population size of at least fully-vaccinated (including fully vaccinated and booster vaccinated); the red line with squares denote the population size of at least vaccinated (including un-fully vaccinated, fully vaccinated, and booster vaccinated). These lines depicts how coverage changes with optimized vaccination process. The line increased from the first dose gives the specific information about which should be vaccinated first.

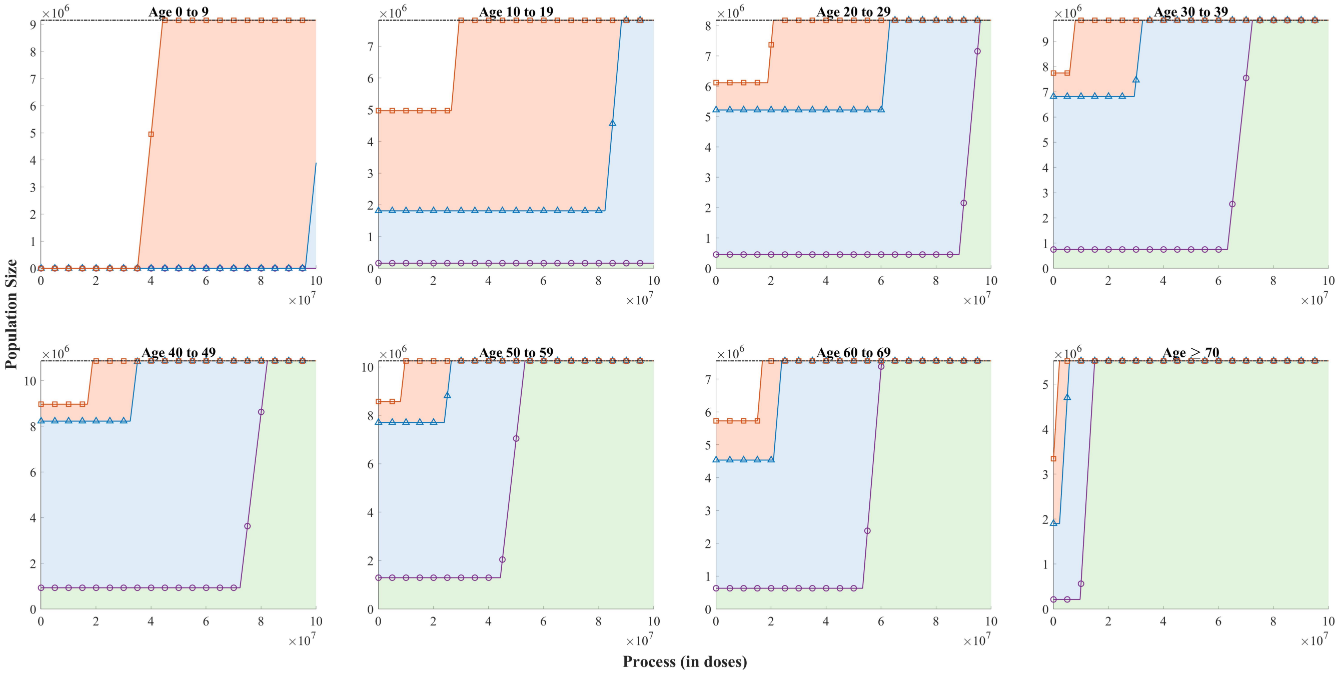

Figure. S86: Optimized Vaccinating Strategy Under Current Contact Pattern and Vaccine Coverage. For minimizing fatality of Omicron variant with $R_0 = 11$. 8 subfigures represent the optimal vaccination process in 8 age groups. The x-axis represent the dose-wise vaccination process; y-axis represents vaccine coverage inside the age group (populatioon size of four vaccination status). The purple line with circles denote the population size of booster vaccinated; the blue line with triangles denote the population size of at least fully-vaccinated (including fully vaccinated and booster vaccinated); the red line with squares denote the population size of at least vaccinated (including un-fully vaccinated, fully vaccinated, and booster vaccinated). These lines depicts how coverage changes with optimized vaccination process. The line increased from the first dose gives the specific information about which should be vaccinated first.

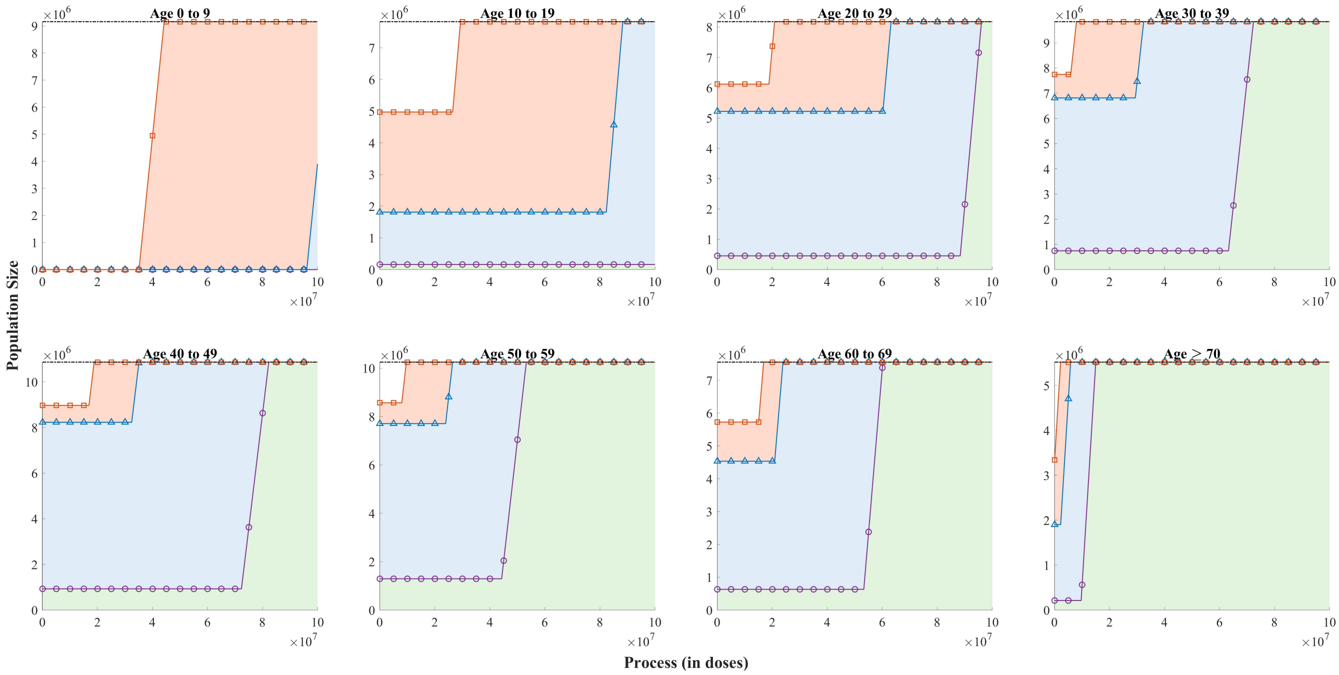

Figure. S87: Optimized Vaccinating Strategy Under Current Contact Pattern and Vaccine Coverage. For minimizing fatality of Omicron variant with $R_0 = 12$. 8 subfigures represent the optimal vaccination process in 8 age groups. The x-axis represent the dose-wise vaccination process; y-axis represents vaccine coverage inside the age group (populatioon size of four vaccination status). The purple line with circles denote the population size of booster vaccinated; the blue line with triangles denote the population size of at least fully-vaccinated (including fully vaccinated and booster vaccinated); the red line with squares denote the population size of at least vaccinated (including un-fully vaccinated, fully vaccinated, and booster vaccinated). These lines depicts how coverage changes with optimized vaccination process. The line increased from the first dose gives the specific information about which should be vaccinated first.

With the optimal vaccination process, the fatality in whole population within 14 days from the first illness onset is shown in figure 4.5.1.

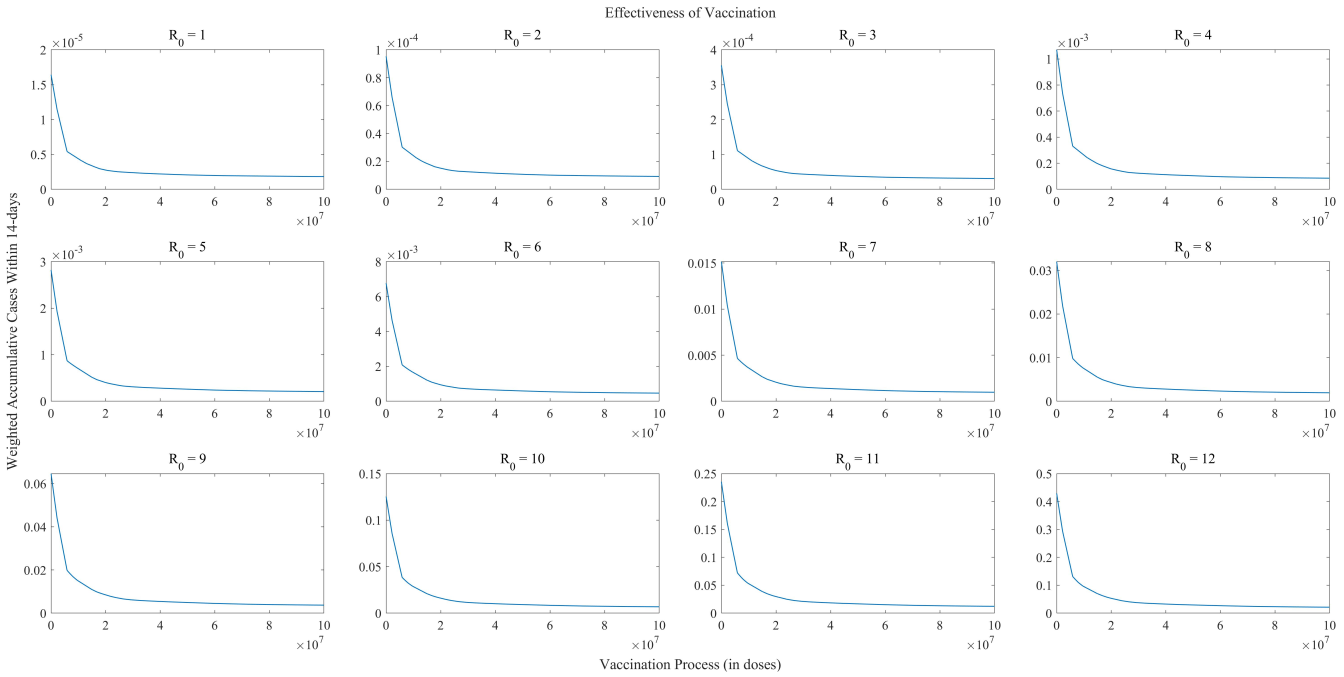

Figure. S88: Effectiveness of Optimized Vaccination. Under Current Contact Pattern and Vaccine Coverage. With parameters of Delta variant.

5 Contact Data Decomposition

We categorize the contact data into following categories:

- Public Transports
- Amusement
- School
- Household
- Hospital
- Working Area
- Service Industry
- Community

- Catering industry
- Uncategorized
- Empty (in original data)

For each category, we select and extract all case-contact data pairs occurs in the category, and trained the corresponding contact matrix via the maximum likelihood estimation proposed in Section 1. Those setting-specific matrices are given as following figures.

The unit of all matrices is (person per day). The contact matrix of all settings of individual contact per day will be a linear combination of those matrix with linear combination coefficient summing up to 1 (since in 24 hours, this man went to different places and produced close contacts in different places with different rates).

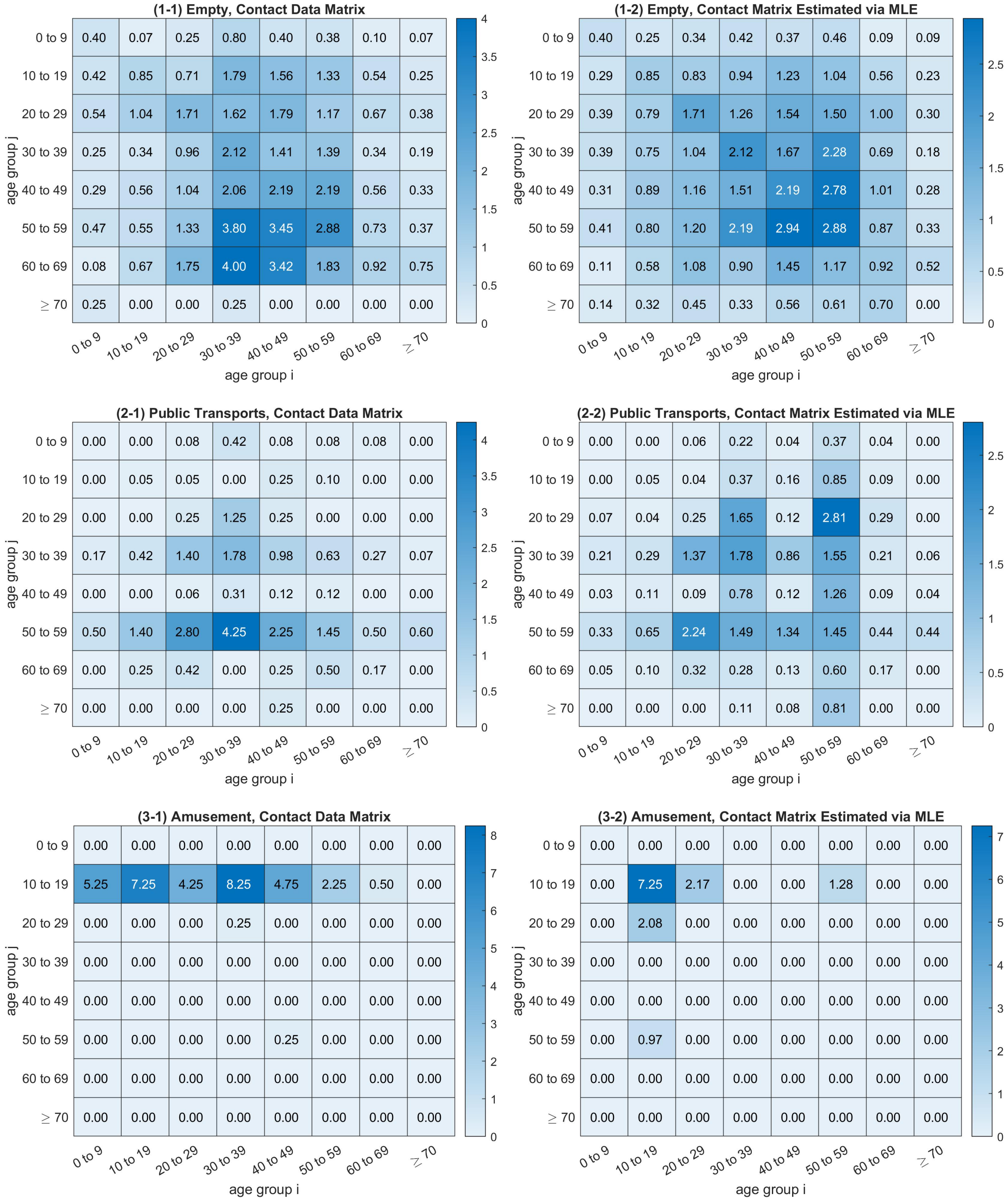
(1-1) Empty, Contact Data Matrix
0 to 9 0.40 0.07 0.25 0.80 0.40 0.38 0.10 0.07
10 to 19 0.42 0.85 0.71 1.79 1.56 1.33 0.54 0.25
20 to 29 0.54 1.04 1.71 1.62 1.79 1.17 0.67 0.38
30 to 39 0.25 0.34 0.96 2.12 1.41 1.39 0.34 0.19
40 to 49 0.29 0.56 1.04 2.06 2.19 2.19 0.56 0.33
50 to 59 0.47 0.55 1.33 3.80 3.45 2.88 0.73 0.37
60 to 69 0.08 0.67 1.75 4.00 3.42 1.83 0.92 0.75
≥ 70 0.25 0.00 0.00 0.25 0.00 0.00 0.00 0.00
(1-2) Empty, Contact Matrix Estimated via MLE
0 to 9 0.40 0.25 0.34 0.42 0.37 0.46 0.09 0.09
10 to 19 0.29 0.85 0.83 0.94 1.23 1.04 0.56 0.23
20 to 29 0.39 0.79 1.71 1.26 1.54 1.50 1.00 0.30
30 to 39 0.39 0.75 1.04 2.12 1.67 2.28 0.69 0.18
40 to 49 0.31 0.89 1.16 1.51 2.19 2.78 1.01 0.28
50 to 59 0.41 0.80 1.20 2.19 2.94 2.88 0.87 0.33
60 to 69 0.11 0.58 1.08 0.90 1.45 1.17 0.92 0.52
≥ 70 0.14 0.32 0.45 0.33 0.56 0.61 0.70 0.00
(2-1) Public Transports, Contact Data Matrix
0 to 9 0.00 0.00 0.08 0.42 0.08 0.08 0.08 0.00
10 to 19 0.00 0.05 0.05 0.00 0.25 0.10 0.00 0.00
20 to 29 0.00 0.00 0.25 1.25 0.25 0.00 0.00 0.00
30 to 39 0.17 0.42 1.40 1.78 0.98 0.63 0.27 0.07
40 to 49 0.00 0.00 0.06 0.31 0.12 0.12 0.00 0.00
50 to 59 0.50 1.40 2.80 4.25 2.25 1.45 0.50 0.60
60 to 69 0.00 0.25 0.42 0.00 0.25 0.50 0.17 0.00
≥ 70 0.00 0.00 0.00 0.00 0.25 0.00 0.00 0.00
(2-2) Public Transports, Contact Matrix Estimated via MLE
0 to 9 0.00 0.00 0.06 0.22 0.04 0.37 0.04 0.00
10 to 19 0.00 0.05 0.04 0.37 0.16 0.85 0.09 0.00
20 to 29 0.07 0.04 0.25 1.65 0.12 2.81 0.29 0.00
30 to 39 0.21 0.29 1.37 1.78 0.86 1.55 0.21 0.06
40 to 49 0.03 0.11 0.09 0.78 0.12 1.26 0.09 0.04
50 to 59 0.33 0.65 2.24 1.49 1.34 1.45 0.44 0.44
60 to 69 0.05 0.10 0.32 0.28 0.13 0.60 0.17 0.00
≥ 70 0.00 0.00 0.00 0.11 0.08 0.81 0.00 0.00
(3-1) Amusement, Contact Data Matrix
0 to 9 0.00 0.00 0.00 0.00 0.00 0.00 0.00 0.00
10 to 19 5.25 7.25 4.25 8.25 4.75 2.25 0.50 0.00
20 to 29 0.00 0.00 0.00 0.25 0.00 0.00 0.00 0.00
30 to 39 0.00 0.00 0.00 0.00 0.00 0.00 0.00 0.00
40 to 49 0.00 0.00 0.00 0.00 0.00 0.00 0.00 0.00
50 to 59 0.00 0.00 0.00 0.00 0.25 0.00 0.00 0.00
60 to 69 0.00 0.00 0.00 0.00 0.00 0.00 0.00 0.00
≥ 70 0.00 0.00 0.00 0.00 0.00 0.00 0.00 0.00
(3-2) Amusement, Contact Matrix Estimated via MLE
0 to 9 0.00 0.00 0.00 0.00 0.00 0.00 0.00 0.00
10 to 19 0.00 7.25 2.17 0.00 0.00 1.28 0.00 0.00
20 to 29 0.00 2.08 0.00 0.00 0.00 0.00 0.00 0.00
30 to 39 0.00 0.00 0.00 0.00 0.00 0.00 0.00 0.00
40 to 49 0.00 0.00 0.00 0.00 0.00 0.00 0.00 0.00
50 to 59 0.00 0.97 0.00 0.00 0.00 0.00 0.00 0.00
60 to 69 0.00 0.00 0.00 0.00 0.00 0.00 0.00 0.00
≥ 70 0.00 0.00 0.00 0.00 0.00 0.00 0.00 0.00
age group j
age group i
0 to 9
10 to 19
20 to 29
30 to 39
40 to 49
50 to 59
60 to 69
≥ 70

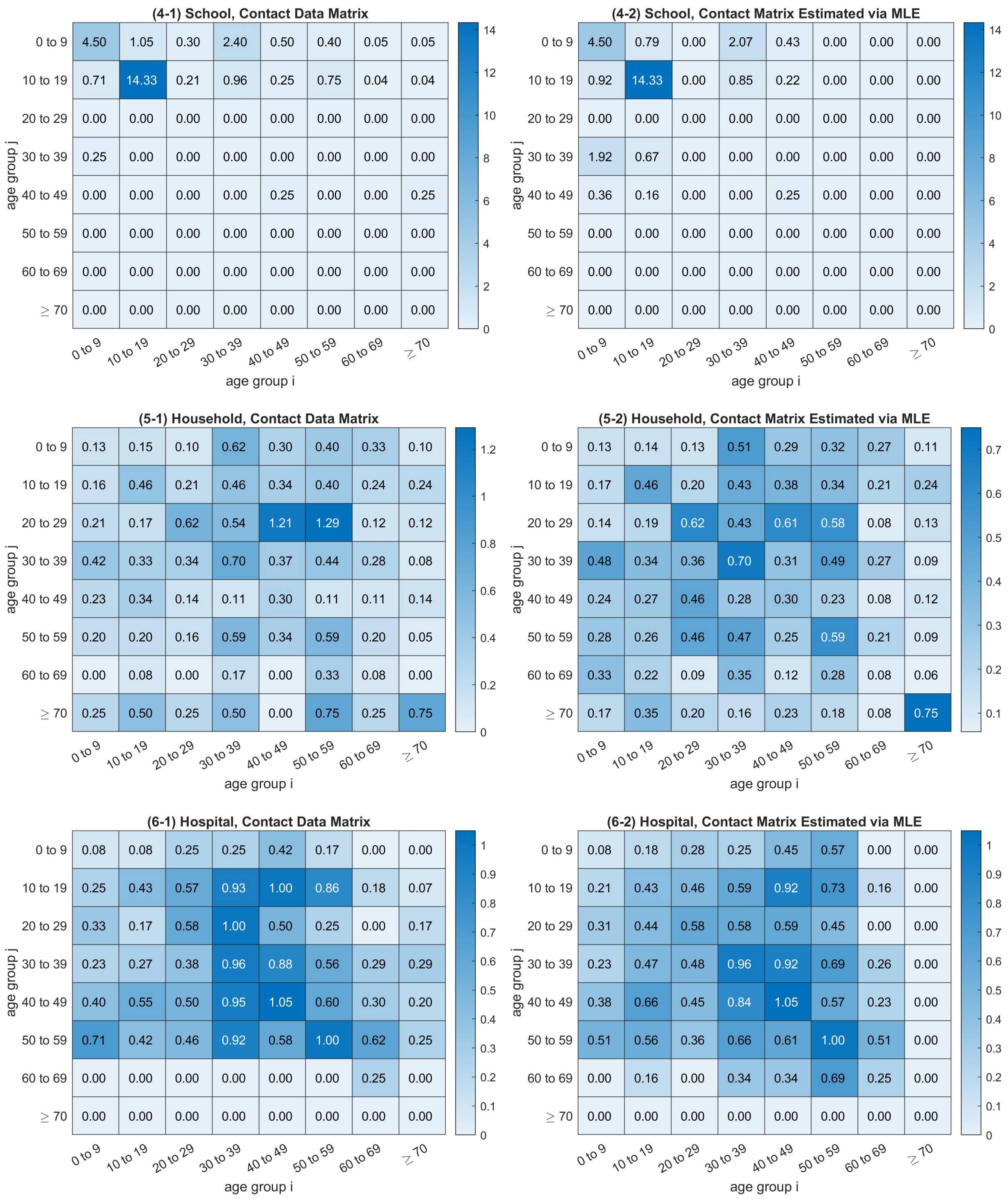
(4-1) School, Contact Data Matrix
0 to 9 4.50 1.05 0.30 2.40 0.50 0.40 0.05 0.05
10 to 19 0.71 14.33 0.21 0.96 0.25 0.75 0.04 0.04
20 to 29 0.00 0.00 0.00 0.00 0.00 0.00 0.00 0.00
30 to 39 0.25 0.00 0.00 0.00 0.00 0.00 0.00 0.00
40 to 49 0.00 0.00 0.00 0.00 0.25 0.00 0.00 0.25
50 to 59 0.00 0.00 0.00 0.00 0.00 0.00 0.00 0.00
60 to 69 0.00 0.00 0.00 0.00 0.00 0.00 0.00 0.00
≥ 70 0.00 0.00 0.00 0.00 0.00 0.00 0.00 0.00
0 to 9 10 to 19 20 to 29 30 to 39 40 to 49 50 to 59 60 to 69 ≥ 70
age group j
age group i
(4-2) School, Contact Matrix Estimated via MLE
0 to 9 4.50 0.79 0.00 2.07 0.43 0.00 0.00 0.00
10 to 19 0.92 14.33 0.00 0.85 0.22 0.00 0.00 0.00
20 to 29 0.00 0.00 0.00 0.00 0.00 0.00 0.00 0.00
30 to 39 1.92 0.67 0.00 0.00 0.00 0.00 0.00 0.00
40 to 49 0.36 0.16 0.00 0.00 0.25 0.00 0.00 0.00
50 to 59 0.00 0.00 0.00 0.00 0.00 0.00 0.00 0.00
60 to 69 0.00 0.00 0.00 0.00 0.00 0.00 0.00 0.00
≥ 70 0.00 0.00 0.00 0.00 0.00 0.00 0.00 0.00
0 to 9 10 to 19 20 to 29 30 to 39 40 to 49 50 to 59 60 to 69 ≥ 70
age group j
age group i
(5-1) Household, Contact Data Matrix
0 to 9 0.13 0.15 0.10 0.62 0.30 0.40 0.33 0.10
10 to 19 0.16 0.46 0.21 0.46 0.34 0.40 0.24 0.24
20 to 29 0.21 0.17 0.62 0.54 1.21 1.29 0.12 0.12
30 to 39 0.42 0.33 0.34 0.70 0.37 0.44 0.28 0.08
40 to 49 0.23 0.34 0.14 0.11 0.30 0.11 0.11 0.14
50 to 59 0.20 0.20 0.16 0.59 0.34 0.59 0.20 0.05
60 to 69 0.00 0.08 0.00 0.17 0.00 0.33 0.08 0.00
≥ 70 0.25 0.50 0.25 0.50 0.00 0.75 0.25 0.75
0 to 9 10 to 19 20 to 29 30 to 39 40 to 49 50 to 59 60 to 69 ≥ 70
age group j
age group i
(5-2) Household, Contact Matrix Estimated via MLE
0 to 9 0.13 0.14 0.13 0.51 0.29 0.32 0.27 0.11
10 to 19 0.17 0.46 0.20 0.43 0.38 0.34 0.21 0.24
20 to 29 0.14 0.19 0.62 0.43 0.61 0.58 0.08 0.13
30 to 39 0.48 0.34 0.36 0.70 0.31 0.49 0.27 0.09
40 to 49 0.24 0.27 0.46 0.28 0.30 0.23 0.08 0.12
50 to 59 0.28 0.26 0.46 0.47 0.25 0.59 0.21 0.09
60 to 69 0.33 0.22 0.09 0.35 0.12 0.28 0.08 0.06
≥ 70 0.17 0.35 0.20 0.16 0.23 0.18 0.08 0.75
0 to 9 10 to 19 20 to 29 30 to 39 40 to 49 50 to 59 60 to 69 ≥ 70
age group j
age group i
(6-1) Hospital, Contact Data Matrix
0 to 9 0.08 0.08 0.25 0.25 0.42 0.17 0.00 0.00
10 to 19 0.25 0.43 0.57 0.93 1.00 0.86 0.18 0.07
20 to 29 0.33 0.17 0.58 1.00 0.50 0.25 0.00 0.17
30 to 39 0.23 0.27 0.38 0.96 0.88 0.56 0.29 0.29
40 to 49 0.40 0.55 0.50 0.95 1.05 0.60 0.30 0.20
50 to 59 0.71 0.42 0.46 0.92 0.58 1.00 0.62 0.25
60 to 69 0.00 0.00 0.00 0.00 0.00 0.00 0.25 0.00
≥ 70 0.00 0.00 0.00 0.00 0.00 0.00 0.00 0.00
0 to 9 10 to 19 20 to 29 30 to 39 40 to 49 50 to 59 60 to 69 ≥ 70
age group j
age group i
(6-2) Hospital, Contact Matrix Estimated via MLE
0 to 9 0.08 0.18 0.28 0.25 0.45 0.57 0.00 0.00
10 to 19 0.21 0.43 0.46 0.59 0.92 0.73 0.16 0.00
20 to 29 0.31 0.44 0.58 0.58 0.59 0.45 0.00 0.00
30 to 39 0.23 0.47 0.48 0.96 0.92 0.69 0.26 0.00
40 to 49 0.38 0.66 0.45 0.84 1.05 0.57 0.23 0.00
50 to 59 0.51 0.56 0.36 0.66 0.61 1.00 0.51 0.00
60 to 69 0.00 0.16 0.00 0.34 0.34 0.69 0.25 0.00
≥ 70 0.00 0.00 0.00 0.00 0.00 0.00 0.00 0.00
0 to 9 10 to 19 20 to 29 30 to 39 40 to 49 50 to 59 60 to 69 ≥ 70
age group j
age group i

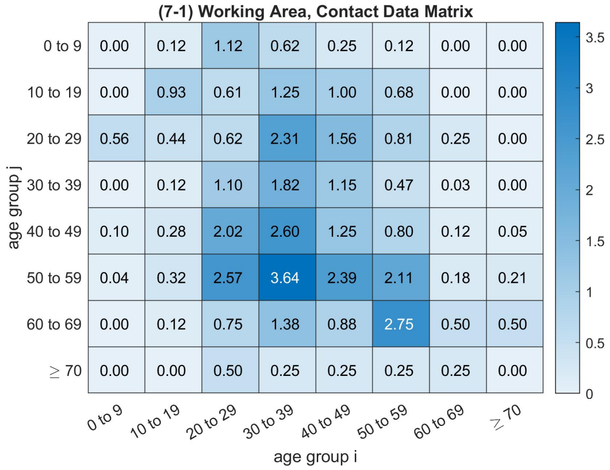
(7-1) Working Area, Contact Data Matrix
0 to 9 0.00 0.12 1.12 0.62 0.25 0.12 0.00 0.00
10 to 19 0.00 0.93 0.61 1.25 1.00 0.68 0.00 0.00
20 to 29 0.56 0.44 0.62 2.31 1.56 0.81 0.25 0.00
30 to 39 0.00 0.12 1.10 1.82 1.15 0.47 0.03 0.00
40 to 49 0.10 0.28 2.02 2.60 1.25 0.80 0.12 0.05
50 to 59 0.04 0.32 2.57 3.64 2.39 2.11 0.18 0.21
60 to 69 0.00 0.12 0.75 1.38 0.88 2.75 0.50 0.50
≥ 70 0.00 0.00 0.50 0.25 0.25 0.25 0.25 0.00
0 to 9 10 to 19 20 to 29 30 to 39 40 to 49 50 to 59 60 to 69 ≥ 70
age group i
age group j
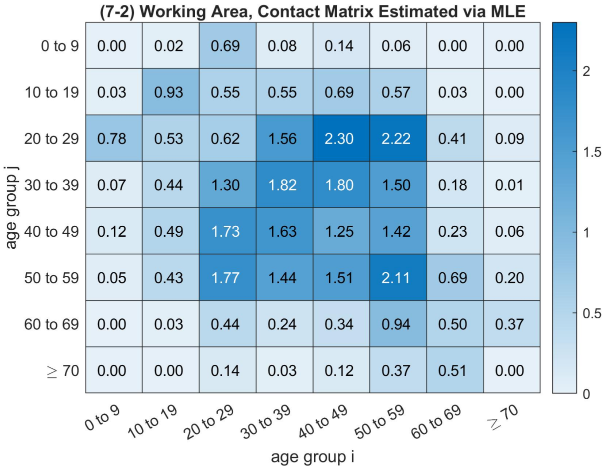
(7-2) Working Area, Contact Matrix Estimated via MLE
0 to 9 0.00 0.02 0.69 0.08 0.14 0.06 0.00 0.00
10 to 19 0.03 0.93 0.55 0.55 0.69 0.57 0.03 0.00
20 to 29 0.78 0.53 0.62 1.56 2.30 2.22 0.41 0.09
30 to 39 0.07 0.44 1.30 1.82 1.80 1.50 0.18 0.01
40 to 49 0.12 0.49 1.73 1.63 1.25 1.42 0.23 0.06
50 to 59 0.05 0.43 1.77 1.44 1.51 2.11 0.69 0.20
60 to 69 0.00 0.03 0.44 0.24 0.34 0.94 0.50 0.37
≥ 70 0.00 0.00 0.14 0.03 0.12 0.37 0.51 0.00
0 to 9 10 to 19 20 to 29 30 to 39 40 to 49 50 to 59 60 to 69 ≥ 70
age group i
age group j
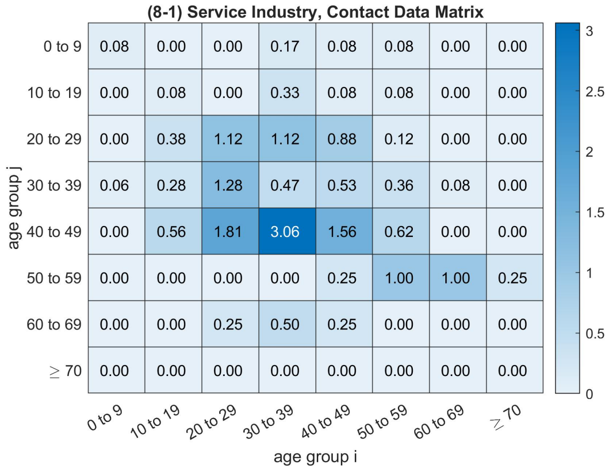
(8-1) Service Industry, Contact Data Matrix
0 to 9 0.08 0.00 0.00 0.17 0.08 0.08 0.00 0.00
10 to 19 0.00 0.08 0.00 0.33 0.08 0.08 0.00 0.00
20 to 29 0.00 0.38 1.12 1.12 0.88 0.12 0.00 0.00
30 to 39 0.06 0.28 1.28 0.47 0.53 0.36 0.08 0.00
40 to 49 0.00 0.56 1.81 3.06 1.56 0.62 0.00 0.00
50 to 59 0.00 0.00 0.00 0.00 0.25 1.00 1.00 0.25
60 to 69 0.00 0.00 0.25 0.50 0.25 0.00 0.00 0.00
≥ 70 0.00 0.00 0.00 0.00 0.00 0.00 0.00 0.00
0 to 9 10 to 19 20 to 29 30 to 39 40 to 49 50 to 59 60 to 69 ≥ 70
age group i
age group j
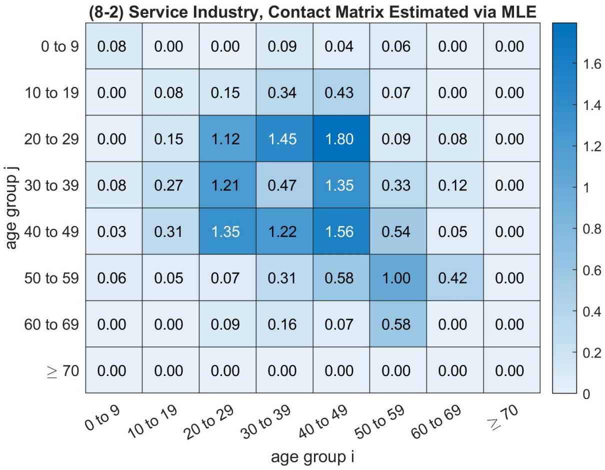
(8-2) Service Industry, Contact Matrix Estimated via MLE
0 to 9 0.08 0.00 0.00 0.09 0.04 0.06 0.00 0.00
10 to 19 0.00 0.08 0.15 0.34 0.43 0.07 0.00 0.00
20 to 29 0.00 0.15 1.12 1.45 1.80 0.09 0.08 0.00
30 to 39 0.08 0.27 1.21 0.47 1.35 0.33 0.12 0.00
40 to 49 0.03 0.31 1.35 1.22 1.56 0.54 0.05 0.00
50 to 59 0.06 0.05 0.07 0.31 0.58 1.00 0.42 0.00
60 to 69 0.00 0.00 0.09 0.16 0.07 0.58 0.00 0.00
≥ 70 0.00 0.00 0.00 0.00 0.00 0.00 0.00 0.00
0 to 9 10 to 19 20 to 29 30 to 39 40 to 49 50 to 59 60 to 69 ≥ 70
age group i
age group j
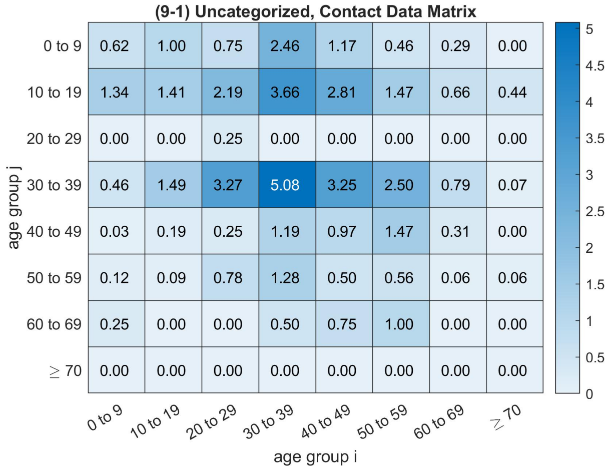
(9-1) Uncategorized, Contact Data Matrix
0 to 9 0.62 1.00 0.75 2.46 1.17 0.46 0.29 0.00
10 to 19 1.34 1.41 2.19 3.66 2.81 1.47 0.66 0.44
20 to 29 0.00 0.00 0.25 0.00 0.00 0.00 0.00 0.00
30 to 39 0.46 1.49 3.27 5.08 3.25 2.50 0.79 0.07
40 to 49 0.03 0.19 0.25 1.19 0.97 1.47 0.31 0.00
50 to 59 0.12 0.09 0.78 1.28 0.50 0.56 0.06 0.06
60 to 69 0.25 0.00 0.00 0.50 0.75 1.00 0.00 0.00
≥ 70 0.00 0.00 0.00 0.00 0.00 0.00 0.00 0.00
0 to 9 10 to 19 20 to 29 30 to 39 40 to 49 50 to 59 60 to 69 ≥ 70
age group i
age group j
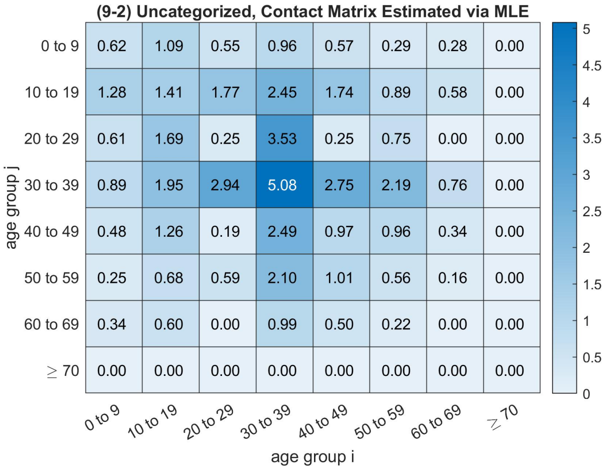
(9-2) Uncategorized, Contact Matrix Estimated via MLE
0 to 9 0.62 1.09 0.55 0.96 0.57 0.29 0.28 0.00
10 to 19 1.28 1.41 1.77 2.45 1.74 0.89 0.58 0.00
20 to 29 0.61 1.69 0.25 3.53 0.25 0.75 0.00 0.00
30 to 39 0.89 1.95 2.94 5.08 2.75 2.19 0.76 0.00
40 to 49 0.48 1.26 0.19 2.49 0.97 0.96 0.34 0.00
50 to 59 0.25 0.68 0.59 2.10 1.01 0.56 0.16 0.00
60 to 69 0.34 0.60 0.00 0.99 0.50 0.22 0.00 0.00
≥ 70 0.00 0.00 0.00 0.00 0.00 0.00 0.00 0.00
0 to 9 10 to 19 20 to 29 30 to 39 40 to 49 50 to 59 60 to 69 ≥ 70
age group i
age group j

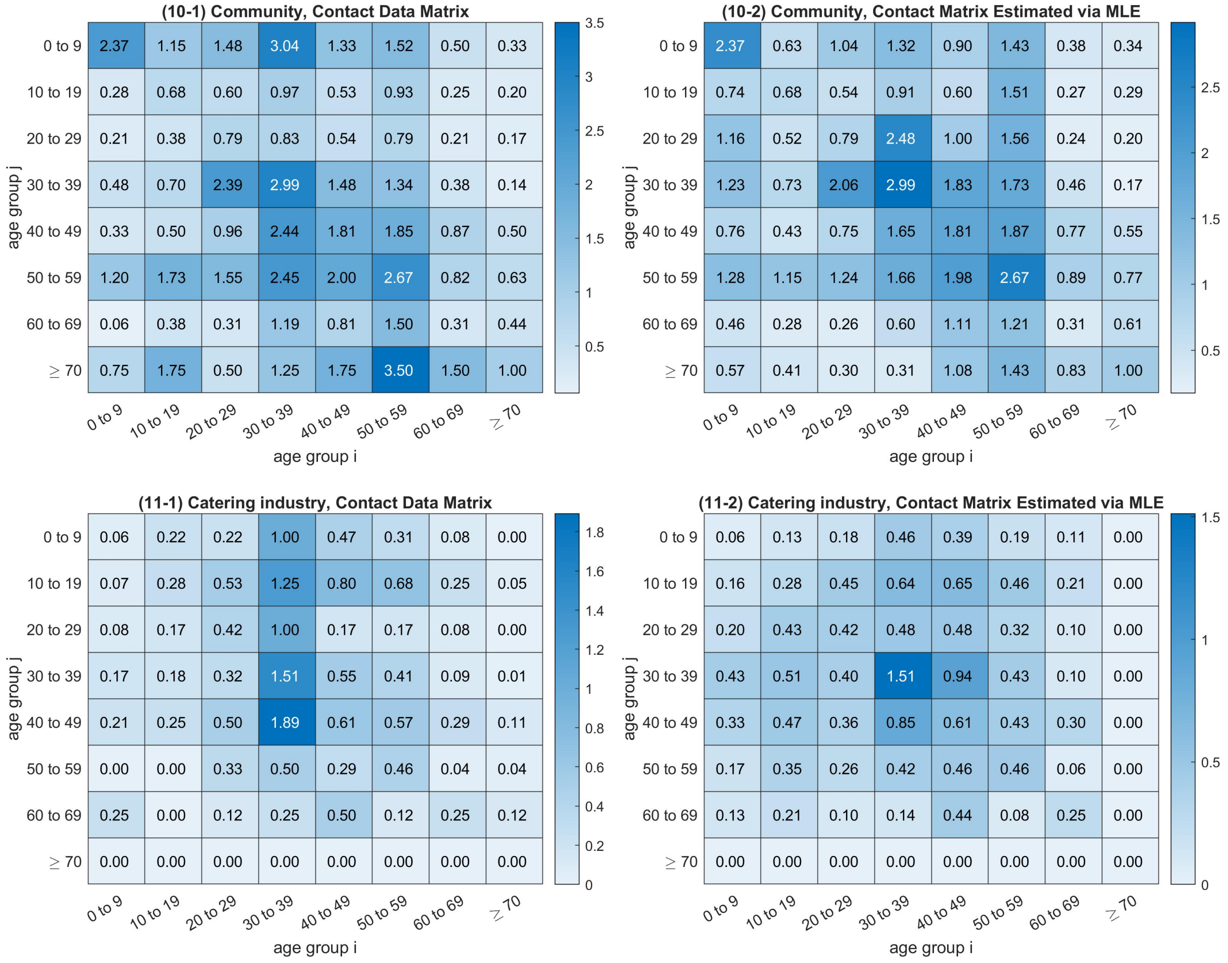

5.1 NPI Simulations

We consider the contact matrix under certain NPI condition as a linear combination of those 11 matrices. For example, school closure means the coefficient of the school component equals 0.
